# Photochemical syntheses, transformations, and bioorthogonal chemistry of *trans*-cycloheptene and sila *trans*-cycloheptene Ag(i) complexes†
†Electronic supplementary information (ESI) available: Full procedures, computational details and characterization data. CCDC 1583975 and 1583976. For ESI and crystallographic data in CIF or other electronic format see DOI: 10.1039/c7sc04773h


**DOI:** 10.1039/c7sc04773h

**Published:** 2018-01-08

**Authors:** Yinzhi Fang, Han Zhang, Zhen Huang, Samuel L. Scinto, Jeffrey C. Yang, Christopher W. am Ende, Olga Dmitrenko, Douglas S. Johnson, Joseph M. Fox

**Affiliations:** a Brown Laboratories , Department of Chemistry and Biochemistry , University of Delaware , Newark , DE 19716 , USA . Email: jmfox@udel.edu; b Pfizer Worldwide Research and Development , Cambridge , Massachusetts 02139 , USA; c Pfizer Worldwide Research and Development , Groton , Connecticut 06340 , USA

## Abstract

A photochemical synthesis of AgNO_3_ complexes of *trans*-cycloheptene (TCH) and *trans*-1-sila-4-cycloheptene (Si-TCH) derivatives is described. A low temperature flow photoreactor was designed to enable the synthesis of carbocyclic TCH derivatives due to their thermal sensitivity in the absence of metal coordination. Unlike the free carbocycles, TCH·AgNO_3_ complexes can be handled at rt and stored for weeks in the freezer (–18 °C). Si-TCH·AgNO_3_ complexes are especially robust, and are bench stable for days at rt, and for months in the freezer. X-ray crystallography was used to characterize a Si-TCH·AgNO_3_ complex for the first time. With decomplexation of AgNO_3_*in situ*, metal-free TCO and Si-TCH derivatives can engage in a range of cycloaddition reactions as well as dihydroxylation reactions. Computation was used to predict that Si-TCH would engage in bioorthogonal reactions that are more rapid than the most reactive *trans*-cyclooctenes. Metal-free Si-TCH derivatives were shown to display good stability in solution, and to engage in the fastest bioorthogonal reaction reported to date (*k*_2_ 1.14 × 10^7^ M^–1^ s^–1^ in 9 : 1 H_2_O : MeOH). Utility in bioorthogonal protein labeling in live cells is described, including labeling of GFP with an unnatural tetrazine-containing amino acid. The reactivity and specificity of the Si-TCH reagents with tetrazines in live mammalian cells was also evaluated using the HaloTag platform. The cell labeling experiments show that Si-TCH derivatives are best suited as probe molecules in the cellular environment.

## Abstract 2

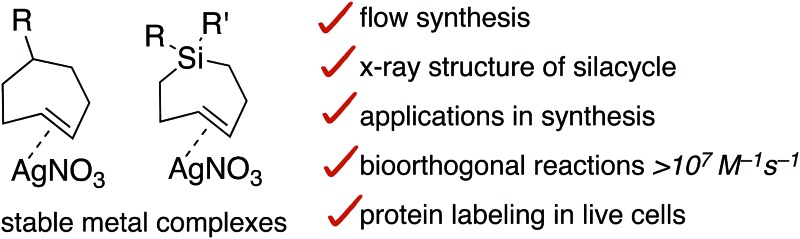
Synthesis and transformations of AgNO_3_ complexes of *trans*-cycloheptene (TCH) and *trans*-1-sila-4-cycloheptene (Si-TCH) derivatives are described.

## Introduction

For nearly seventy years,^1^–^3^ the unusual bonding, reactivity and planar chirality of *trans*-cycloalkenes have captured the imagination of scientists. The unique reactivity of *trans*-cycloalkenes has produced an impressive collection of applications in synthesis, including reactions with dienes,^4^–^6^ 1,3-dipoles^7^ and ketenes.^8^–^10^ Additionally, strained *trans*-cycloalkenes can serve as excellent ligands for transition metals. In the field of bioorthogonal chemistry,^11^–^19^ *trans*-cycloalkenes hold special significance due to their particularly fast kinetics in cycloaddition reactions.^20^–^23^ Relative to *trans*-cyclooctene, the chemistry of the homolog *trans*-cycloheptene is less explored. *trans*-Cycloheptene was first trapped with diphenylisobenzofuran by Corey and Winter from *trans*-1,2-cycloheptenethionocarbonate through treatment with P(OMe)_3_.^24^ Studies by Marshall,^25^ Kropp^26^ and Beauchemin^27^ on the photoprotonation reactions of cyclic alkenes, including cycloheptene, have shown that *cis*/*trans* equilibria could be driven by selective addition reactions of *trans*-cycloalkenes.

*trans*-Cycloheptene was first spectroscopically characterized by Inoue *via* singlet sensitized photoisomerization of *cis*-cycloheptene at –35 °C.^28^,^29^ Unlike *trans*-cyclooctene, which is stable at room temperature, *trans*-cycloheptene undergoes rapid isomerization under ambient conditions *via* a proposed ‘interrupted dimerization’ mechanism.^30^*trans*-Cycloheptene has also been prepared *via* ligand exchange from a *trans*-cycloheptene·CuOTf complex.^31^ 1-Phenyl-*trans*-cycloheptene and *trans*-cycloheptenone derivatives are also known to be thermally unstable at ambient temperature, but can be trapped *in situ*.^32^–^38^

While the parent *trans*-cycloheptene is thermally labile, it has been demonstrated in several studies that metal complexes can be isolated. CuOTf has been proposed to catalyze photodimerization reactions of cyclic olefins *via* photoinduced *cis*–*trans* isomerization,^39^ with predominant formation of a cyclotrimer from cycloheptene.^40^ A stable *trans*-cycloheptene·CuOTf complex has been prepared through irradiation of *cis*-cycloheptene·CuOTf, but a yield for the process was not reported.^41^ A pybox–RuCl_2_ complex of *trans*-cycloheptene has been prepared by irradiation of the corresponding ethylene complex in the presence of *cis*-cycloheptene under singlet sensitized conditions.^42^ Jendralla described the preparation of AgOTf and AgClO_4_ complexes of 3-methoxy-*trans*-cycloheptene and 6-methoxy-(*Z*),4(*E*)-cycloheptadiene.^43^,^44^ These compounds can be prepared through the Ag-mediated ring opening of a nitrosourea derivative of bicyclo[4.1.0]heptane. In unspecified yields, the AgClO_4_·3-methoxy-*trans*-cycloheptene complex was combined with a number of dienes to give the products of metal decomplexation and [4 + 2] cycloaddition.^43^,^44^

Because C–Si bonds are long, the inclusion of silicon into the cyclic backbone can alleviate olefinic strain and impart stability to *trans*-cycloalkenes.^45^–^52^ In 1997, (*E*)-1,1,3,3,6,6-hexamethyl-1-sila-4-cycloheptene was synthesized, resolved, and characterized crystallographically.^46^,^47^ Here, the exhaustive allylic substitution imparts a high degree of stability to the *trans*-alkene. Recent studies by Woerpel^48^–^51^ and Tomooka^52^ have provided demonstrations of the utility of backbone heteroatom-containing *trans*-cycloalkene derivatives in synthesis. Woerpel has elegantly developed a general method for the preparation of *trans*-oxasilacycloheptenes—seven membered rings that contain *trans*-alkenes and siloxy bonds in the backbone.^48^–^51^

There has been little investigation into the reaction chemistry of *trans*-cycloheptene derivatives. In 1980, Jendralla showed that silver complexes of 3-methoxy-*trans*-cycloheptene are isolable, and can reversibly dissociate and in unspecified yields undergo cycloaddition reactions.^43^,^44^ Woerpel has described selective addition reactions and difunctionalization reactions of *trans*-oxasilacycloheptenes, and recently has reported the Diels–Alder reactions of *trans*-oxasilacycloheptenes with furan and tetrazine derivatives.^48^–^51^ At 25 °C in benzene, an oxasilacycloheptene was 7-fold more reactive toward 2,5-diphenylisobenzofuran than a conformationally strained *trans*-cyclooctene (‘s-TCO’) derivative.^48^ An oxasilacycloheptene derivative was shown to react with 3,6-diphenyl-*s*-tetrazine in benzene at rt in less than 10 min and in 90% NMR yield.^48^

Our group has described a closed-loop flow reactor for the synthesis of *trans*-cyclooctene derivatives, whereby selective complexation with AgNO_3_ is used to drive the formation of *trans*-isomer from *cis*-cyclooctene.^21^,^53^–^55^ Described herein is an approach to the synthesis of *trans*-cycloheptene (TCH) and *trans*-1-sila-4-cycloheptene (Si-TCH) derivatives *via* flow photochemical synthesis. The derivatives described are especially stable as their AgNO_3_ metal complexes, which can be stored neat or in solution for long periods. With decomplexation of AgNO_3_*in situ*, metal-free TCH and Si-TCH derivatives can engage in a range of cycloaddition reactions as well as dihydroxylation reactions. Unlike the carbocycles, Si-TCH derivatives display good stability in solution and are shown to engage in the fastest bioorthogonal reaction reported to date. Decomplexation of AgNO_3_ can be carried out *in situ* directly in cell media for bioorthogonal protein labeling in live cells.

## Results and discussion

For the photochemical synthesis of *trans*-cyclooctene derivatives, the *trans*-cycloalkene is first scavenged on AgNO_3_/SiO_2_ and then liberated through treatment with aqueous or methanolic ammonia.^21^,^53^–^55^ However, our attempts to directly apply this procedure to the synthesis of carbocyclic TCHs was unsuccessful, most likely due to the susceptibility of carbocyclic TCHs to readily isomerize to their *cis*-isomers.^30^ Recently, we demonstrated that the shelf-life of conformationally strained *trans*-cyclooctenes could be enhanced by storing the cycloalkenes as their AgNO_3_ complexes, and that the free alkenes could be liberated *in situ* through treatment with NaCl in aqueous solution or cell media.^56^ We reasoned that TCH and Si-TCH derivatives may also be isolable and better stored as their Ag-complexes, and that the corresponding free-alkenes could be liberated at later time points as required.

**Fig. 1:**
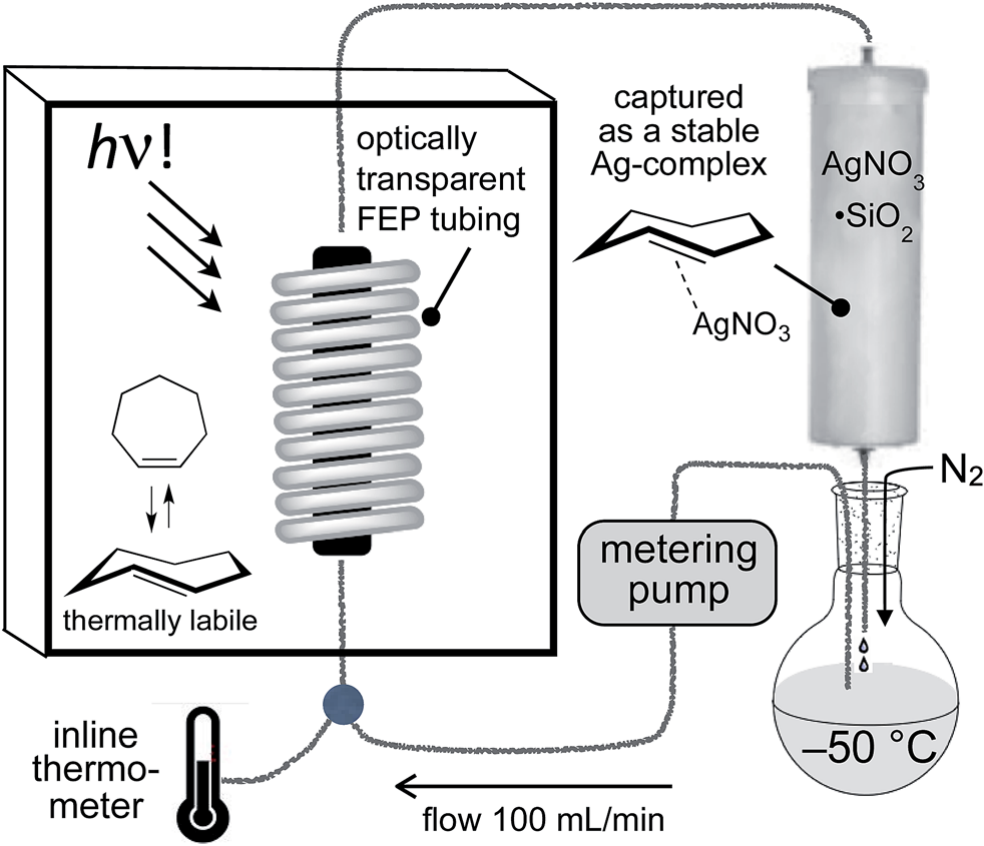
The apparatus for photoisomerization of carbocyclic *trans*-cycloheptene derivatives was designed to minimize loss of *trans*-cycloheptene due to thermal isomerization by using FEP tubing and inline cooling. For the synthesis of *trans*-1-sila-4-cycloheptenes, photoisomerizations could be carried out at rt using a conventional flow photoisomerization setup.

Photoisomerizations to form Si-TCH·AgNO_3_ derivatives were carried out at rt using the previously described flow-photoisomerization apparatus,^21^,^53^,^54^ with the modification that Si-TCH·AgNO_3_ complexes were directly isolated from SiO_2_ without Ag-decomplexation. The metal complexes that were obtained were stable in neat form for >1 month in the freezer. However, it was necessary to alter our reactor design for the synthesis of carbocyclic *trans*-cycloheptenes due to their thermal lability (Fig. 1). As with the standard photoreactor, this system uses methyl benzoate as a sensitizer and a metering pump to pass a solution of substrate at a rate of 100 mL min^–1^ through a photowell and then through a column of AgNO_3_·SiO_2_, where the *trans*-cycloalkene is selectively captured as a AgNO_3_ complex. In the reactor for carbocyclic TCH synthesis, a reservoir of solvent chilled in a cold bath (–50 °C) was positioned before the photowell, and the photoisomerization was conducted in a coil of optically transparent FEP tubing.^57^ The fluoropolymer tubing provides a high surface area and minimal volume (only 30 mL for 8 m tubing) thereby minimizing the residence time before product adsorption on AgNO_3_/SiO_2_. In our standard setup, an inline thermometer was included to measure the temperature for the flowing mixture either before or just after the UV lamp. The temperature was measured as 0 °C before entering the Rayonet photoreactor, and as 20 °C after exiting the photoreactor. With this apparatus, TCH·AgNO_3_ complexes were eluted from the column, and isolated as semisolids that are moderately stable at rt but stable for weeks in the freezer.

**Scheme 1:**
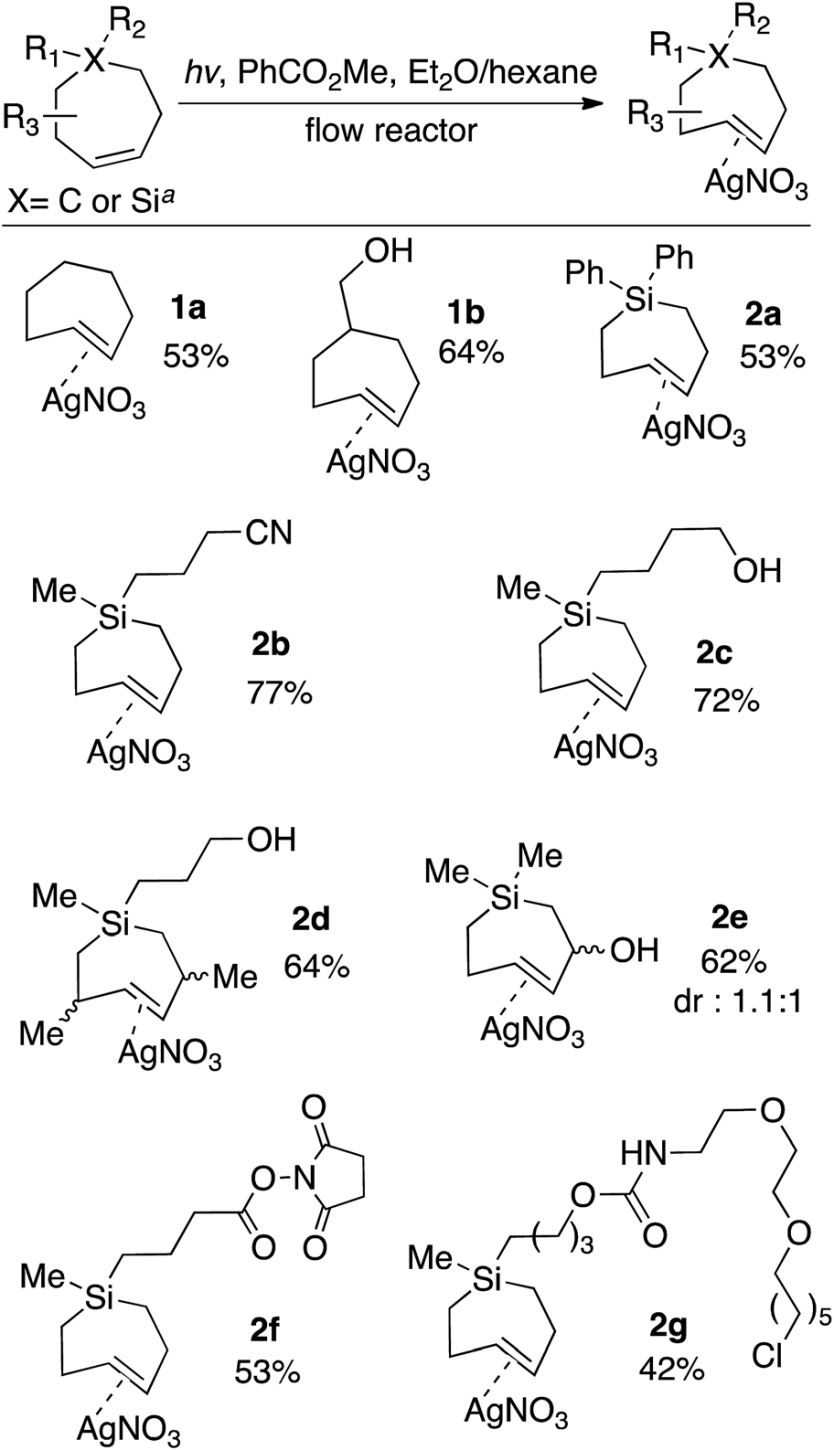
Flow-photochemical synthesis of AgNO_3_ complexes of *trans*-cycloheptenes and *trans*-1-sila-4-cycloheptenes. TCH·AgNO_3_ and Si-TCH·AgNO_3_ complexes were isolated as semisolids that contained 20–30% free AgNO_3_. Yields are the average of two runs, and were corrected by measuring the ^1^H NMR against an internal standard. ^*a*^Photoisomerization of carbocycles were conducted at low temperature using the flow apparatus described in Fig. 1. Photoisomerizations of silicycles were conducted at room temperature using the flow apparatus previously described for *trans*-cyclooctene synthesis.

The scope of TCH and Si-TCH synthesis is shown in Scheme 1. With the exception of cycloheptene itself, the carbocyclic and sila-cycloheptene precursors were prepared in 2–7 steps using olefin metathesis as a key step (ESI†). Silver nitrate complexes of *trans*-cycloheptene (**1a**) and *trans*-5-hydroxymethylcycloheptene (**1b**) were prepared in 53% and 64% yields, respectively. These TCH·AgNO_3_ complexes are stable enough to handle on the bench for modest periods (hours), and to longer-term storage in the freezer (–18 °C). NMR monitoring showed 90% fidelity for a CD_3_OD solution of **1a** after 10 days storage in freezer (–18 °C), and 92% fidelity for a CD_3_OD solution of **1a** after 10 hours at rt on the bench.

The silver complexes of Si-TCH are much more stable. NMR monitoring showed 93% fidelity for a CD_3_OD solution of **2a** after 8 days at rt, and 96% fidelity for **2a** after storage for 1 month in the freezer (–18 °C, neat). The photoisomerization method could be used to produce diphenyl (**2a**) or dialkyl (**2b–g**) substituted silacycles. Cyano (**2b**) and hydroxyl (**2c–e**) groups were tolerated, as were NHS ester (**2f**) and chloroalkane (**2g**) groups that could be used to enable conjugation to fluorophores and HaloTag^58^,^59^ fusion proteins, respectively. TCH·AgNO_3_ and Si-TCH·AgNO_3_ complexes were isolated as semisolids that contained 20–30% free AgNO_3_. The isolated yields were corrected by measuring the ^1^H NMR against an internal standard.

**Fig. 2:**
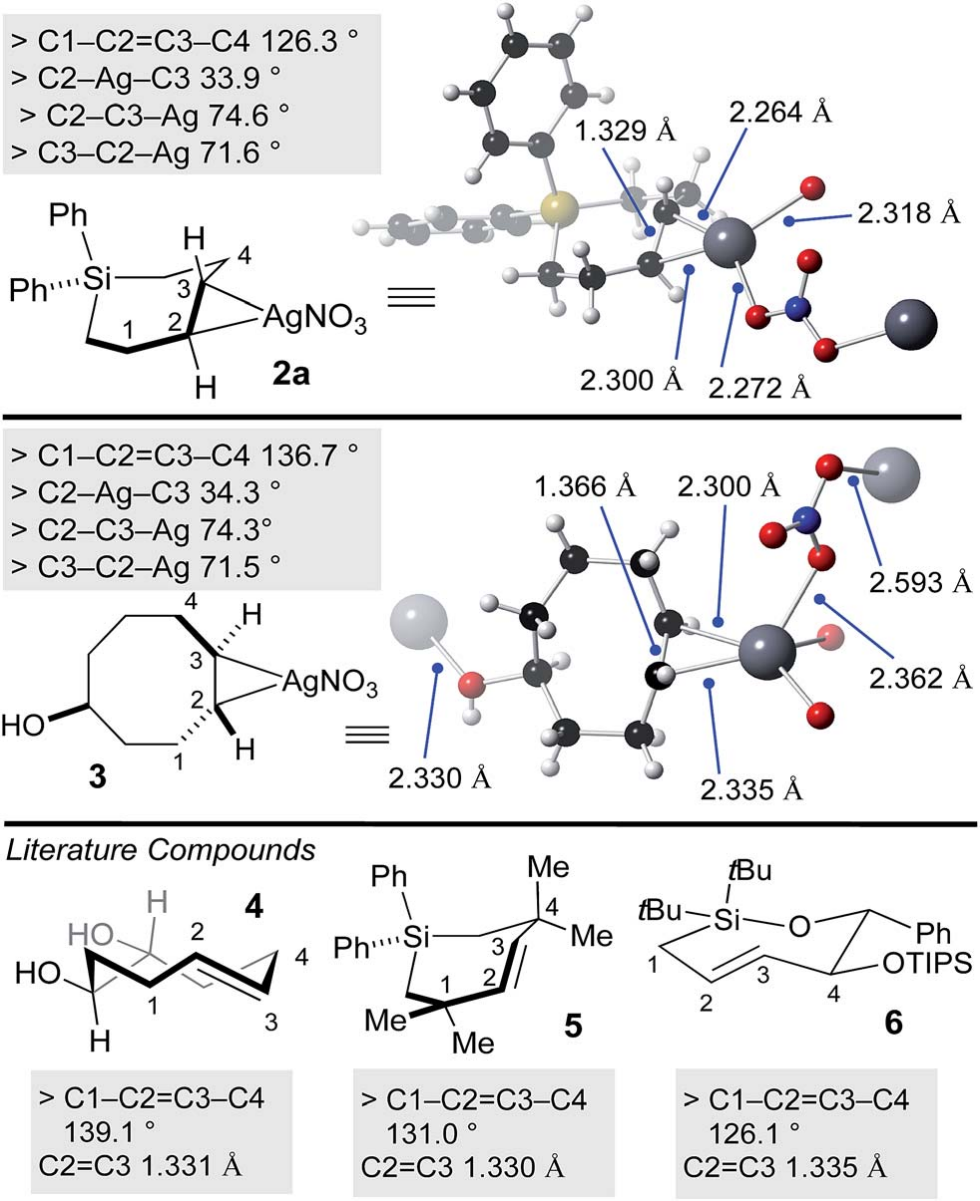
X-ray crystal structures of SiTCH·AgNO_3_ and TCO·AgNO_3_ complexes, with comparisons to known metal-free *trans*-cycloalkenes that have been crystallographically characterized.

Previously, a crystal structure showed that *trans*-cyclooctene coordinates with AgNO_3_ as a 1 : 1 complex.^60^ X-ray quality crystals of silver complex **2a** were grown from ethyl acetate/methanol. Selected bond lengths and angles are displayed in Fig. 2. The coordination environment at silver is distorted trigonal, with bridging coordination of nitrite in an extended polymeric structure in the solid state. For comparison, we also grew crystals of the silver(i) nitrate complex of the equatorial diastereomer of 5-hydroxy-*trans*-cyclooctene **3** (Fig. 2). Here, the coordination environment at silver is distorted tetrahedral due to the ability of the hydroxyl to serve as a second bridging ligand. The C–Ag bond lengths and bond angles were similar for **2a** and **3**. As expected, the C–C=C–C dihedral angle for **3** (136.7°) is smaller than metal-free TCO **4** (139.1°).^53^ Similarly, the C–C=C–C dihedral angle for **2a** (126.3°) was smaller than that of metal free Si-*trans*-cycloheptene **5** (130.9°),^46^ but comparable to **6** (126.1 °C)^50^—a compound with additional strain due to relatively short C–O and Si–O bonds in the cyclic backbone. Reflecting the low level of metal backbonding that is common for Ag(i) alkene complexes, the C=C bond length for the Ag(i) complexes (1.329 Å for **2a**, 1.366 Å for **3**) was very similar to that of the metal-free complexes **4–6** (1.331–1.335 Å).

Silver-free Si-TCH compounds could be prepared by treating their corresponding silver nitrate complexes with an excess of aq. NH_4_OH or aq. NaCl, followed by extraction with organic solvent. For example, silver complex **2a** upon treatment with aq. NH_4_OH was extracted with C_6_D_6_ to give a solution of Si-TCH **7a** (98% *trans* isomer). Consistent with previous reports on a biomolecular mechanism for TCH isomerization,^30^ variable amounts (20–30%) of the *cis*-isomer of **7a** was observed when **7a** was concentrated to dryness on the rotovap. However, **7a** displayed high stability when maintained in solution, with only 8% isomerization observed for a 100 mM solution of **7a** that was stored for 24 hours at room temperature, and <5% isomerization for a similar solution that was stored for 24 hours in a freezer (–20 °C).

**Fig. 3:**
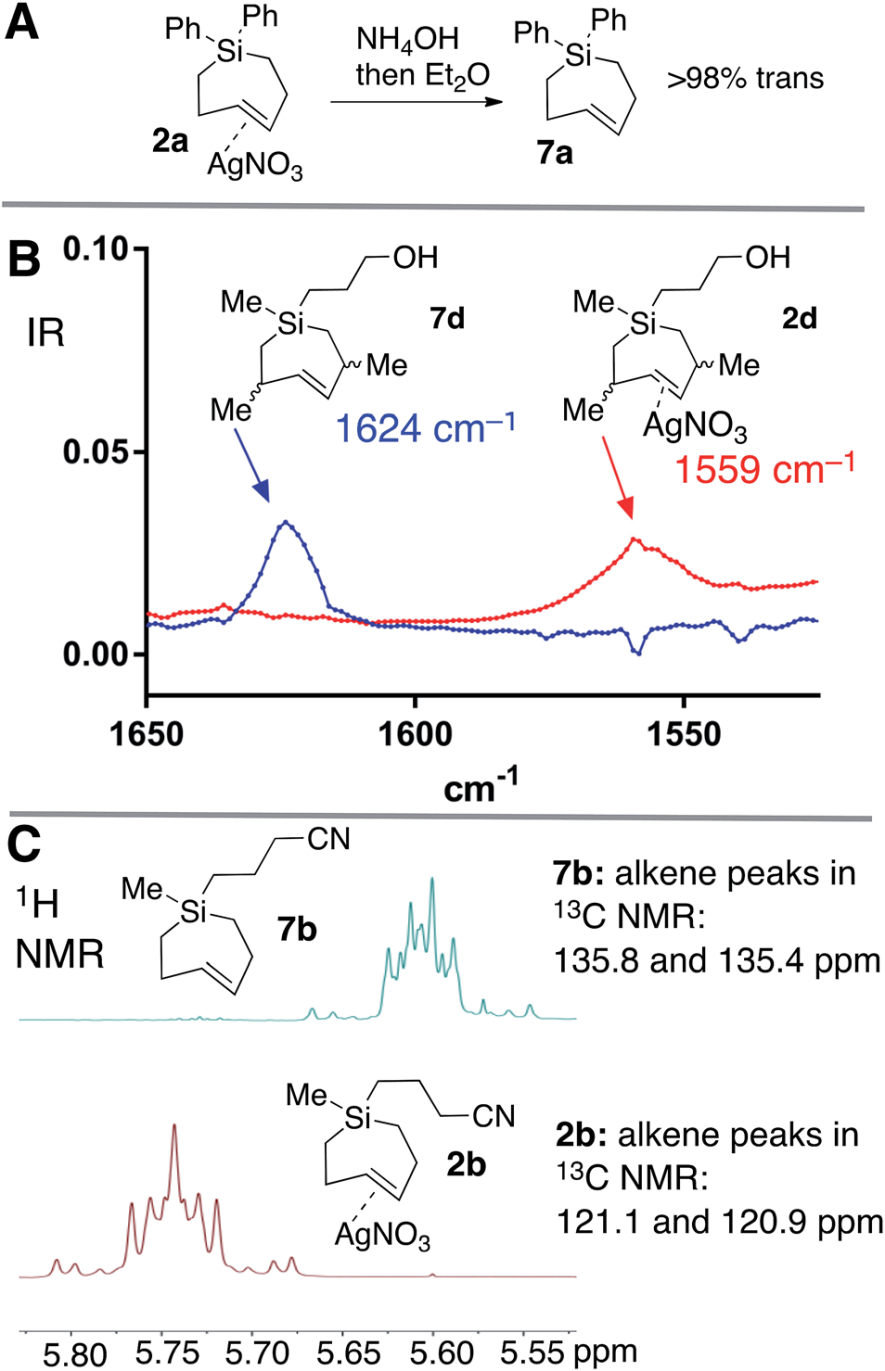
(a) Decomplexation of AgNO_3_ with NH_4_OH gives metal-free Si-TCH **7a** which is stable to overnight storage in solution. Aq. NaCl can also be used to liberate metal from SiTCH·AgNO_3_ complexes. The metal-free complexes are stable in solution, but give variable amounts of isomerization when concentrated to dryness. (b) FT-IR spectrum of the C=C stretches of metal complex **2d** and free alkene **7d**. (c) Diagnostic shifts in alkene peaks in the ^1^H and ^13^C NMR spectra of metal complex **2b** and metal-free **7b**.

Ag(i)-complex **2d** could also be freed of metal to give alkene **7d** as a mixture of diastereomers (Fig. 3A). The allylic substituents of **7d** protect the alkene from biomolecular chemistry, and unlike most other metal-free Si-TCH compounds, **7d** is stable when stored in neat form and can be characterized by FT-IR (Fig. 3B). The weak C=C double bond stretch of **7d** at 1624 cm^–1^ is shifted to 1559 cm^–1^ for the Ag(i) complex **2d**. This 65 cm^–1^ shift is consistent both in magnitude and direction for a Ag(i) alkene complex.^61^ Finally, we noted that Ag(i)-complexation leads to signature shifts of alkene resonances in both the ^1^H and ^13^C NMR spectra (Fig. 3C). For example, alkene resonances in the ^1^H NMR spectrum of metal complex **2b** were shifted downfield relative to metal-free **7b** by 0.15 ppm, while in ^13^C NMR spectra alkene resonances of **2b** were shifted up field by ∼16 ppm. In the ^1^H NMR spectra, complexity arises due to the higher order effects for a ddd couplet.

**Scheme 2:**
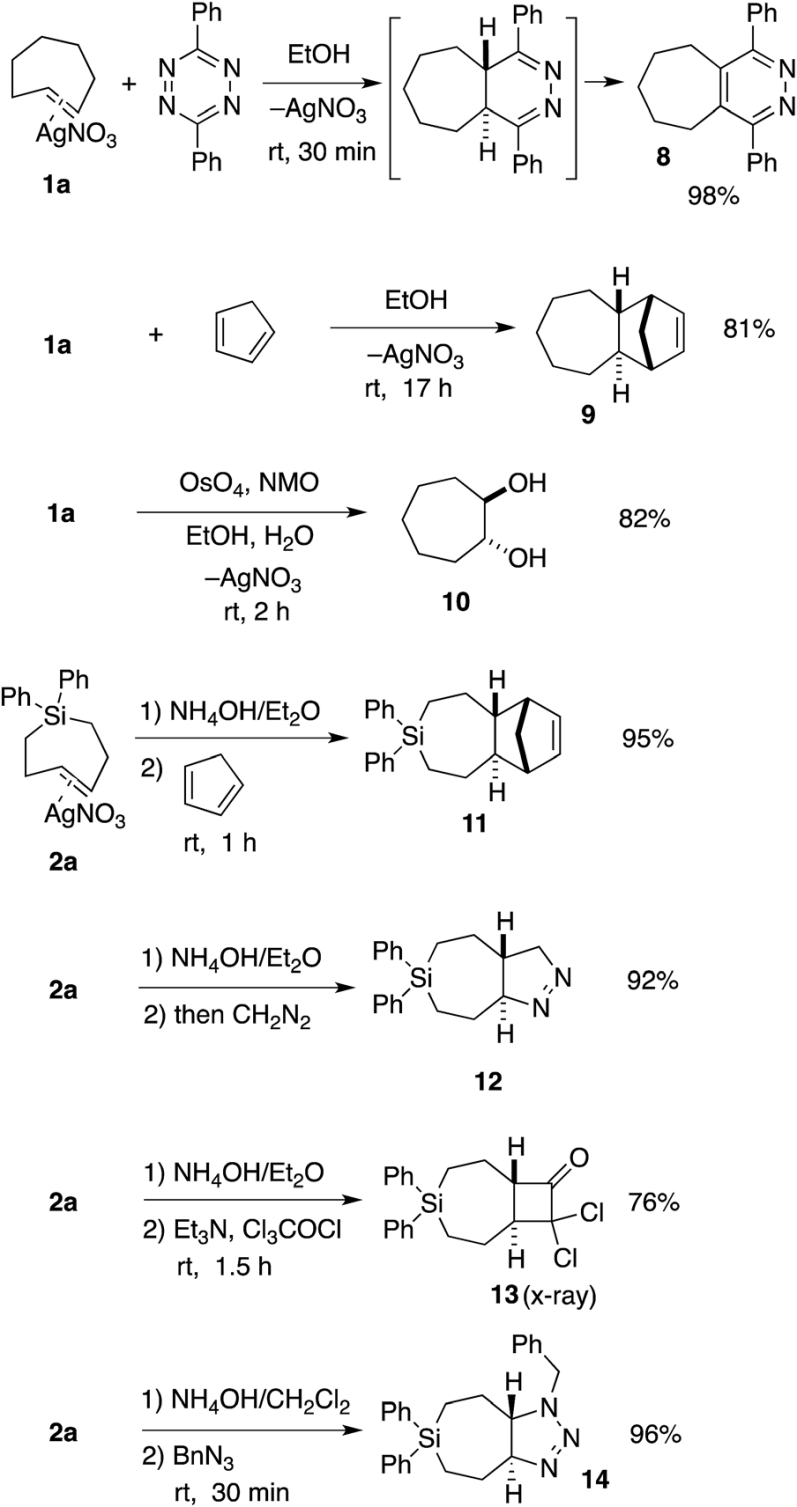
Reactions of TCH **1a** and Si-TCH **2a**. ^*a*^Yields represent isolated yields as average of two runs.

TCH **1a** and Si-TCH **2a** were shown to engage in a range of reactions as shown in Scheme 2. Metal complex **1a** was directly combined with 3,6-diphenyl-1,2,4,5-tetrazine to give pyridazine **8**—the product of metal dissociation, Diels–Alder/retro-Diels–Alder, and oxidation, in 98% yield. Cyclopenta-1,3-diene was also used to trap *trans*-cycloheptene, delivering the [4 + 2] cycloaddition adduct **9** in 81% yield as a single diastereomer. We also investigated the vicinal dihydroxylation of **1a**, and found that catalytic OsO_4_ and NMO gave **10** in 82% yield as a single diastereomer. The observation that the dihydroxylation of **1a** is stereospecific is in line with earlier observations by Cope with *trans*-cyclooctene.^2^

Si-TCH·AgNO_3_ complex **2a** could be freed from silver by treatment with NH_4_OH (Scheme 2), and subsequently combined with cyclopentadiene, diazomethane, dichloroketene, and benzyl azide to provide the cycloadducts **11–14** in 76–96% yields (Scheme 2). In each case, a single diastereomer was obtained. Attempts to combine *cis*-1-diphenylsila-4-cycloheptene (the *cis*-isomer of **7a**) with dichloroketene, benzylazide or diazomethane only returned unreacted starting material. An X-ray structure was obtained for the dichloroketene adduct **13** (ESI†).

**Scheme 3:**
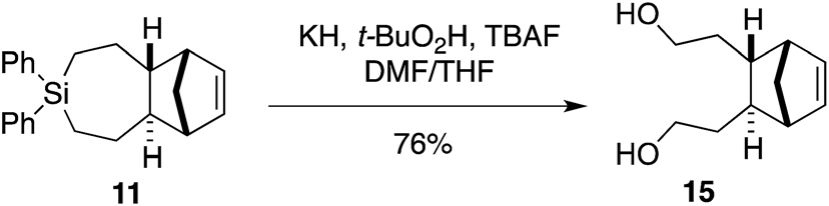
Fleming–Tamao oxidation.

We sought to demonstrate that Si-TCH cycloadducts could be oxidized to give formal cycloadducts of 1,2,-dialkylolefins—which are recalcitrant substrates in intermolecular Diels–Alder reactions.^62^ With the cycloadduct **11**, we demonstrated that the diphenylsila-group could be oxidized to a diol-product. Thus **11** was subjected to Tamao–Fleming reaction under Woerpel's conditions^63^ to give the unknown diol product **15** in 76% yield (Scheme 3).

**Scheme 4:**
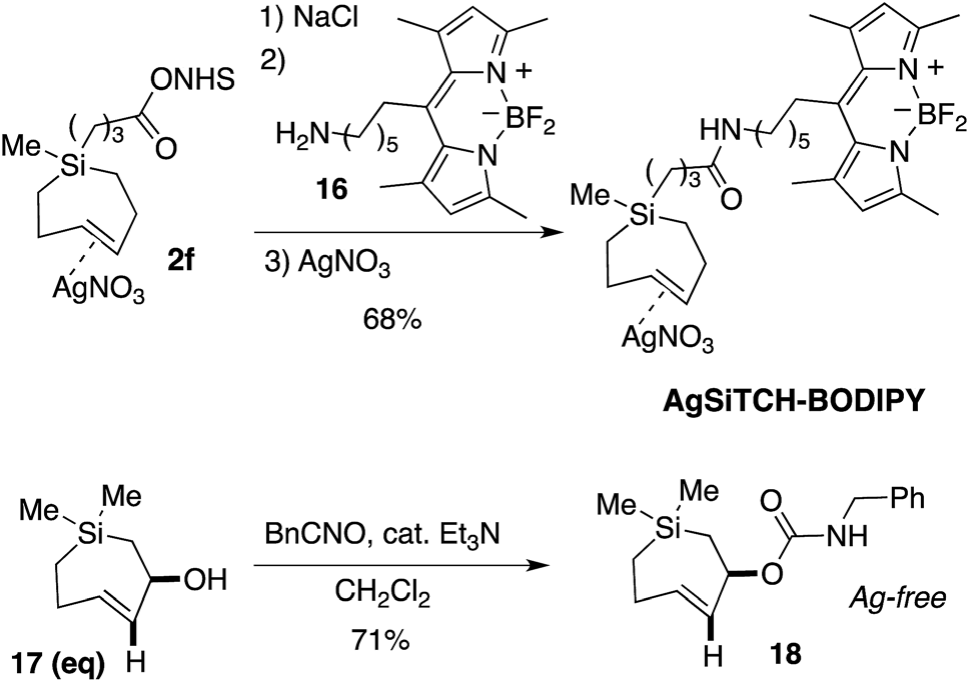
Transformations of Si-TCH derivatives.

We further demonstrated that NHS-ester **2f** could be modified though coupling to a BODIPY-fluorophore conjugate. As shown in Scheme 4, NHS ester **2f** could be decomplexed from AgNO_3_ by treatment with brine and extraction into CH_2_Cl_2_. The resulting free Si-TCH was then conjugated to an aminohexyl BODIPY **16**, and the resulting conjugate was isolated and stored as a AgNO_3_ complex (**AgSiTCH-BODIPY**). As discussed below, this fluorophore conjugate finds utility for bioorthogonal labeling in live cells. We also demonstrated that the equatorial allylic alcohol **17(eq)**, derived from Ag-complex **2e**, could be elaborated to the carbamate **18** through treatment with benzylisocyanate (Scheme 4). *trans*-Cyclooctenes with allylic carbamate leaving groups have been used by Robillard^64^,^65^ and Chen^66^,^67^ for the tetrazine-ligation initiated decaging of doxorubicin and other cargo molecules. The 7-membered analog **18** is particularly stable as the Ag-free *trans*-cycloalkene, and can be handled neat and stored without AgNO_3_ in the freezer for long periods. Efforts to synthesize and explore the ability of analogs of **18** to function for payload release is a topic of ongoing study.

**Scheme 5:**
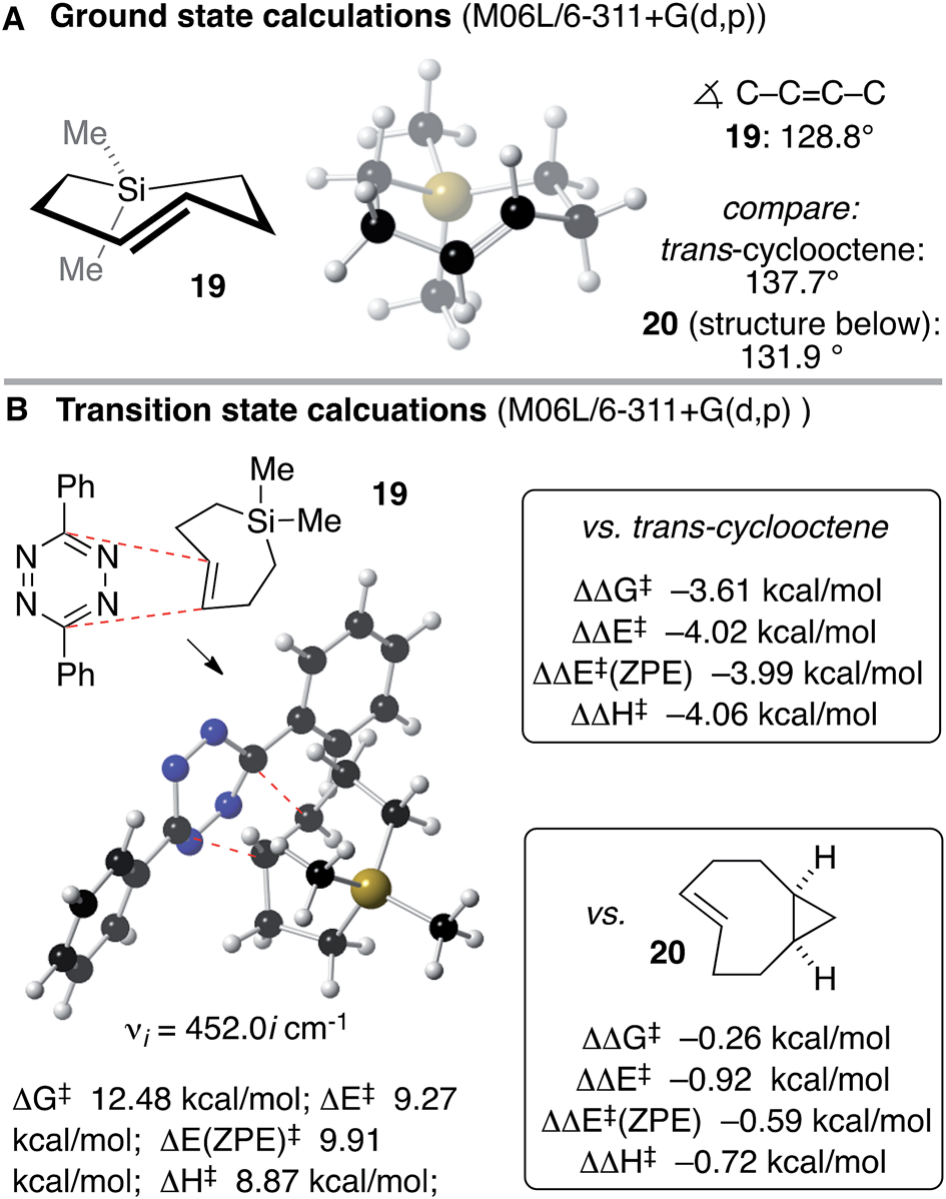
(A) Ground state calculations on Si-TCH **19** show that the <C–C=C–C dihedral angle is more distorted than that of *trans*-cyclooctene or the conformationally constrained **20**. (B) Transition state calculations predict that Si-TCH **19** would be more reactive than *trans*-cyclooctene and **20**.

Anticipating that Si-TCH compounds may be useful in bioorthogonal chemistry, computation was used to study the structure and Diels–Alder reactivity of Si-TCH **19** (Scheme 5). We compared **19** to the parent *trans*-cyclooctene and a conformationally strained ‘s-TCO’ derivative **20** that was previously studied in our labs.^21^,^54^ We reasoned that the 7-membered ring in the backbone of **19** would augment the olefinic strain of the *trans*-cycloalkene, and thereby increase the reactivity in tetrazine ligation. Previously, thiacycloheptynes and dibenzoselenocycloheptynes have been studied in bioorthogonal chemistry.^68^ As shown in Scheme 5A, ground state calculations were carried out for Si-TCH **19** at the M06L/6-311+G(d,p) level, and compared to previous calculations on *trans*-cyclooctene and s-TCO **20**.^21^,^54^ The calculated C–C=C–C dihedral angle for **19** (128.8°) is smaller than that for *trans*-cyclooctene (137.7°) or **20** (131.9°). M06L/6-311+G(d,p) calculations were also carried out to compare the Diels–Alder reactivity of Si-TCH **19** to *trans*-cyclooctene and s-TCO **20** (Scheme 5B). These calculations were carried out with diphenyl-*s*-tetrazine so that they could be benchmarked against previous calculations.^21^,^54^ For Si-TCH **19**, the calculated barriers relative to a pre-reaction complex for the Diels–Alder reaction with 3,6-diphenyl-*s*-tetrazine are Δ*G*^‡^ 12.48 kcal mol^–1^; Δ*E*^‡^ 9.27 kcal mol^–1^, Δ*E*^‡^ (ZPE) 9.91 kcal mol^–1^ and Δ*H*^‡^ 8.87 kcal mol^–1^. The barrier is significantly lower than that of *trans*-cyclooctene with 3,6-diphenyl-*s*-tetrazine (ΔΔ*G*^‡^ –3.61 kcal mol^–1^, ΔΔ*E*^‡^ –4.02 kcal mol^–1^, ΔΔ*E*^‡^ (ZPE) –3.99 kcal mol^–1^, ΔΔ*H*^‡^ 4.06 kcal mol^–1^). The barrier is also lower than that calculated for s-TCO **20** (ΔΔ*G*^‡^ –0.26 kcal mol^–1^, ΔΔ*E*^‡^ –0.92 kcal mol^–1^, ΔΔ*E*^‡^ (ZPE) –0.59 kcal mol^–1^, ΔΔ*H*^‡^ –0.72 kcal mol^–1^). The computations prompted us to conduct experimental investigations into the utilization of Si-TCH complexes for applications in bioorthogonal chemistry.

**Scheme 6:**
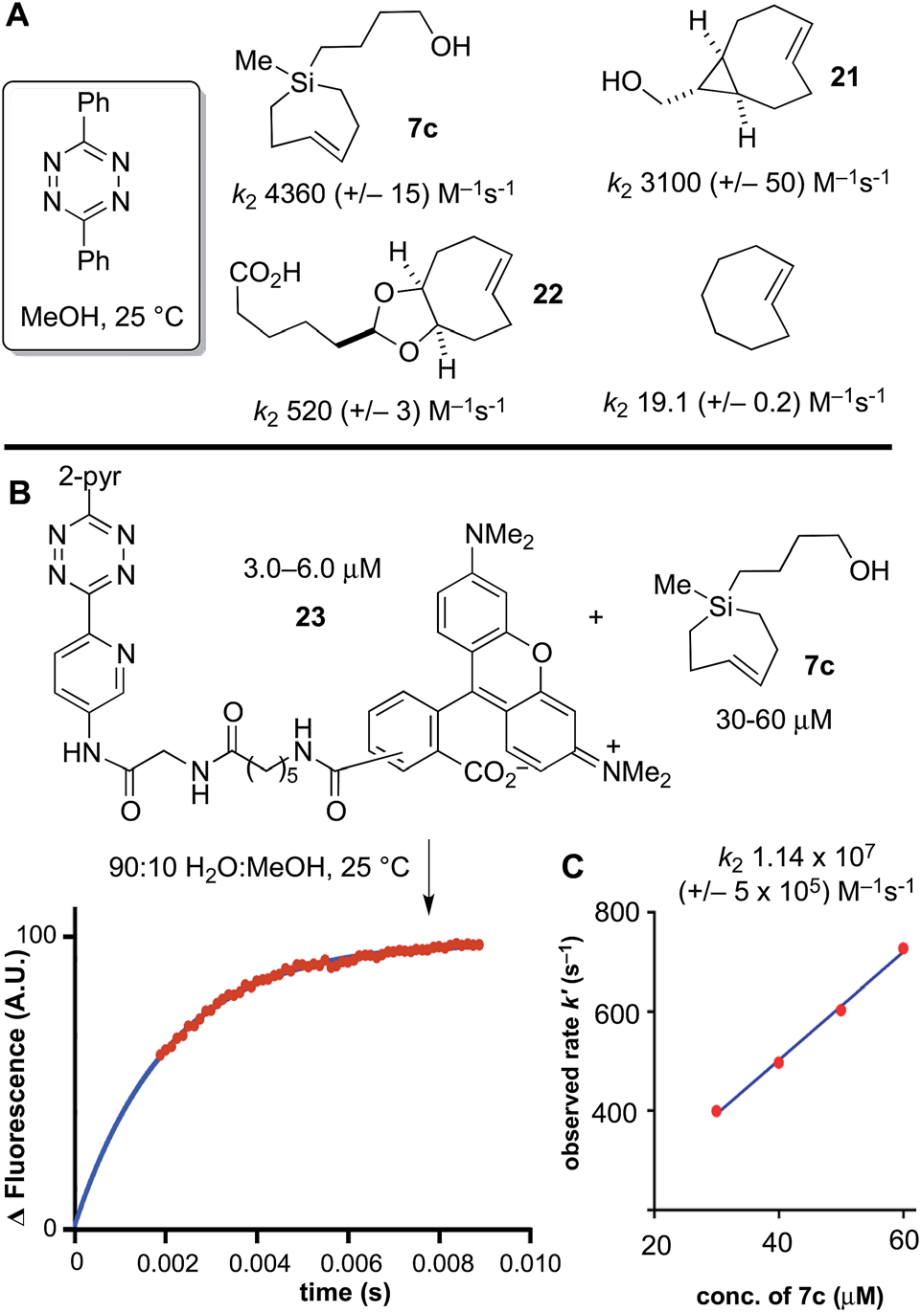
Second order rate constants (*k*_2_) were determined with a stopped-flow spectrophotometer under pseudo-first order conditions using excess *trans*-cycloalkene and limiting tetrazine. (A) The kinetics of the cycloaddition of 3,6-diphenyl-*s*-tetrazine in MeOH at 25 °C with Si-TCH **7c** was measured by stopped flow spectroscopy with UV-vis monitoring at 295 nm, and compared to previously reported rate constants for s-TCO **21**, d-TCO **22** and *trans*-cyclooctene. (B) Stopped flow kinetics with a TAMRA-3,6-dipyridyl-*s*-tetrazine conjugate were monitored in 9 : 1 H_2_O : MeOH with monitoring by fluorescence (Ex: 556 nm; Em: 576 nm). Data points are shown in red, and the fit is shown in blue. (C) Second order rate constants (*k*_2_) were determined by plotting *k*_obs_*vs.* the concentration of Si-TCH **7c**.

Previously, we had studied the reaction of diphenyl-*s*-tetrazine in MeOH at 25 °C with s-TCO,^21^,^54^ d-TCO,^21^,^22^ and *trans*-cyclooctene,^69^ with second order rate constants of 3100 (+/–50), 520 (+/–3) and 19.1 (+/–0.2) M^–1^ s^–1^, respectively. Si-TCH **7c** was liberated from AgNO_3_, and in agreement with computational prediction, was found to react with diphenyl-*s*-tetrazine with a rate constant of 4360 (+/–430) M^–1^ s^–1^—1.4 times faster than sTCO, 8.4 times faster than dTCO, and 228 times faster than *trans*-cyclooctene itself (Scheme 6A).

Under aqueous conditions, tetrazine ligations are accelerated due to the hydrophobic effect. We previously had studied the reactions of s-TCO, d-TCO and *trans*-cyclooctene with a water soluble dipyridyl-*s*-tetrazine derivative under stopped flow conditions in water with UV-vis monitoring.^21^ In the most rapid example, a rate constant of 3.3 × 10^6^ M^–1^ s^–1^ was measured for an s-TCO derivative.^21^ However, our attempts to conduct a similar measurement using Si-TCH **7c** were complicated because the reaction was complete before we could collect data even with stopped flow monitoring. To enable the measurement, we synthesized a fluorescent tetrazine-TAMRA conjugate **23**, and used fluorescence ‘turn-on’^70^ to monitor reaction progress. The fluorogenic reaction enabled reaction monitoring at much lower concentrations (down to 3 μM in tetrazine). As shown in Scheme 6B and C, the reaction of **23** with Si-TCH **7c** in 9 : 1 water : MeOH proceeds at 25 °C with a second order rate constant *k*_2_ 1.14 × 10^7^ (+/–5 × 10^5^) M^–1^ s^–1^. This is the fastest rate constant reported to date for a bioorthogonal reaction.

**Scheme 7:**
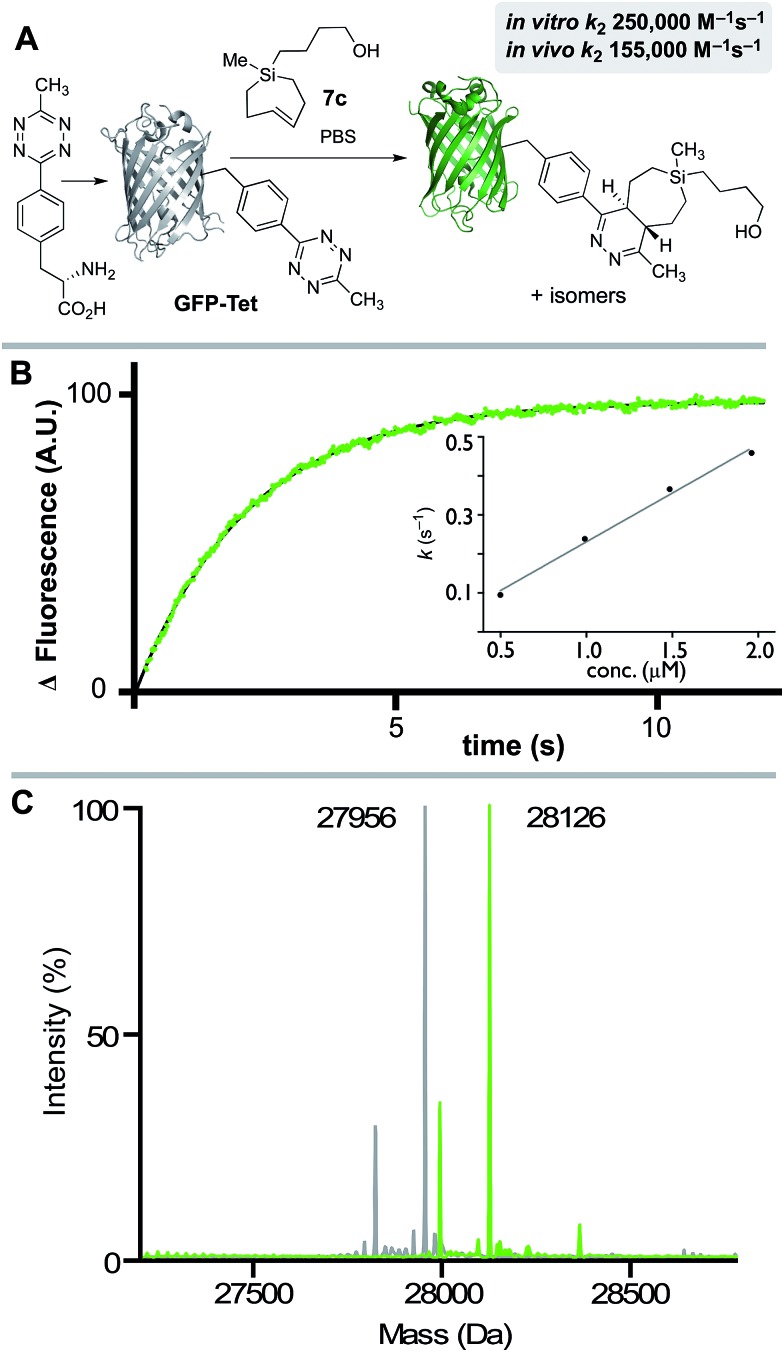
(A) **GFP-Tet** reacts with **7c** to give a Diels–Alder adduct. Because the tetrazine quenches GFP fluorescence, the reaction progress can be measured by monitoring fluorescence increase. (B) Stopped-flow kinetic data for the reaction of **GFP-Tet** (50 nM) with **7c** (1.96 μM) at 25 °C in PBS with fluorescence monitoring (Ex: 488 nm; Em: 506 nm). Data points are shown in green, and the fit is shown in black. A second order rate constant (*k*_2_) was determined by plotting *k*_obs_*vs.* the concentration of Si-TCH **7c**. (C) Quantitative determination of the cycloadduct was confirmed by ESI-MS. Deconvoluted mass spectra of **GFP-Tet** (grey) and the cycloadduct (green) are displayed and show the expected mass shift of 170 Da. Smaller peaks in each spectrum correspond to proteins that are truncated by a methionine.

We also studied the *in vitro* and *in vivo* cycloaddition of SiTCH and a green fluorescent protein with an unnatural tetrazine-containing amino acid (sfGFP-150Tet-v.2.0, referred to as **GFP-Tet**), encoded *via* the procedure of Mehl and coworkers.^71^ Thus, 4-(6-methyl-*s*-tetrazin-3-yl)phenylalanine was site-specifically introduced into a C-terminally hexahistidine-tagged GFP (sfGFP-150TAG-His6) *via* orthogonal translation using the evolved aminoacyl-tRNA synthetase MjRS/tRNACUA pair. Co-expression of these components in *E. coli* resulted in the amino acid-dependent synthesis of full-length recombinant **GFP-Tet** (Scheme 7A). The reaction of **GFP-Tet** with dienophiles is fluorogenic, and it is therefore possible to determine the reaction kinetics by monitoring the increase in GFP fluorescence.

Although **GFP-Tet** is highly reactive, with *in vitro* rates as fast as 87 000 M^–1^ s^–1^ toward sTCO, **GFP-Tet** is not as rapid as **23** due to the less reactive nature of the tetrazine. Kinetic measurements were carried out with silver free Si-TCH **7c**, which was obtained by treating Ag-complex **2c** with NH_4_OH and extracting with ether. The second order rate constant of the reaction between Si-TCH **7c** and **GFP-Tet** was determined to be 250 000 ± 15 000 M^–1^ s^–1^ in PBS at rt (Scheme 7B). The reaction was quantitative under these conditions as determined by ESI-MS (Scheme 7C), and is the fastest rate measured to date for **GFP-Tet**—2.9 times faster than previously measured rate constant for sTCO.^71^ Si-TCH **18** was also shown to display very rapid labeling of **GFP-Tet** when carried out in live bacteria. The kinetics of the *in vivo* tetrazine ligation were monitored in a suspension (PBS) of *E. coli* overexpressing **GFP-Tet** by measuring the increase in whole-cell fluorescence upon addition of **7c**. At room temperature, a second-order rate constant of 155 000 ± 20 000 M^–1^ s^–1^ was measured for the *in vivo* reaction, which is 62% as rapid as the *in vitro* ligation. The modest reduction in rate is in line with previous observations with TCO-based dienophiles.^21^,^71^,^72^ Quantitative determination of the bioorthogonal reaction was verified by cell washing, lysis, purification by IMAC, and analysis by ESI-MS. Thus, Si-TCH **7c** is capable of crossing the bacterial cell membrane and engaging in rapid, high yielding conjugation inside a living cell.

**Scheme 8:**
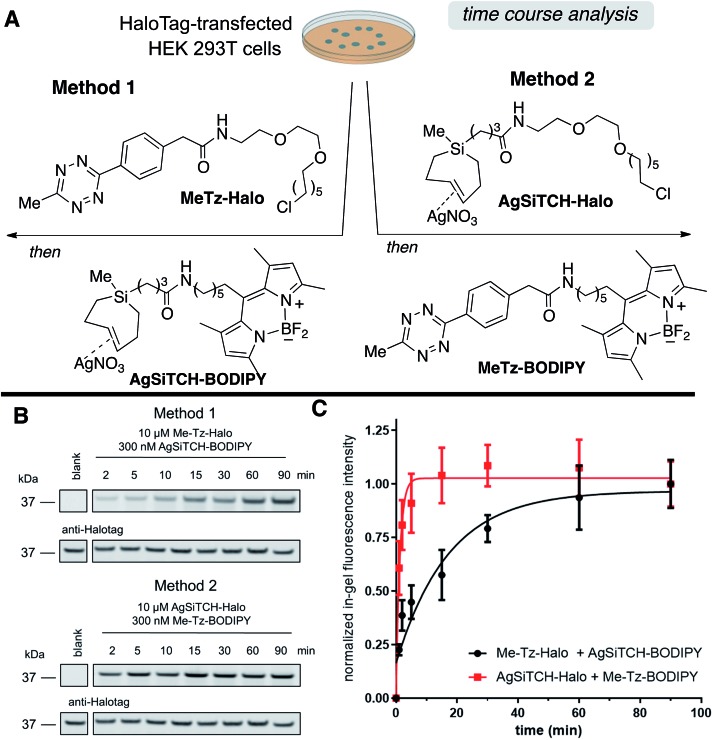
Reactions of AgSiTCH with 3-methyl-6-*s*-tetrazine derivatives in live HEK293T cells expressing HaloTag. (A) Cells were treated with 10 μM **MeTz-Halo** (method 1) or **AgSiTCH-Halo** (method 2) for 30 min, followed by two 30 min washings and treatment with 300 nM **AgSiTCH-BODIPY** (method 1) or 300 nM **MeTz-BODIPY** (method 2) for the indicated time. (B) Analyzed by in-gel fluorescence and western blot for each method: in-gel fluorescence is shown on the top panel, and western blot with an anti-HaloTag antibody is on the bottom panel. (C) Time course for the two reactions in (B). In-gel fluorescence intensity from *n* = 3 independent replicates was normalized by the corresponding western blot signal, and was fit to a one-phase exponential equation. Data were plotted as mean ± SEM.

The reactivity and specificity of the SiTCH reagent with tetrazines in live mammalian cells was evaluated using the HaloTag platform,^56^ which we have previously used to benchmark the efficiency of various bioorthogonal reactions in the cellular environment. We have previously shown that *trans*-cycloalkene–AgNO_3_-complexes liberate AgNO_3_ immediately in cell media due to the high NaCl content, and perform identically to their metal-free analogs in cell labeling experiments.^56^ Thus, we synthesized chloroalkane derivatives of SiTCH (**AgSiTCH-Halo**) and methyl-tetrazine (**MeTz-Halo**) and used these clickable HaloTag ligands to covalently label HaloTag protein expressed in HEK293T cells with the clickable tag. In a competitive pulse-chase experiment, it was shown that 10 μM of these HaloTag ligands completely blocked incorporation of BODIPY-Halo substrate (Fig. S25†), and therefore this concentration was used for subsequent experiments. Next we evaluated the tetrazine ligation of SiTCH in mammalian cells by reacting the **AgSiTCH-Halo** and **MeTz-Halo** protein conjugates with the corresponding **MeTz-BODIPY** or **AgSiTCH-BODIPY** fluorescent probes (300 nM) for different times (2–90 min) (Scheme 8 and Fig. S26†). The reaction was quenched at the various time points by chasing with excess non-fluorescent tetrazine-amine (method 1) or TCO-amine (method 2) (Fig. S27†), and in-gel fluorescence was used to quantify conversion *vs.* time.

We found that the reaction of **SiTCH-Halo** with **MeTz-BODIPY** was complete within 15 minutes (Scheme 8B, method 2). Interestingly, the reaction appeared to be slower with the reverse pairing where **SiTCH-BODIPY** was reacted with **MeTz-Halo** and did not reach saturation until 90 minutes (Scheme 8B, method 1). We believe this is due to the suboptimal permeability and nonspecific protein binding of **AgSiTCH-BODIPY** which results in lower free concentrations available for the reaction. Compared to **MeTz-BODIPY** or fluorescent 5-hydroxy-*trans*-cyclooctenes,^56^**AgSiTCH-BODIPY** also resulted in more nonspecific protein labeling (Fig. S26†). However, compared to in-gel fluorescence with a TAMRA-labeled bicyclononyne (BCN),^56^**AgSiTCH-BODIPY** appears to be more selective.

**Scheme 9:**
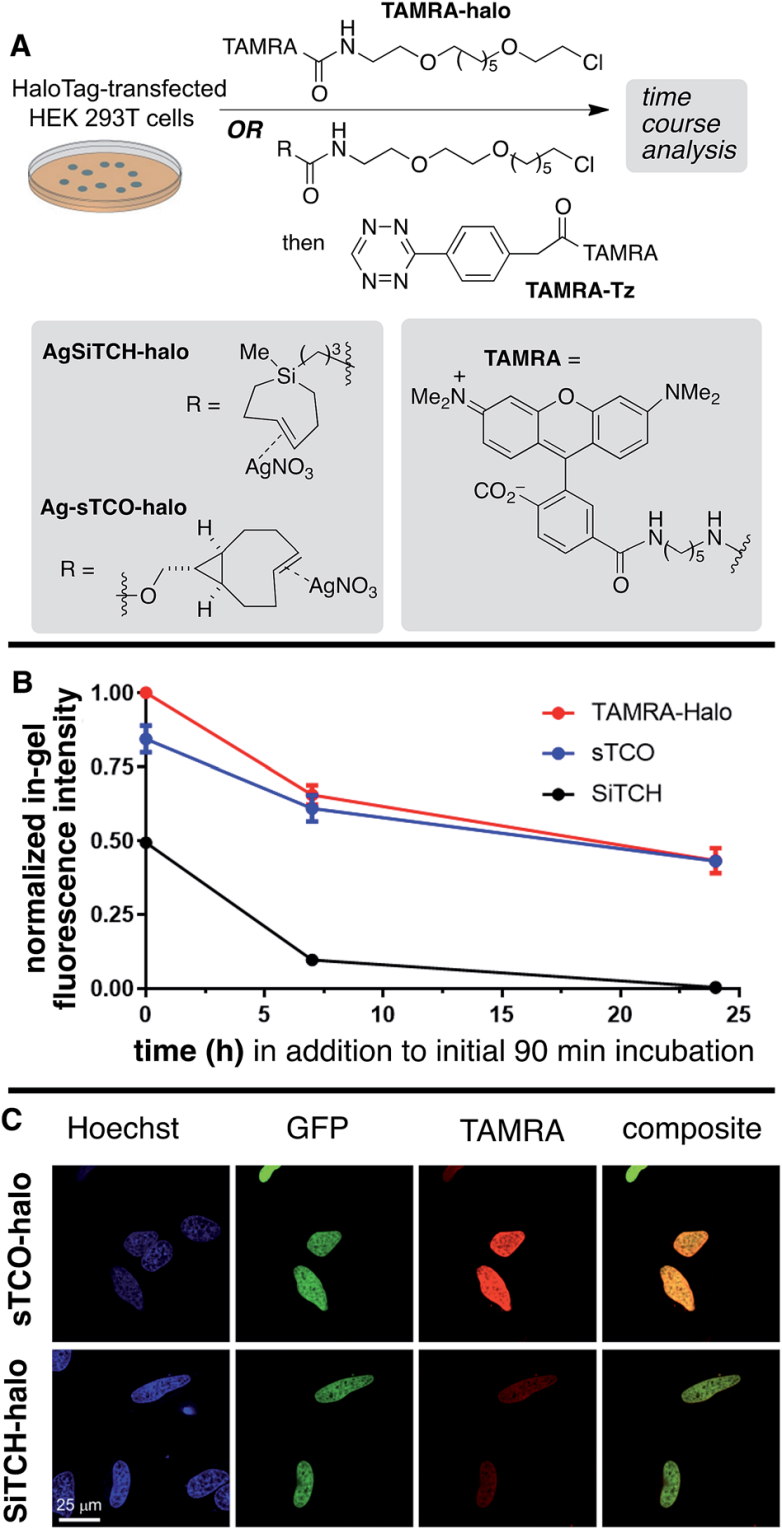
Live cell stability and imaging studies of SiTCH-Halo. (A) In the stability experiment, HEK293T cells expressing HaloTag were treated with 10 μM **Ag-sTCO-Halo** or **AgSiTCH-Halo** for 30 min, followed by two 30 min wash periods. Control cells were treated with 2 μM **TAMRA-Halo**. At 0, 7, and 24 h time points, 2 μM **TAMRA-Tz** was added and allowed to react for 1 h before analysis. (B) Cellular stability of **Ag-sTCO-Halo** and **AgSiTCH-Halo**. In-gel fluorescent intensities were normalized by the corresponding western blot signals, and were subsequently normalized by the value of **TAMRA-Halo** at time 0. Data were plotted as mean ± SEM. (C) Live cell images of HeLa cells expressing Halo-H2B-GFP. Cells were labeled with 10 μM **Ag-sTCO-Halo** or **AgSiTCH-Halo** for 30 min, followed by two 30 min wash periods, before the treatment with 1 μM **TAMRA-Tz** for 5 min. The reactions were quenched with 100 μM **TCO-amine** for 10 min, followed by two 30 min wash periods prior to addition of 8 μM Hoechst 33342 to visualize the nuclei and confocal imaging. Signals of Hoechst, GFP, and TAMRA were shown in blue, green, and red, respectively. Colocalization was demonstrated in composite images of GFP and TAMRA. Scale bar = 25 μm.

To investigate *in vivo* stability of incorporated SiTCH probes in the intracellular environment, we compared HaloTag protein tagging by **AgSiTCH-Halo** to the previously described **Ag-sTCO-Halo**.^56^ Subsequently the **TAMRA-Tz** fluorescent probe was attached *via* the tetrazine ligation (Scheme 9A). As a benchmark, we also compared directly incorporated **TAMRA-Halo** without a second bioorthogonal step. In these experiments, HaloTag-transfected cells were treated with 10 μM HaloTag ligands for 30 min, followed by two 30 min wash periods. After this initial 90 min exposure to cells, time course experiments were carried out to test the intracellular stability of the sTCO- and SiTCH-labeled HaloTag proteins. Thus, cells were allowed to incubate for up to 24 additional hours, and cells initially tagged by **AgSiTCH-Halo** or **Ag-sTCO-Halo** were then treated with **TAMRA-Tz** at the indicated time points. Loss of fluorescence intensity relative to the **TAMRA-Halo** benchmark indicated instability of the bioorthogonal protein tag.

Compared to the **TAMRA-Halo** ligand, the **Ag-sTCO-Halo** tagged protein displayed 84% fluorescence intensity when the **TAMRA-Tz** probe was attached immediately after the 30 min HaloTag-labeling and two 30 min wash periods (Scheme 9B, Fig. S28 and S29†). By contrast, **AgSiTCH-Halo** showed only 50% fluorescence intensity at this initial timepoint. After incubation for 7 h and 24 h, **Ag-sTCO-Halo** tagged protein showed 61% and 43% fluorescence intensity—very similar to the **TAMRA-Halo** benchmark. However, the **AgSiTCH-Halo** tagged protein displayed only 10% fluorescence after 7 h, and negligible fluorescence after 24 h. The reduced cellular labeling efficiency with **AgSiTCH-Halo** could also be observed in live cell images according to the published protocol (Scheme 9C and Fig. S30†).^56^ Specifically, HeLa cells overexpressing Halo-H2B-GFP were labeled with **AgSiTCH-Halo** or **Ag-sTCO-Halo**, and after two 30 min wash periods, treated with **TAMRA-Tz** for imaging. In both cases, specific fluorescent signals were observed in the nucleus with good colocalization with GFP signals. When **AgSiTCH-Halo** was used, however, the fluorescent intensity was only about half of that achieved with **Ag-sTCO-Halo**, confirming that the reduced stability of **SiTCH** in cells resulted in lower labeling efficiency. The lower labelling efficiency with **AgSiTCH-Halo** is likely due to *trans*-to-*cis* deactivation of the cycloalkane in the intracellular environment. Previously, we have studied the isomerization of *trans*-cyclooctenes under various conditions, and have shown that isomerization of *trans*-cyclooctenes likely occurs *via* radical mechanism that can be promoted by high thiol concentrations. Consistent with the faster *in cellulo* deactivation of **SiTCH-Halo** relative to **sTCO-Halo**, we observe that SiTCH **7b** (30 mM) completely isomerizes in 2 h at 22 °C in CD_3_OD with mercaptoethanol (30 mM), whereas as control sample without thiol was >98% stable under these conditions. At –17 °C, **7b** isomerizes more slowly in the presence of 30 mM mercaptoethanol, with 47% isomerization after 24 h. By comparison, the *trans*-cyclooctenes d-TCO, s-TCO, and oxo-TCO are much more stable toward thiol promoted isomerization. In CD_3_OD with mercaptoethanol (30 mM) at room temperature, s-TCO (30 mM) isomerized only after an 8 hour induction period, with complete conversion to the *cis*-isomer after 4 additional hours.^54^ In a similar experiment with d-TCO (30 mM), the induction period was 10 hours. After the induction period, there was 42% isomerization after 4 hours, and 92% isomerization after 14 hours.^56^ 5-OxoTCO (25 mM) in the presence of mercaptoethanol (25 mM) showed only 8% isomerization in CD_3_OD over a 22 hour period at room temperature.^72^

Together, the labeling experiments in bacteria and HEK293T cells show that SiTCH derivatives can serve as useful probe molecules in the cellular environment, as the unprecedented speed of the bioorthogonal reactions of SiTCH are much more rapid than competing deactivation pathways. However, the utility of SiTCH derivatives as protein tagging molecules, where extended incubation in the cellular environment takes place prior to bioorthogonal reactivity, appears much more limited in utility plausibly due to alkene isomerization in the cellular environment.

## Conclusions

In conclusion, AgNO_3_ complexes of *trans*-cycloheptene and *trans*-1-sila-4-cycloheptene derivatives have been prepared *via* a flow photochemical synthesis, using a new low temperature flow photoreactor to enable the synthesis of carbocyclic TCH derivatives. TCH·AgNO_3_ complexes can be handled for brief periods at rt and stored for weeks in the freezer (–18 °C). Si-TCH·AgNO_3_ complexes are especially stable, and can be stored on the bench stable for >1 week at rt, and for months in the freezer. X-ray crystallography was used to characterize a Si-TCH·AgNO_3_ complex for the first time. With decomplexation of AgNO_3_*in situ*, metal-free TCO and Si-TCH derivatives can engage in a range of cycloaddition reactions as well as dihydroxylation reactions. Computation predicted that Si-TCH would display faster bioorthogonal reactions toward tetrazines than even the most reactive *trans*-cyclooctenes. Metal-free Si-TCH derivatives were shown to display good stability in solution, and to engage in the fastest bioorthogonal reaction reported to date (*k*_2_ 1.14 × 10^7^ M^–1^ s^–1^ in 9 : 1 H_2_O : MeOH). Utility in bioorthogonal protein labeling in live cells is described, including labeling of GFP with an unnatrual tetrazine-containing amino acid. The reactivity and specificity of the Si-TCH reagents with tetrazines in live mammalian cells was also evaluated using the HaloTag platform. The cell labeling experiments show that Si-TCH derivatives are suitable as highly reactive probe molecules in the cellular environment.

## Conflicts of interest

There are no conflicts to declare.

## Supplementary Material

Supplementary information

Supplementary information

Crystal structure data
