# Supplementary material for: Photochemical syntheses, transformations, and bioorthogonal chemistry of trans-cycloheptene and sila trans-cycloheptene Ag(i) complexes
Source: Chem Sci. 2018 Jan 8;9(7):1953–63. doi: 10.1039/c7sc04773h (PMC5892336; doi:10.1039/c7sc04773h)

Electronic Supplementary Material (ESI) for Chemical Science.
This journal is © The Royal Society of Chemistry 2018

^{1}H NMR (600 MHz, $CDCl_3$)

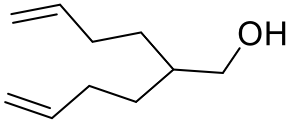

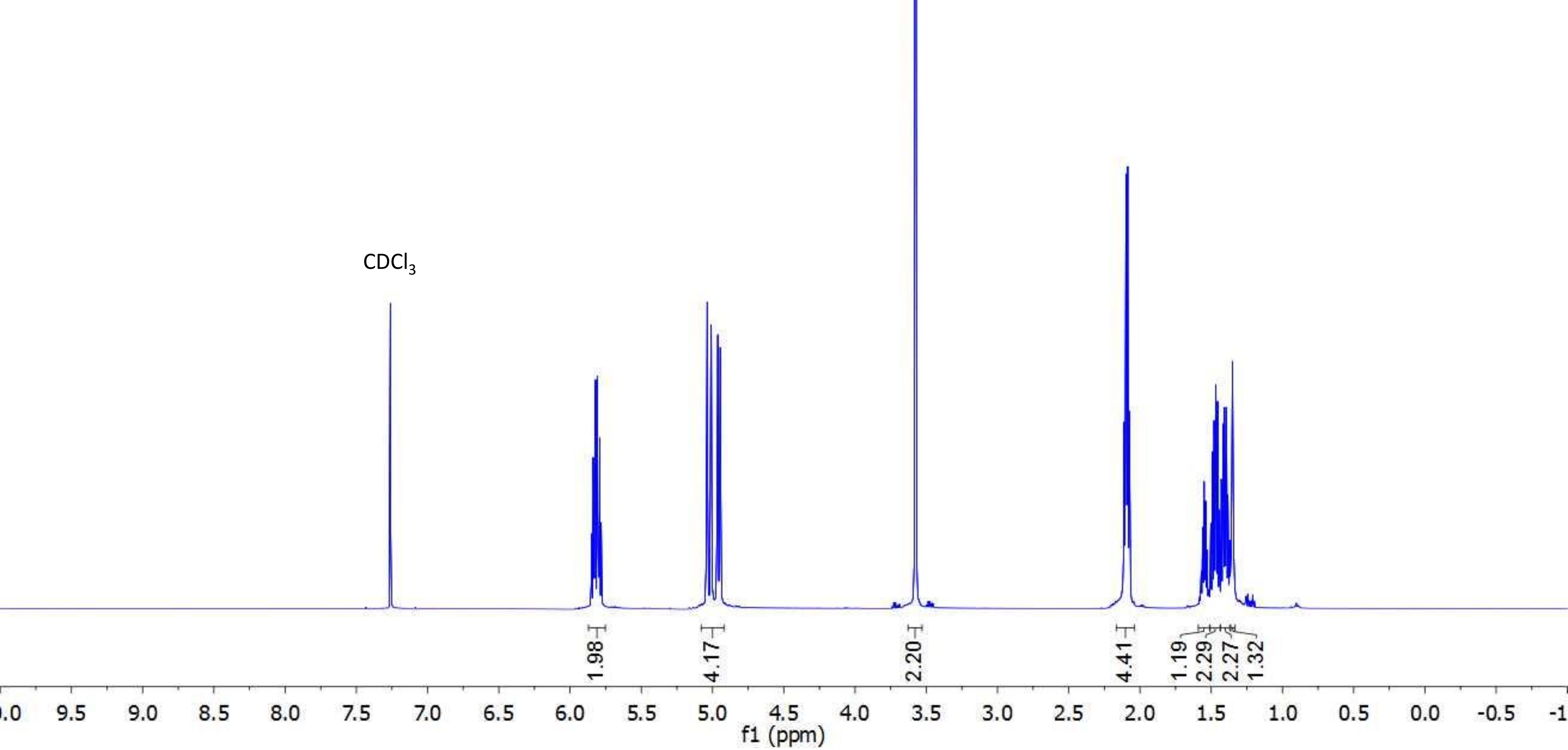

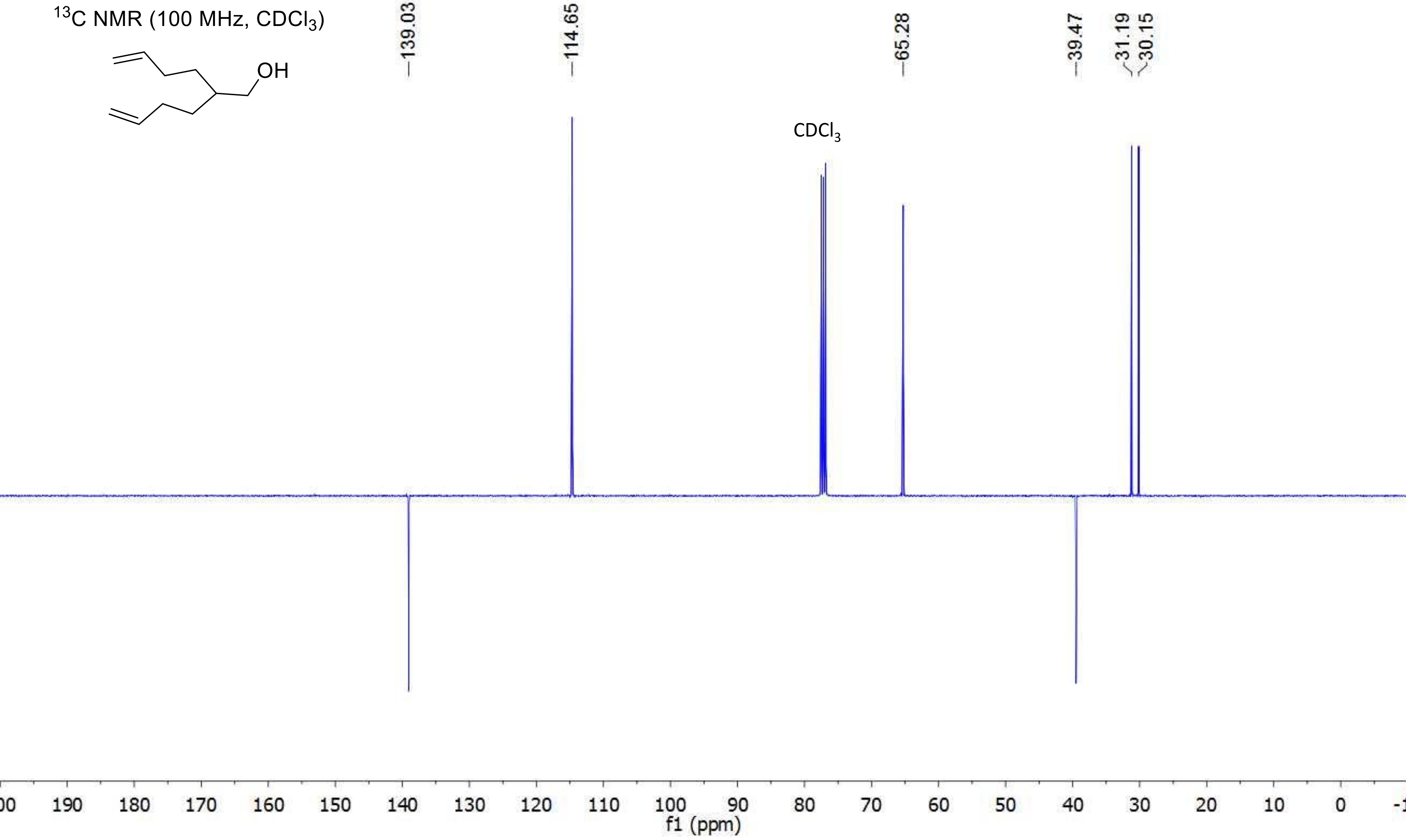
13C NMR (100 MHz, CDCl3)
OH
139.03
114.65
65.28
39.47
31.19
30.15
CDCl3
200
190
180
170
160
150
140
130
120
110
100
90
80
70
60
50
40
30
20
10
0
f1 (ppm)

^{1}H NMR (400 MHz, $CDCl_3$)

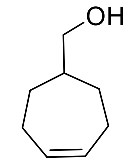

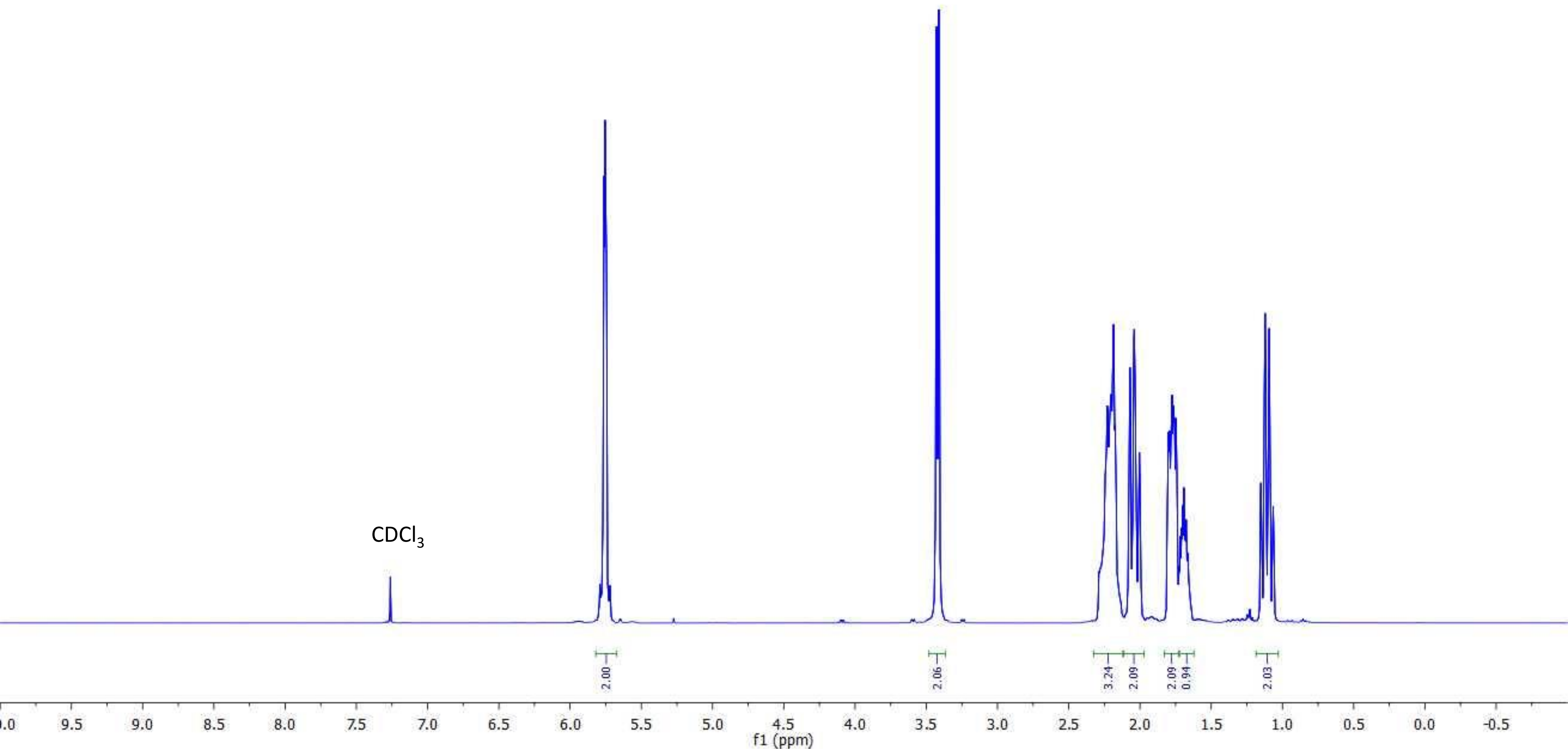

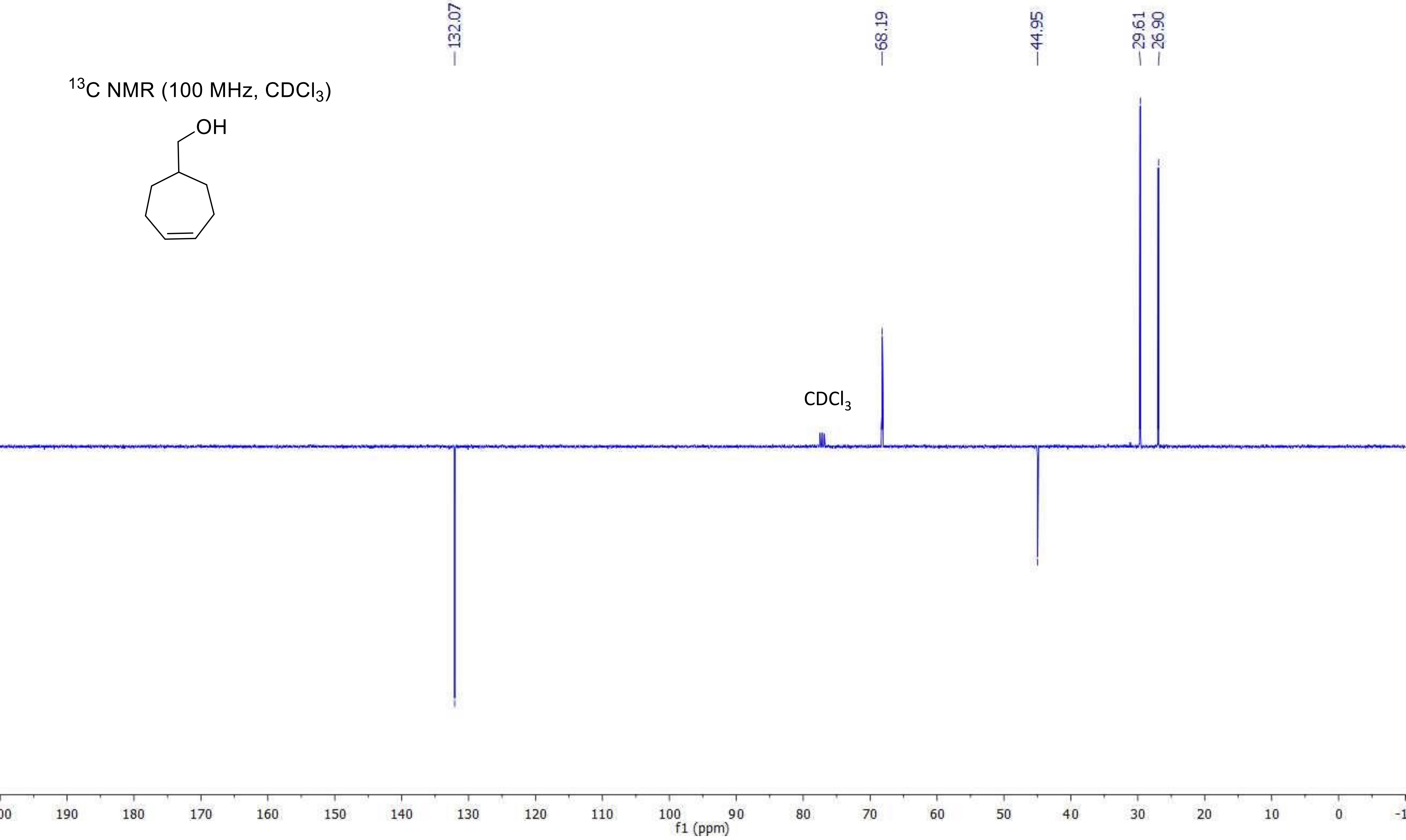

132.07
68.19
44.95
29.61
26.90
^{13}C NMR (100 MHz, $CDCl_3$)
OH
$CDCl_3$
200
190
180
170
160
150
140
130
120
110
100
90
80
70
60
50
40
30
20
10
0
-10
f1 (ppm)

^1H NMR (600 MHz, $CDCl_3$)

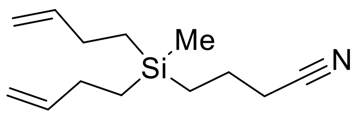

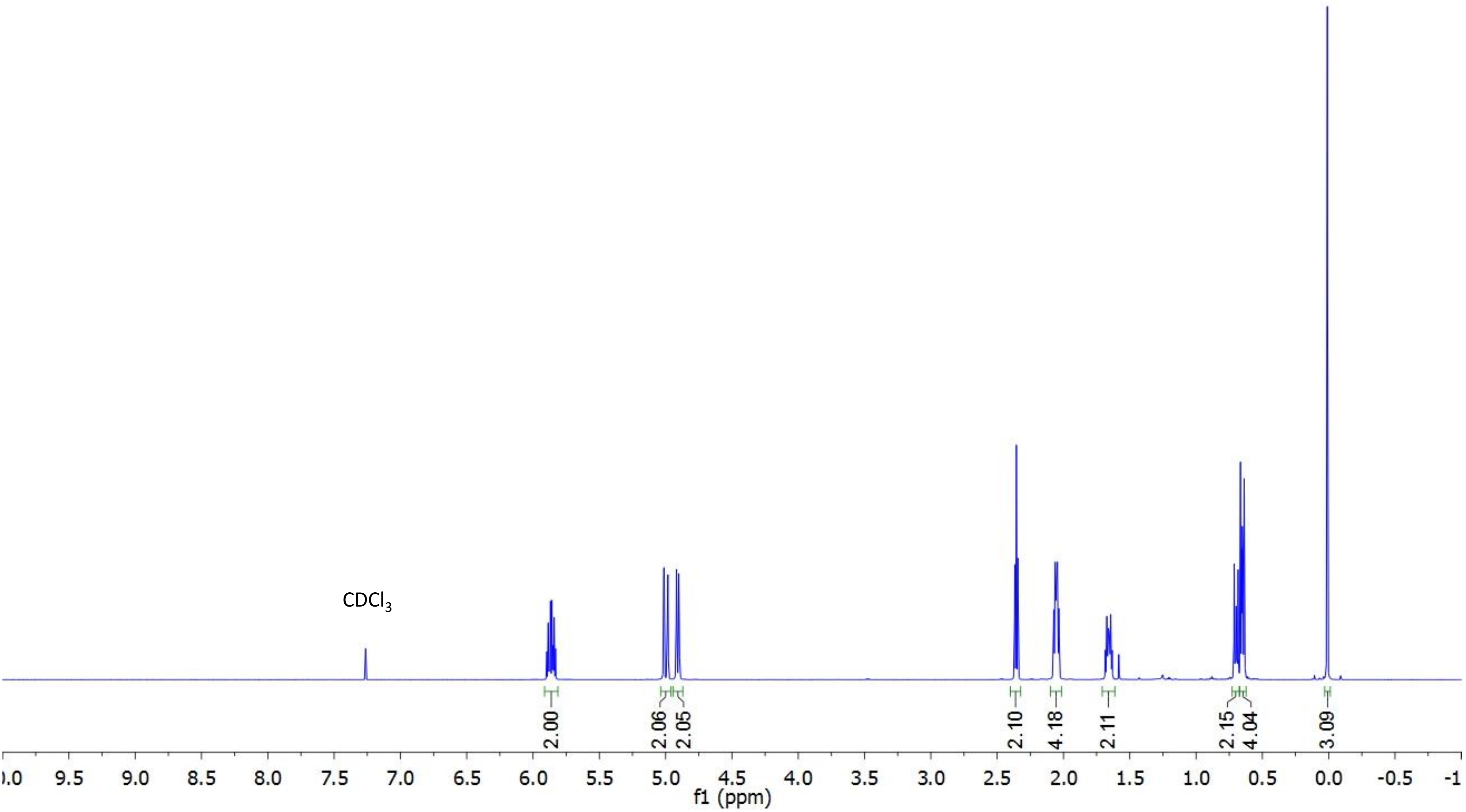

^{13}C NMR (150 MHz, $CDCl_3$)

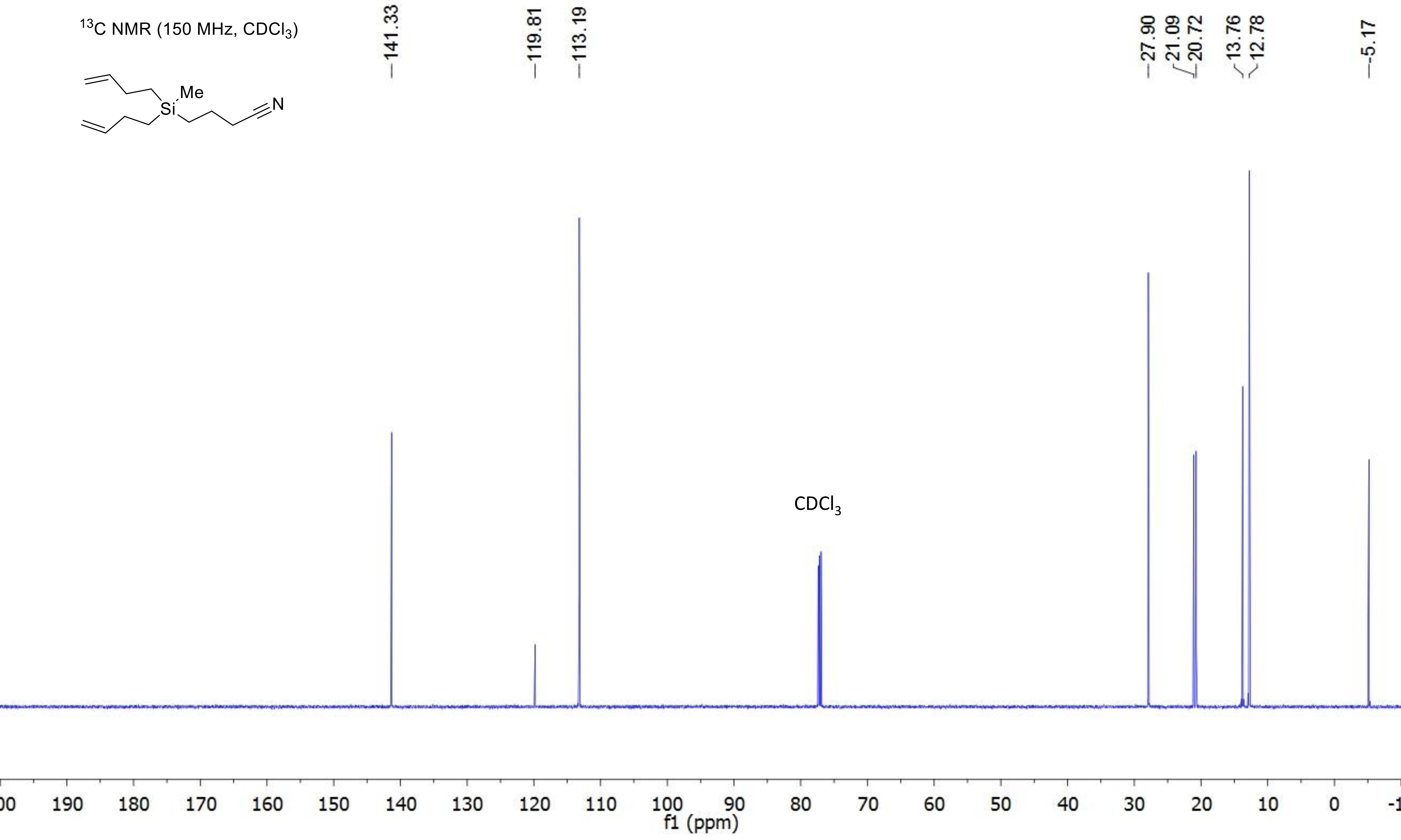

^1H NMR (400 MHz, $CDCl_3$)

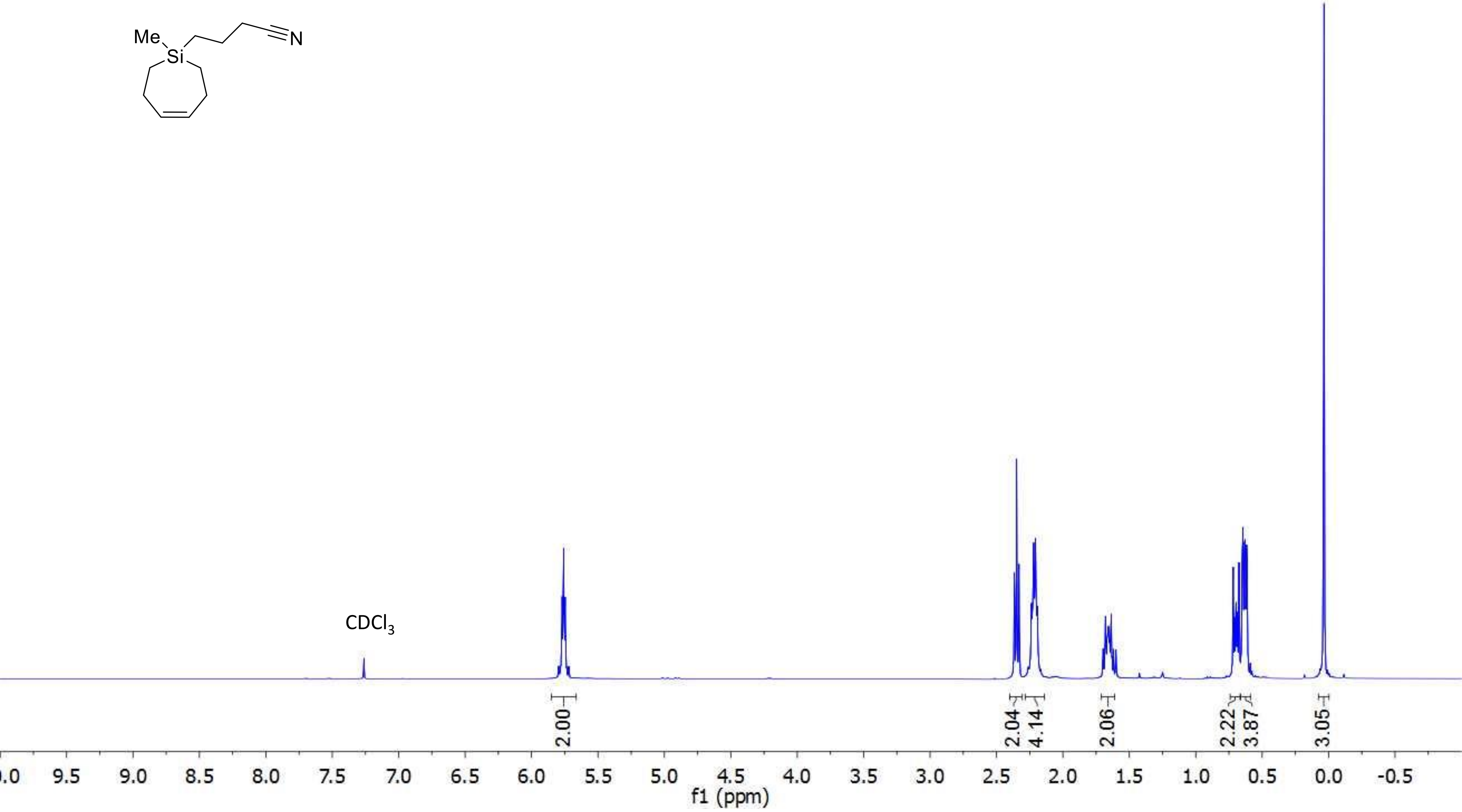

^{13}C NMR (100 MHz, $CDCl_3$)

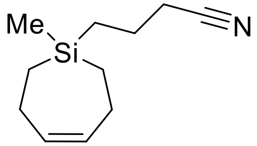

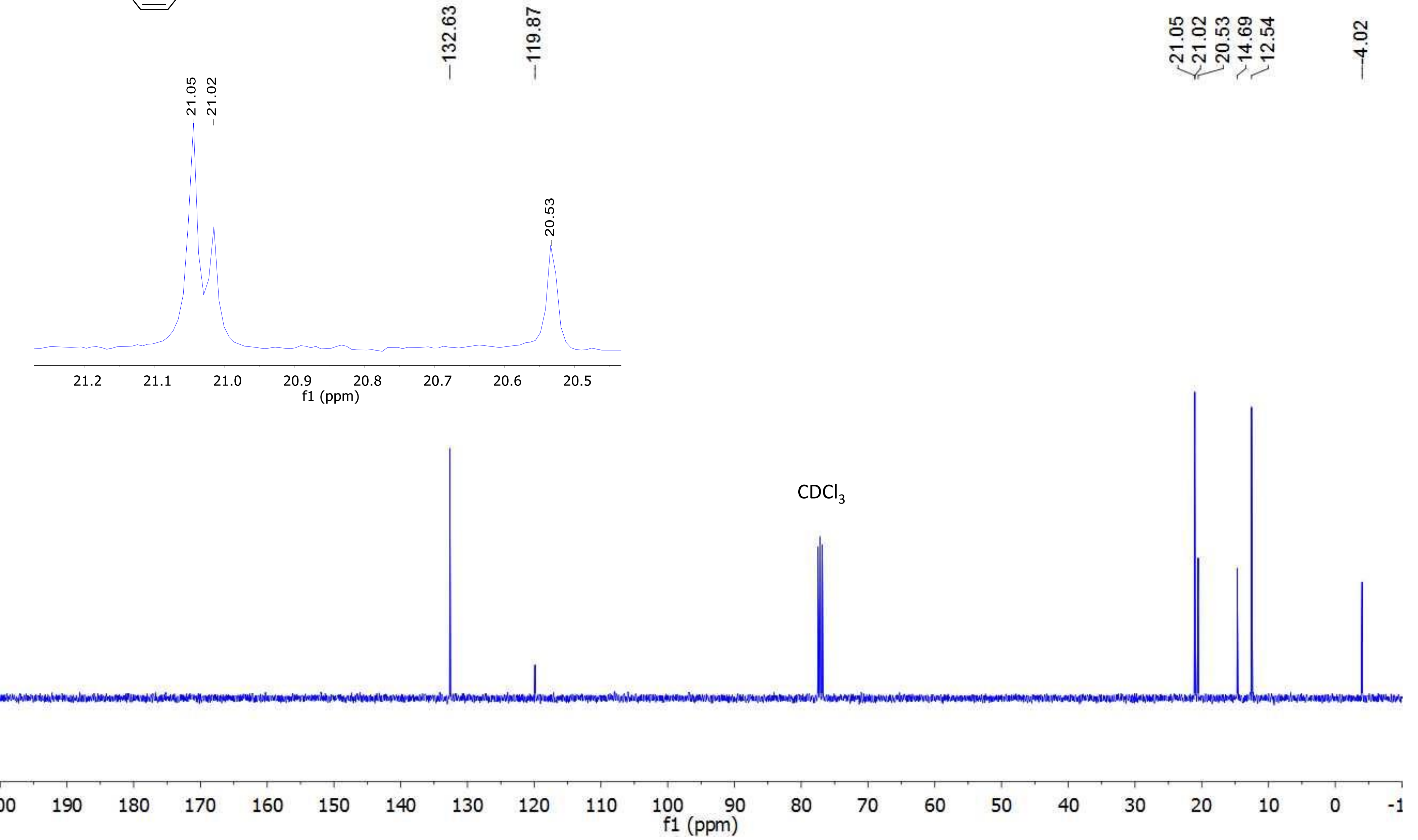

^1H NMR (400 MHz, $CDCl_3$)

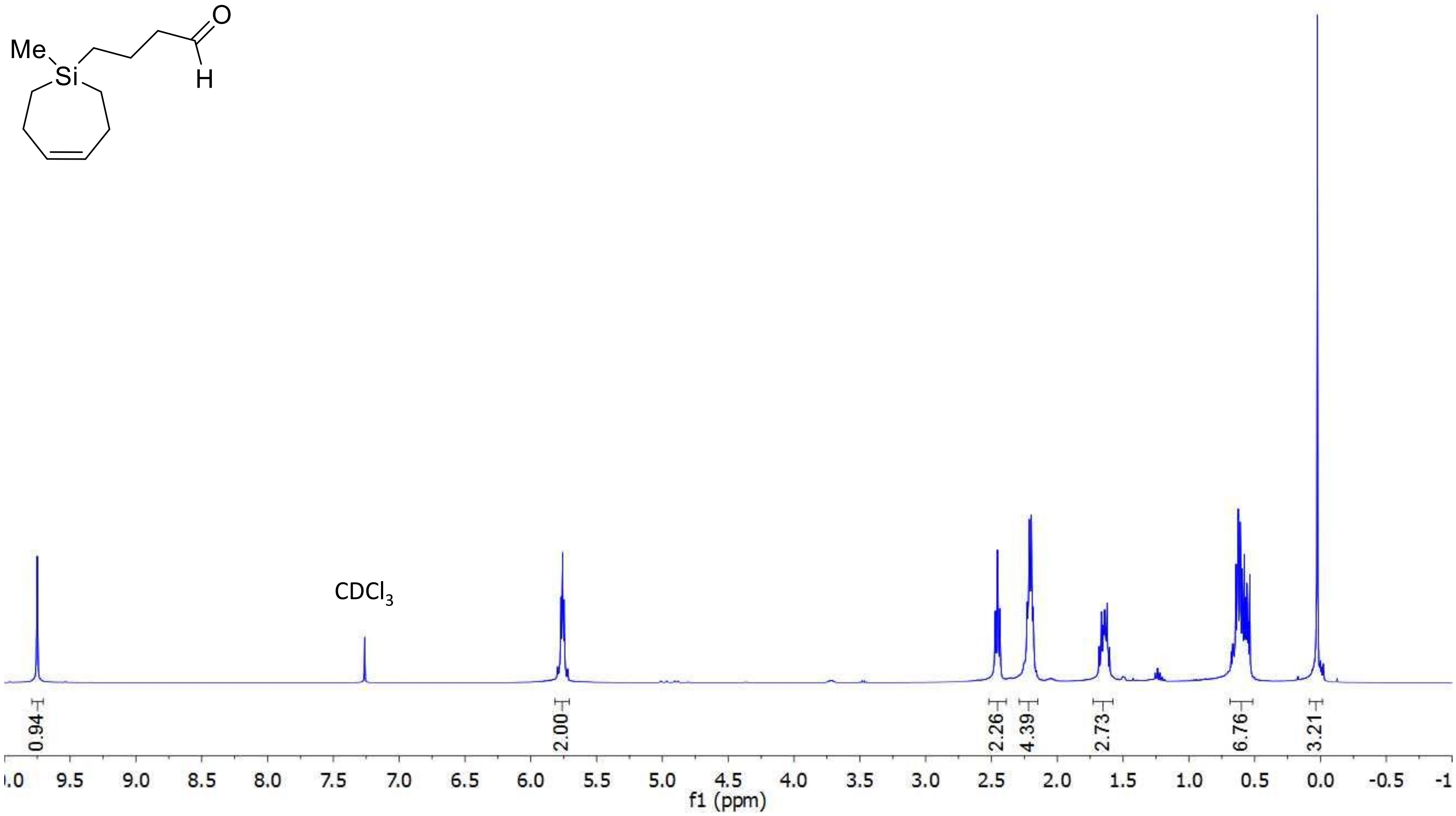

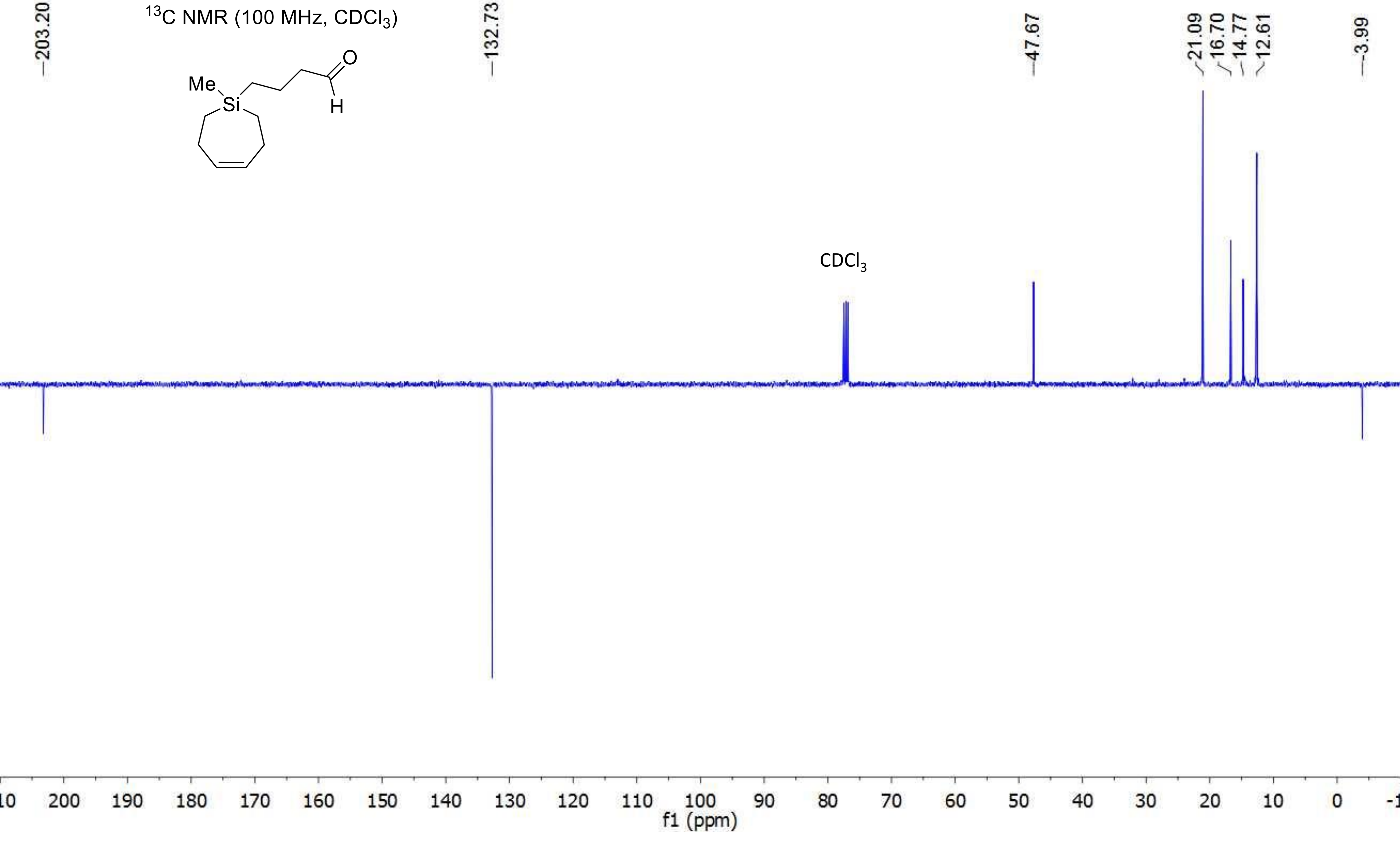

13C NMR (100 MHz, CDCl3)
Me
Si
O
H
CDCl3
203.20
132.73
47.67
21.09
16.70
14.77
12.61
3.99
f1 (ppm)

^{1}H NMR (400 MHz, $CDCl_3$)

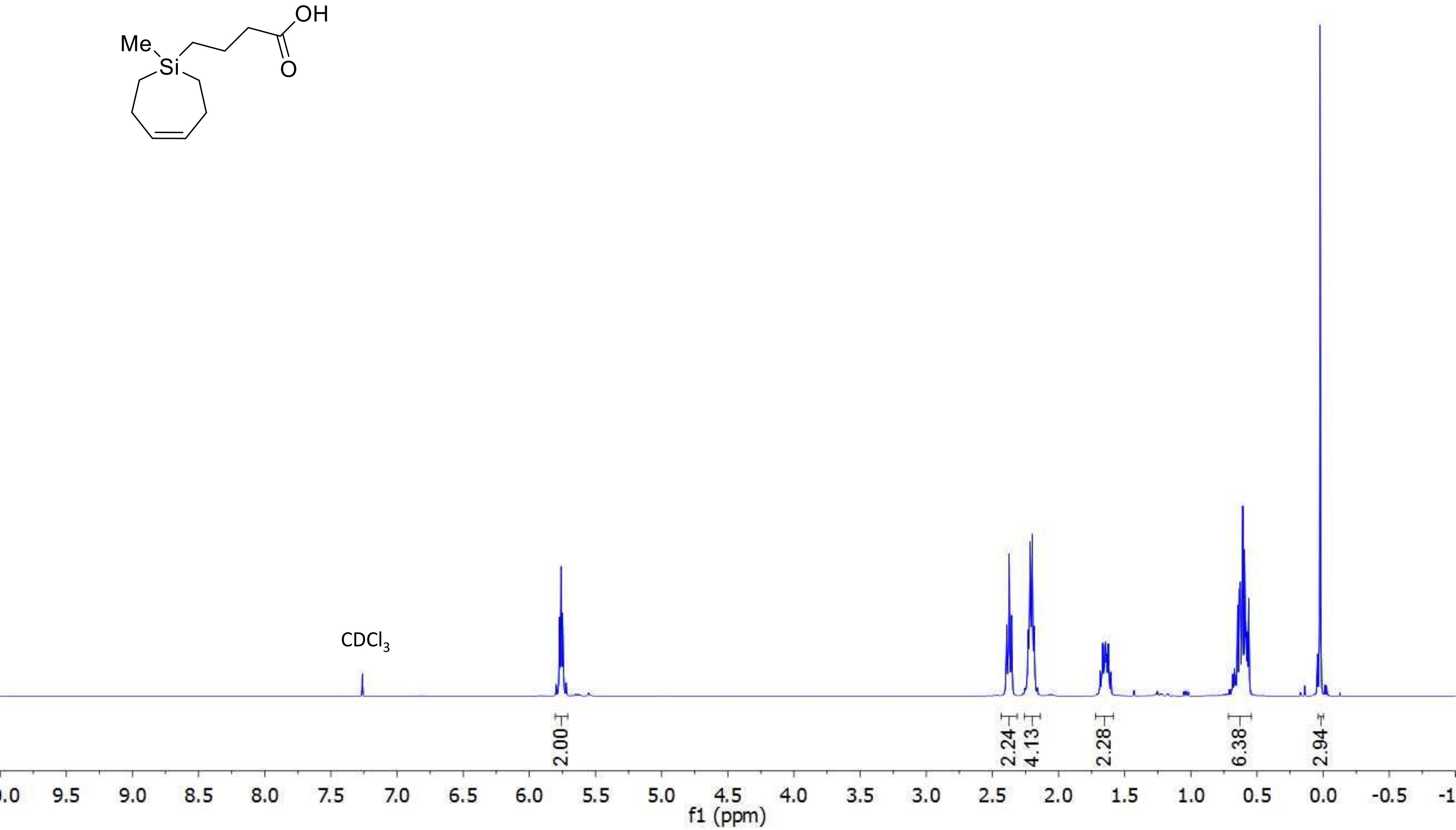

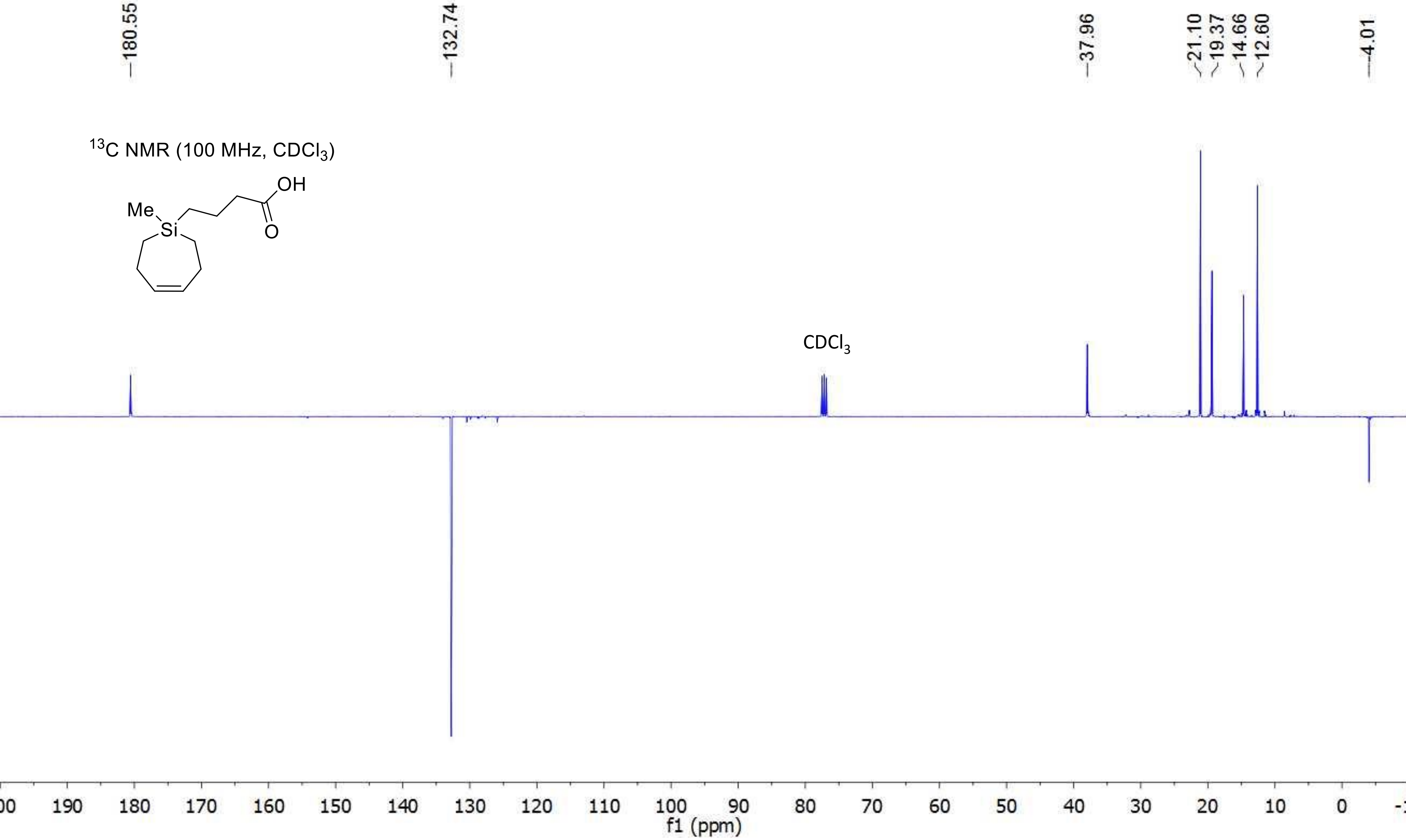

180.55
132.74
37.96
21.10
19.37
14.66
12.60
-4.01
13C NMR (100 MHz, CDCl3)
OH
Me
Si
O
CDCl3
190
180
170
160
150
140
130
120
110
100
90
80
70
60
50
40
30
20
10
0
f1 (ppm)

^1H NMR (400 MHz, $CDCl_3$)

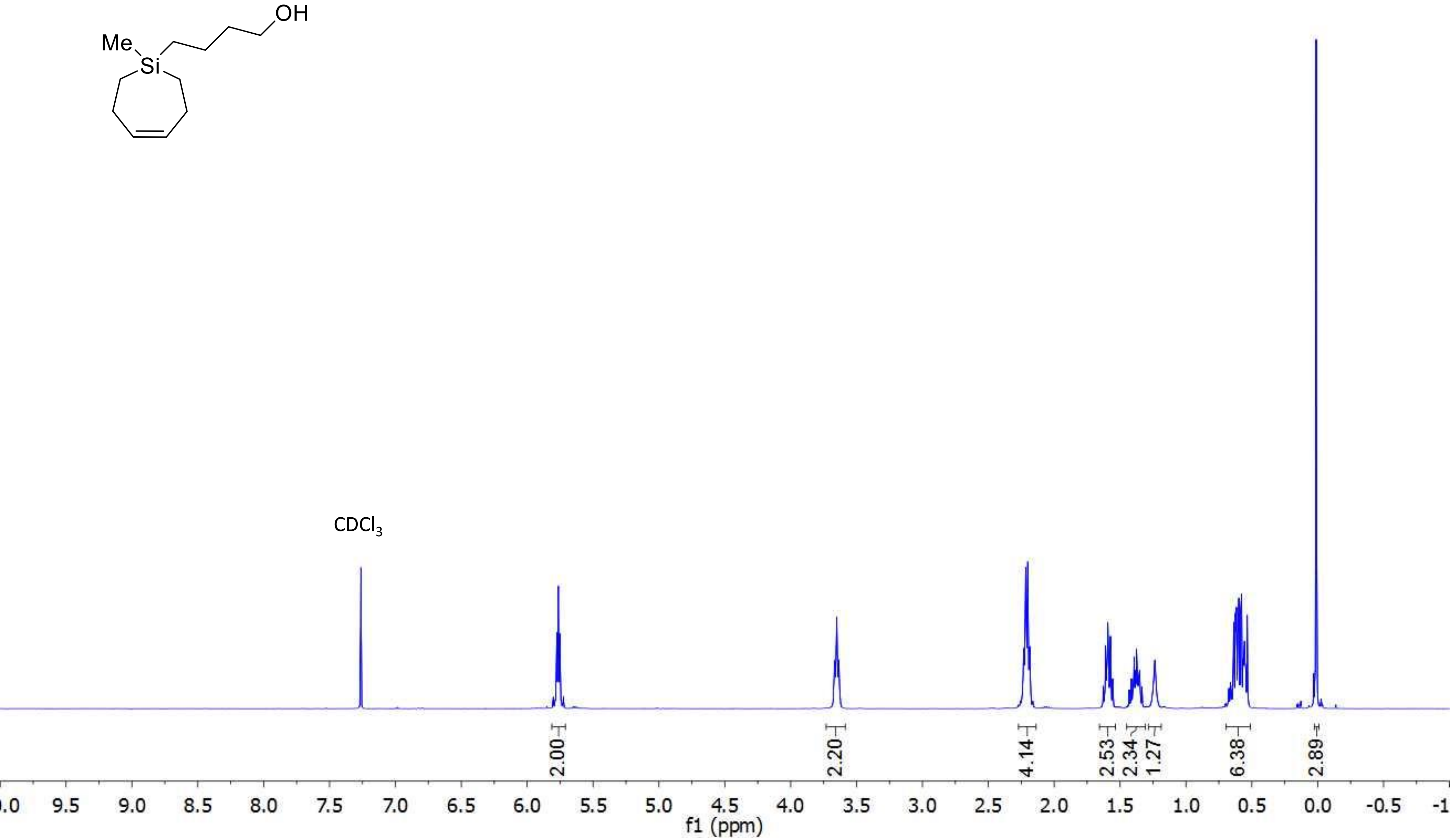

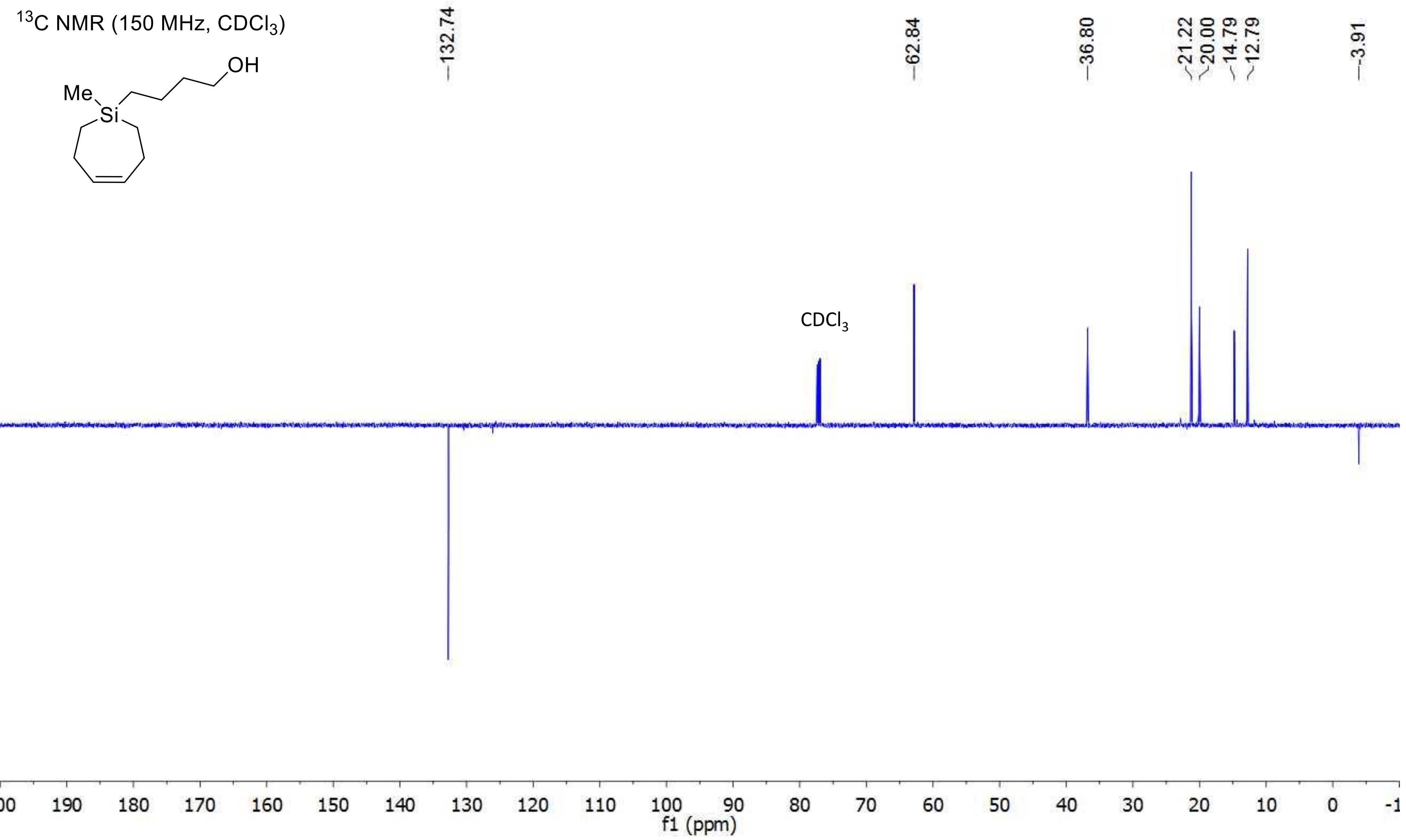

13C NMR (150 MHz, CDCl3)
Me
Si
OH
132.74
62.84
36.80
21.22
20.00
14.79
12.79
-3.91
CDCl3
00
190
180
170
160
150
140
130
120
110
100
90
80
70
60
50
40
30
20
10
0
-1
f1 (ppm)

^{1}H NMR (600 MHz, $CDCl_3$)

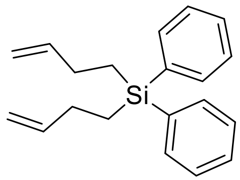

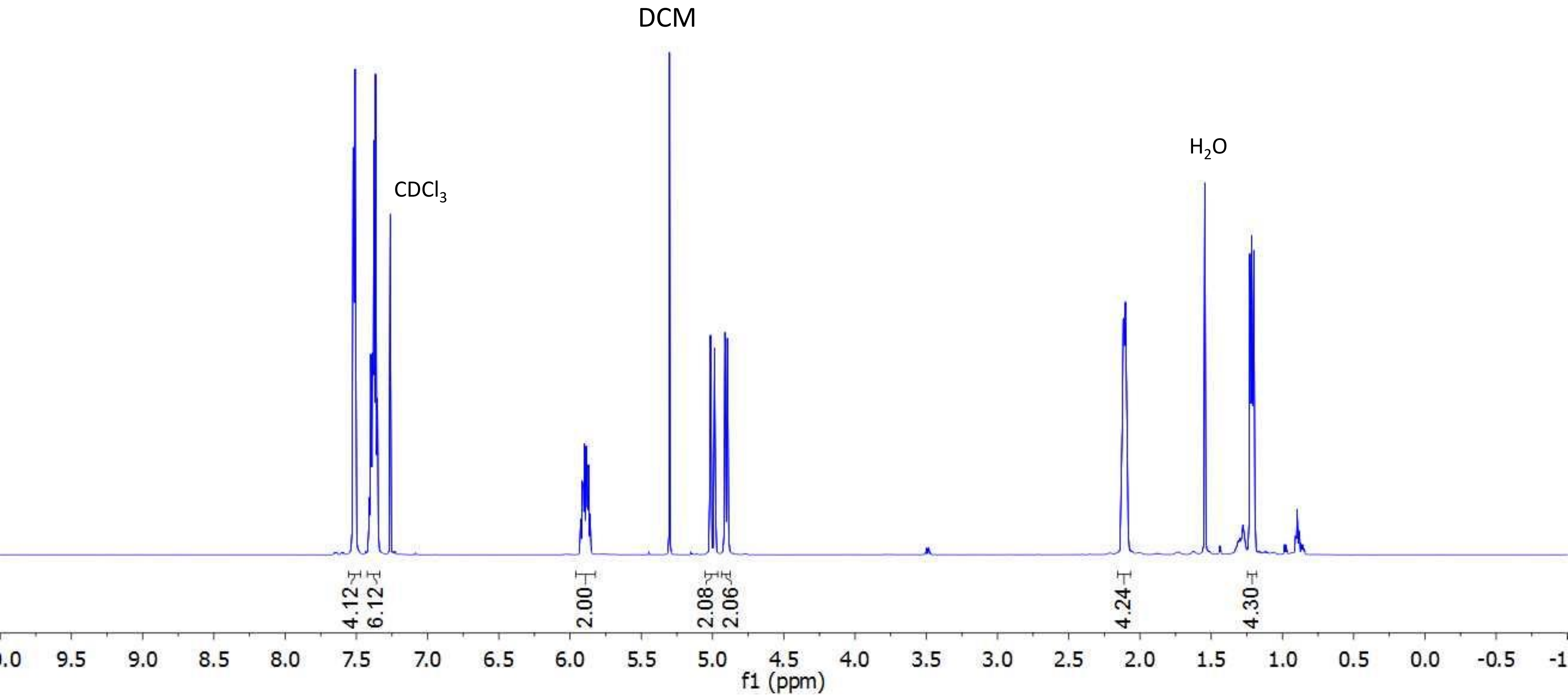

^{13}C NMR (100 MHz, $CDCl_3$)

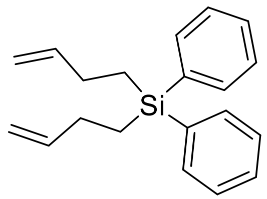

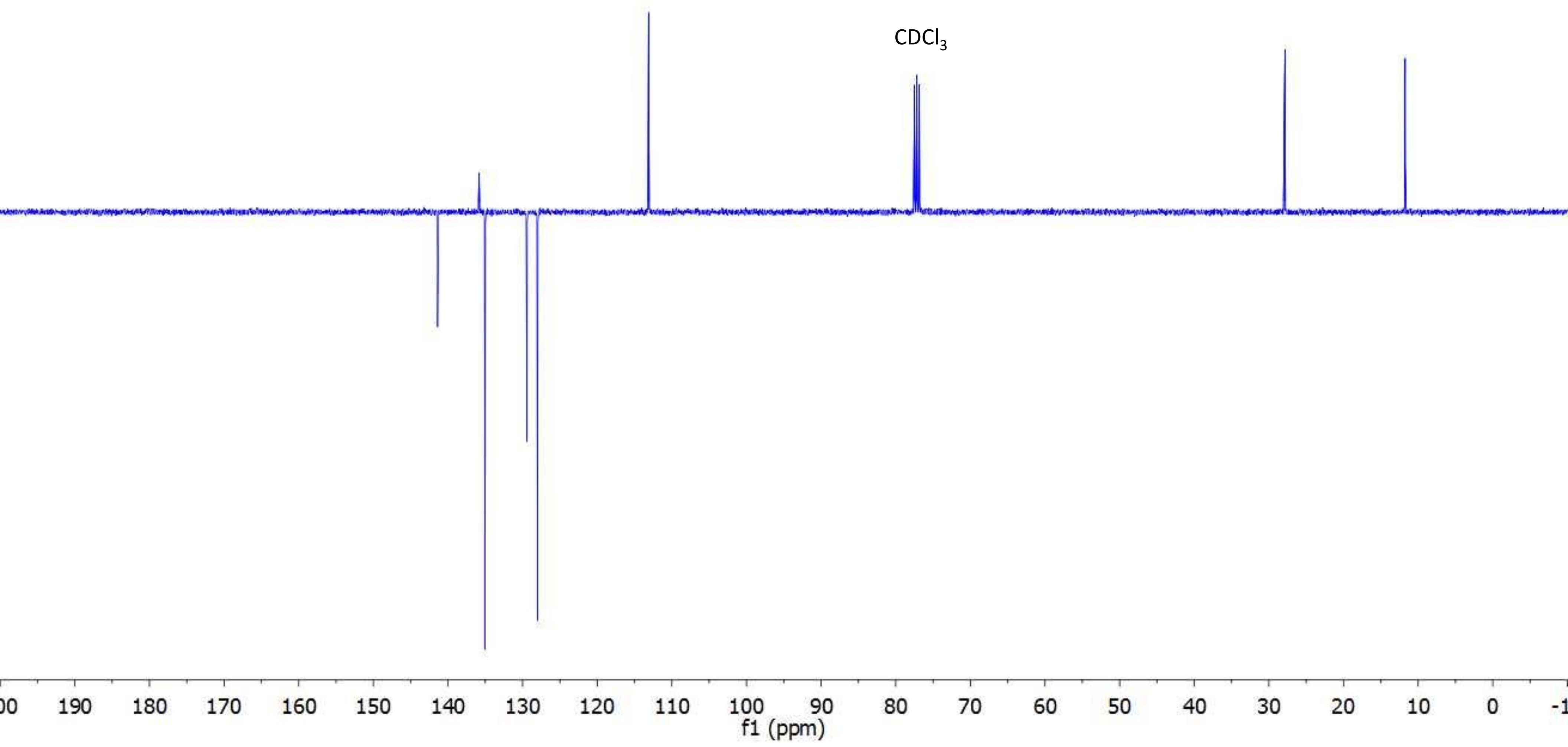

^{1}H NMR (600 MHz, $CDCl_3$)

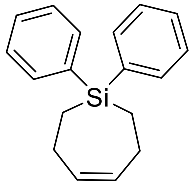

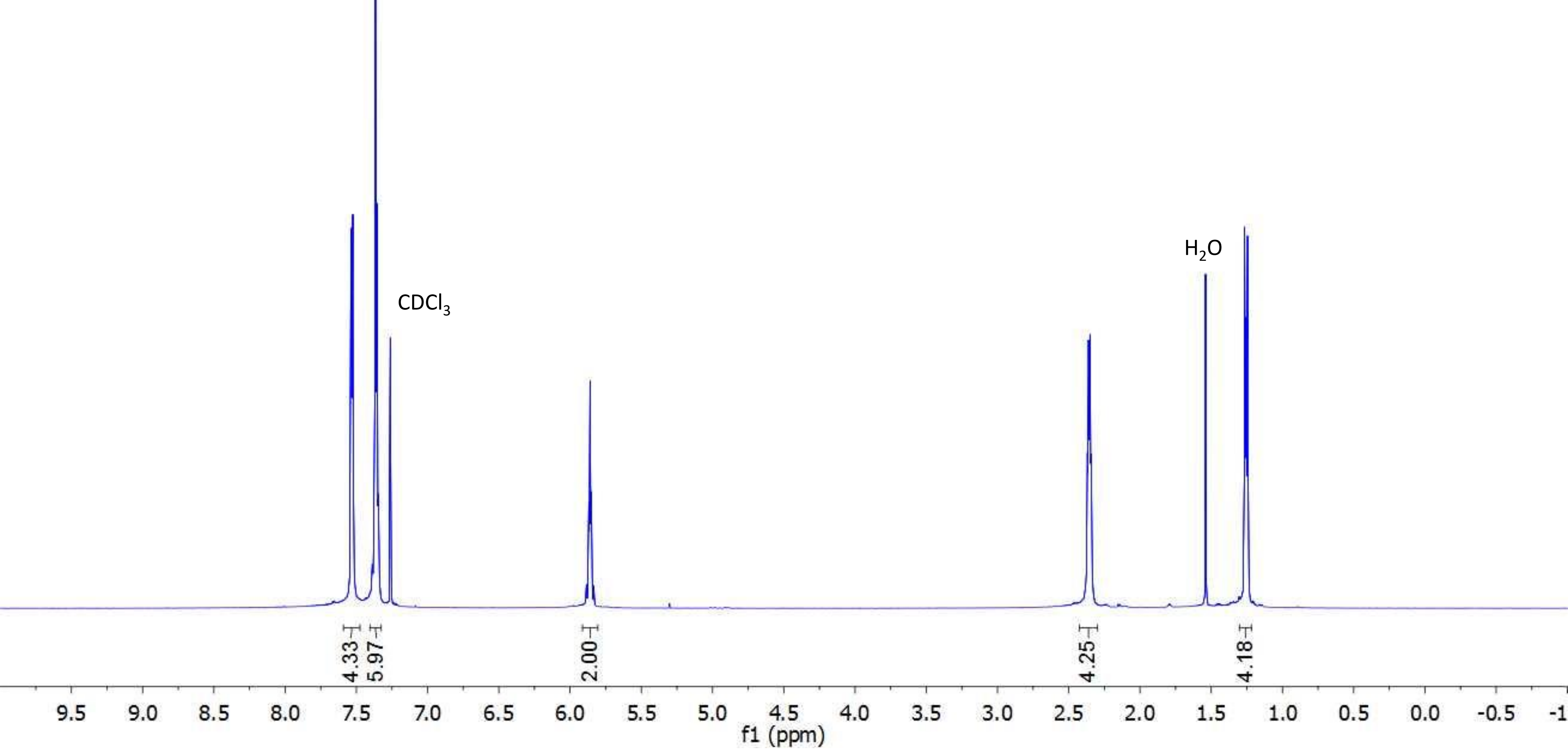

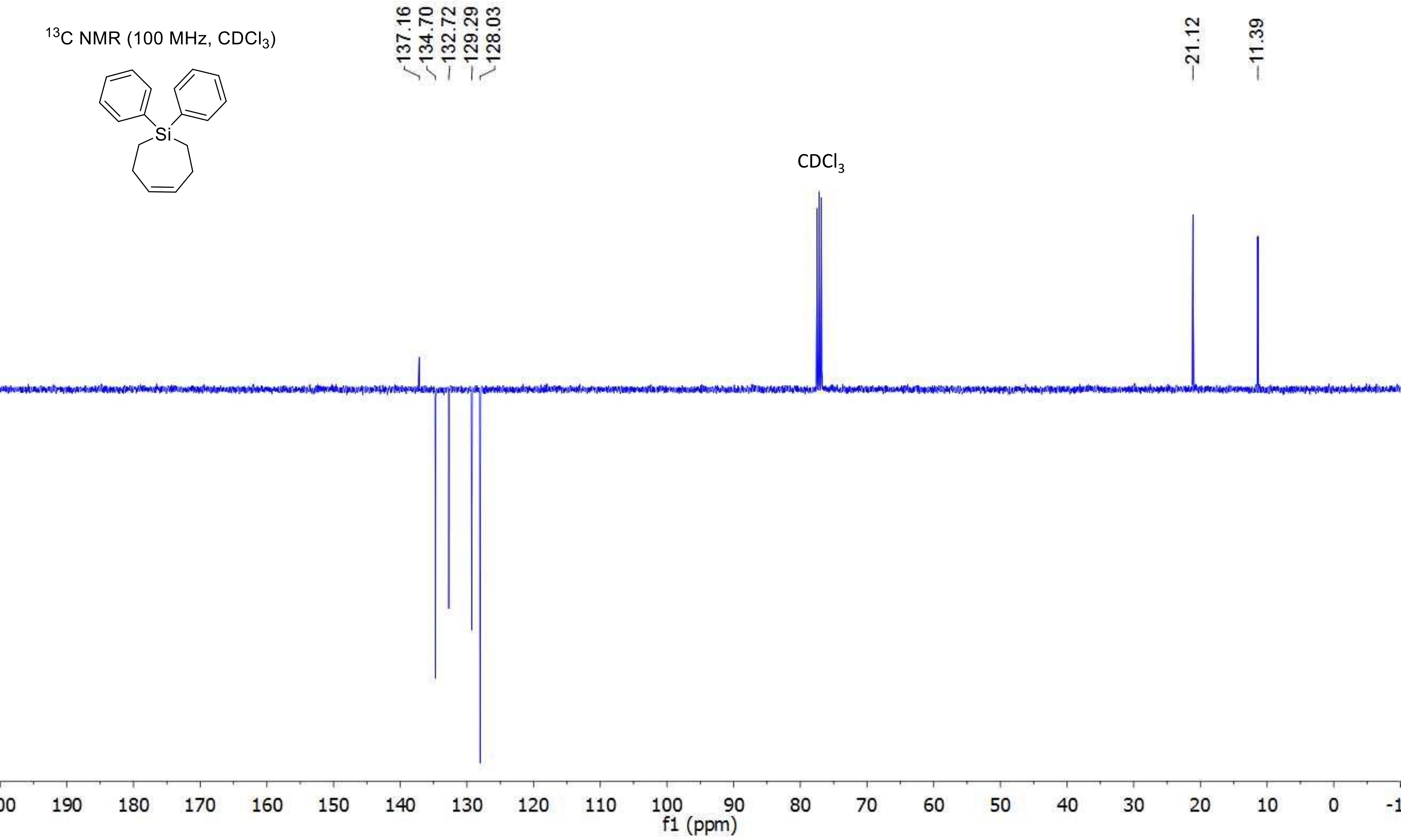

13C NMR (100 MHz, CDCl3)
Si
137.16
134.70
132.72
129.29
128.03
CDCl3
21.12
11.39
190
180
170
160
150
140
130
120
110
100
90
80
70
60
50
40
30
20
10
0
f1 (ppm)

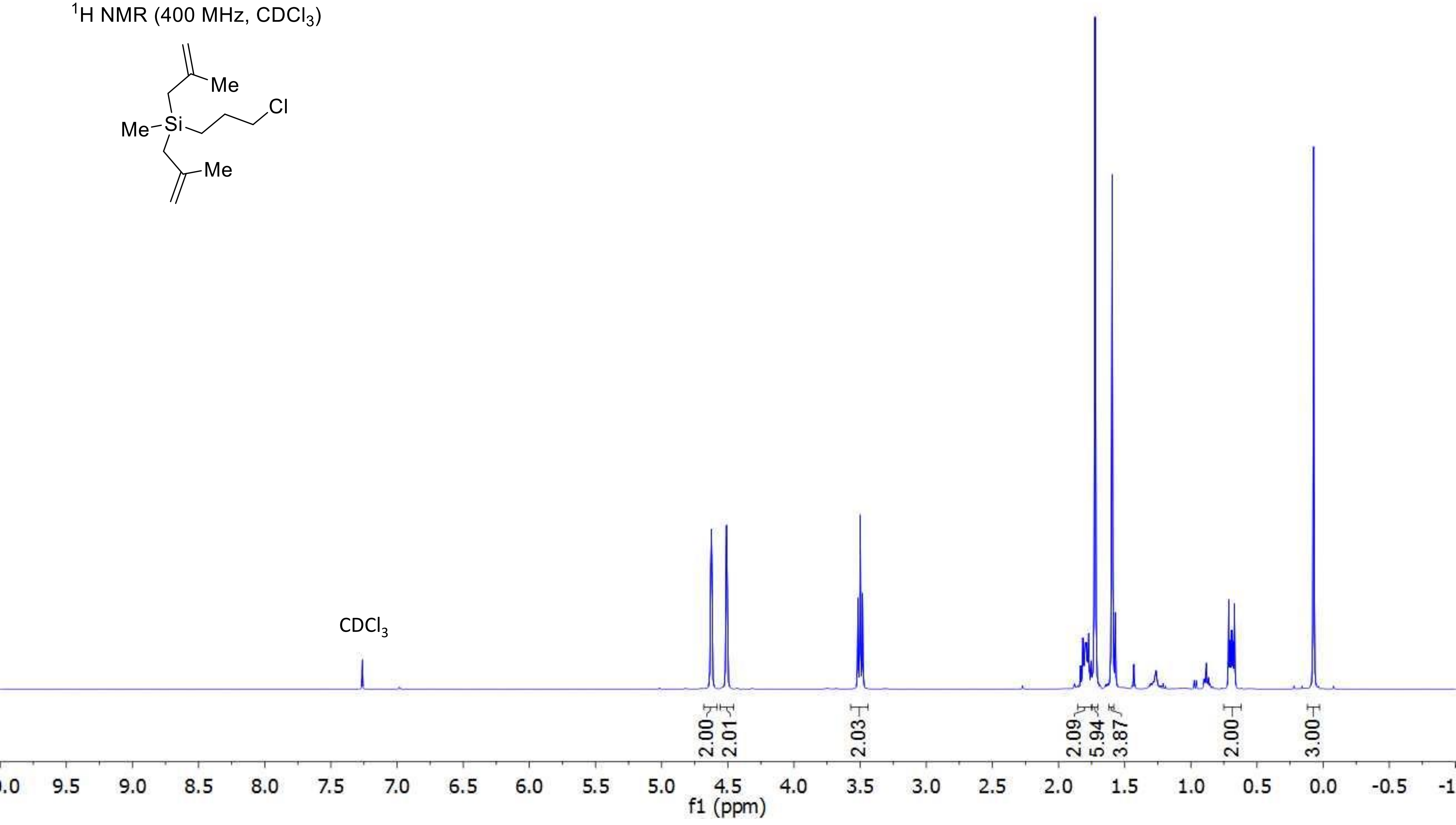

^{1}H NMR (400 MHz, $CDCl_3$)
Me
Cl
Me
Si
Me
$CDCl_3$
2.00
2.01
2.03
2.09
5.94
3.87
2.00
3.00
f1 (ppm)

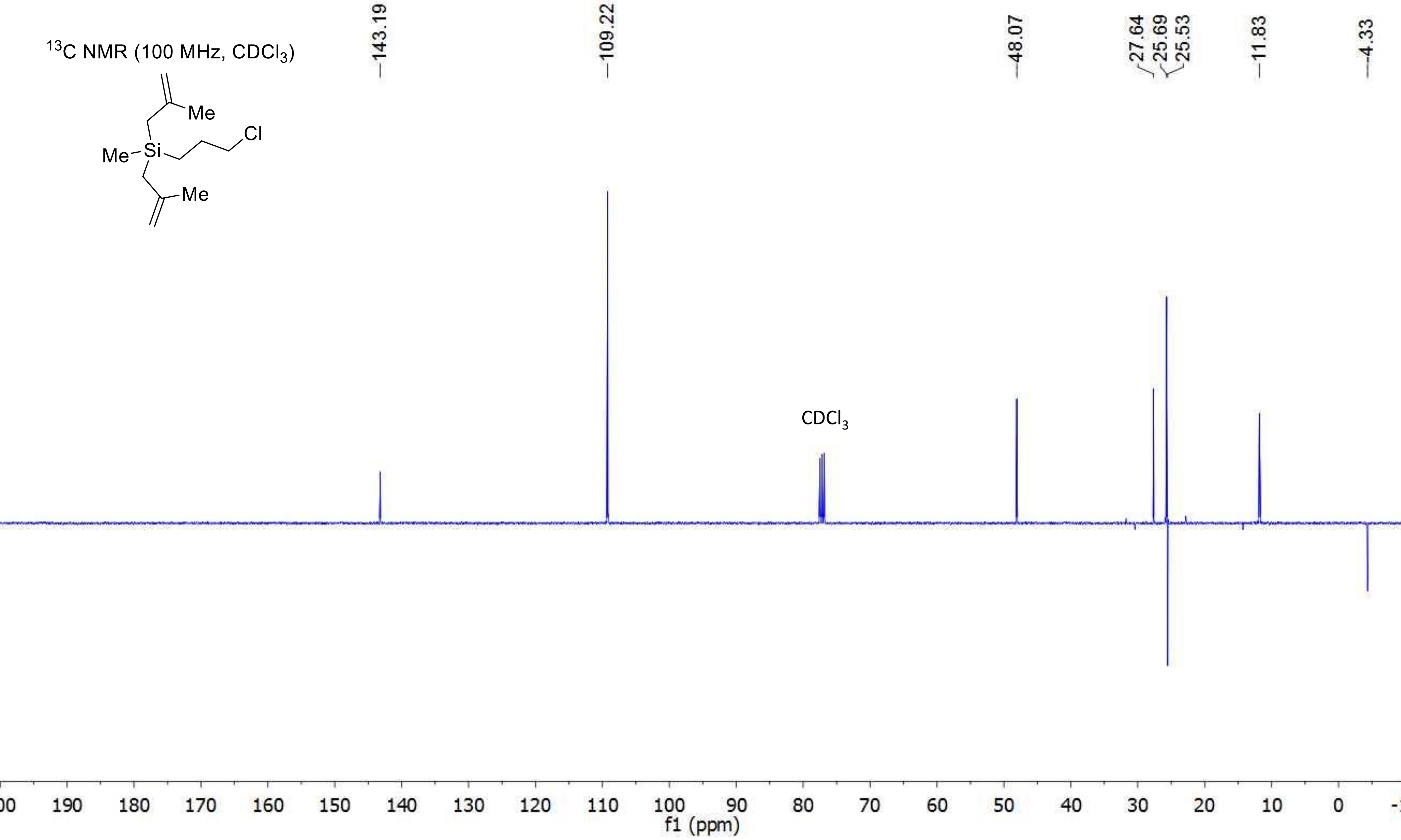

13C NMR (100 MHz, CDCl3)
Me
Cl
Me
Si
Me
143.19
109.22
48.07
27.64
25.69
25.53
11.83
-4.33
CDCl3
190
180
170
160
150
140
130
120
110
100
90
80
70
60
50
40
30
20
10
0
f1 (ppm)

^{1}H NMR (600 MHz, $CDCl_3$)

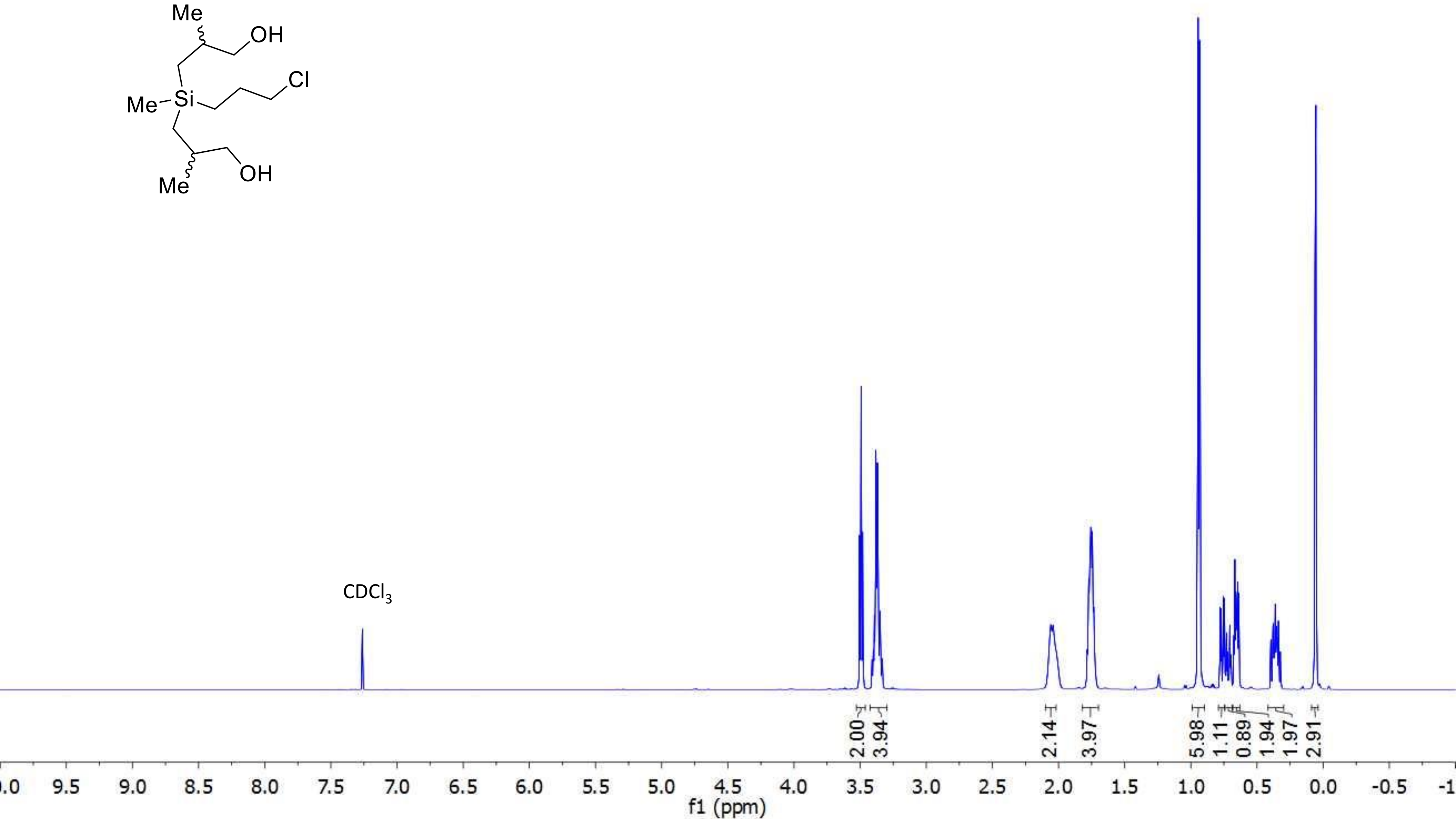

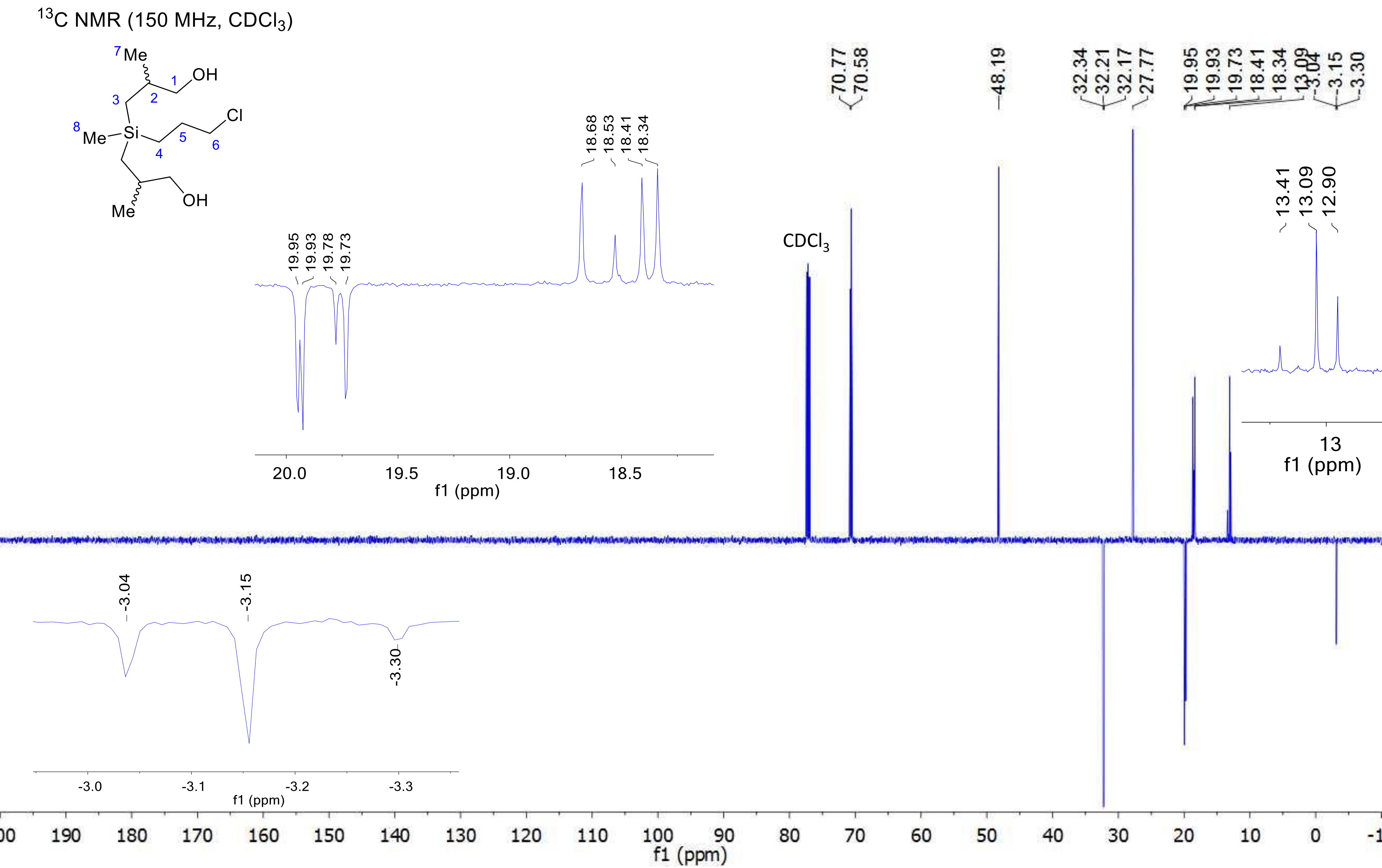

^{13}C NMR (150 MHz, $CDCl_3$)
7 Me
1 OH
2
3
8 Me
Si
4
5
6
Cl
Me
OH
CDCl3
70.77
70.58
48.19
32.34
32.21
32.17
27.77
19.95
19.93
19.73
18.41
18.34
13.09
-3.04
-3.15
-3.30
18.68
18.53
18.41
18.34
19.95
19.93
19.78
19.73
13.41
13.09
12.90
-3.04
-3.15
-3.30
f1 (ppm)

^{1}H NMR (600 MHz, $CDCl_3$)

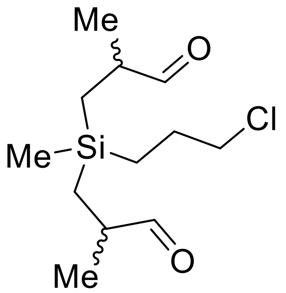

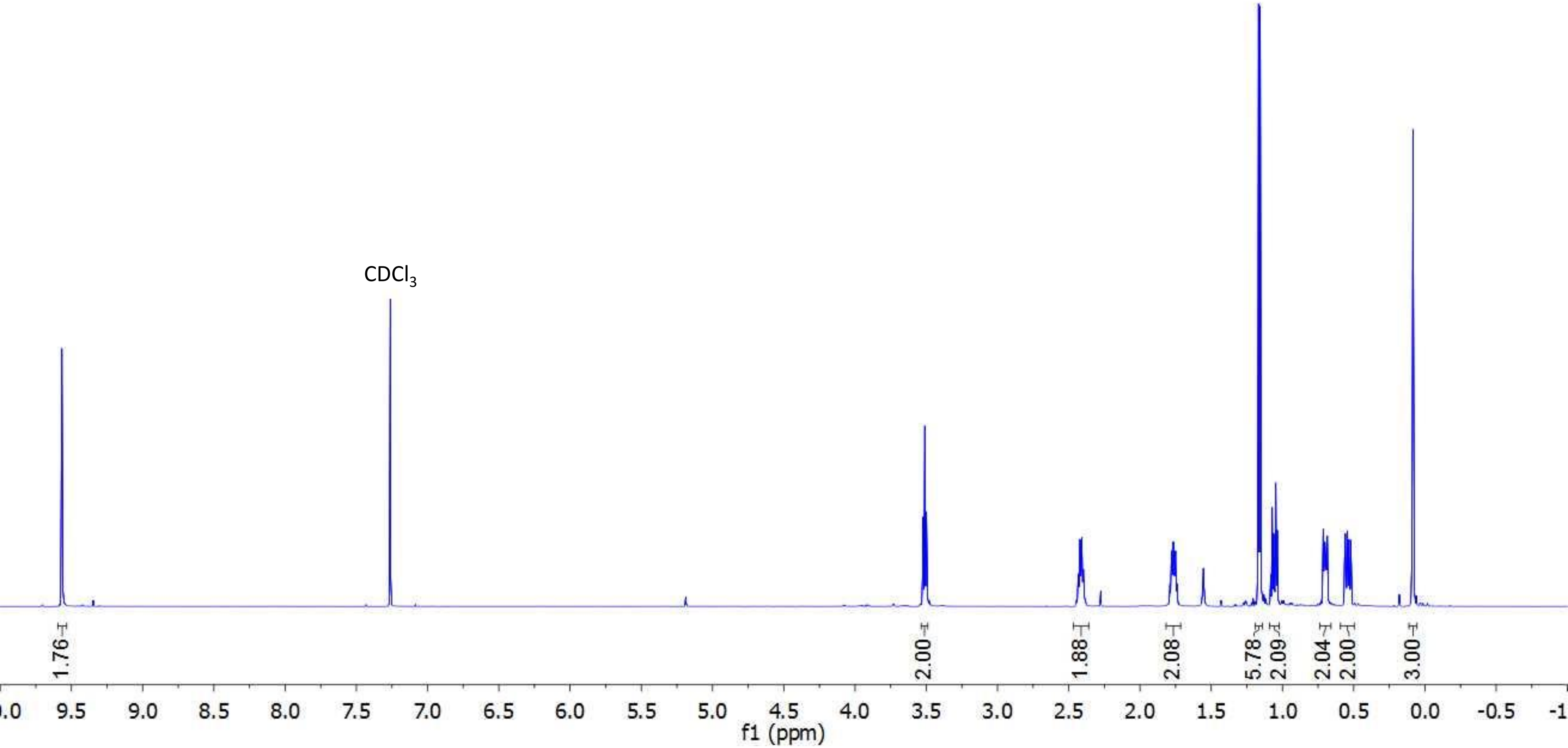

^{13}C NMR (150 MHz, $CDCl_3$)

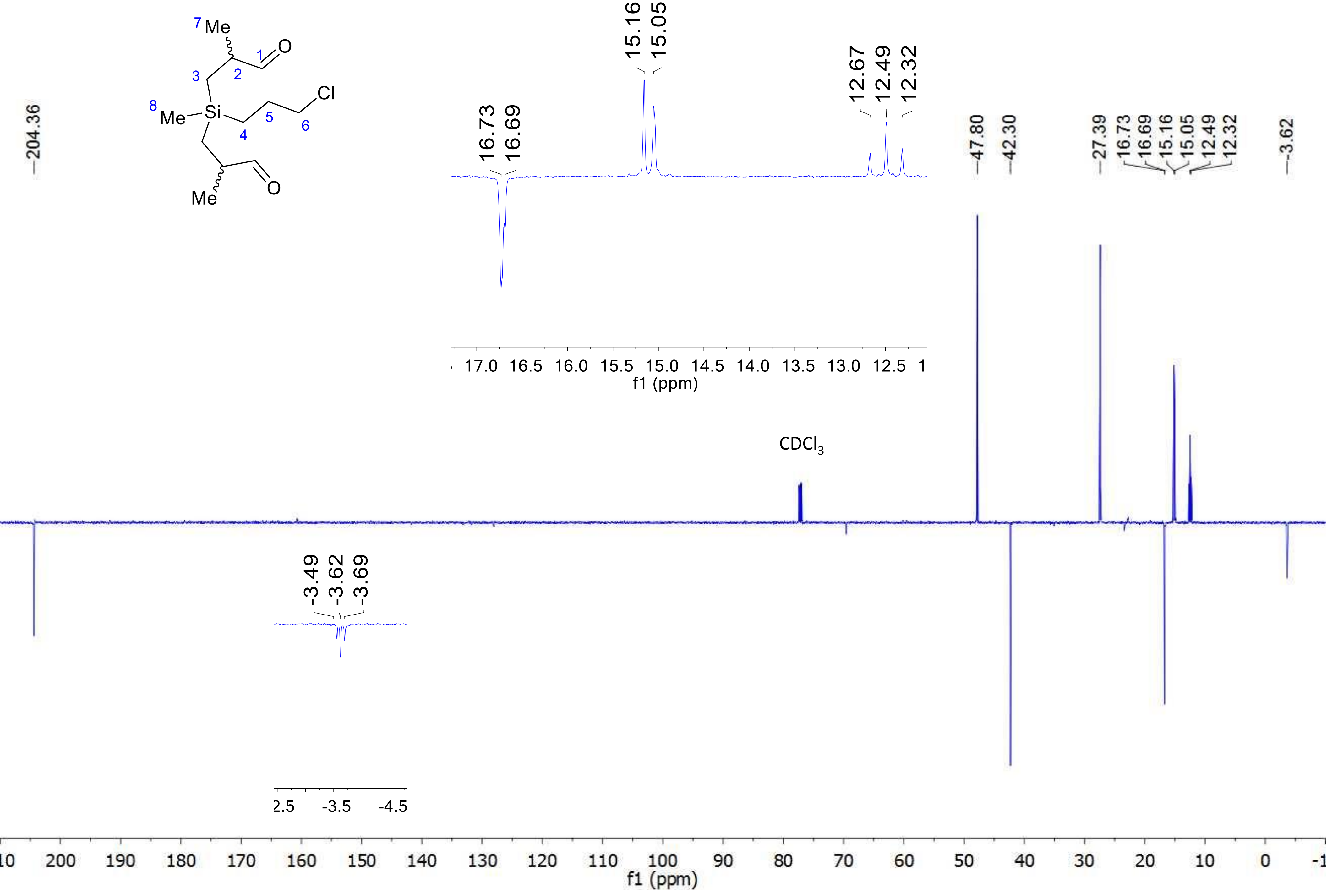

^1H NMR (600 MHz, $CDCl_3$)

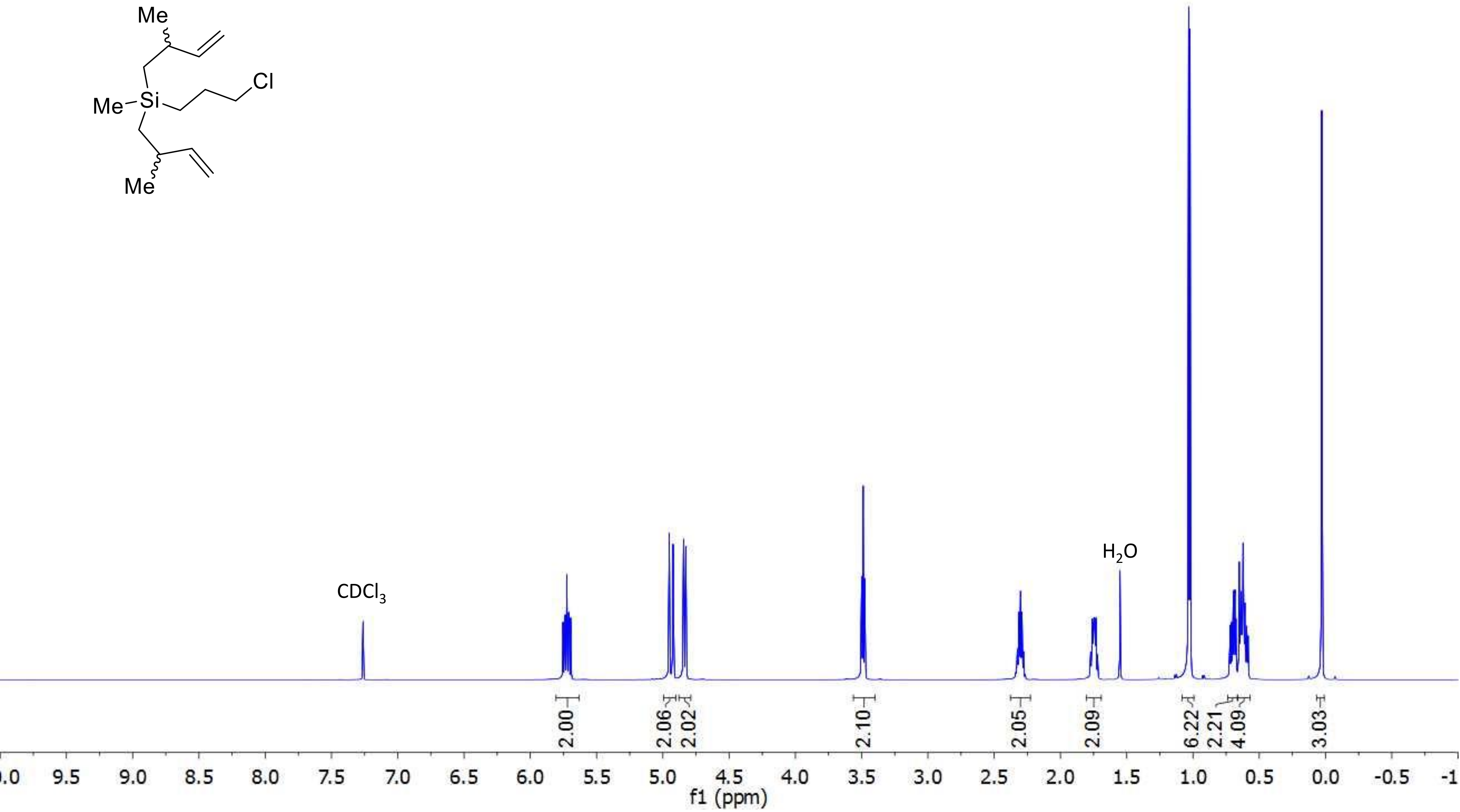

^{13}C NMR (150 MHz, $CDCl_3$)

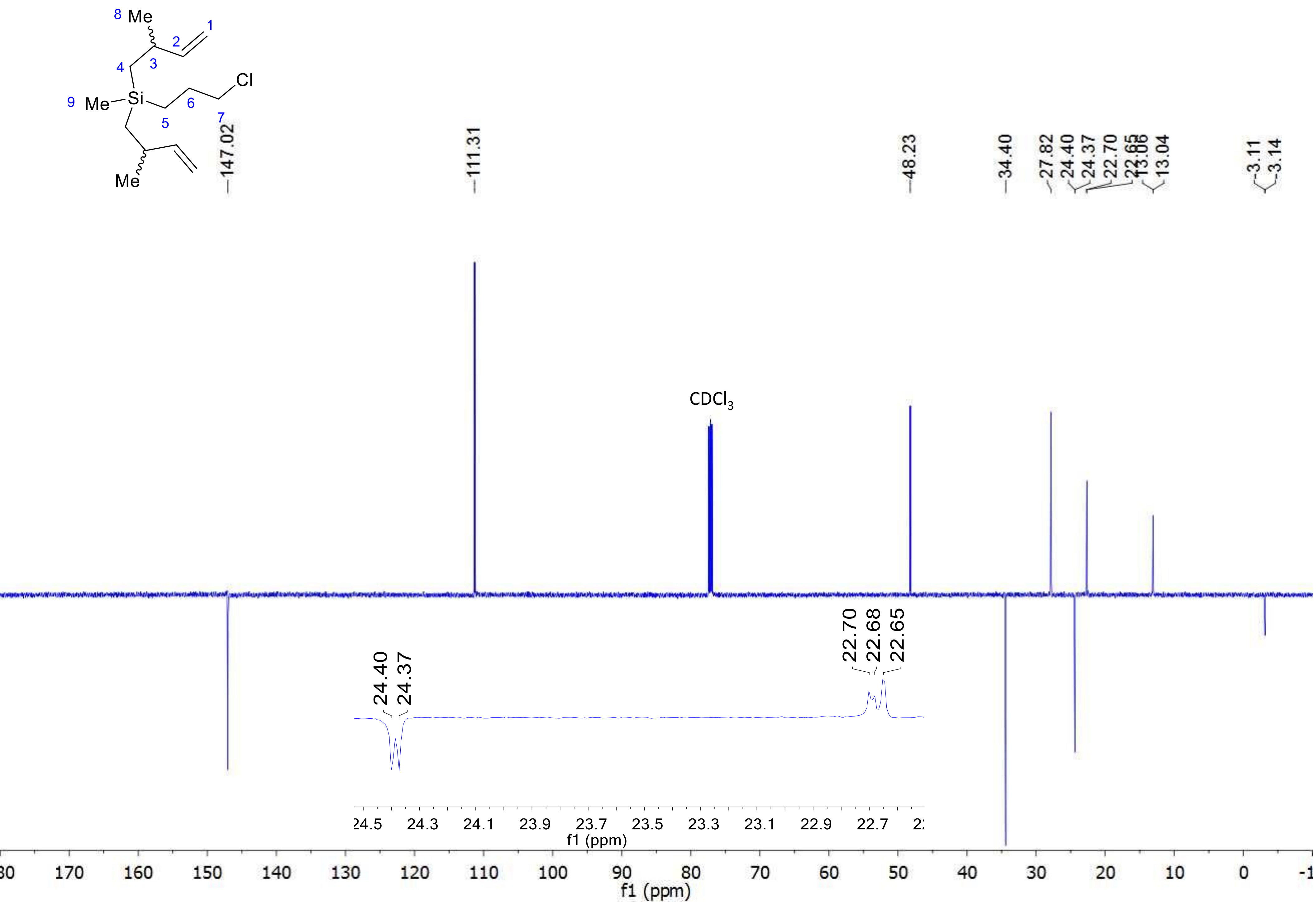

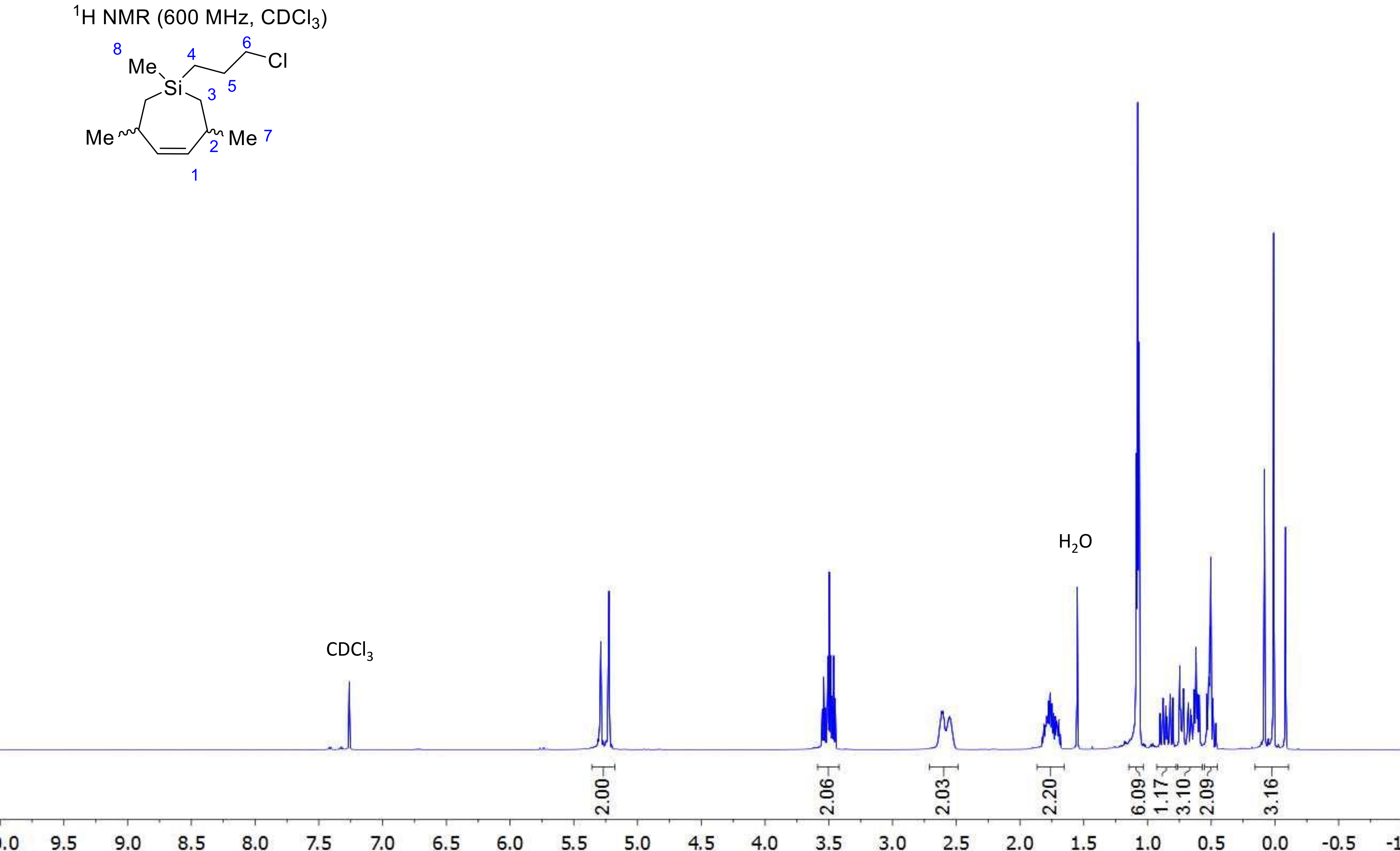

1H NMR (600 MHz, CDCl3)
8
Me
Si
4
6
Cl
3
5
Me
2
Me
7
1
CDCl3
H2O
2.00
2.06
2.03
2.20
6.09
1.17
3.10
2.09
3.16
9.5
9.0
8.5
8.0
7.5
7.0
6.5
6.0
5.5
5.0
4.5
4.0
3.5
3.0
2.5
2.0
1.5
1.0
0.5
0.0
-0.5

^{13}C NMR (150 MHz, $CDCl_3$)

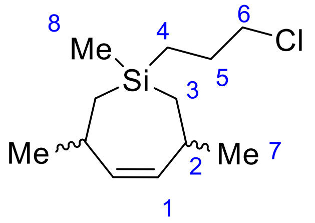

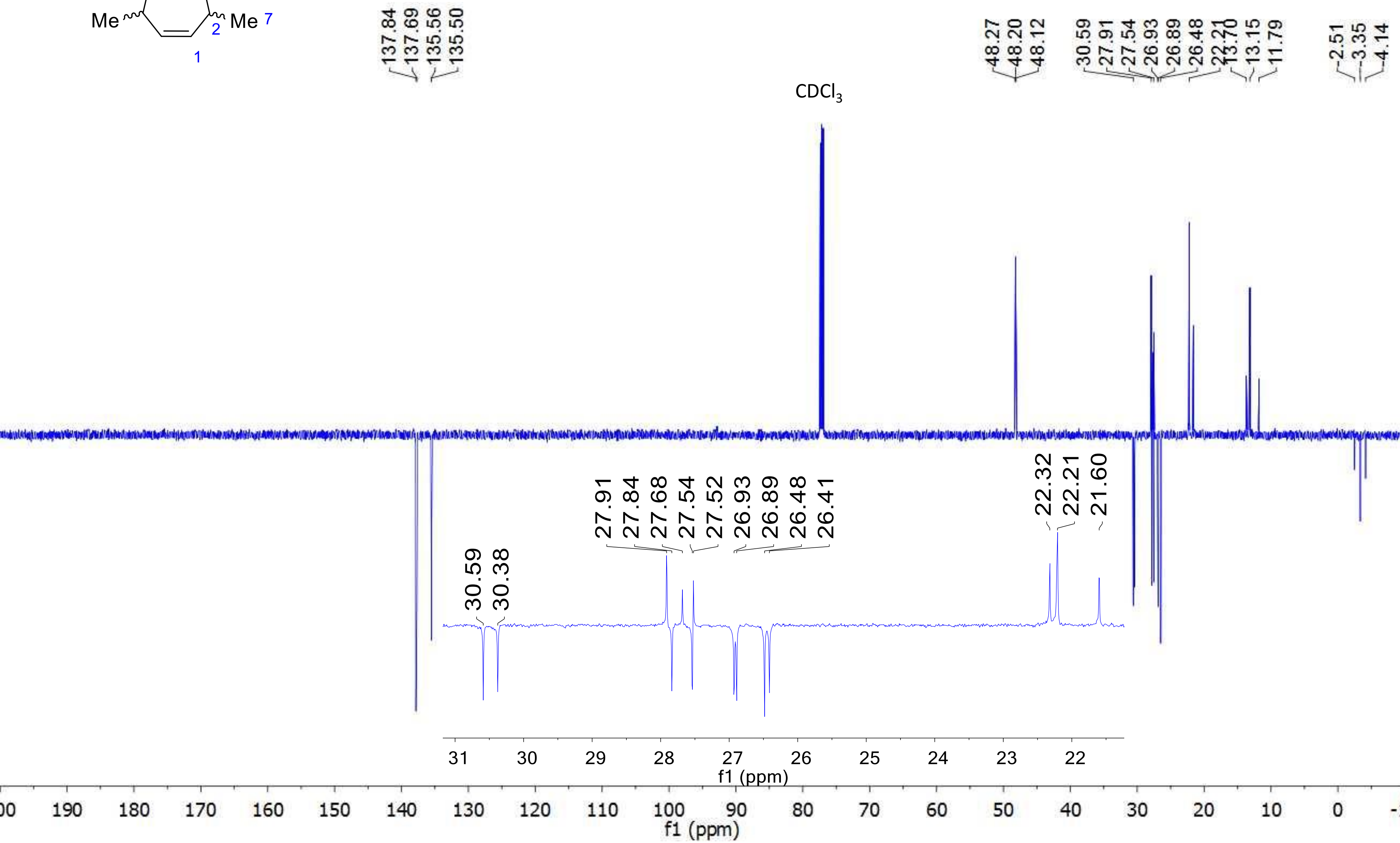

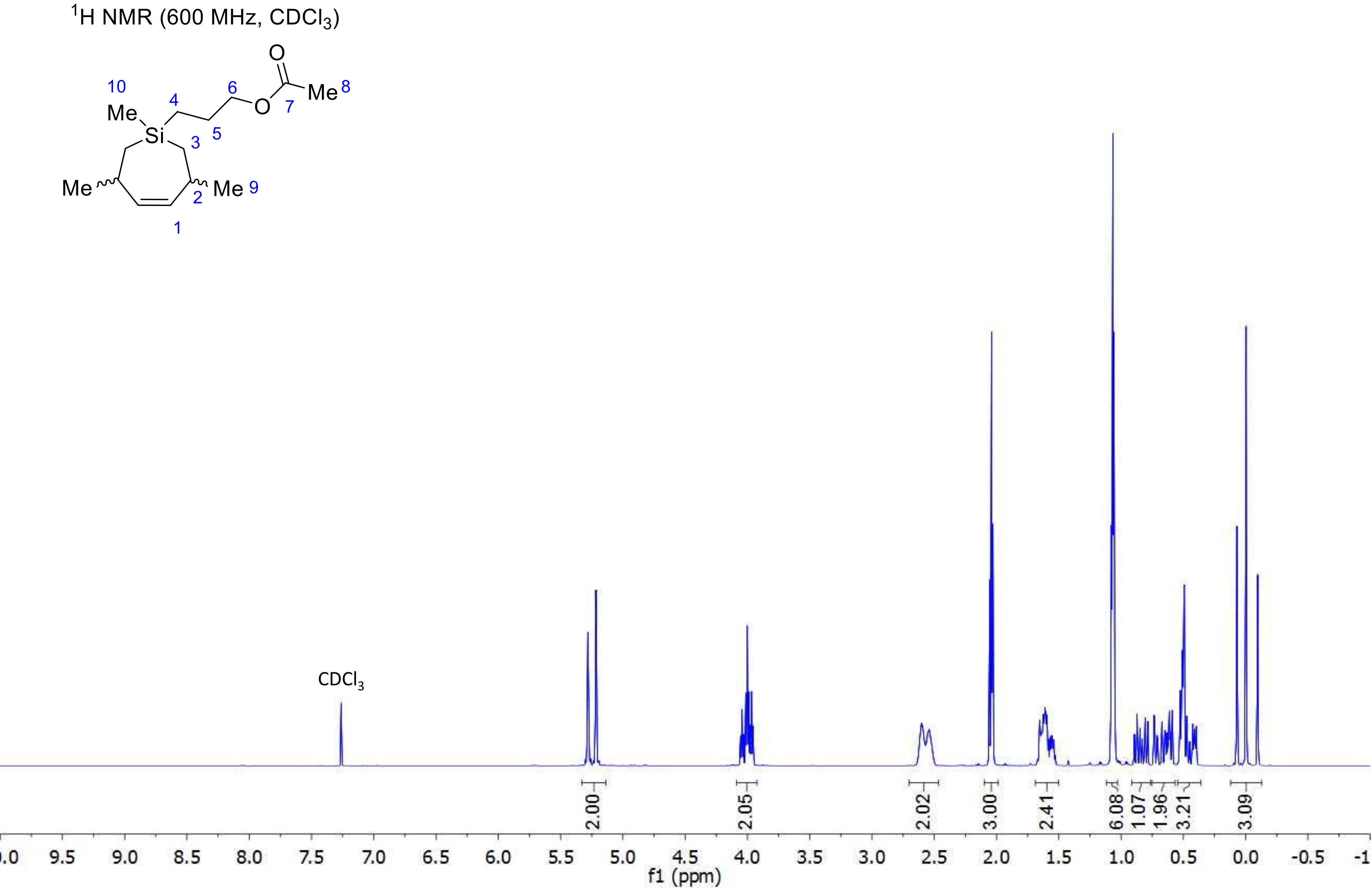

1H NMR (600 MHz, CDCl3)
O
Me 8
10
Me
4
6
O
7
Si
5
3
Me
Me 9
2
1
CDCl3
2.00
2.05
2.02
3.00
2.41
6.08
1.07
1.96
3.21
3.09
9.5
9.0
8.5
8.0
7.5
7.0
6.5
6.0
5.5
5.0
4.5
4.0
3.5
3.0
2.5
2.0
1.5
1.0
0.5
0.0
-0.5
f1 (ppm)

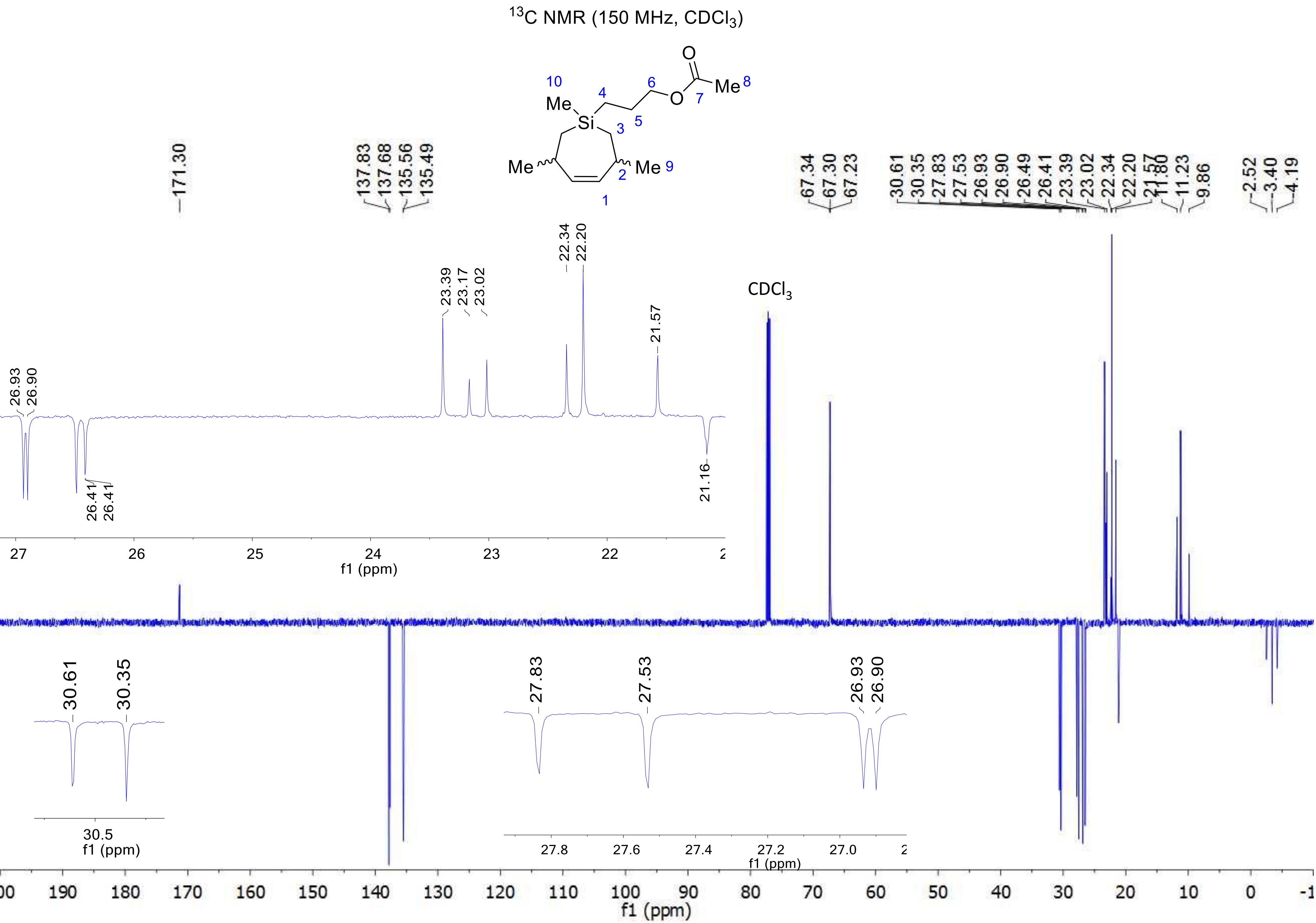

^{13}C NMR (150 MHz, $CDCl_3$)
O
Me 8
10
Me
Si
4
6
O
7
3
5
Me
Me 9
2
1
171.30
137.83
137.68
135.56
135.49
67.34
67.30
67.23
30.61
30.35
27.83
27.53
26.93
26.90
26.49
26.41
23.39
23.02
22.34
22.20
21.57
11.80
11.23
9.86
-2.52
-3.40
-4.19
CDCl3
23.17
21.16
f1 (ppm)

^{1}H NMR (600 MHz, $CDCl_3$)

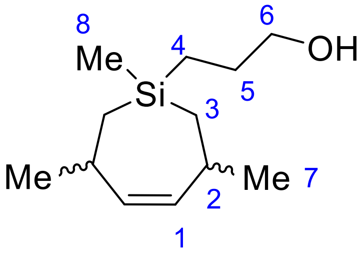

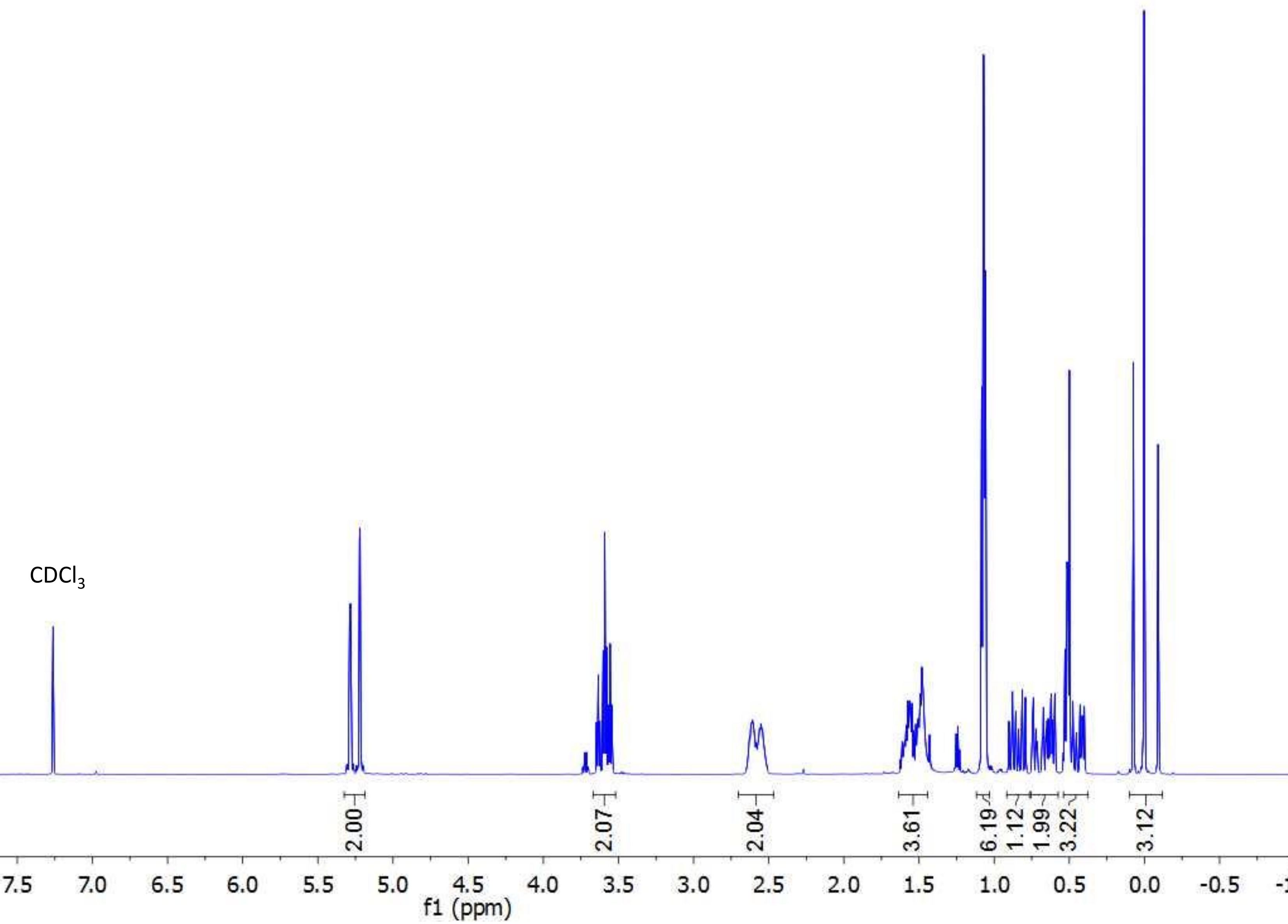

^{13}C NMR (150 MHz, $CDCl_3$)

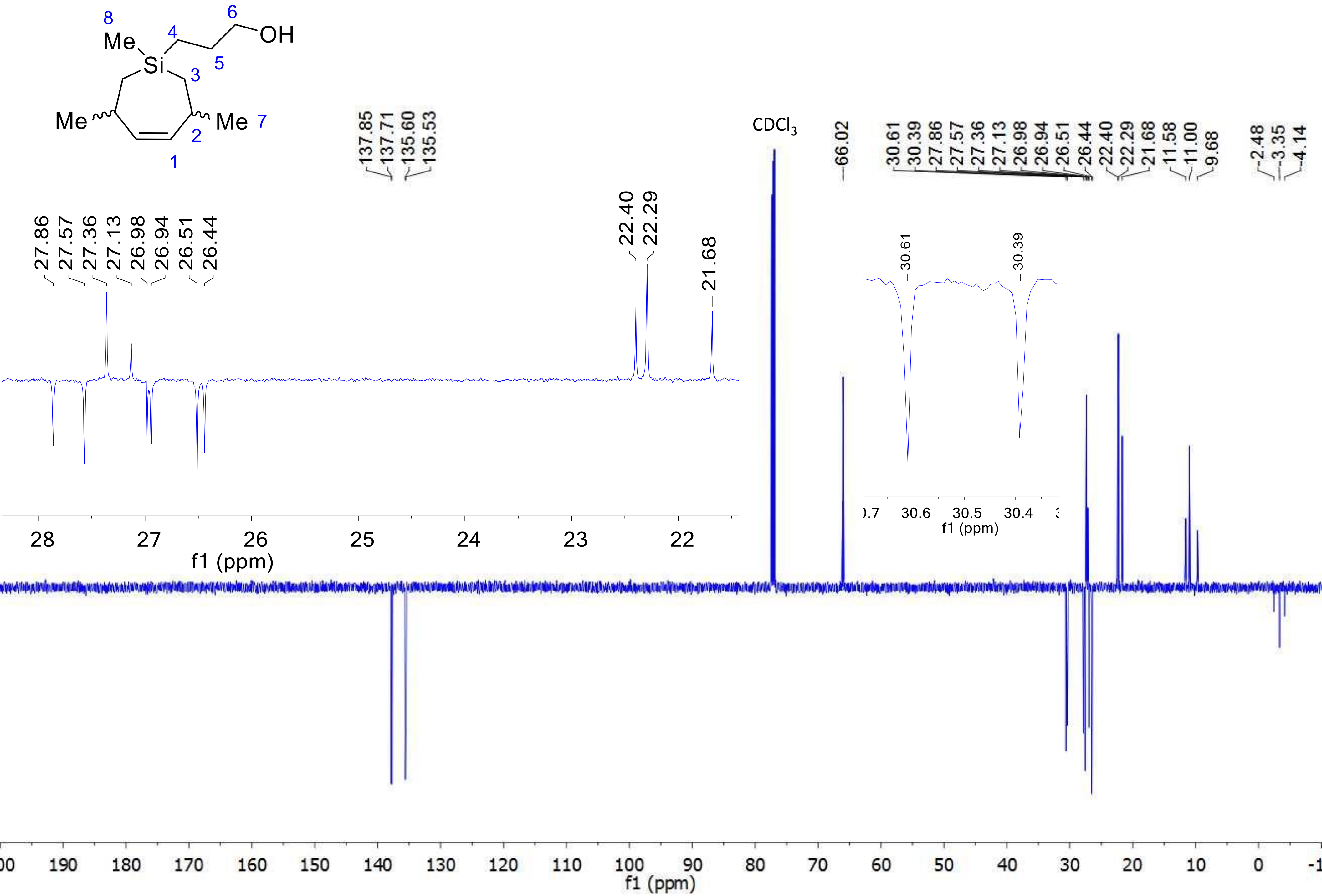

^{1}H NMR (400 MHz, $CDCl_3$)

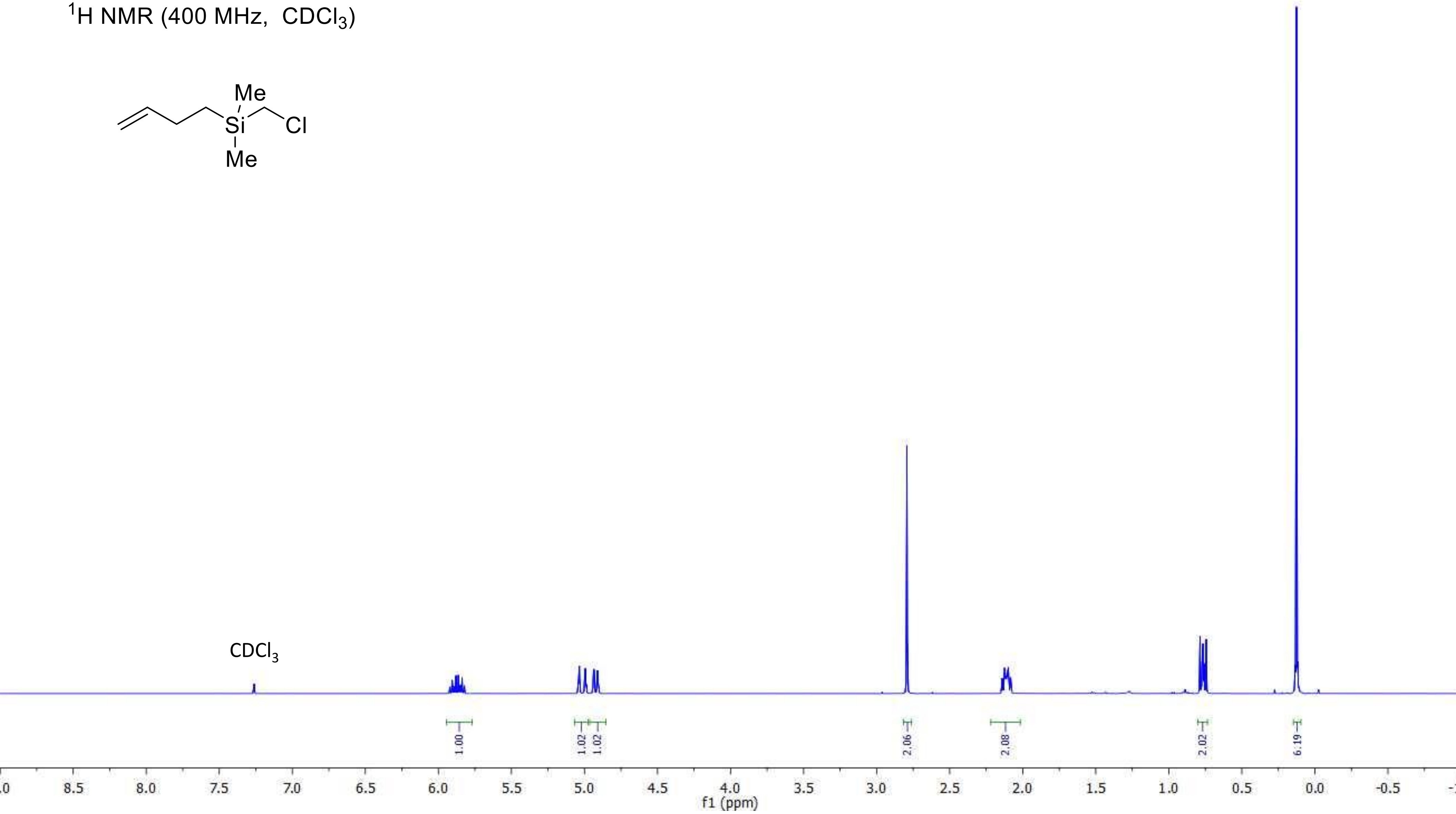

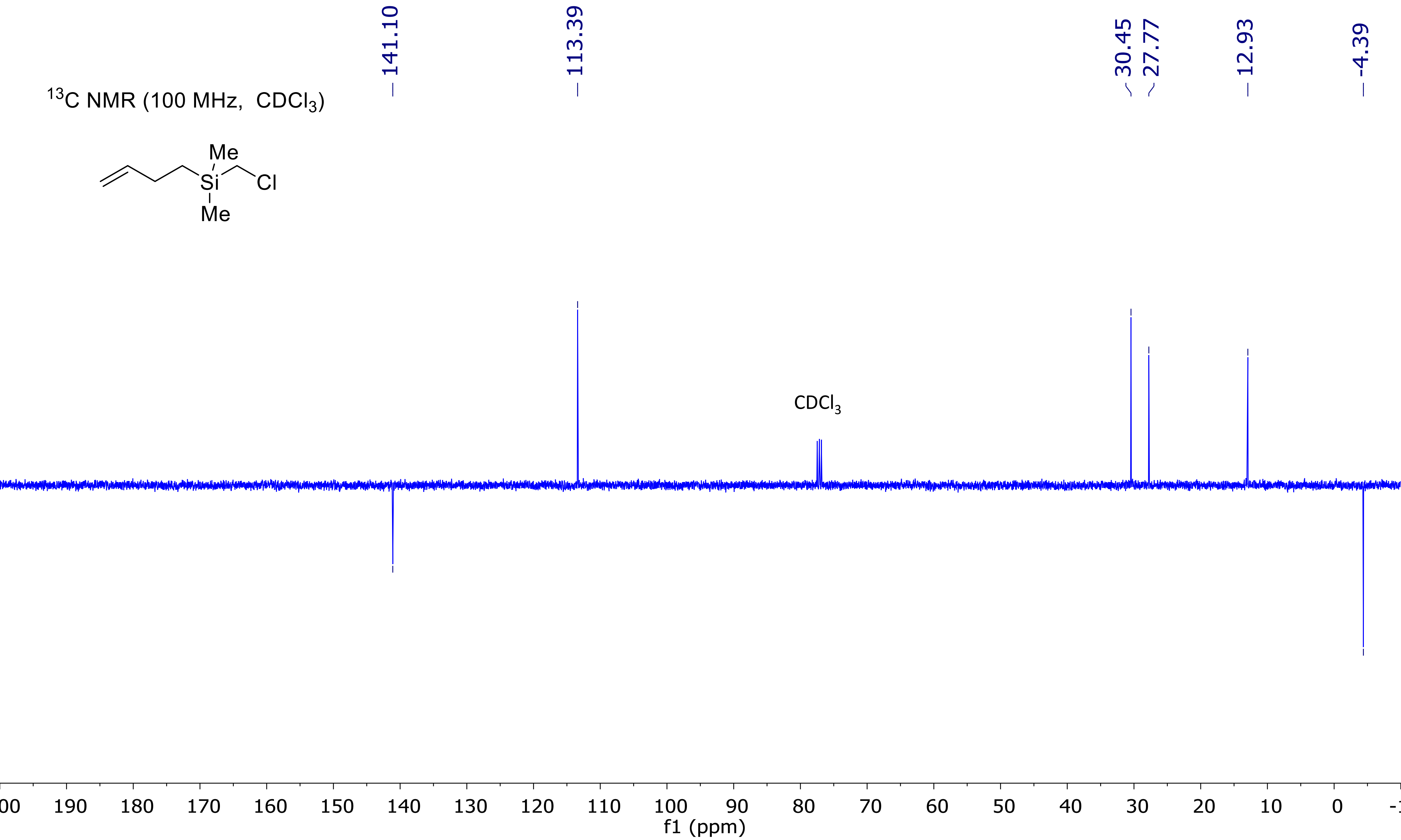

^{13}C NMR (100 MHz, $CDCl_3$)
Me
Si
Cl
Me
141.10
113.39
30.45
27.77
12.93
-4.39
$CDCl_3$
190 180 170 160 150 140 130 120 110 100 90 80 70 60 50 40 30 20 10 0
f1 (ppm)

^{1}H NMR (600 MHz, $CDCl_3$)

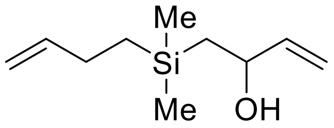

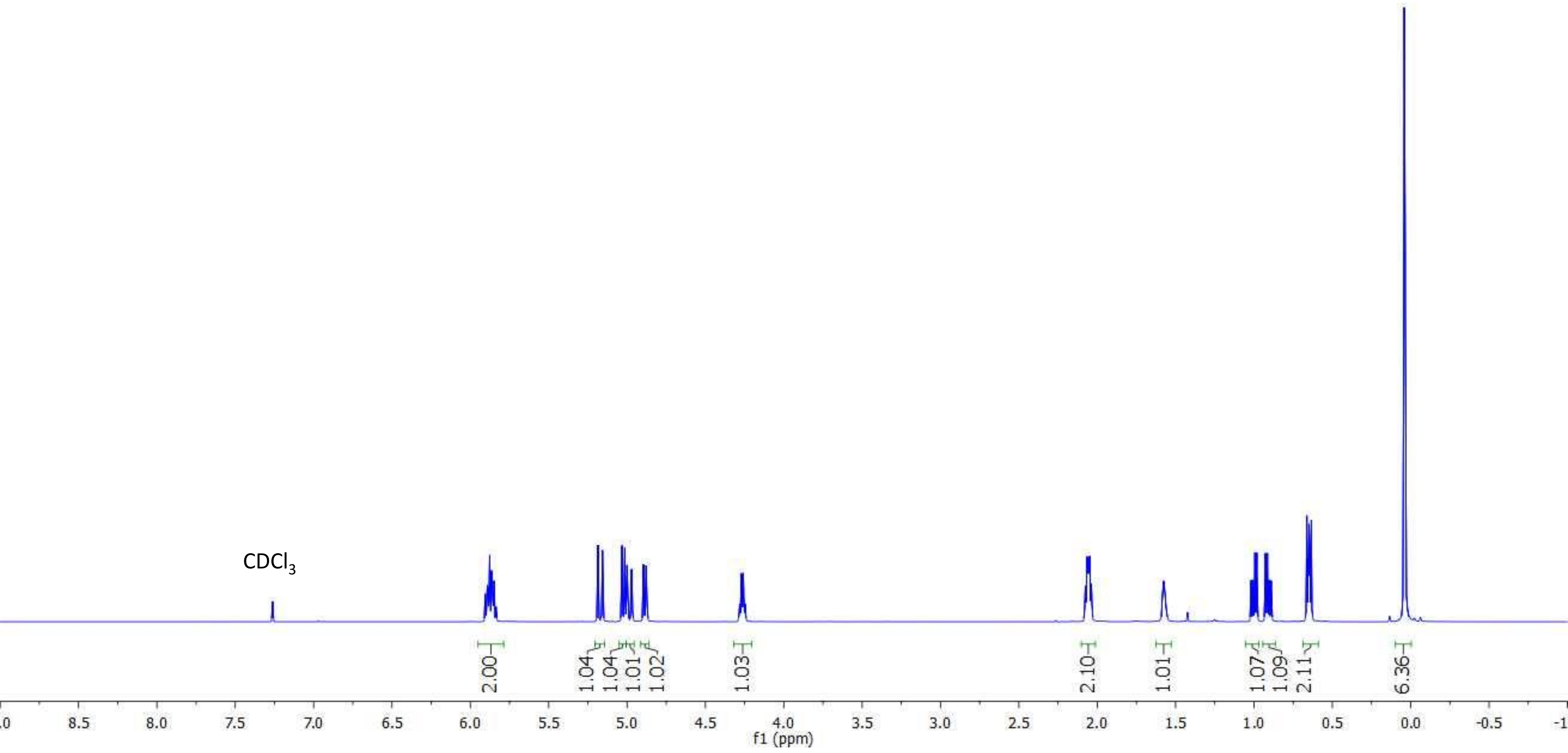

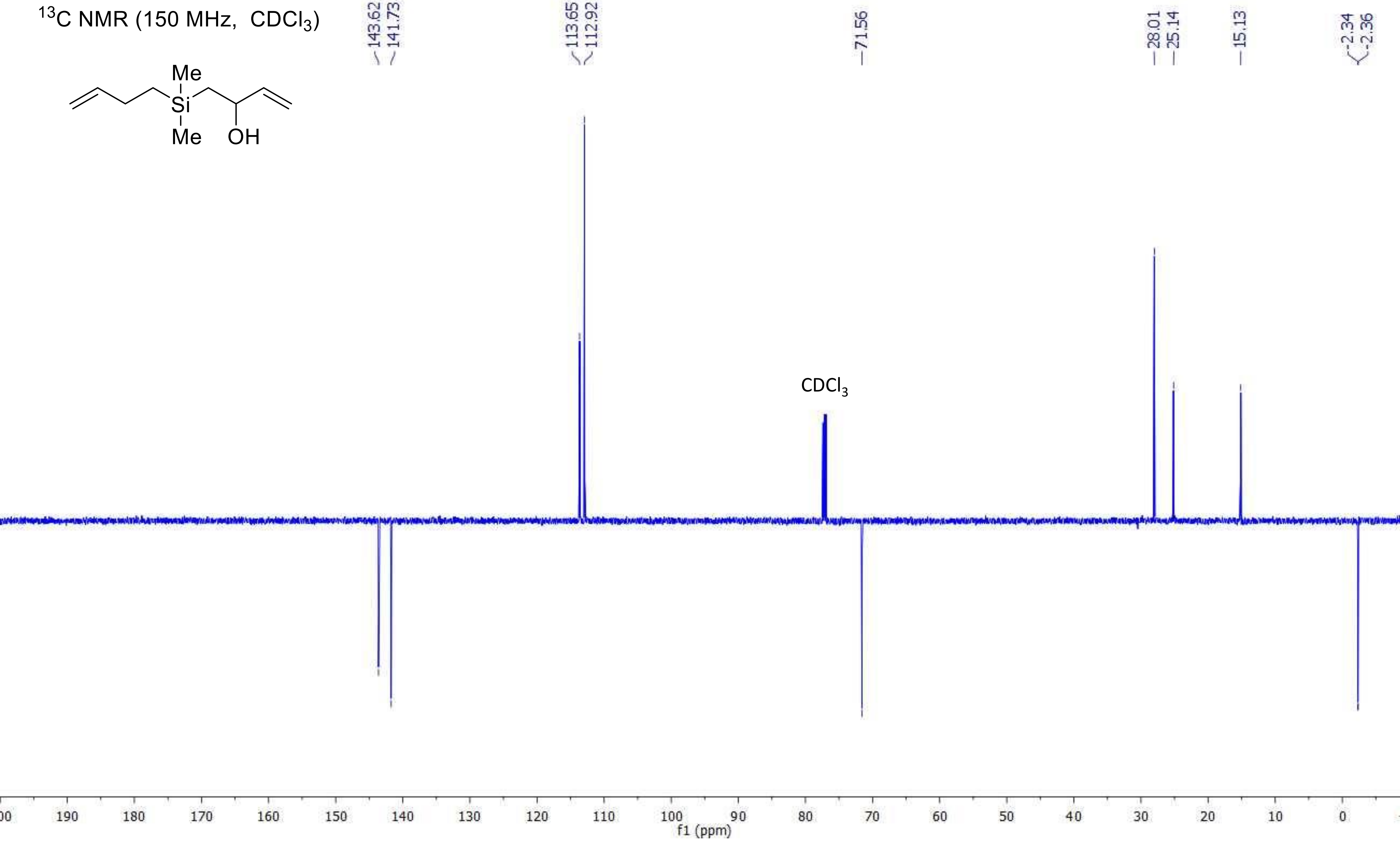

^{13}C NMR (150 MHz, $CDCl_3$)
Me
Si
Me
OH
143.62
141.73
113.65
112.92
71.56
28.01
25.14
15.13
-2.34
-2.36
$CDCl_3$
190
180
170
160
150
140
130
120
110
100
90
80
70
60
50
40
30
20
10
0
f1 (ppm)

^{1}H NMR (400 MHz, $CDCl_3$)

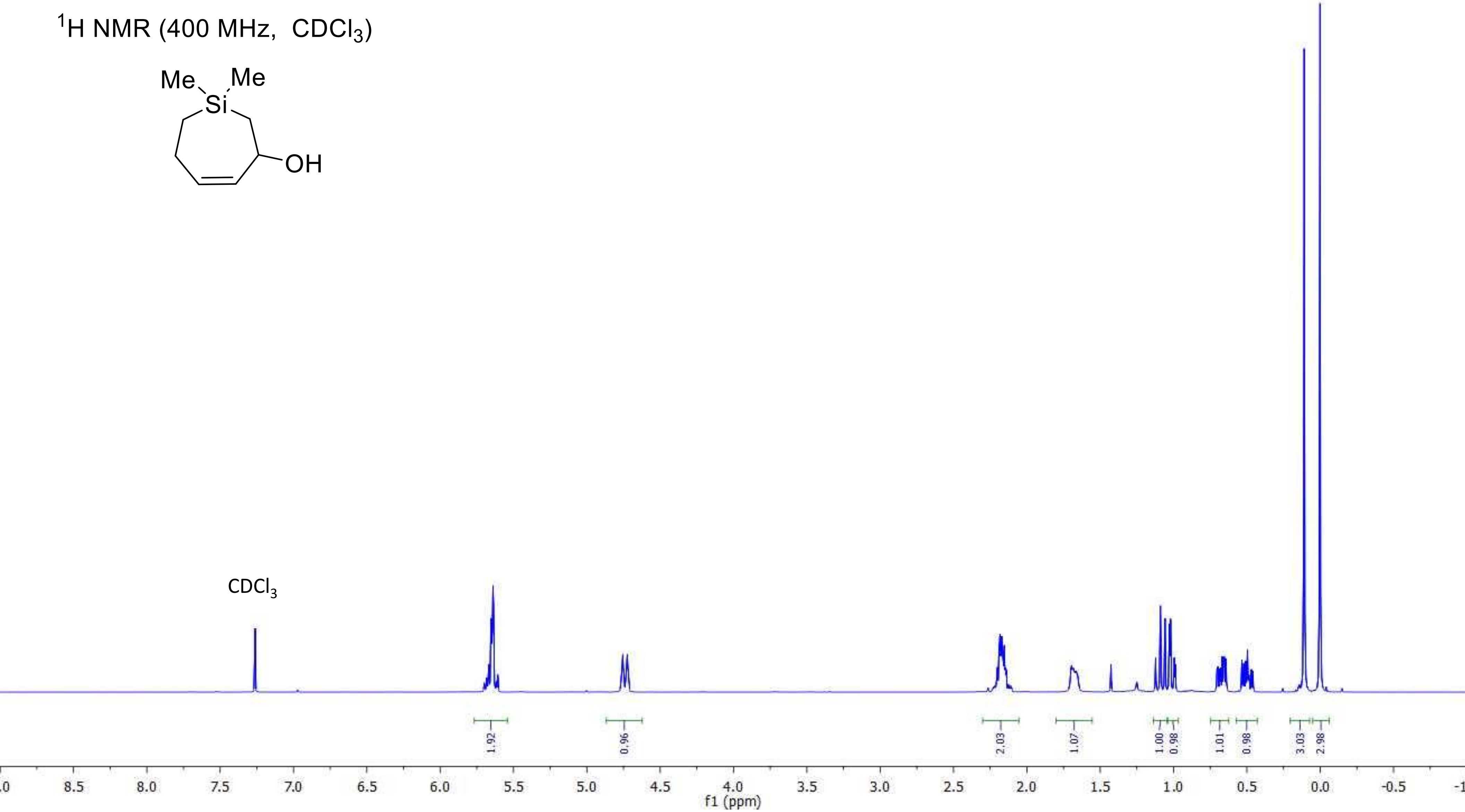

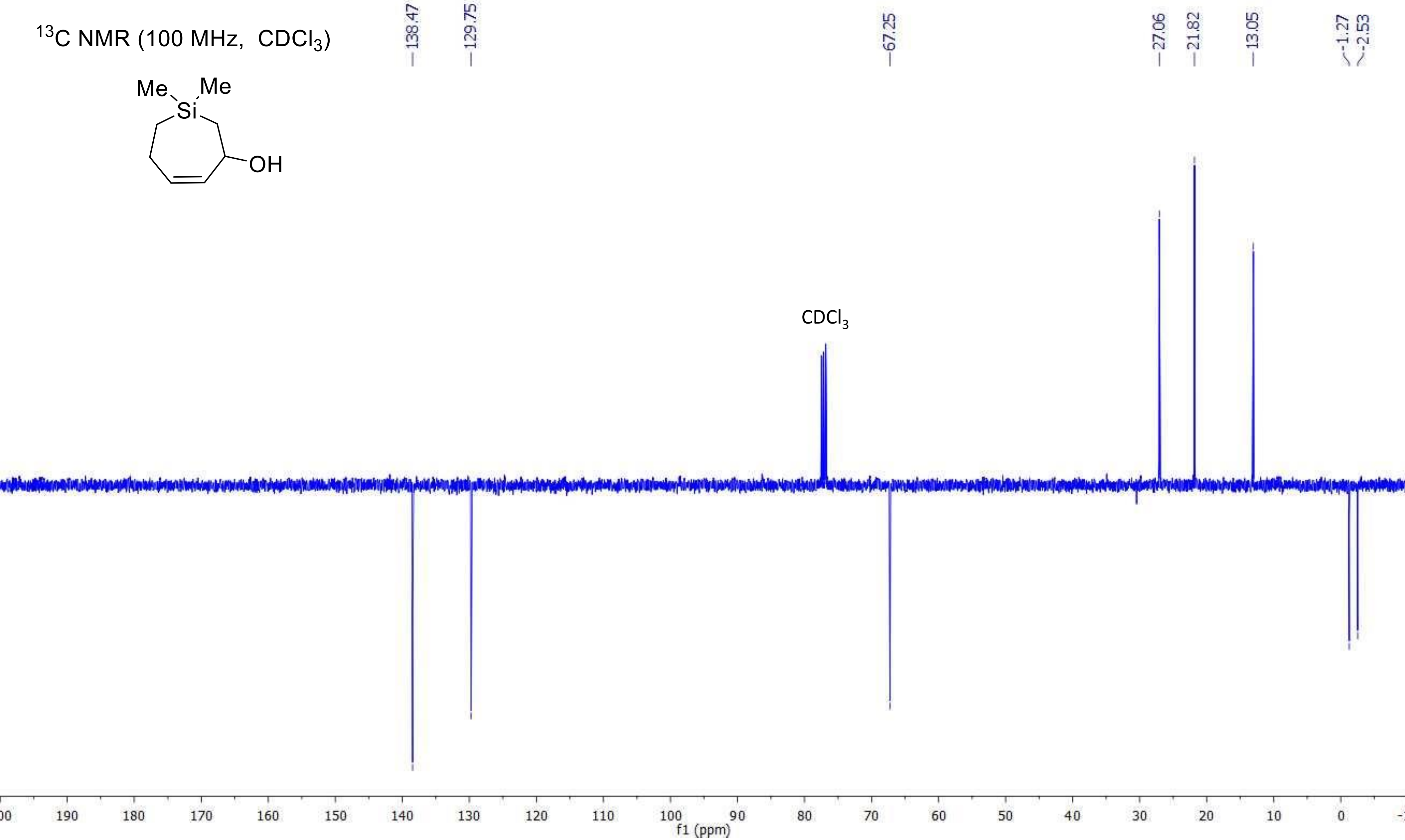

^{13}C NMR (100 MHz, CDCl$_3$)
Me
Me
Si
OH
138.47
129.75
67.25
27.06
21.82
13.05
-1.27
-2.53
CDCl$_3$
190
180
170
160
150
140
130
120
110
100
90
80
70
60
50
40
30
20
10
0
f1 (ppm)

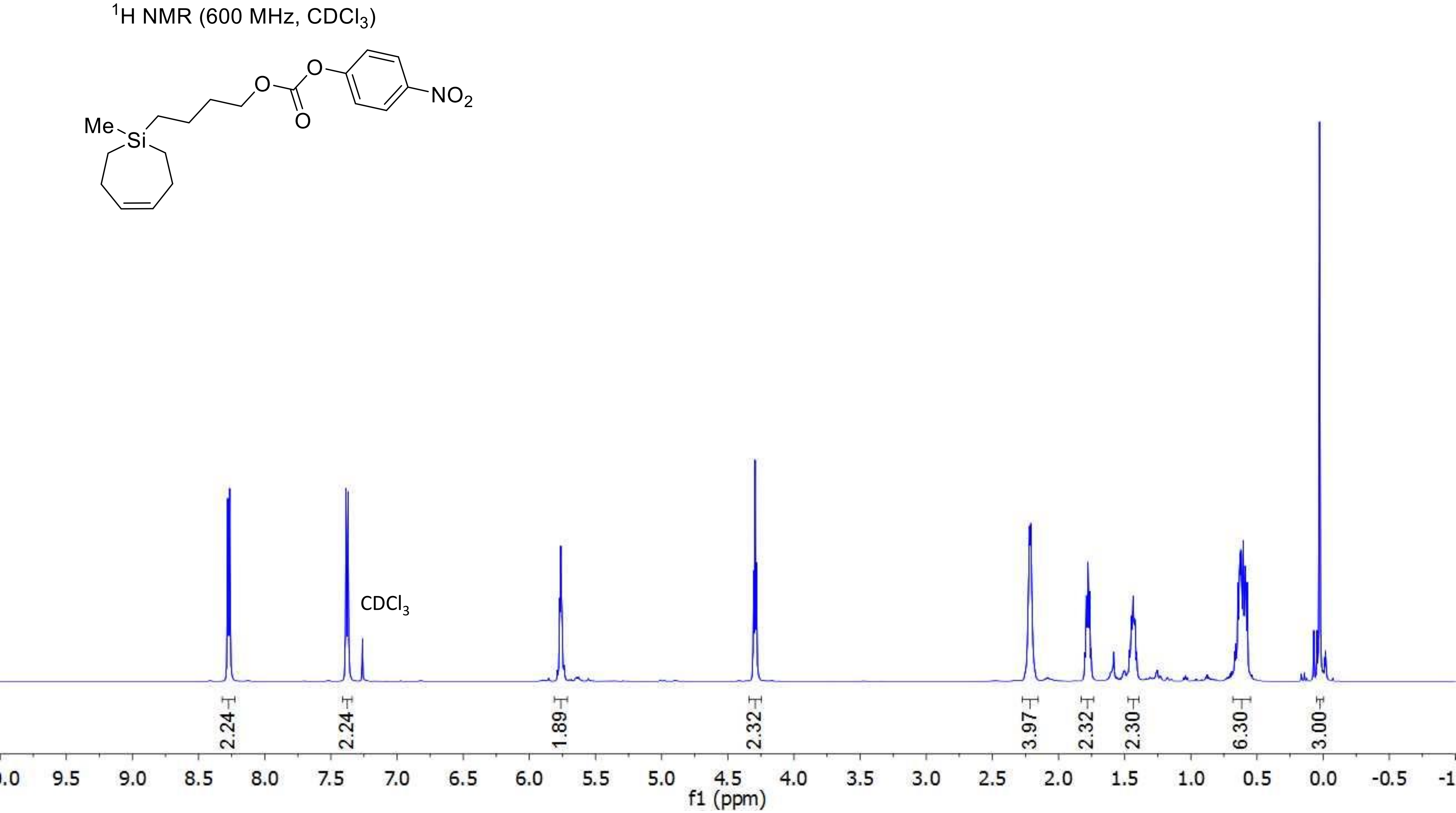

1H NMR (600 MHz, CDCl3)
Me
Si
O
O
O
NO2
CDCl3
2.24
2.24
1.89
2.32
3.97
2.32
2.30
6.30
3.00
9.5 9.0 8.5 8.0 7.5 7.0 6.5 6.0 5.5 5.0 4.5 4.0 3.5 3.0 2.5 2.0 1.5 1.0 0.5 0.0 -0.5
f1 (ppm)

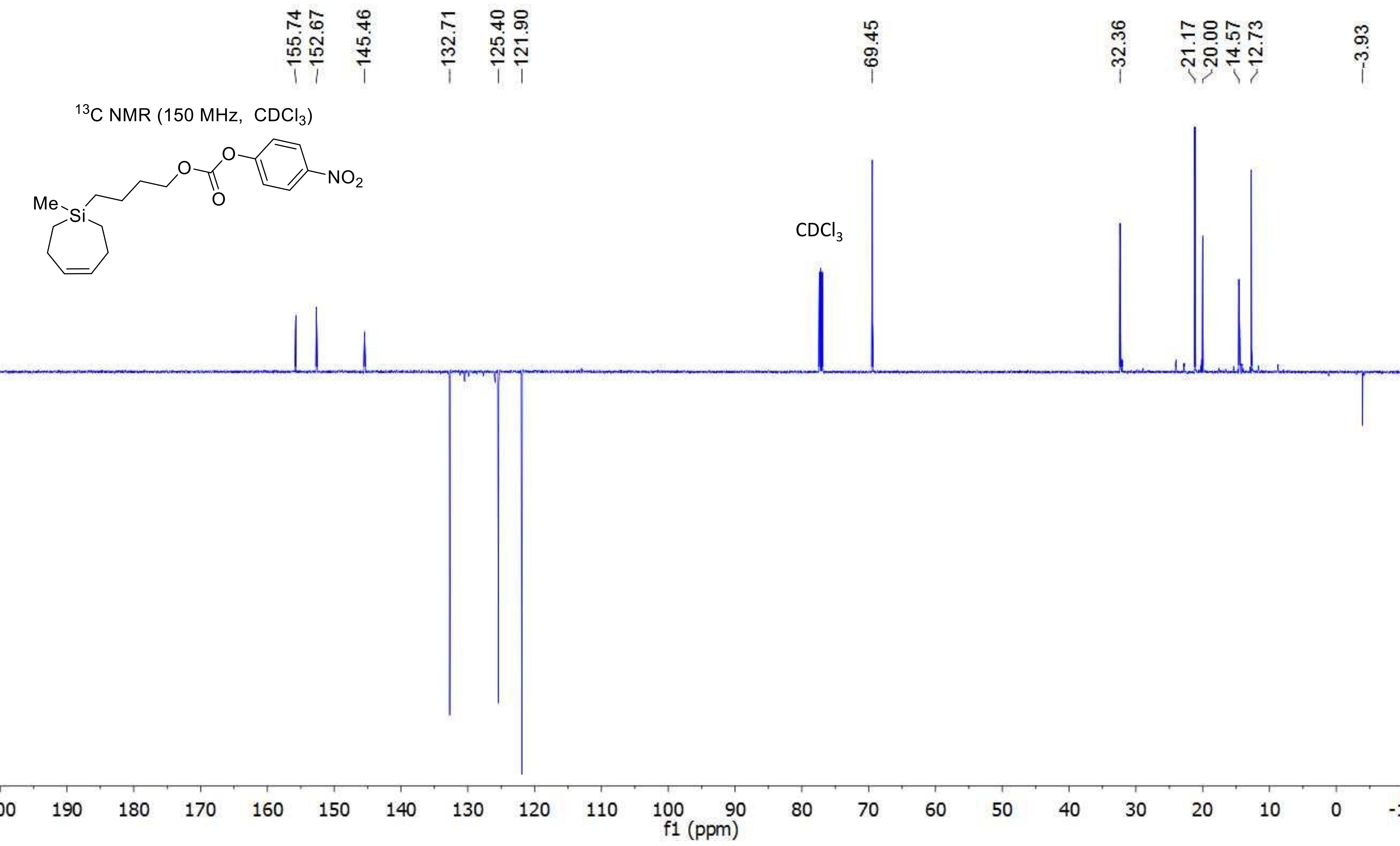

155.74
152.67
145.46
132.71
125.40
121.90
69.45
32.36
21.17
20.00
14.57
12.73
-3.93
13C NMR (150 MHz, CDCl3)
Me
Si
O
O
O
NO2
CDCl3
200 190 180 170 160 150 140 130 120 110 100 90 80 70 60 50 40 30 20 10 0
f1 (ppm)

^{1}H NMR (600 MHz, $CDCl_3$)

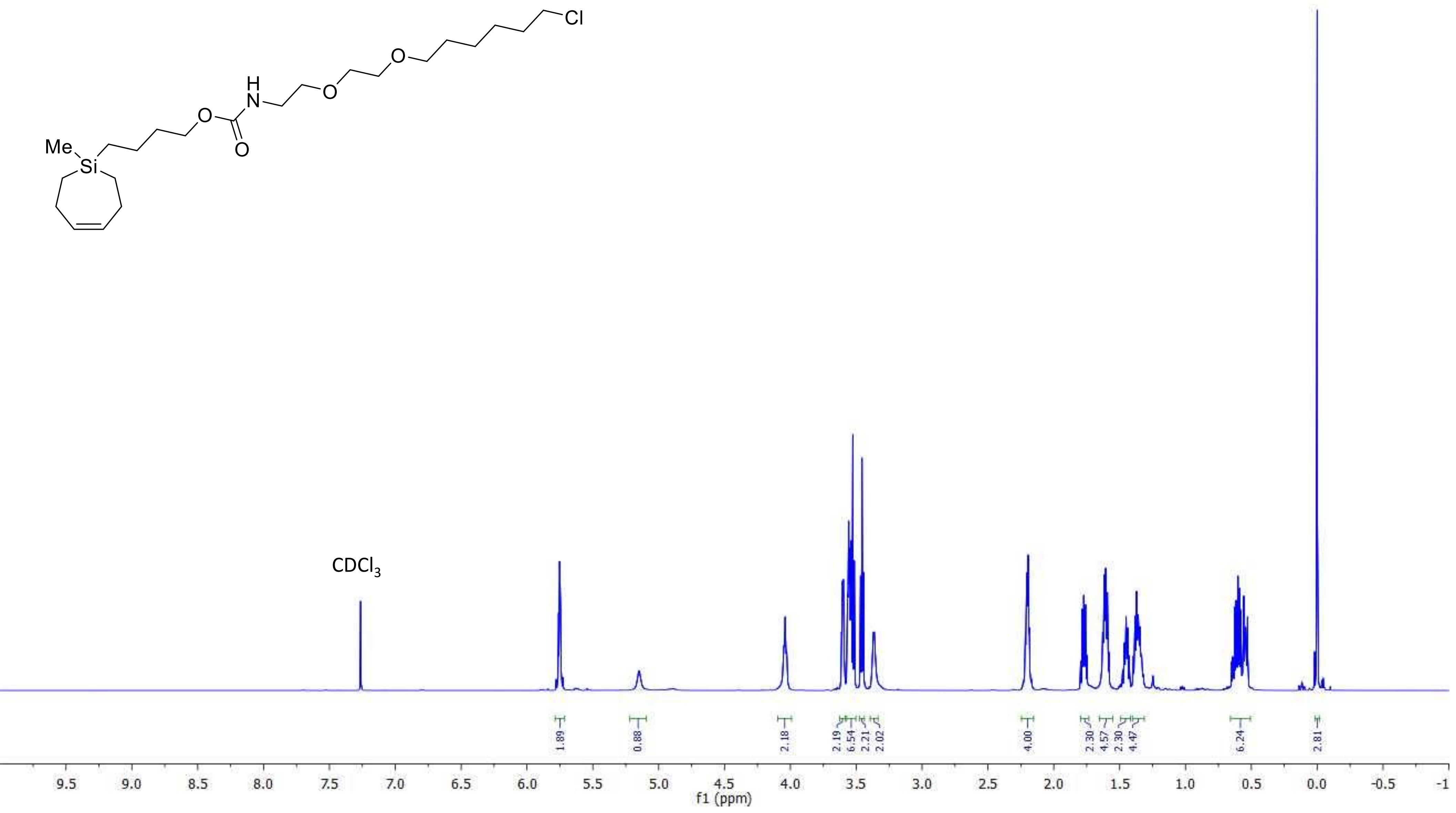

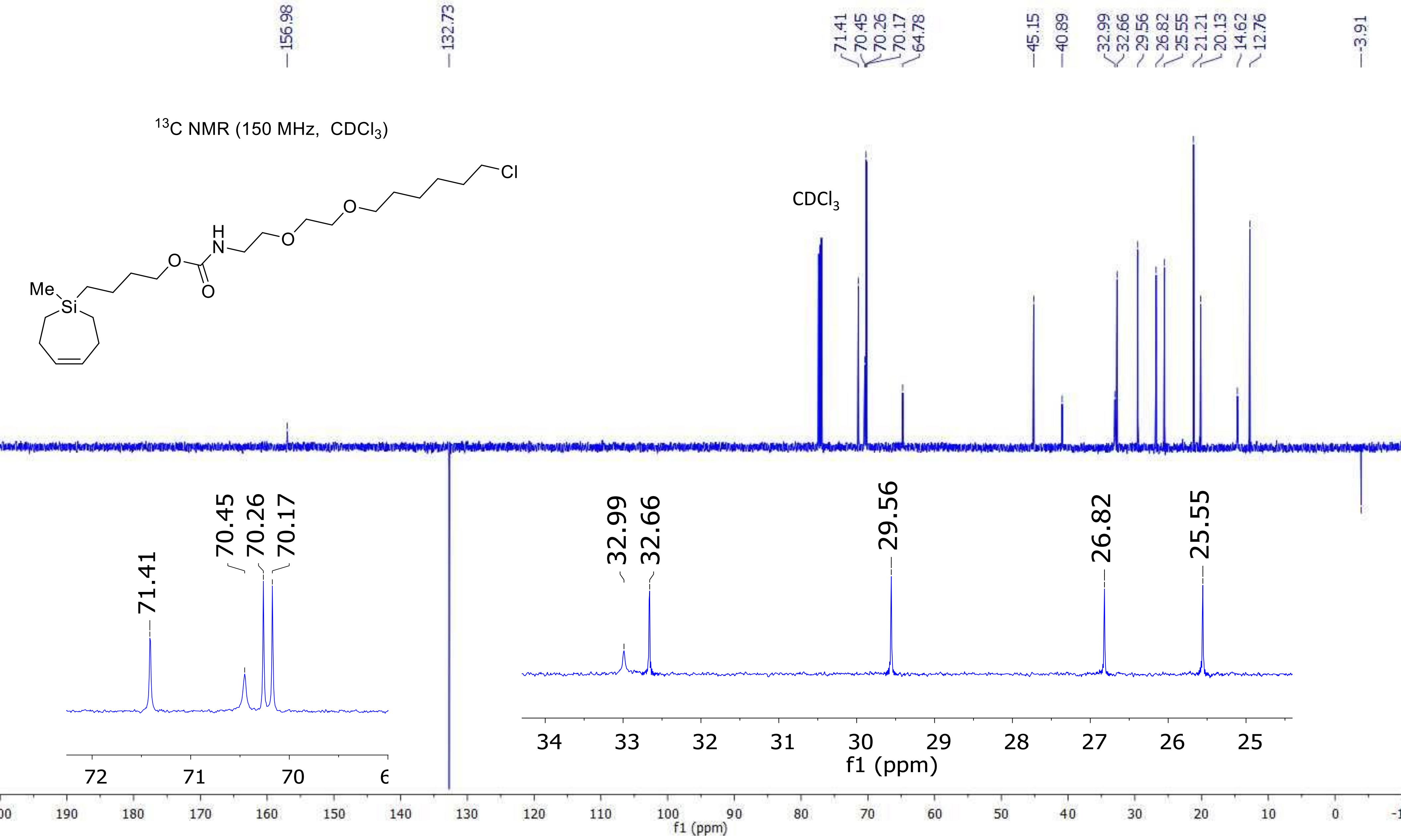

^{13}C NMR (150 MHz, CDCl$_3$)
Me
Si
O
H
N
O
O
O
Cl
CDCl$_3$
156.98
132.73
71.41
70.45
70.26
70.17
64.78
45.15
40.89
32.99
32.66
29.56
26.82
25.55
21.21
20.13
14.62
12.76
-3.91
f1 (ppm)

^{1}H NMR (600 MHz, $CDCl_3$)

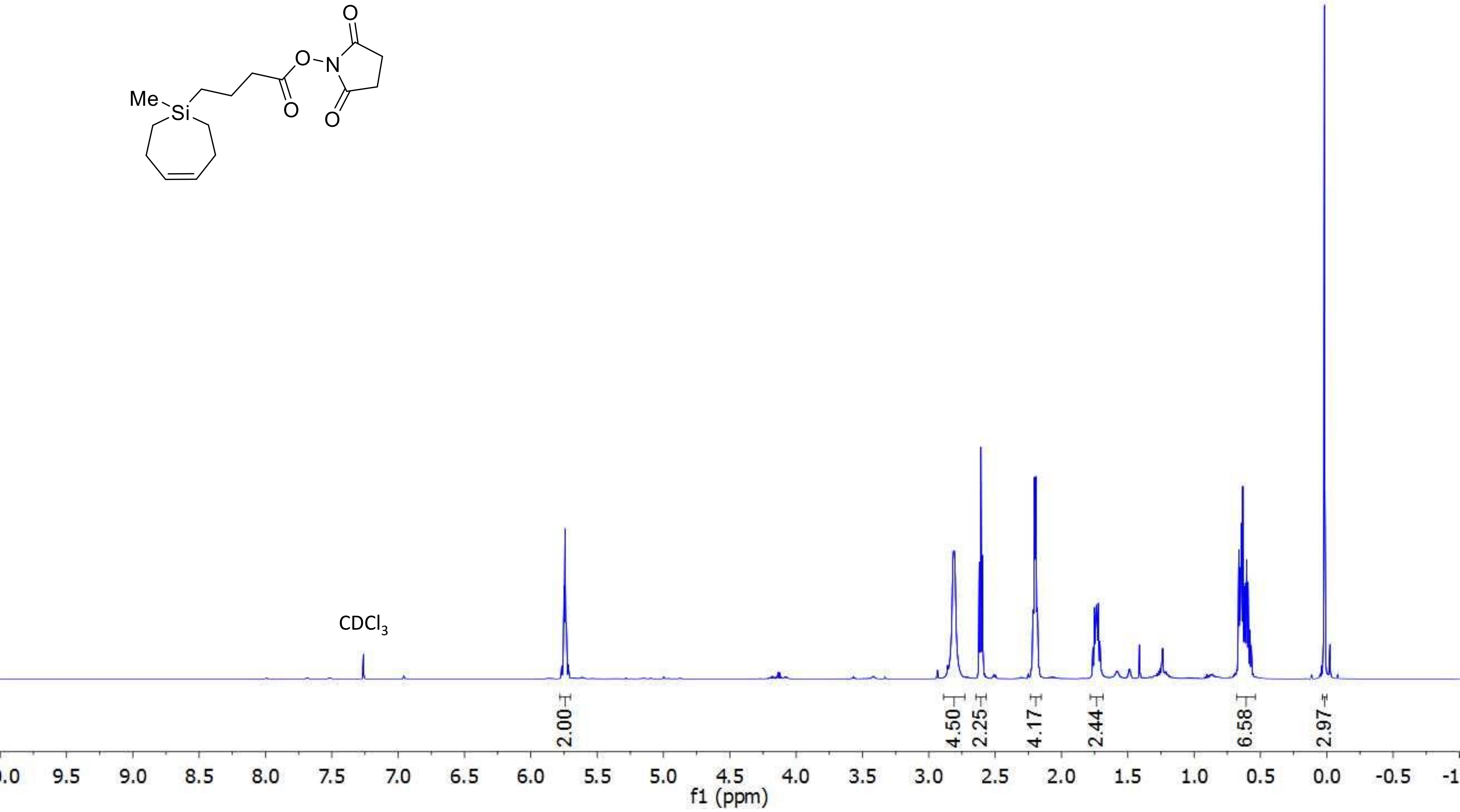

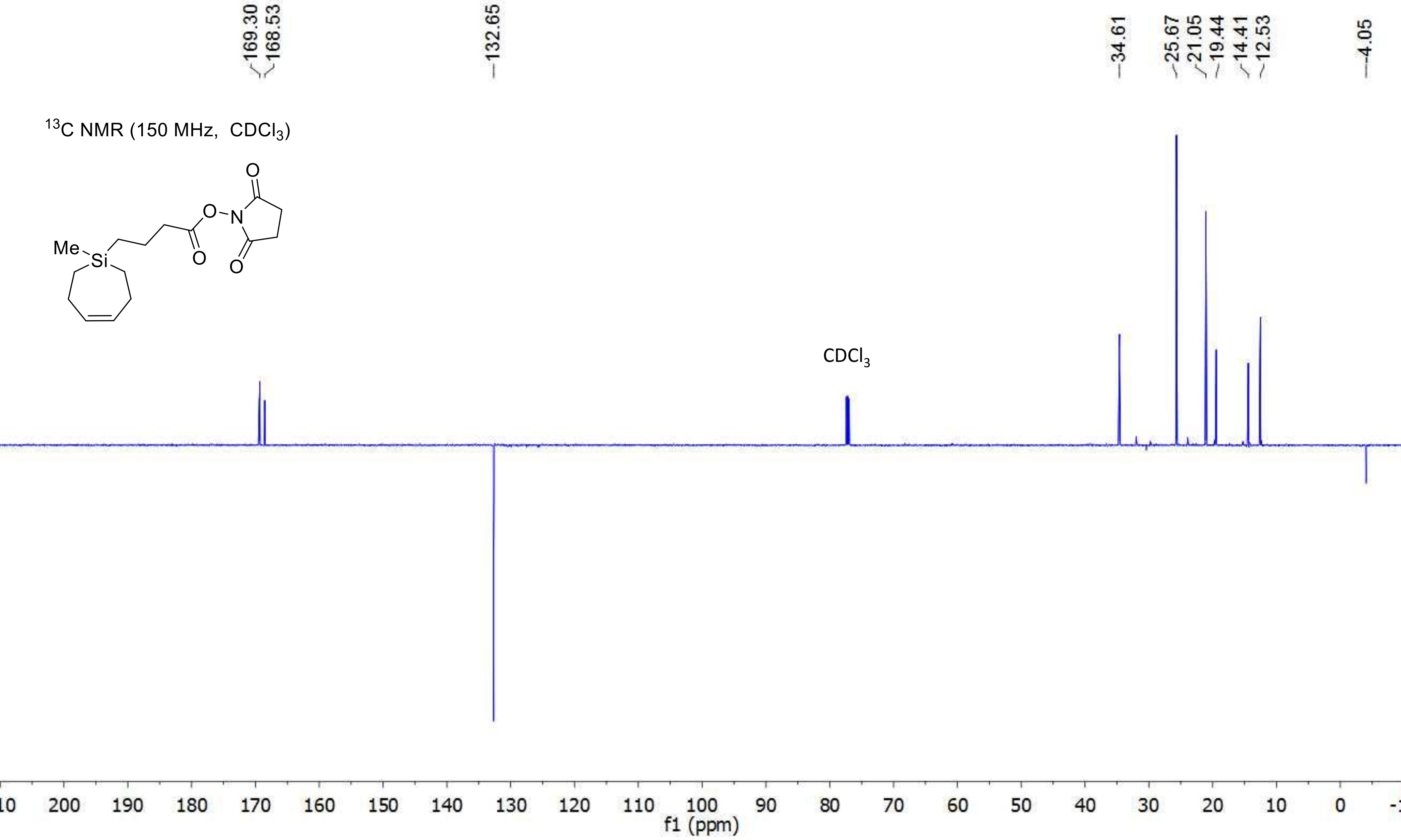

169.30
168.53
132.65
34.61
25.67
21.05
19.44
14.41
12.53
-4.05
13C NMR (150 MHz, CDCl3)
Me
Si
O
N
CDCl3
210 200 190 180 170 160 150 140 130 120 110 100 90 80 70 60 50 40 30 20 10 0
f1 (ppm)

^{1}H NMR (400 MHz, CD_3OD)

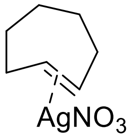

1a

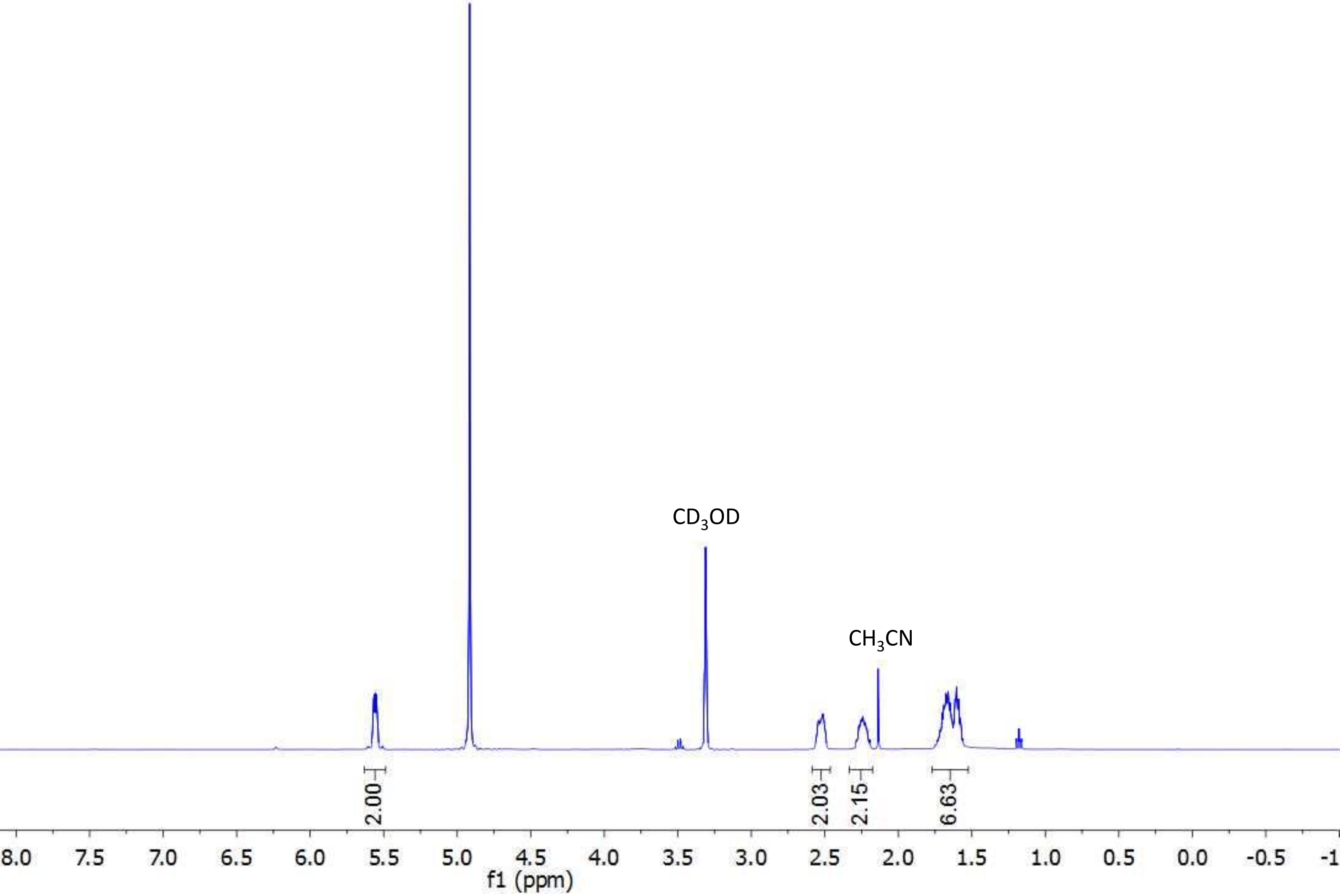

^{13}C NMR (100 MHz, CD_3OD)

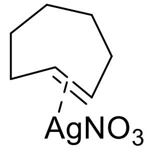

1a

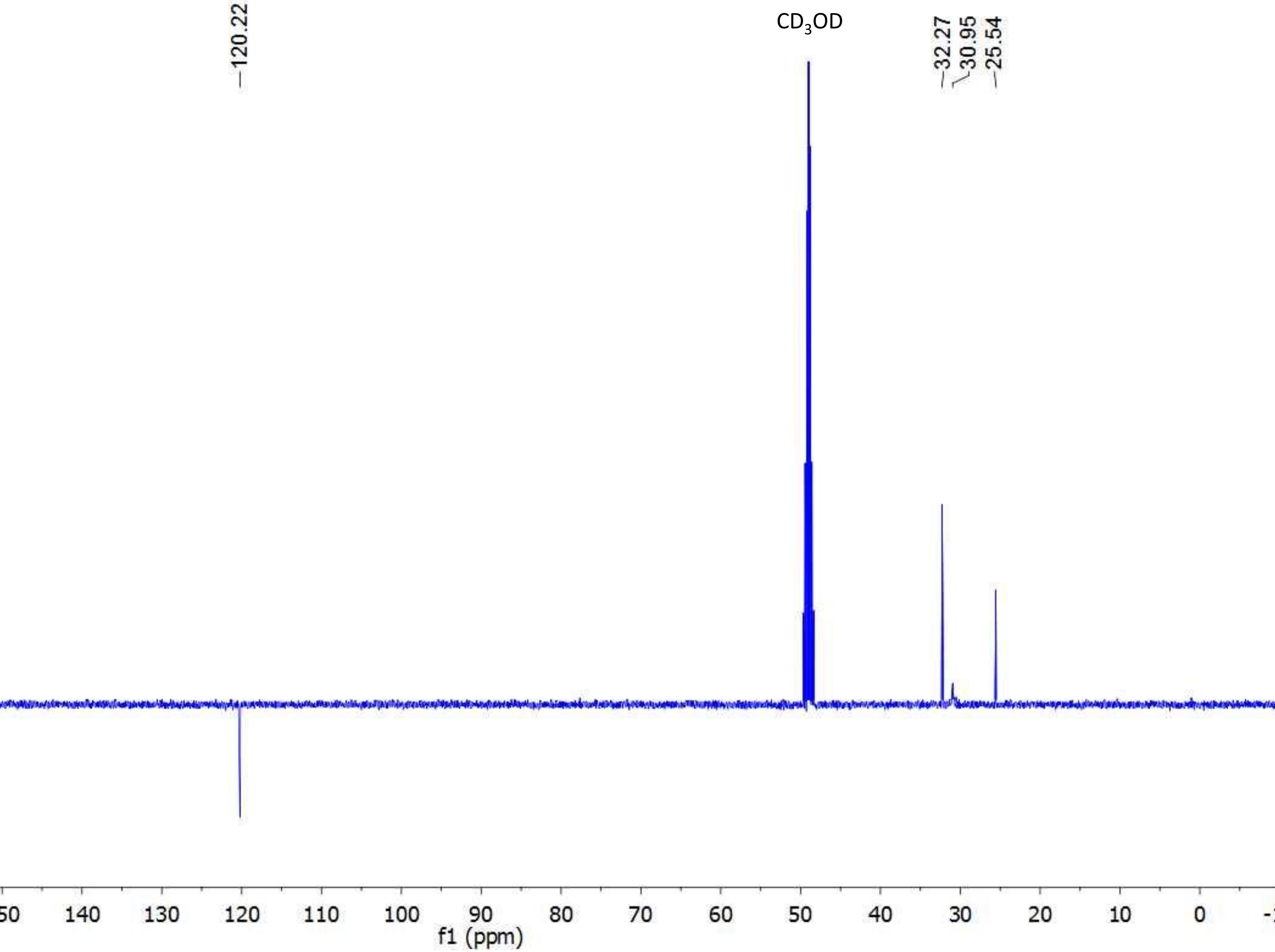

HMQC (400 MHz, CD_3OD)

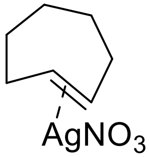

1a

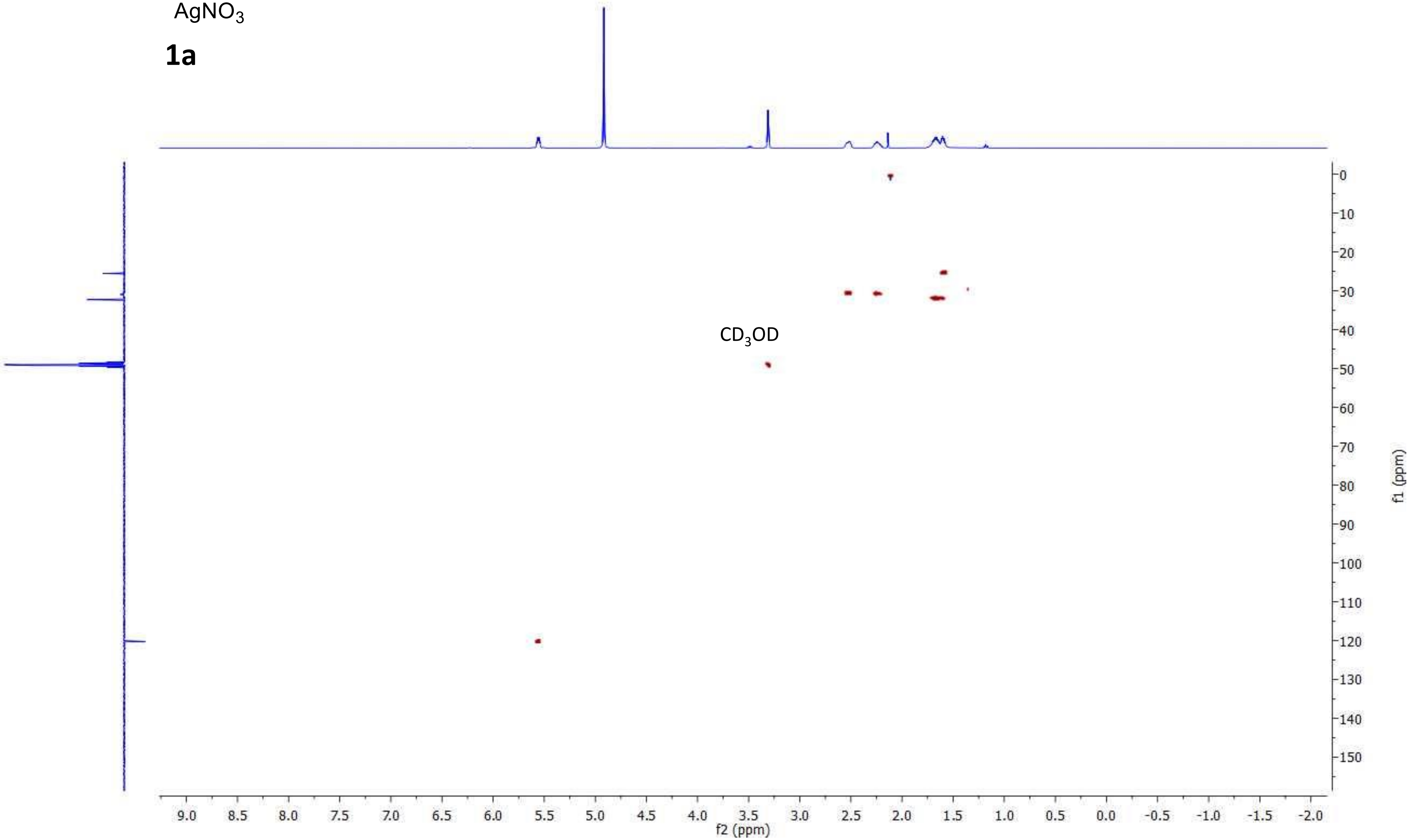

^{1}H NMR (400 MHz, CD_3OD)

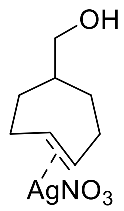

1b

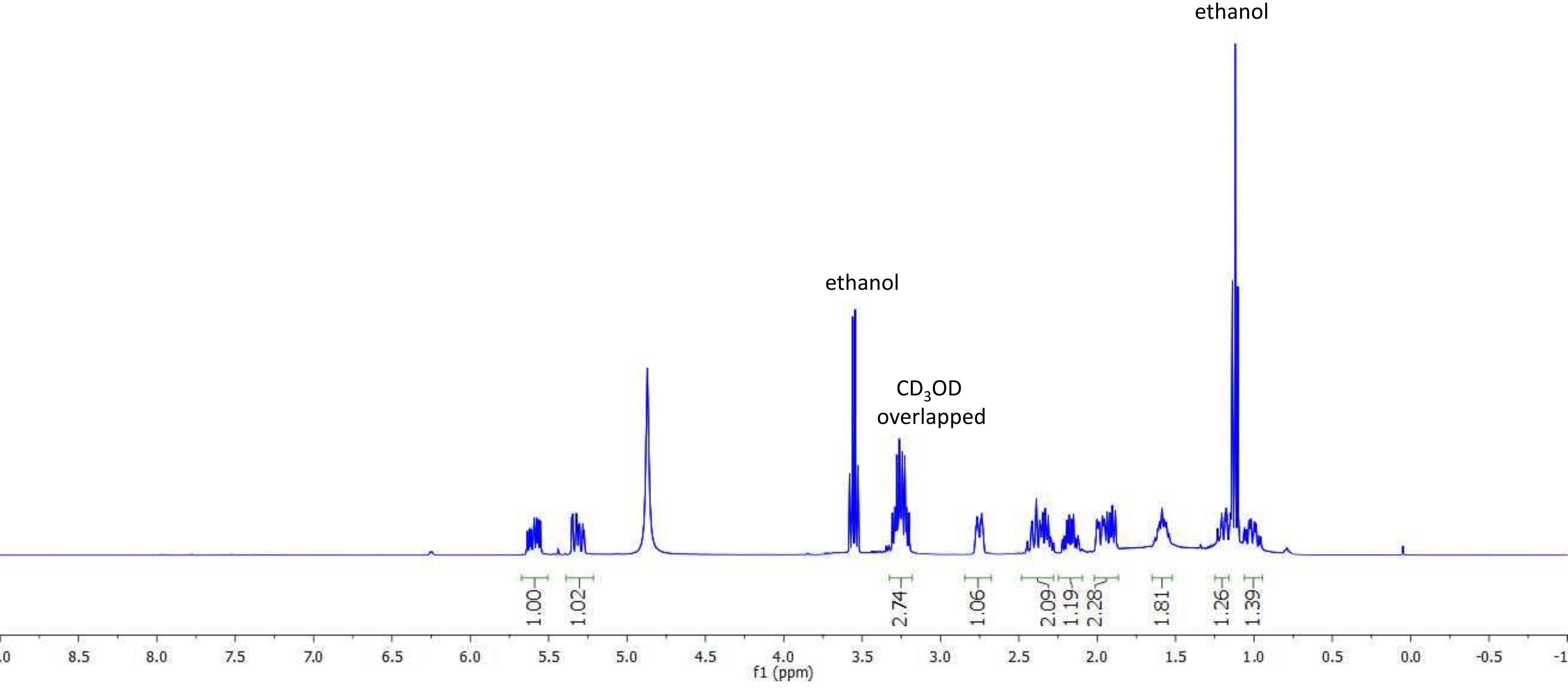

^{13}C NMR (100 MHz, CD_3OD)

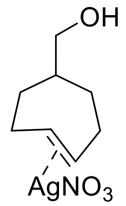

1b

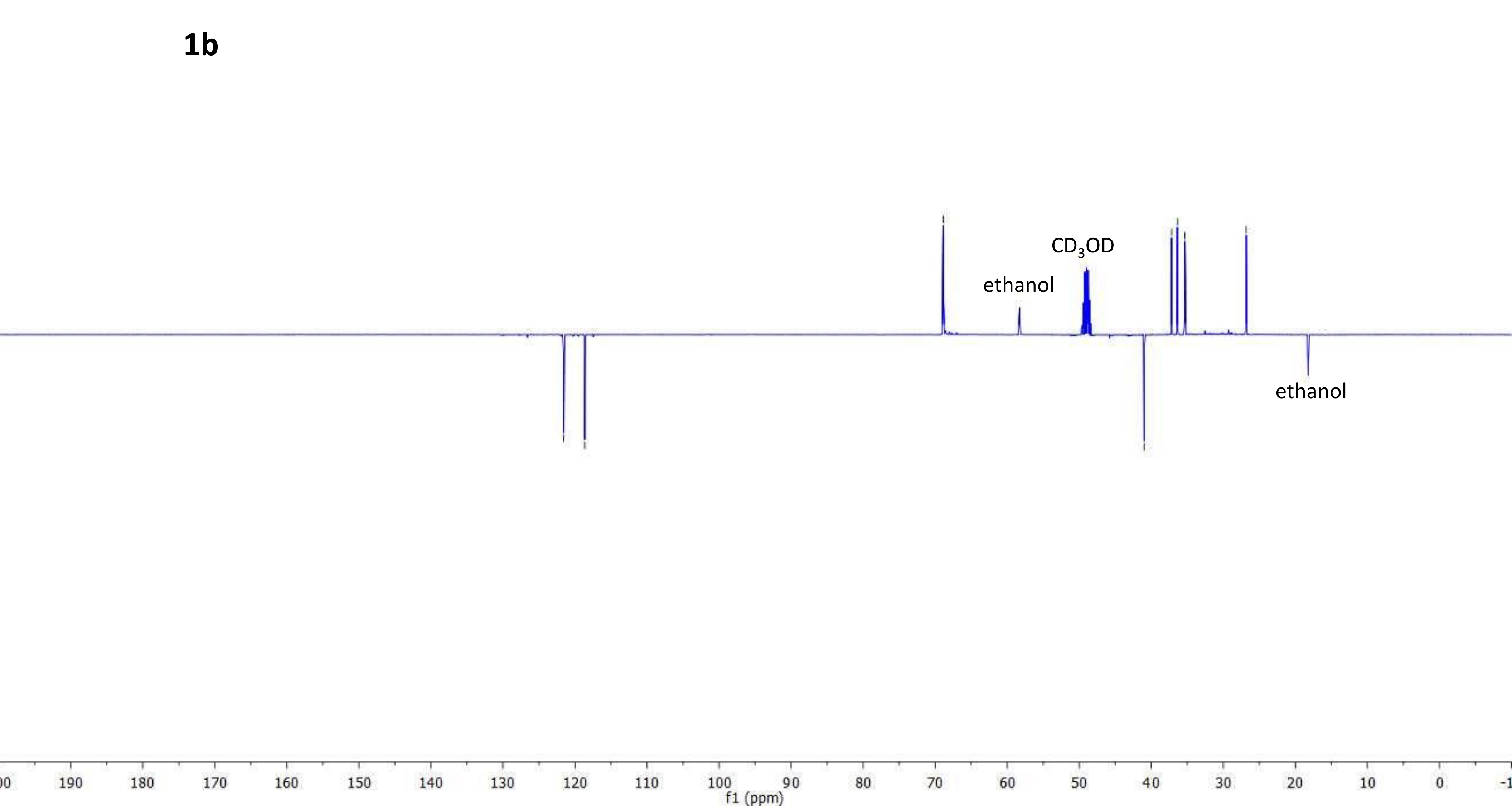

^{1}H NMR (400 MHz, CD_3OD)

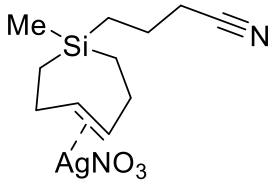

2b

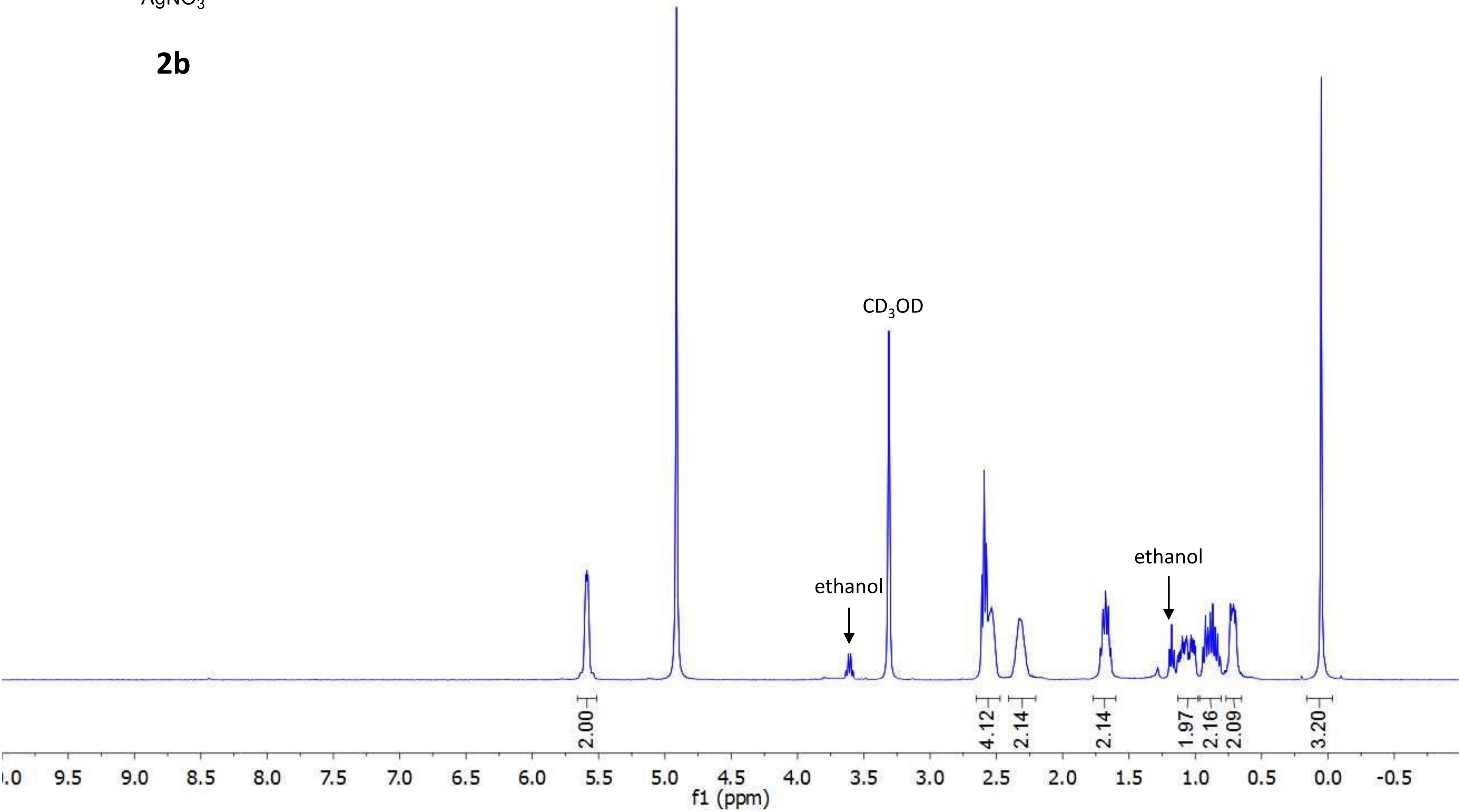

^{13}C NMR (100 MHz, CD_3OD)

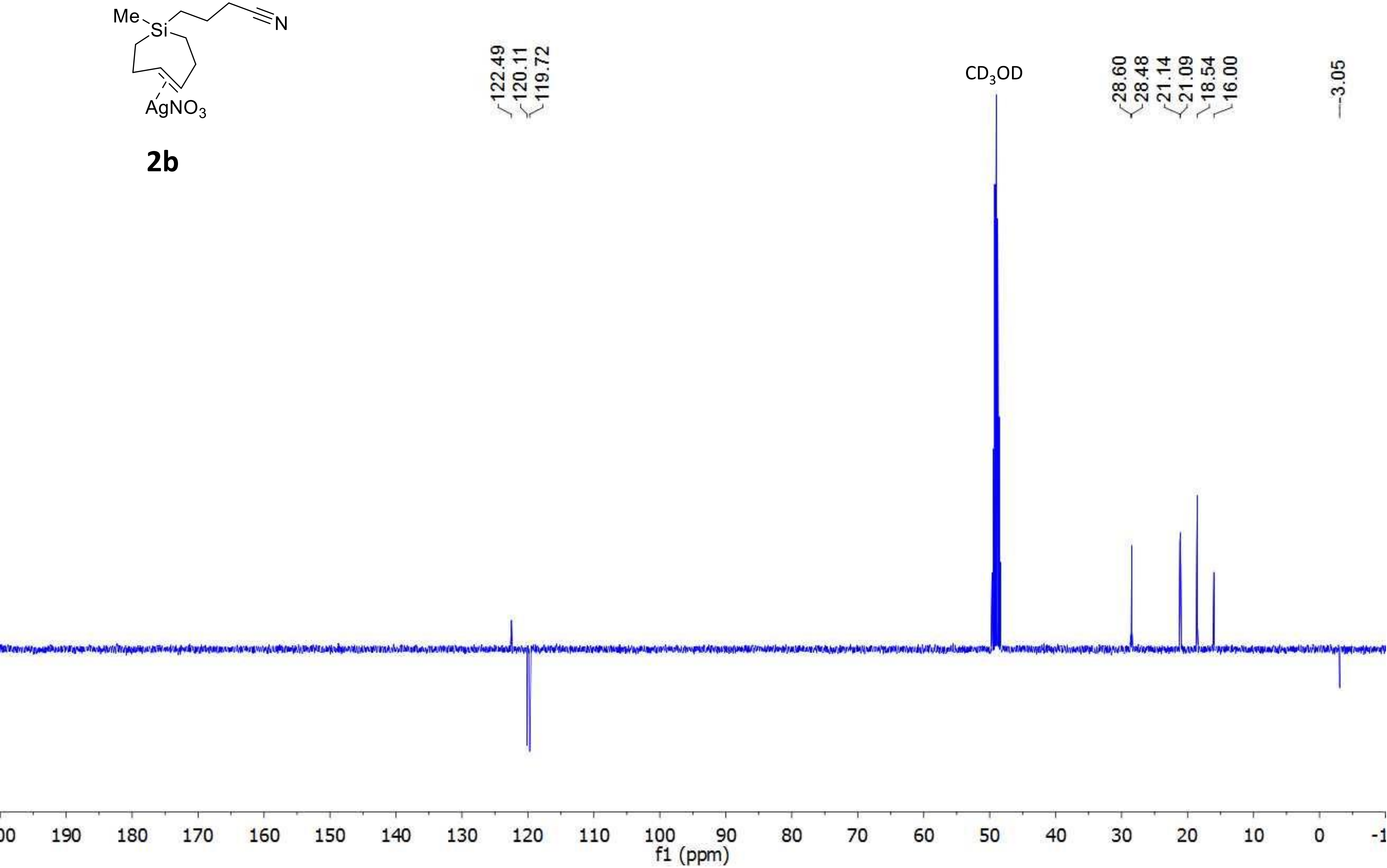

2b

HMQC (400 MHz, CD_3OD)

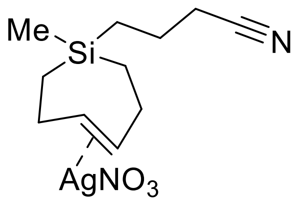

2b

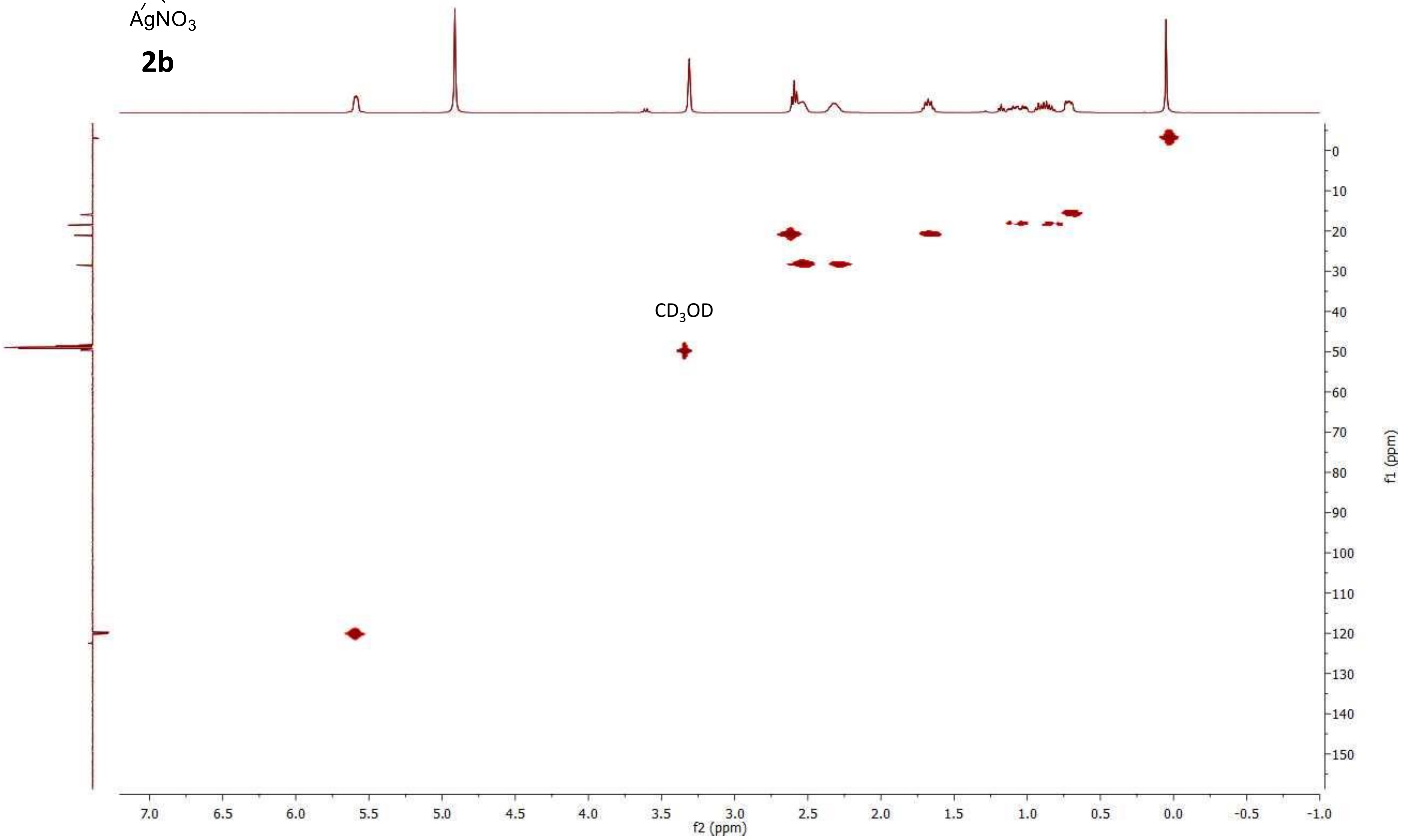

^{1}H NMR (400 MHz, C_6D_6)

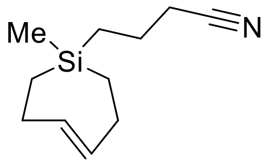

7b (98% trans)

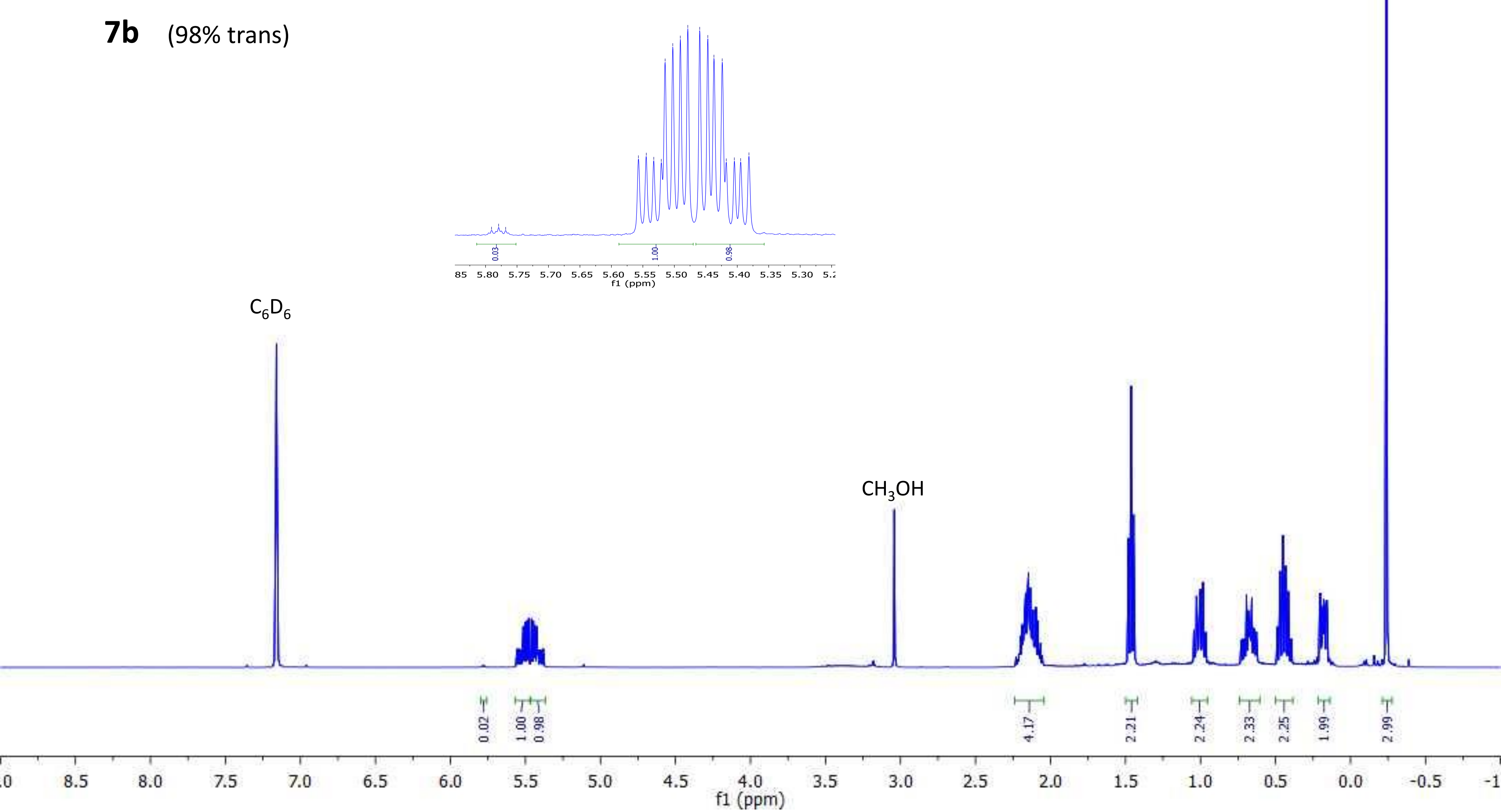

^{13}C NMR (100 MHz, C_6D_6)

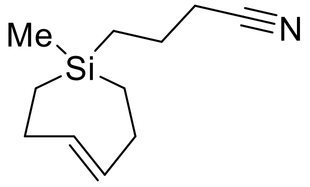

7b (98% trans)

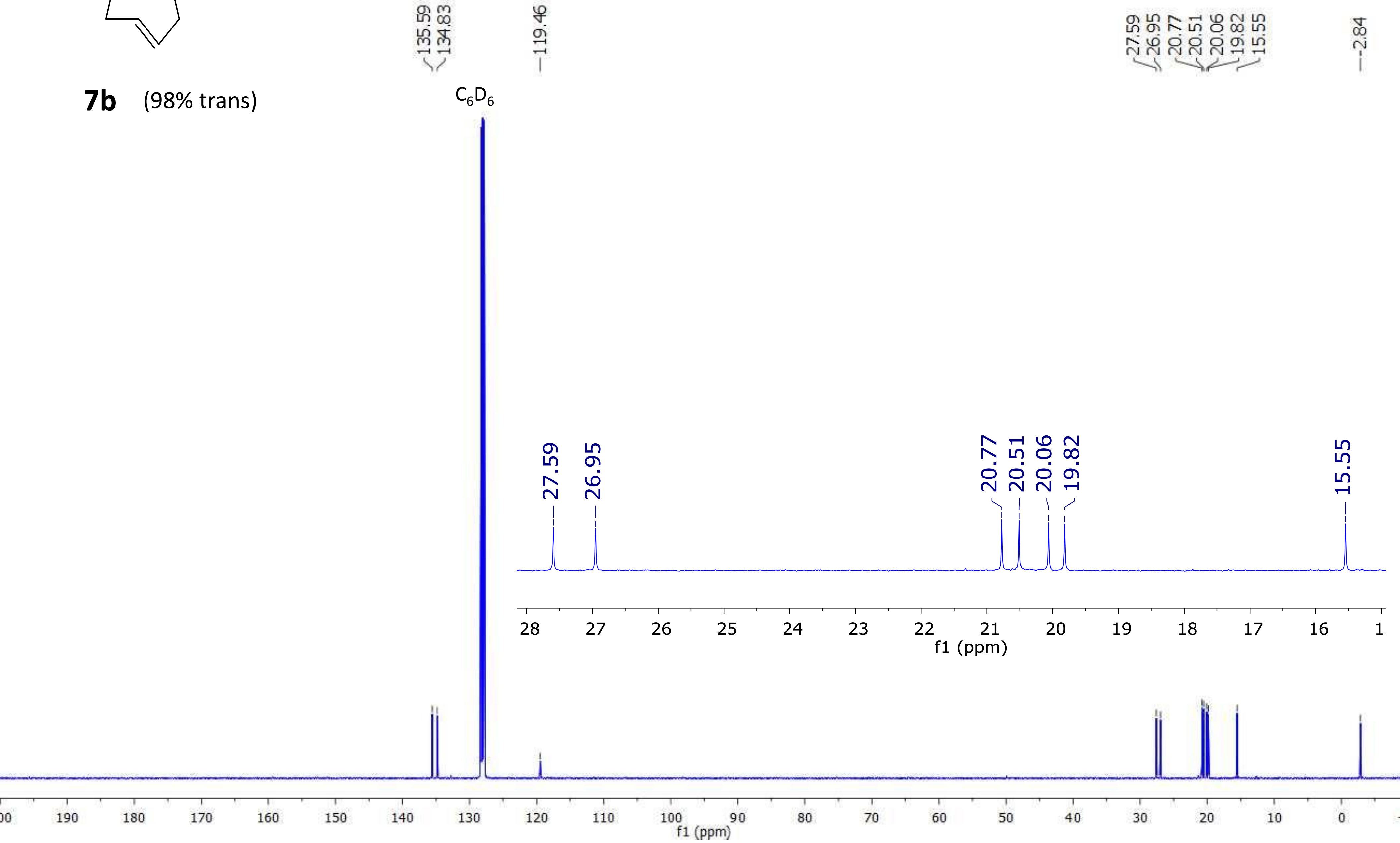

^{1}H NMR (400 MHz, CD_3OD)

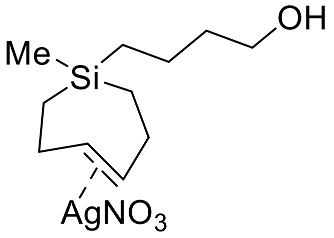

2c

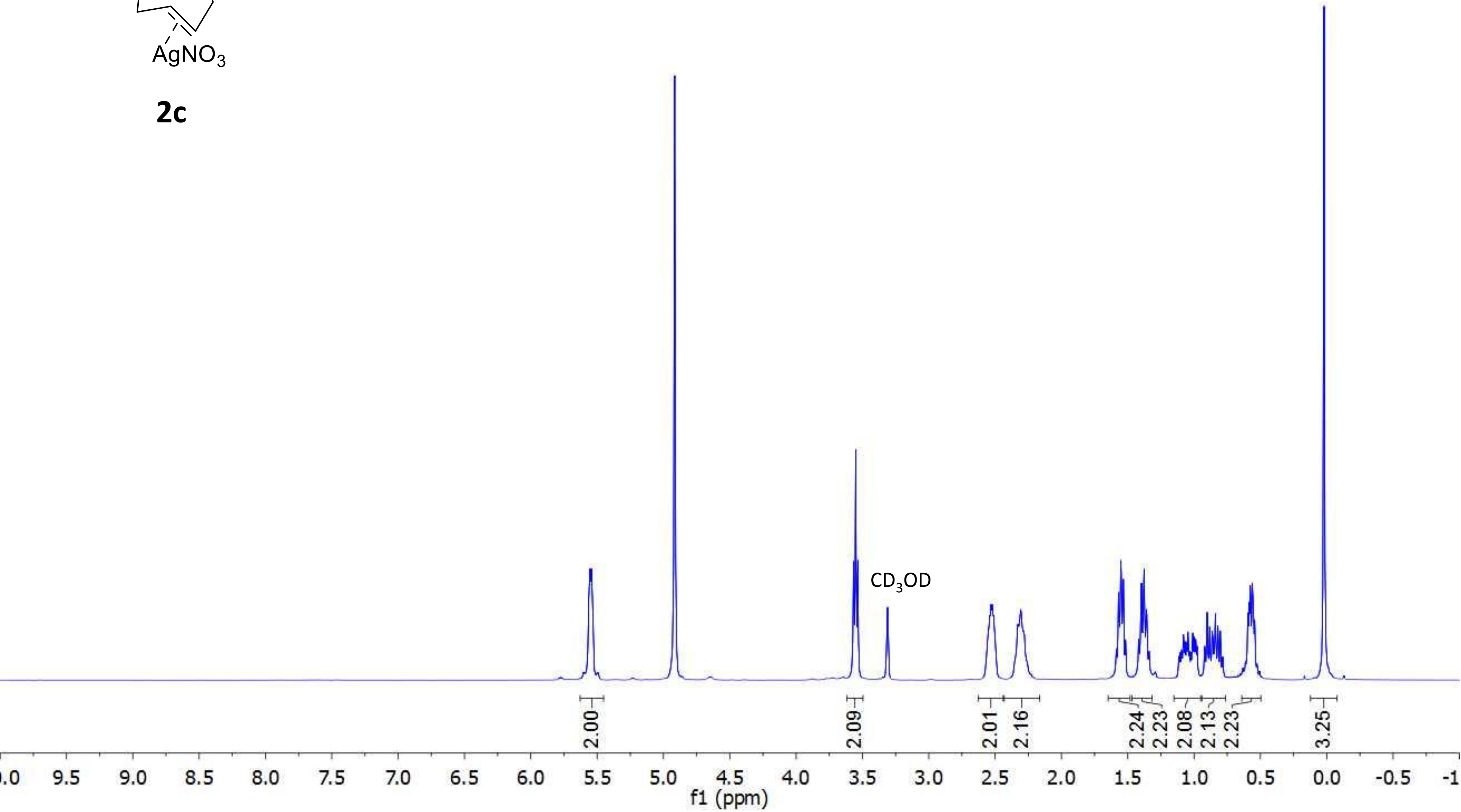

^{13}C NMR (100 MHz, CD_3OD)

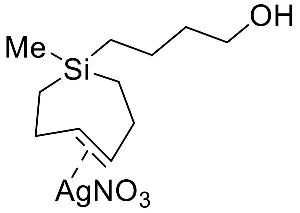

2c

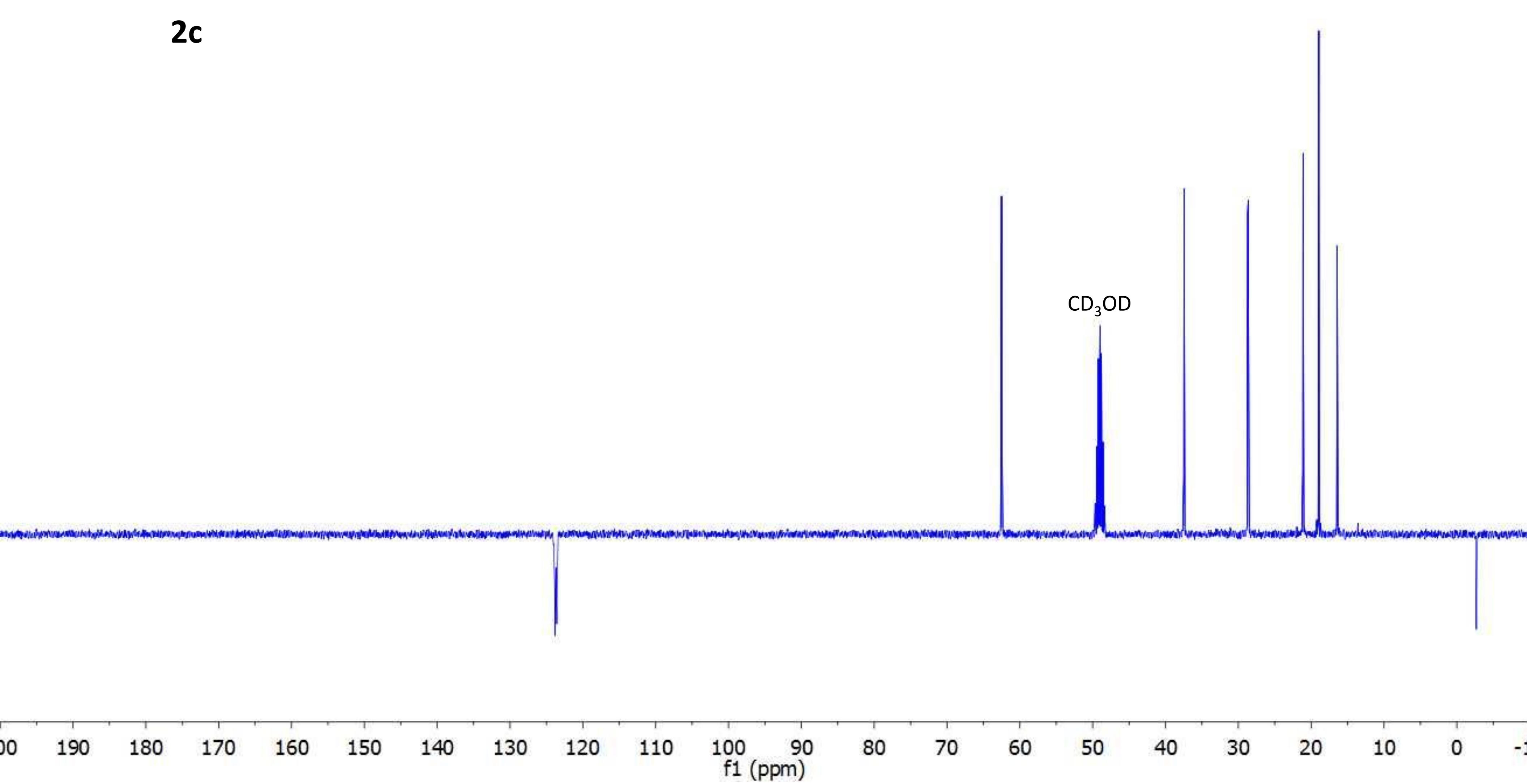

HMQC (400 MHz, CD_3OD)

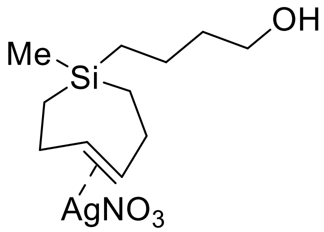

2c

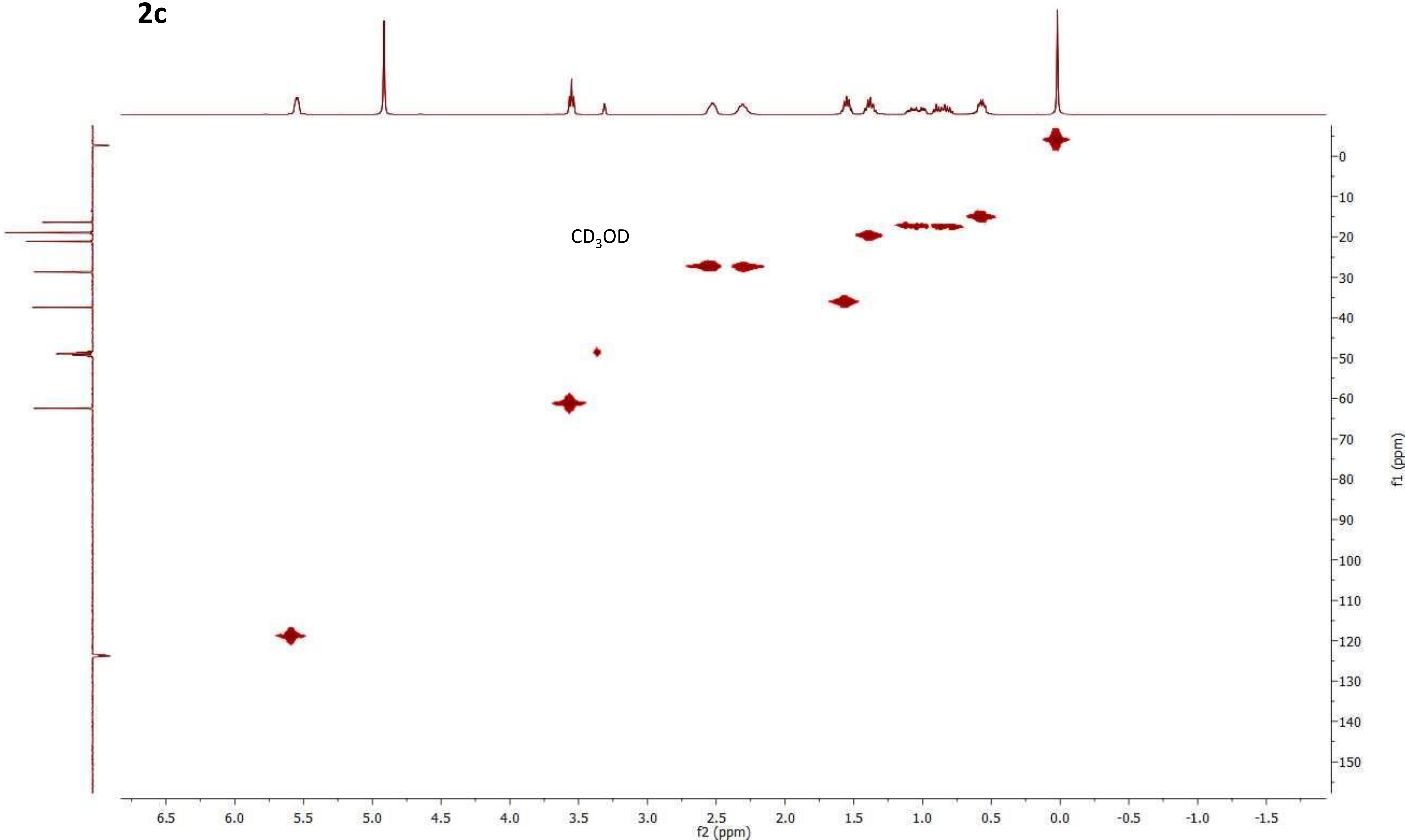

^{1}H NMR (400 MHz, C_6D_6)

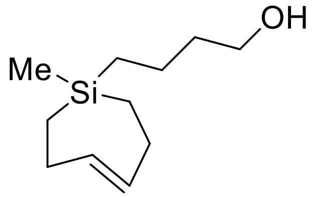

7c (98% trans)

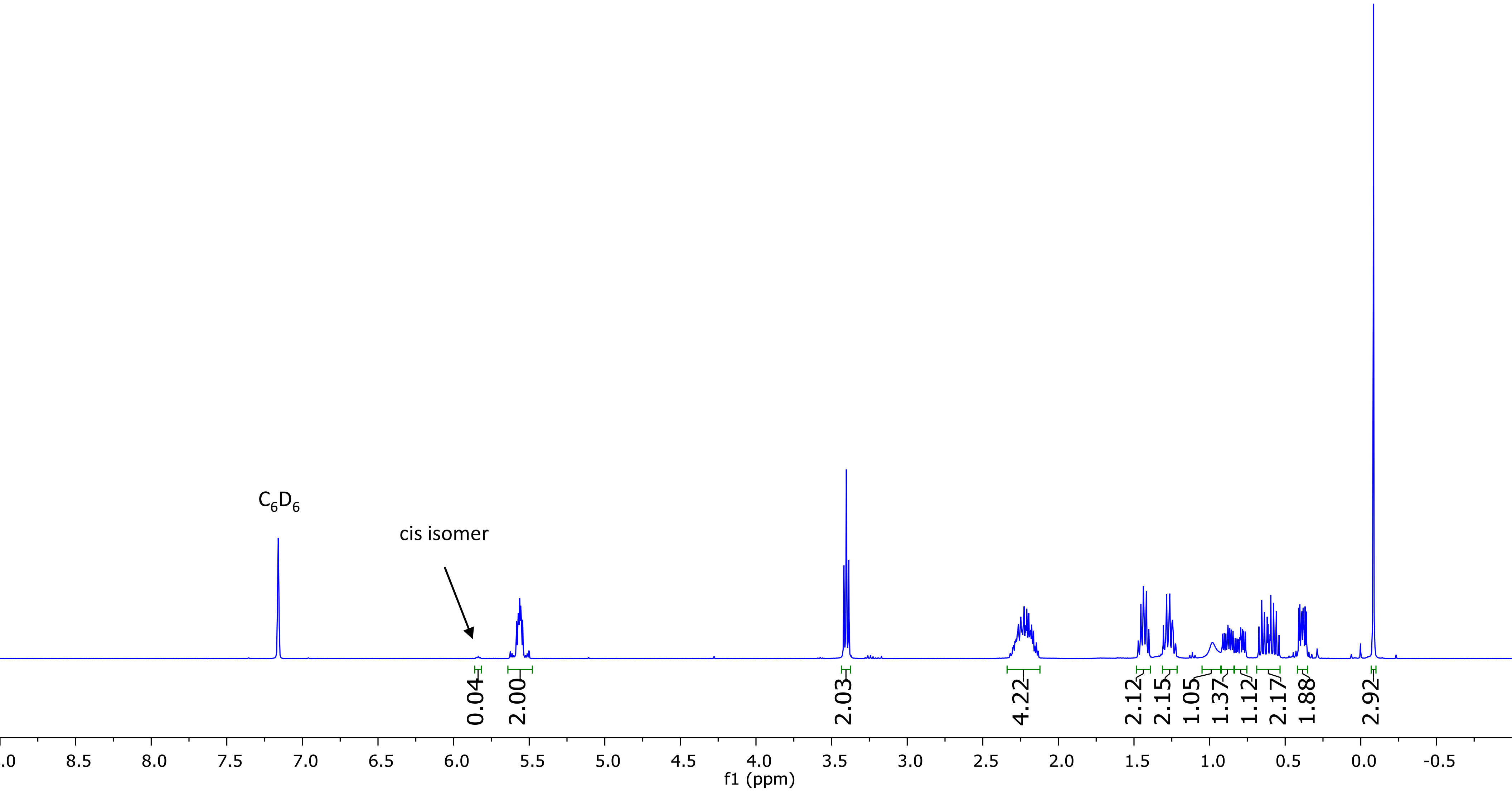

^{13}C NMR (100 MHz, C_6D_6)

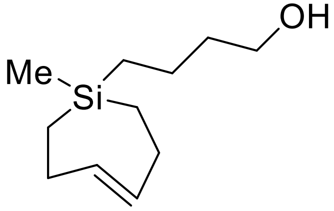

7c (98% trans)

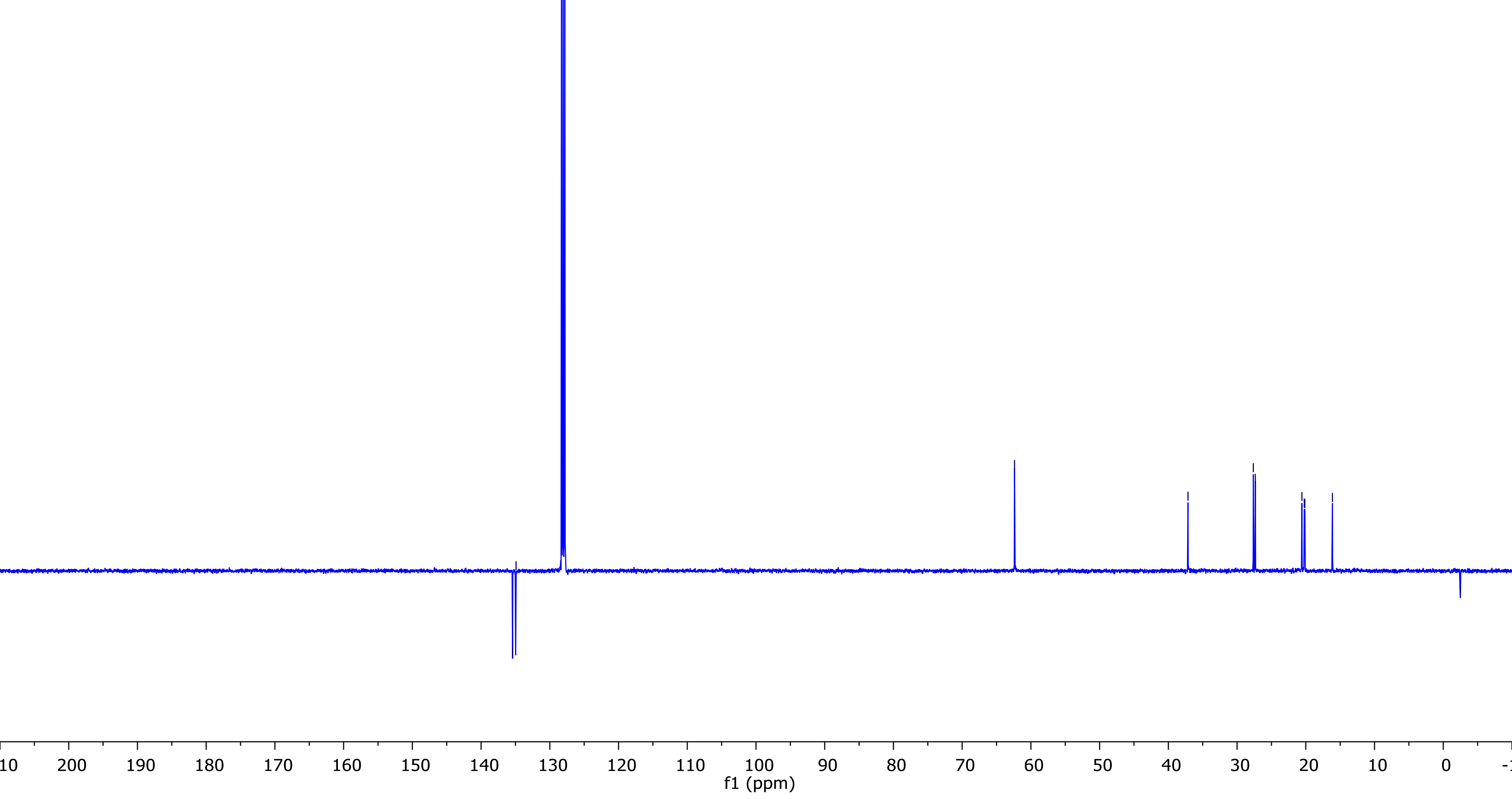

^{1}H NMR (400 MHz, CD_3OD)

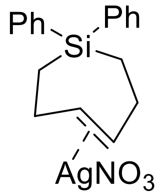

2a

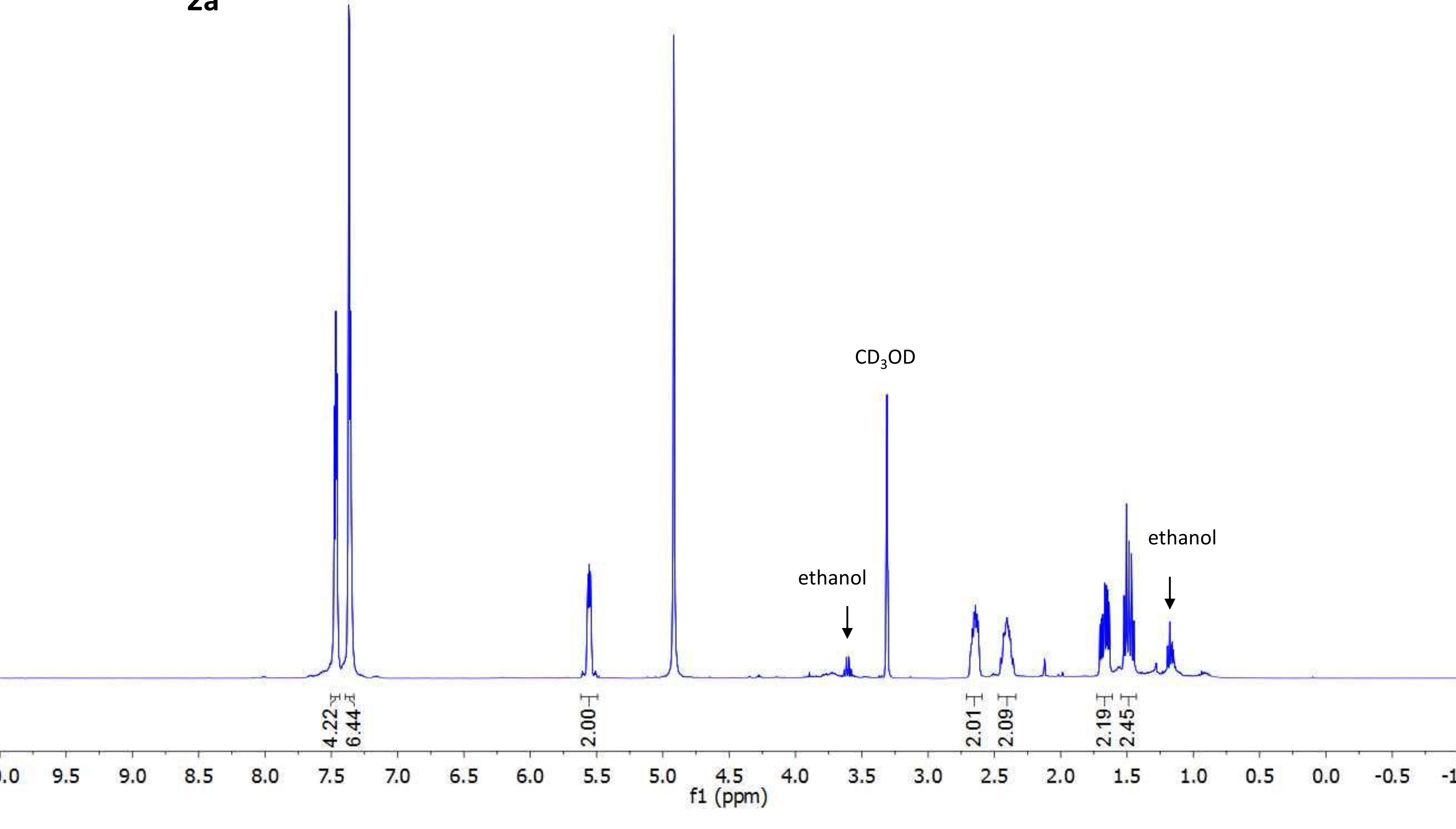

^{13}C NMR (100 MHz, CD_3OD)

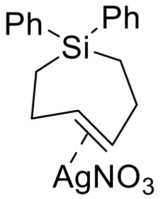

2a

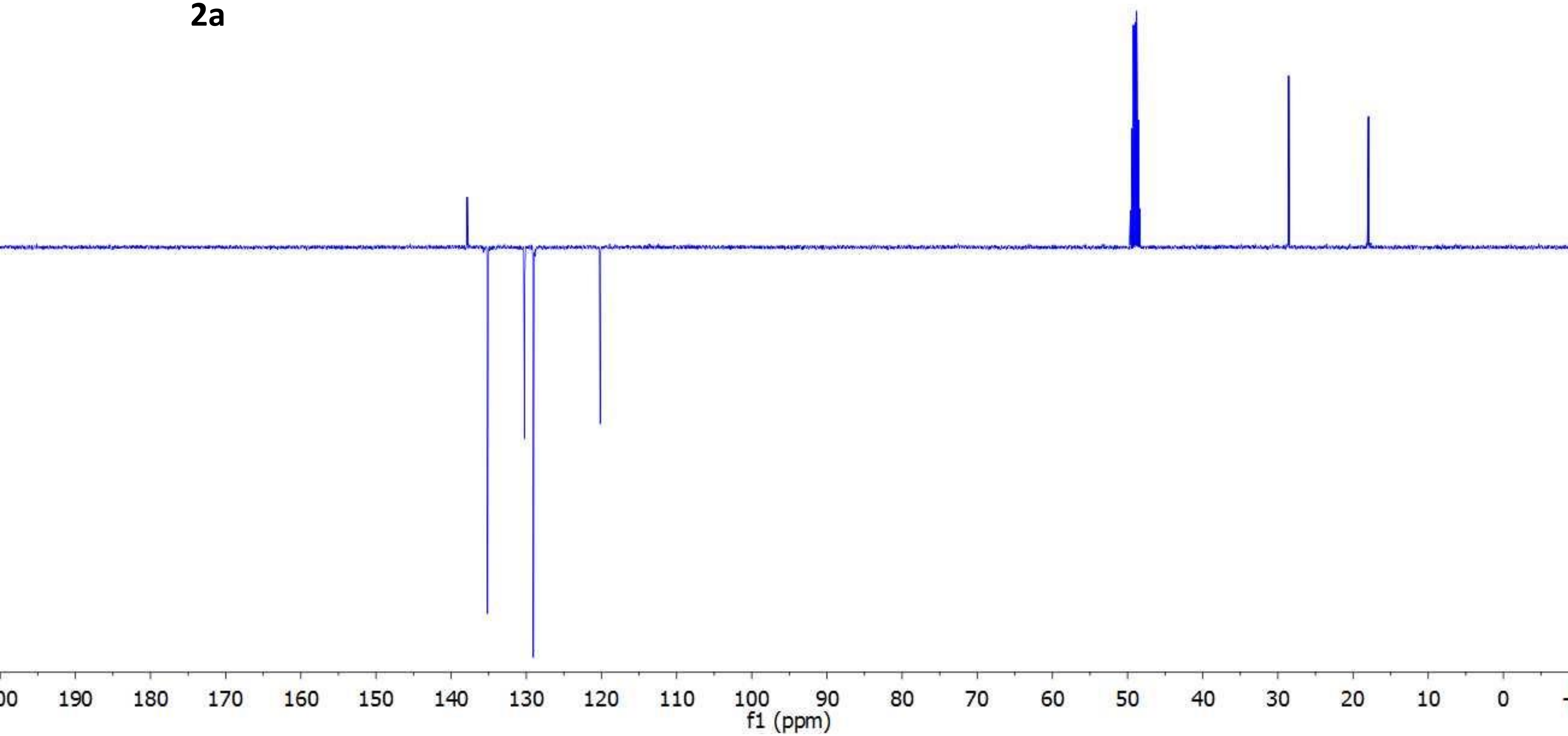

^{1}H NMR (600 MHz, CD_3OD)

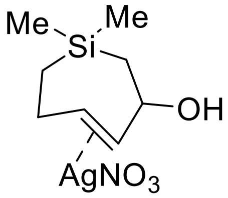

1.1: 1 dr

2e

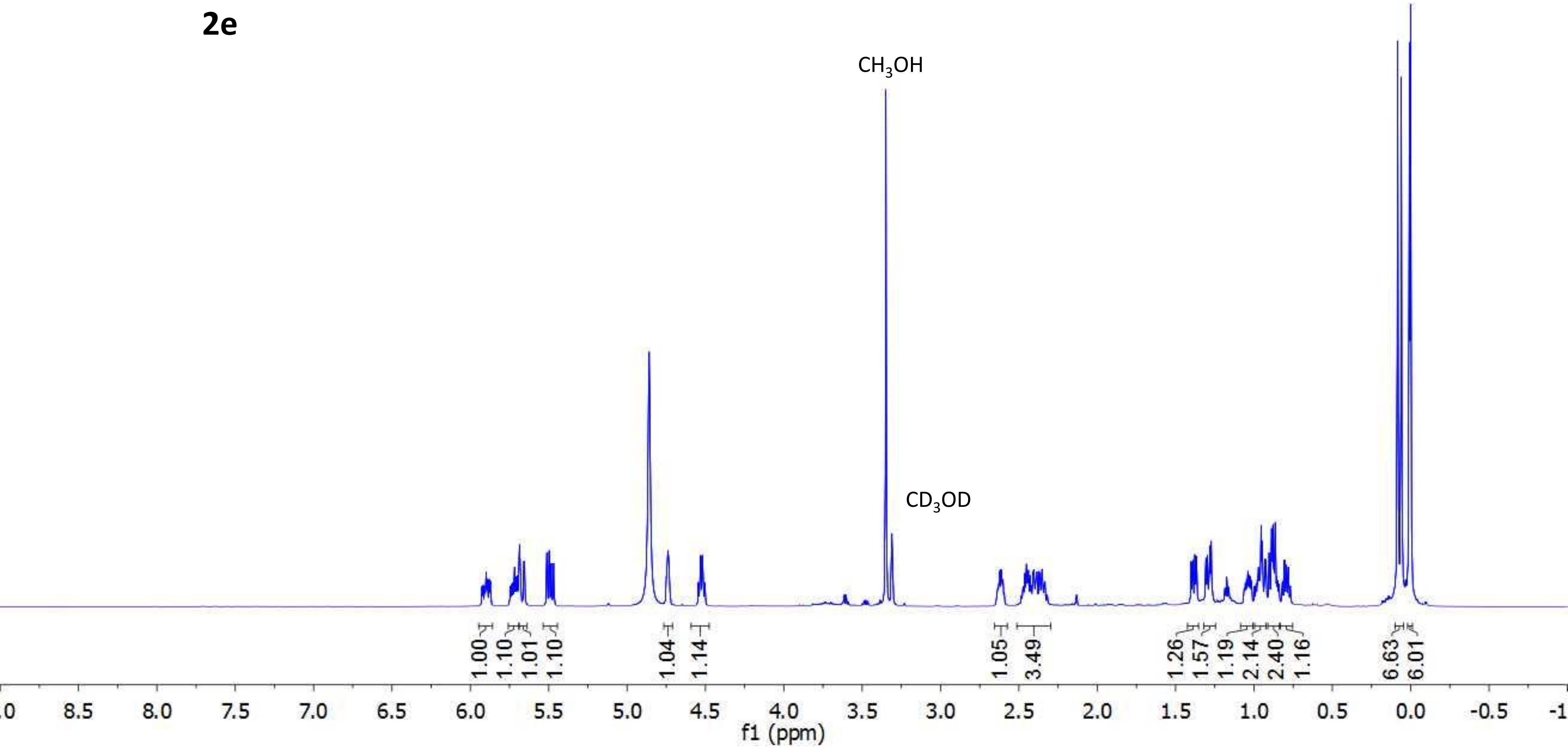

^{13}C NMR (150 MHz, CD_3OD)

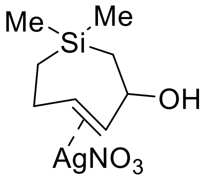

1.1: 1 dr

2e

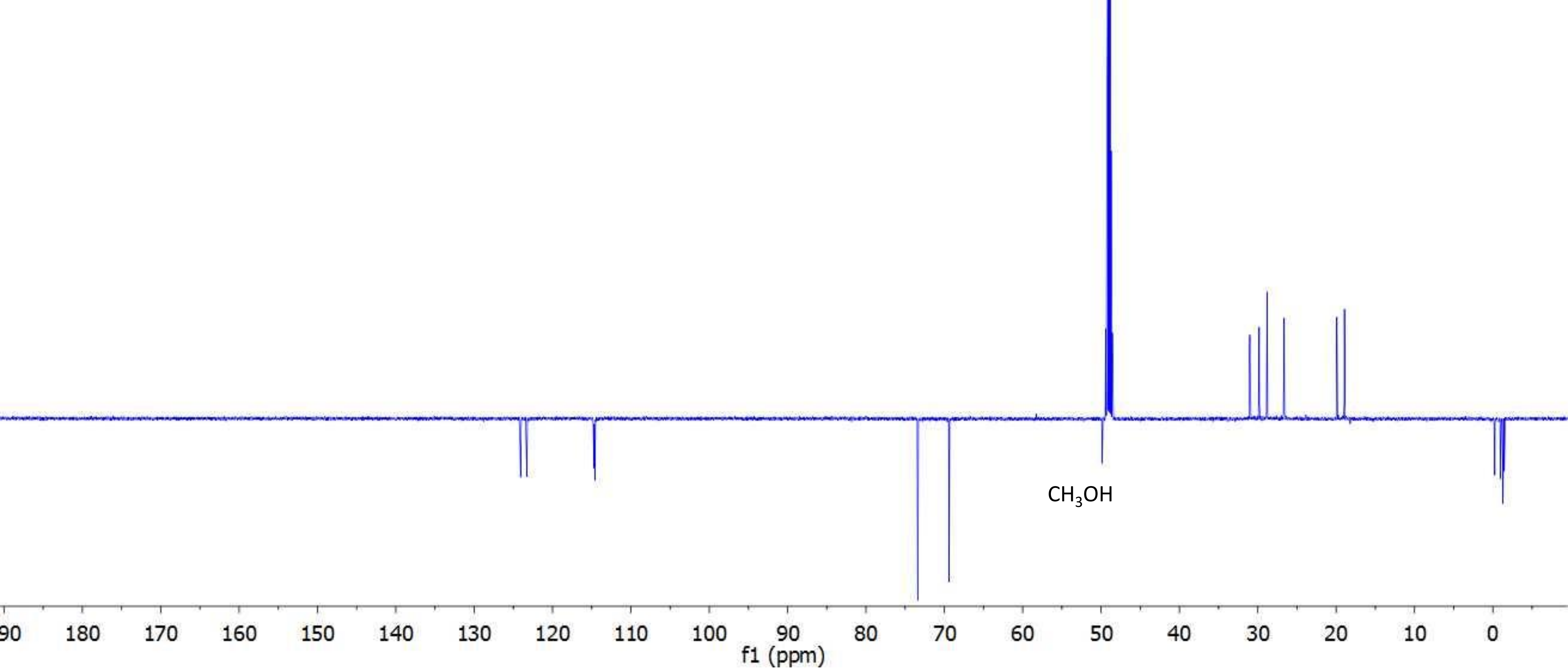

^{1}H NMR (600 MHz, CD_3OD)

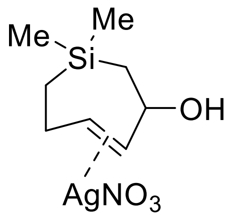

Major diastereomer

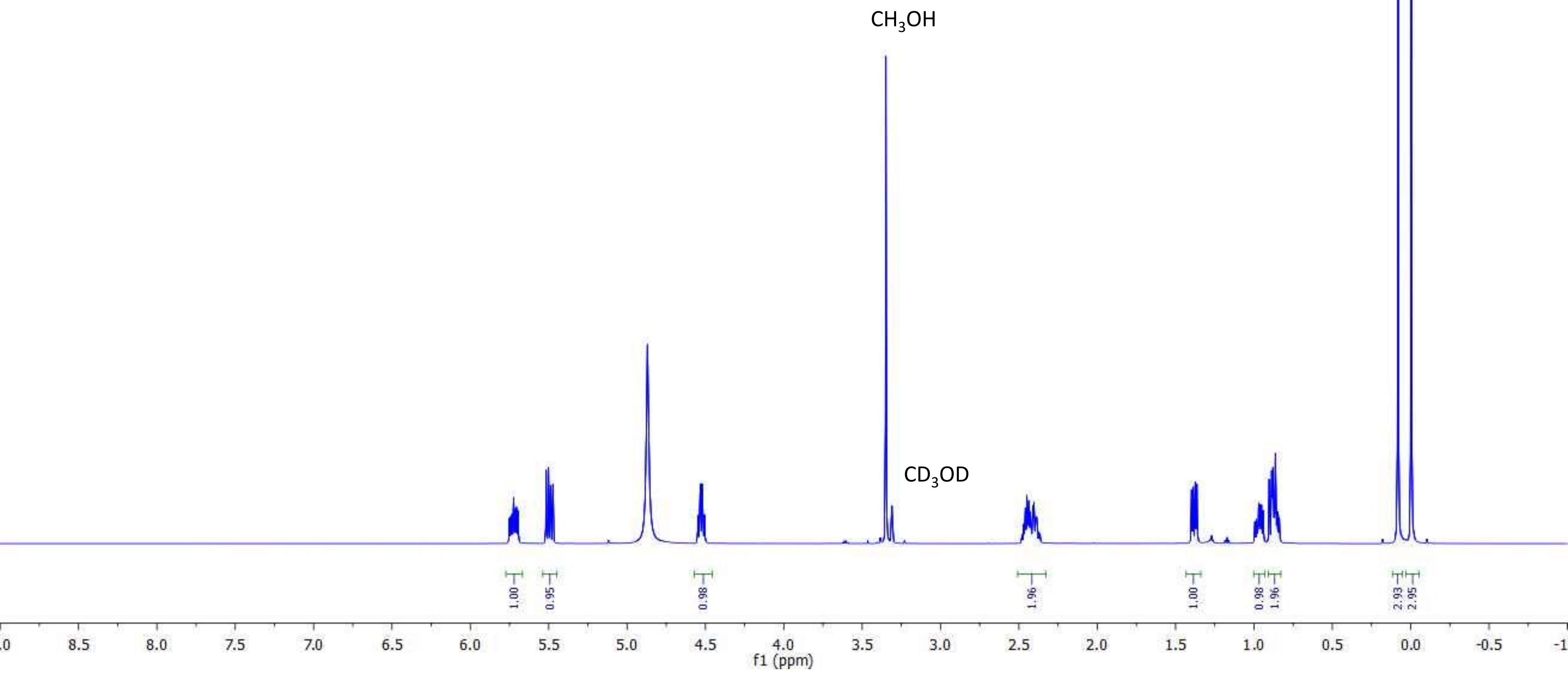

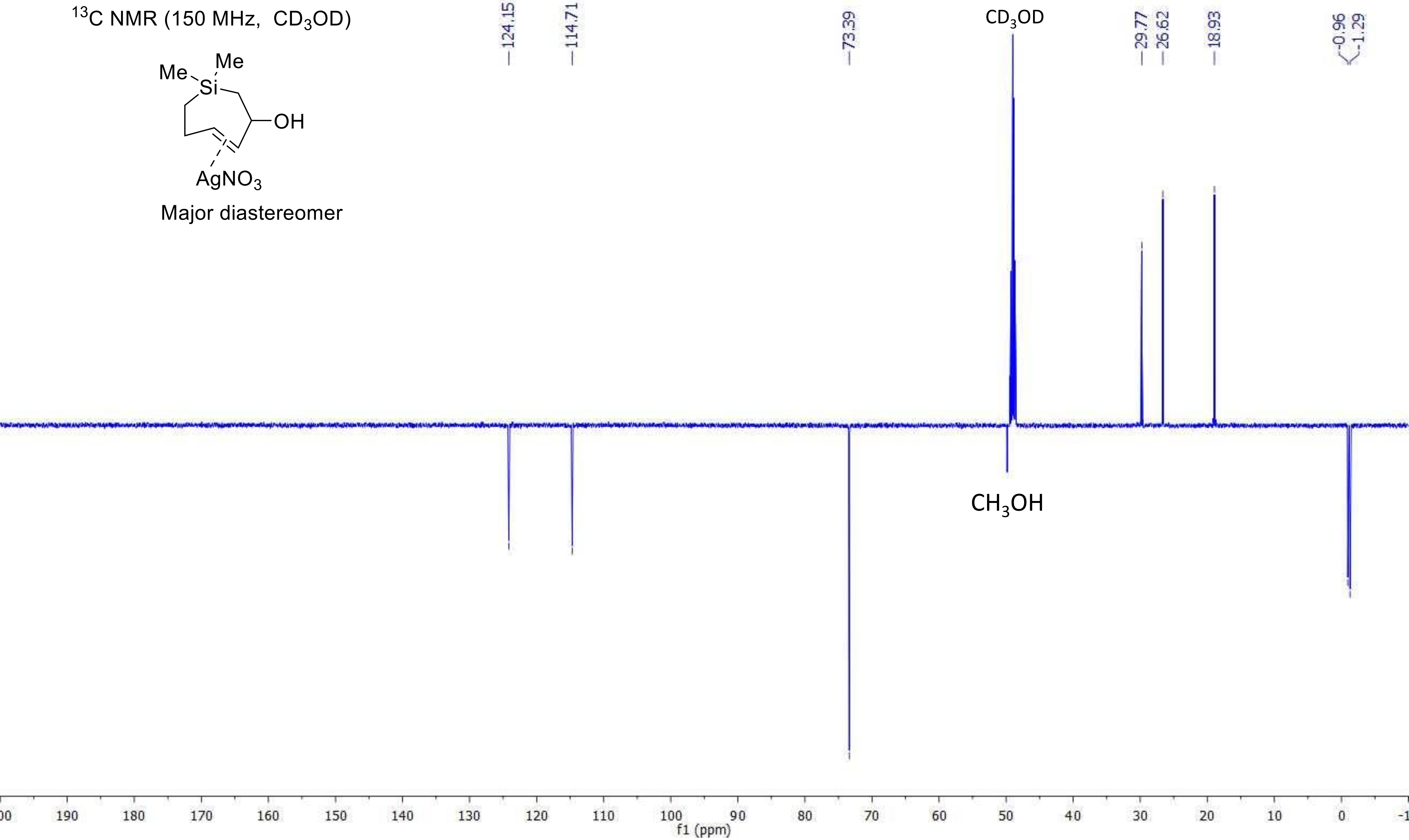

^{13}C NMR (150 MHz, CD$_3$OD)
Me
Me
Si
OH
AgNO$_3$
Major diastereomer
124.15
114.71
73.39
CD$_3$OD
29.77
26.62
18.93
-0.96
-1.29
CH$_3$OH
f1 (ppm)

^{1}H NMR (600 MHz, CD_3OD)

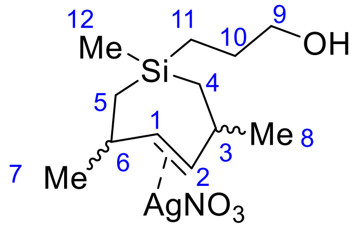

2d

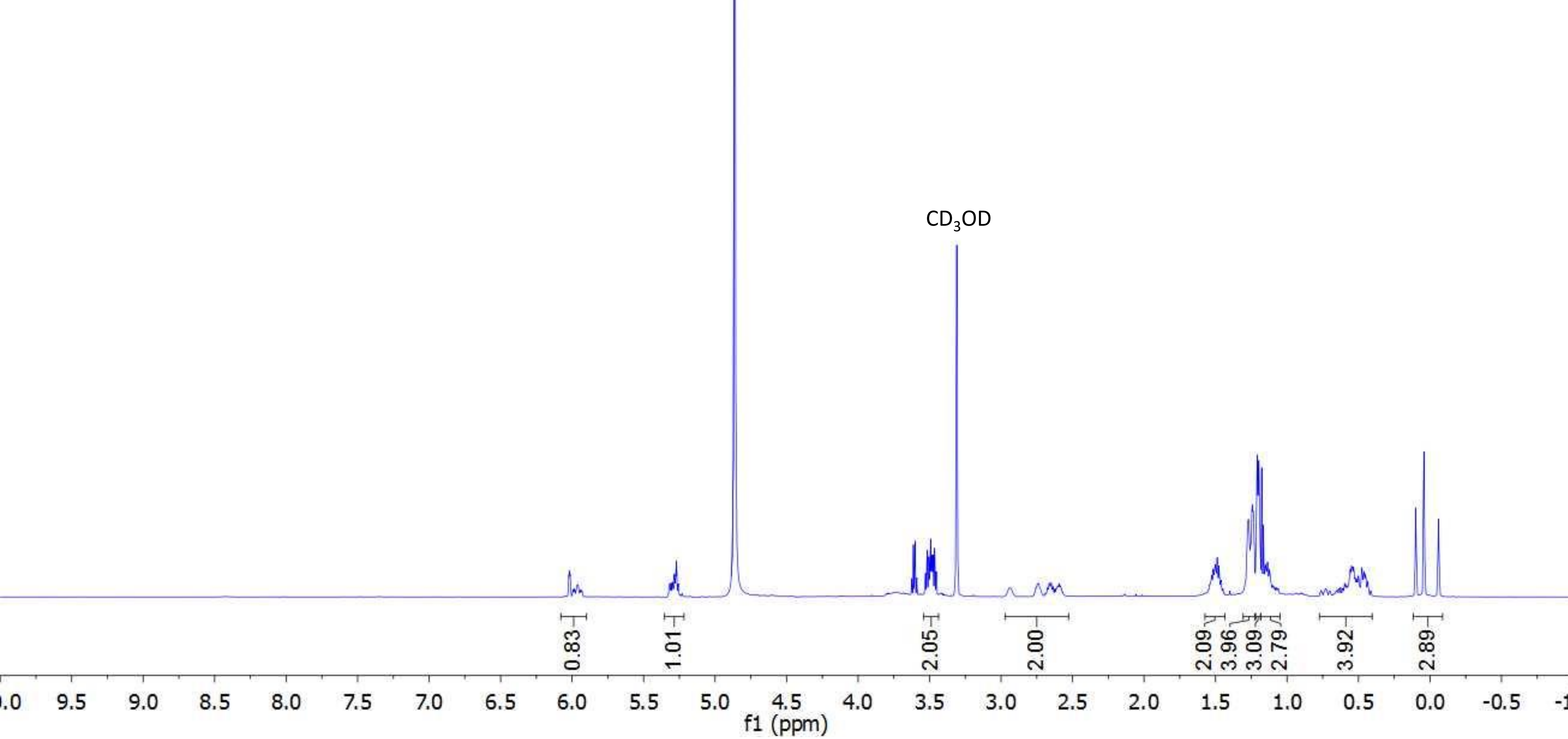

^{13}C NMR (100 MHz, CD_3OD)

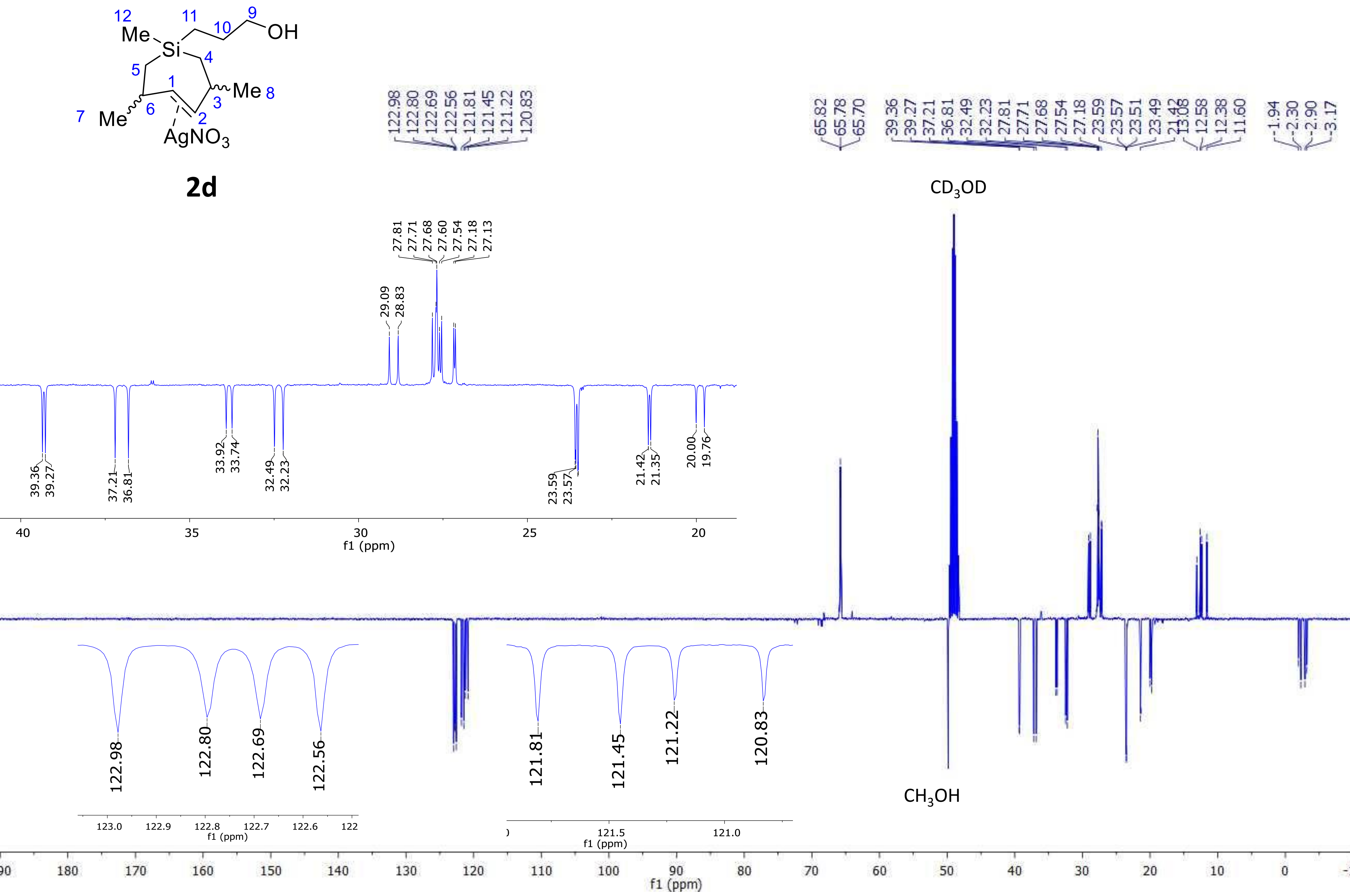

^1H NMR (600 MHz, C_6D_6)

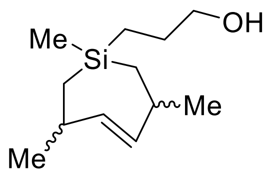

7d

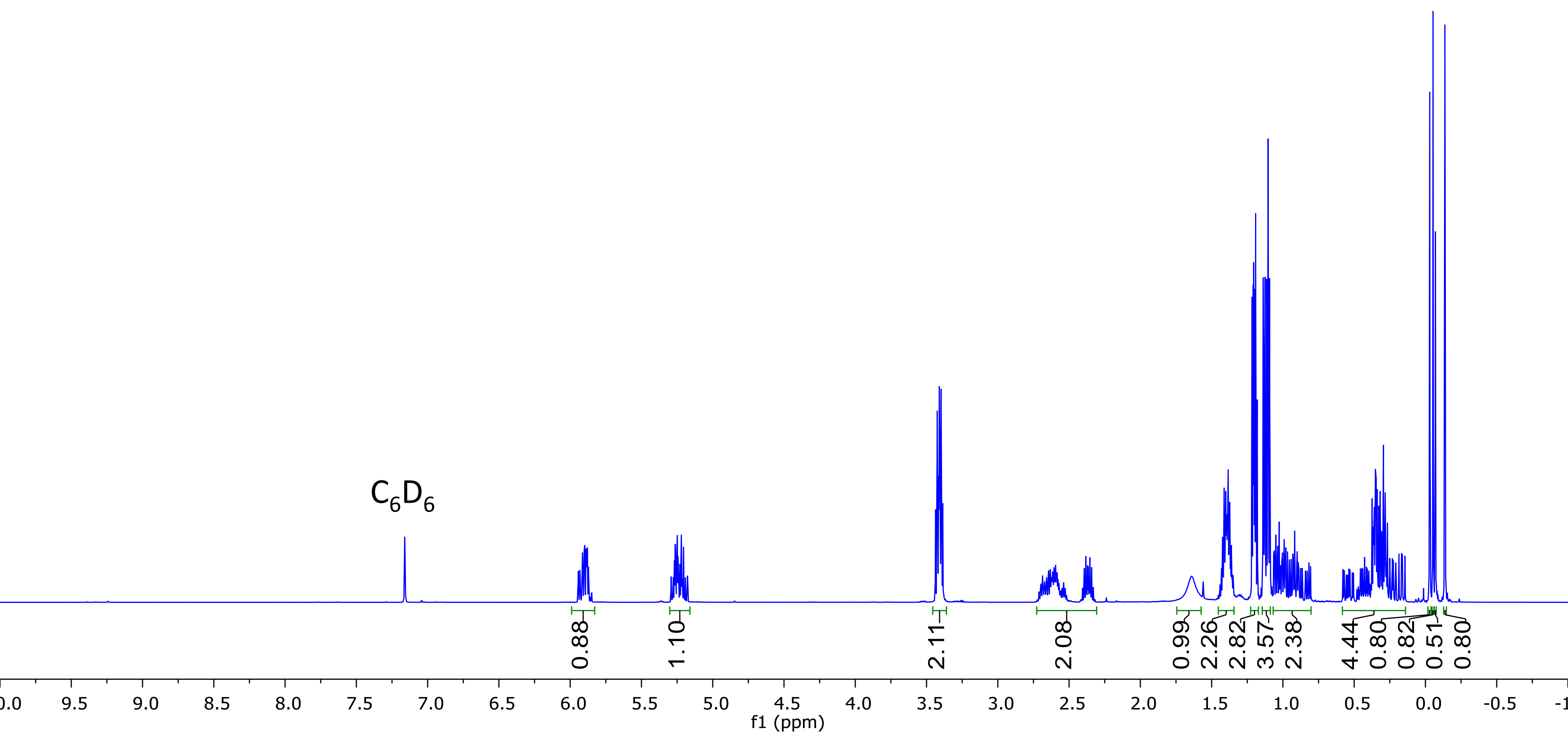

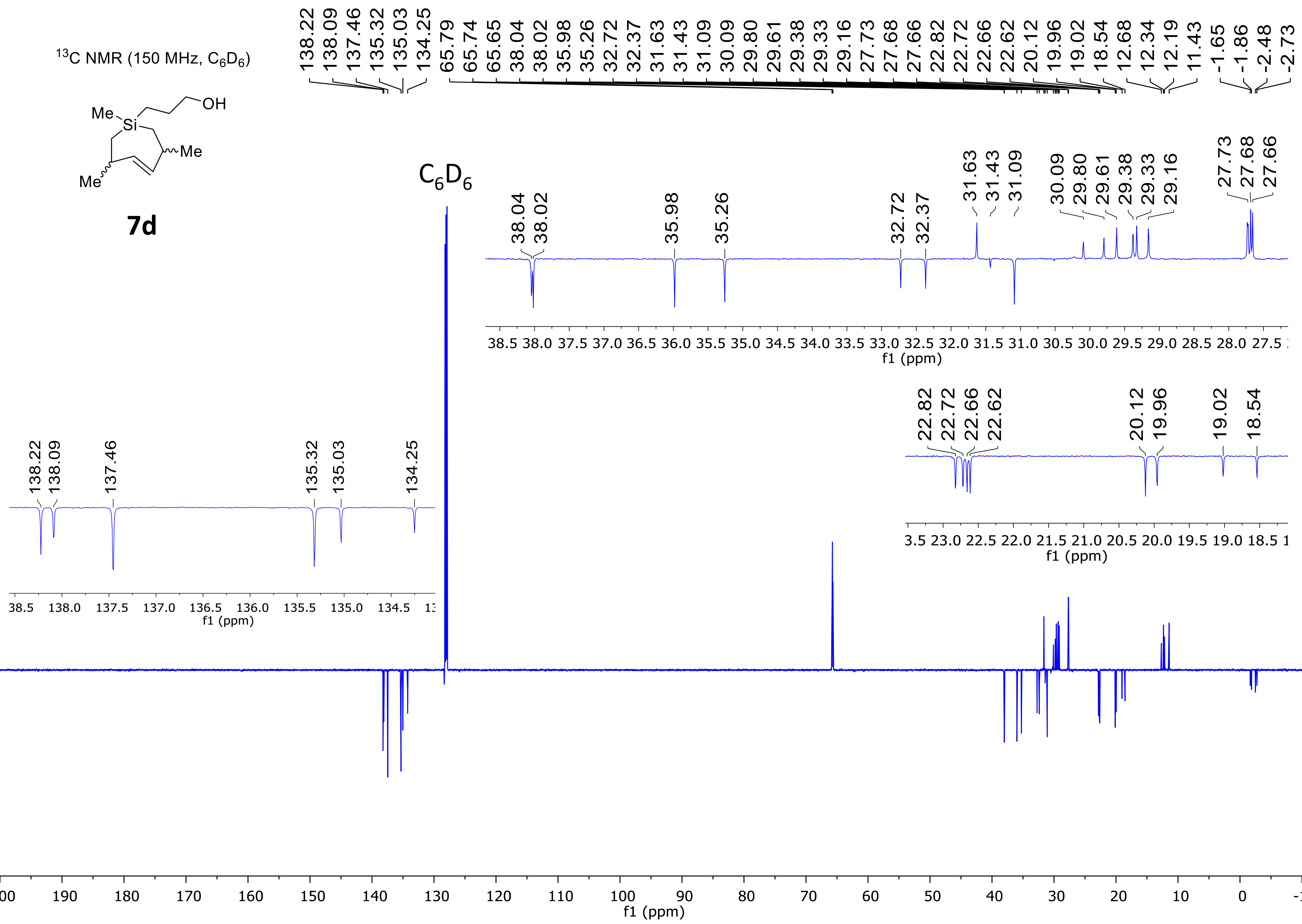

^{13}C NMR (150 MHz, C_6D_6)
Me
Si
OH
Me
Me
7d
C_6D_6
138.22 138.09 137.46 135.32 135.03 134.25 65.79 65.74 65.65 38.04 38.02 35.98 35.26 32.72 32.37 31.63 31.43 31.09 30.09 29.80 29.61 29.38 29.33 29.16 27.73 27.68 27.66 22.82 22.72 22.66 22.62 20.12 19.96 19.02 18.54 12.68 12.34 12.19 11.43 -1.65 -1.86 -2.48 -2.73
f1 (ppm)

^{1}H NMR (400 MHz, CD_3CN)

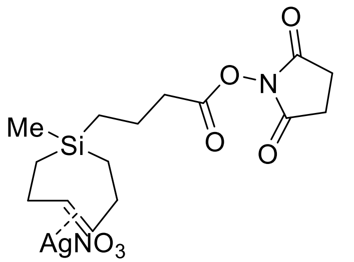

2f

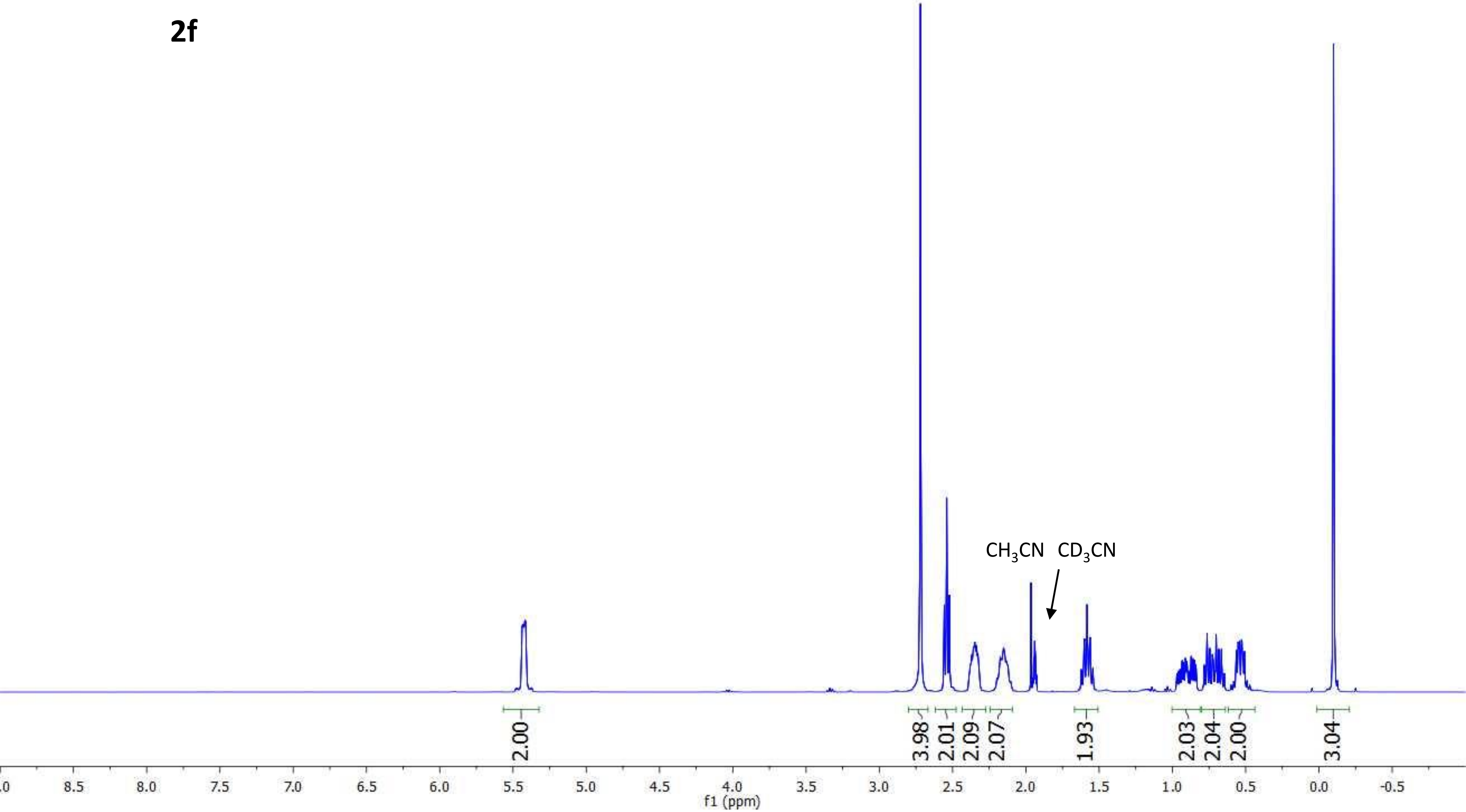

^{13}C NMR (100 MHz, CD_3CN)

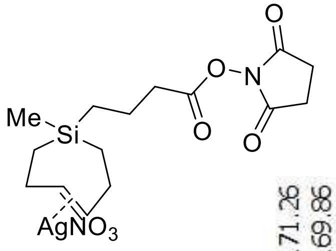

2f

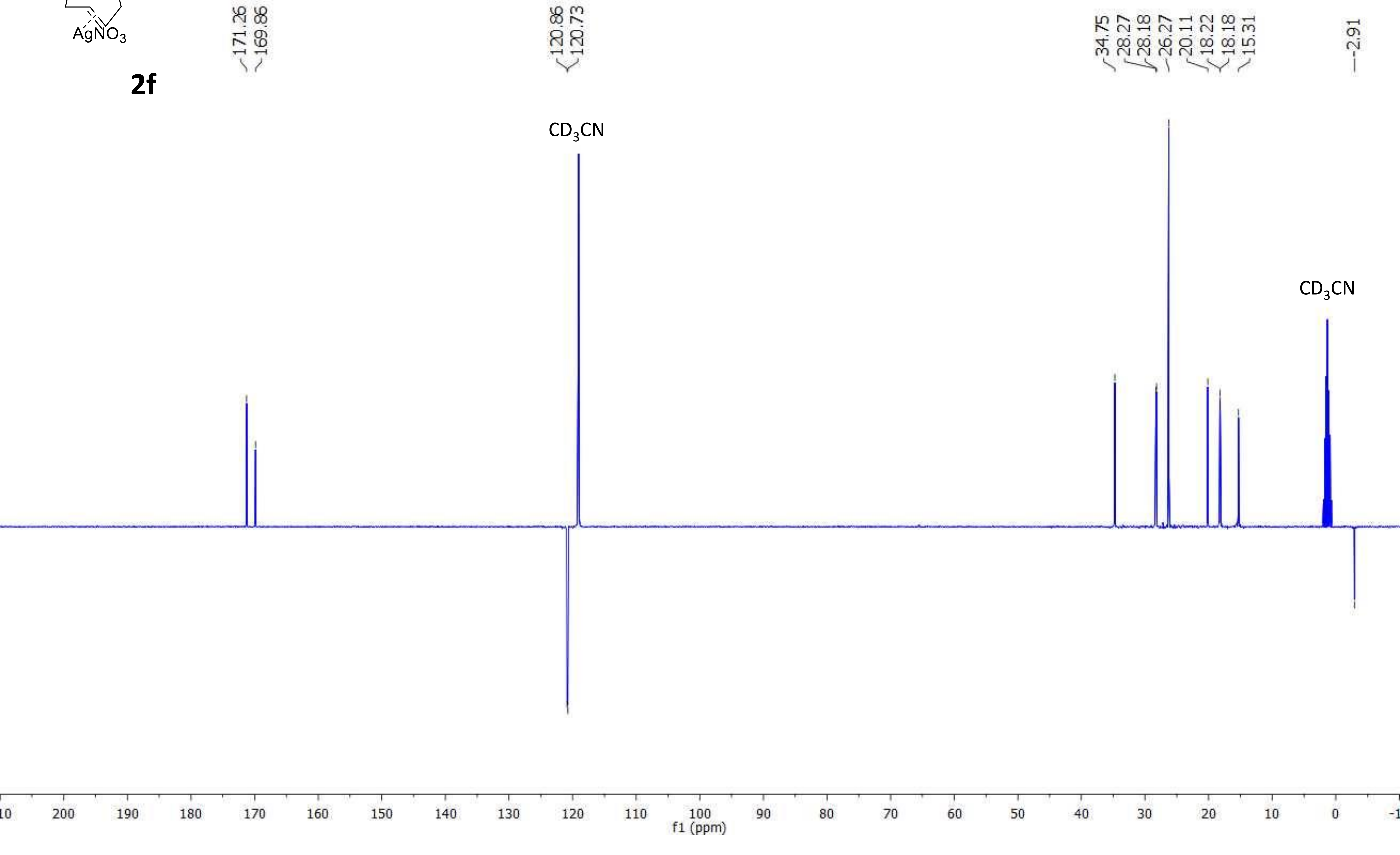

^{1}H NMR (600 MHz, CD_3OD)

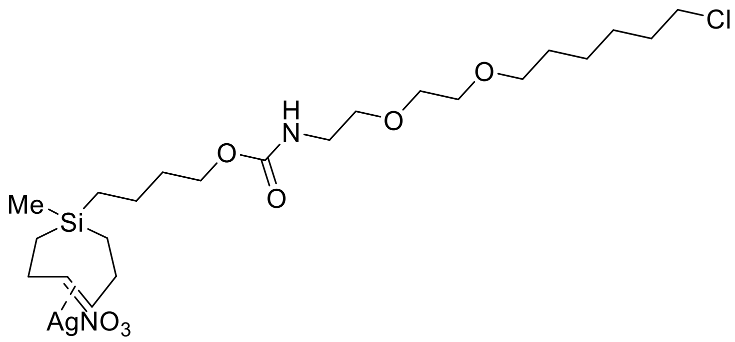

2g

AgSiTCH-Halo

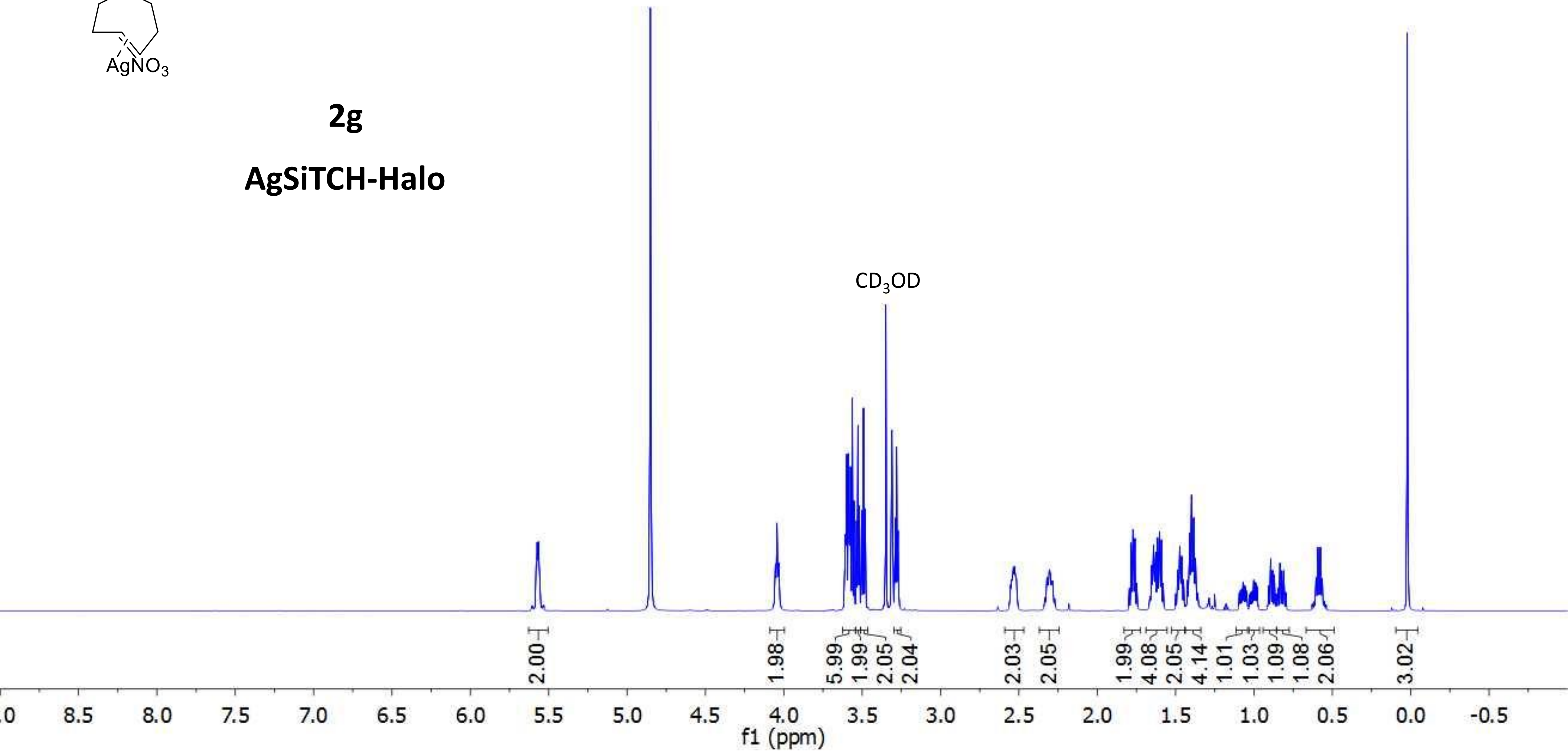

^{13}C NMR (100 MHz, CD_3OD)

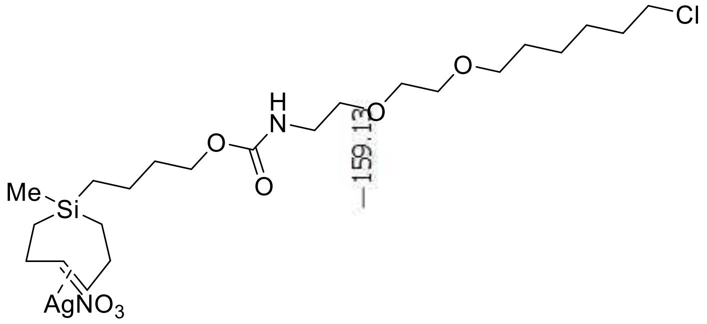

2g

AgSiTCH-Halo

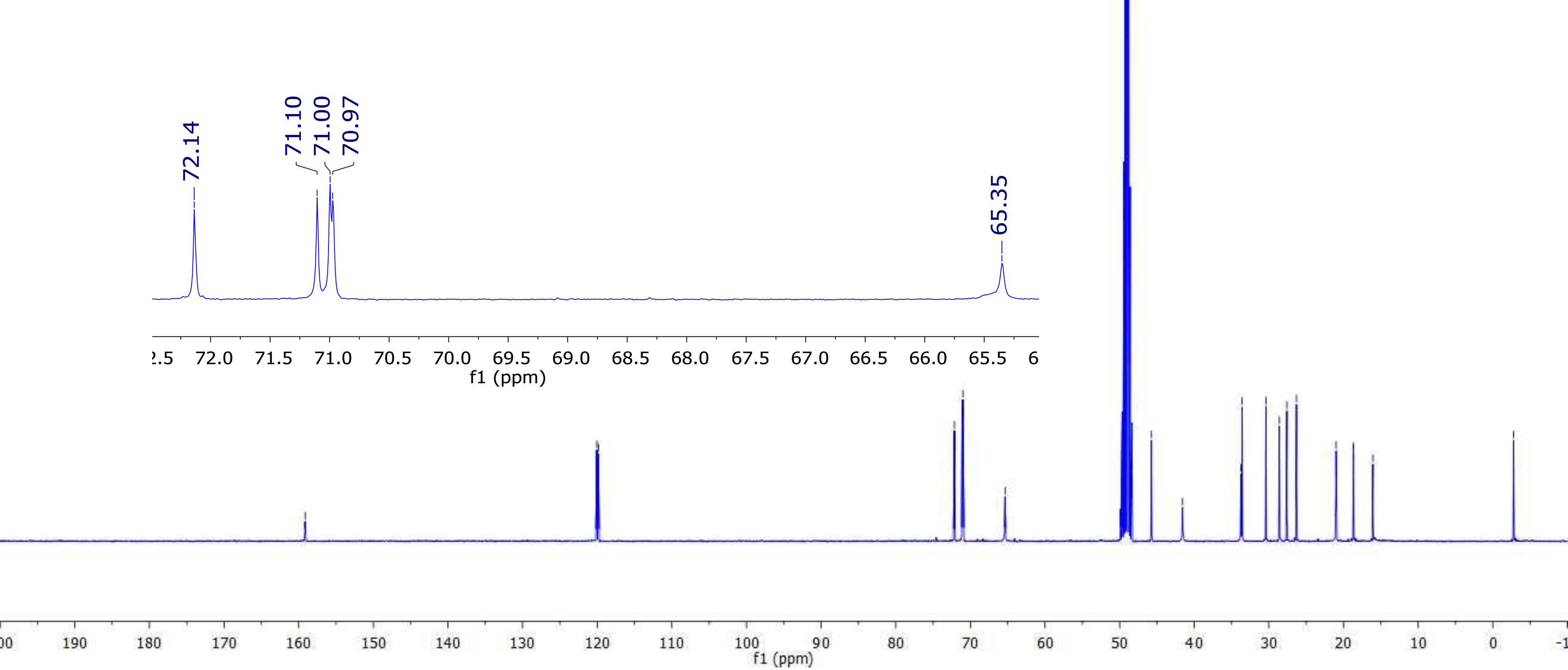

HMQC (400 MHz, CD_3OD)

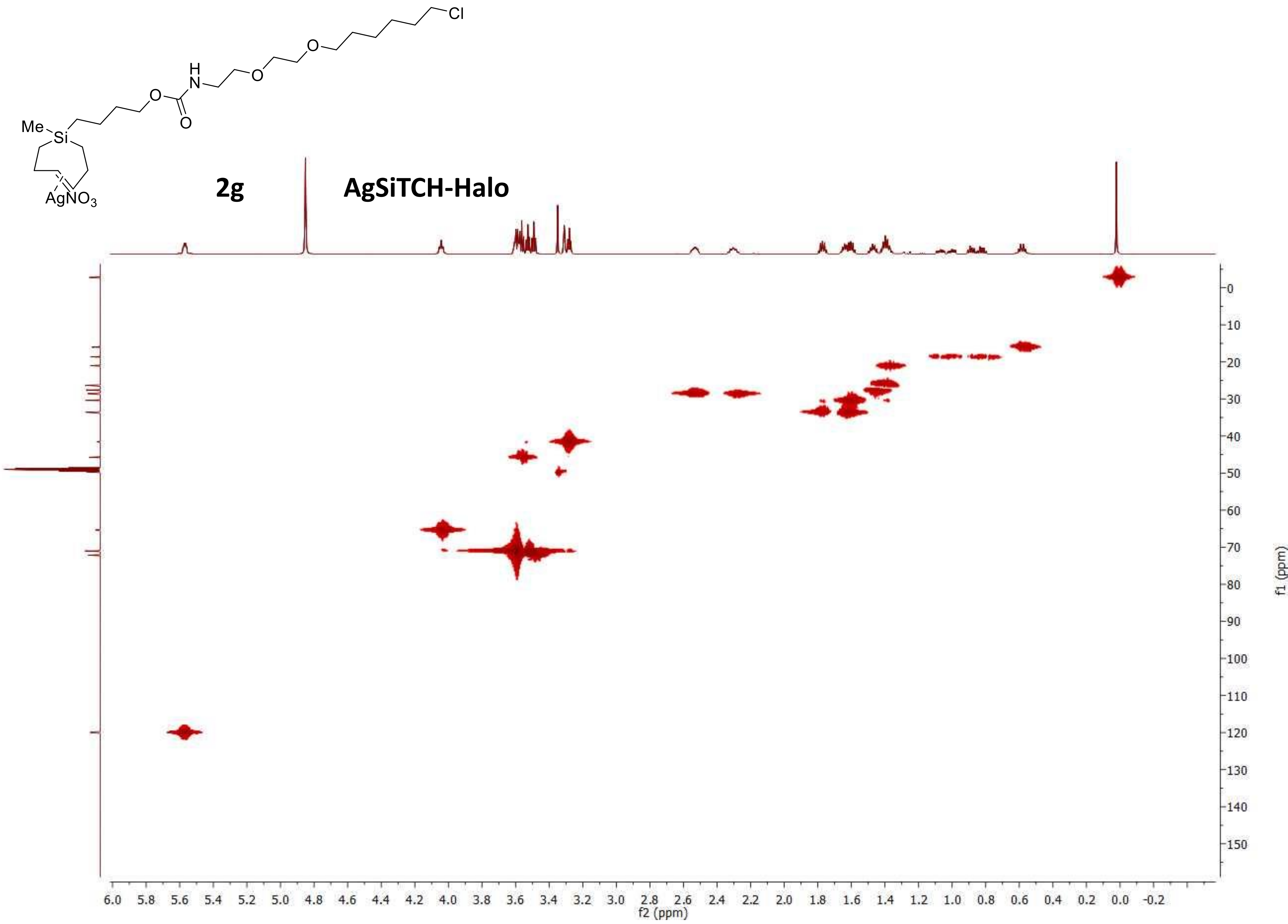

^{1}H NMR (600 MHz, $CDCl_3$)

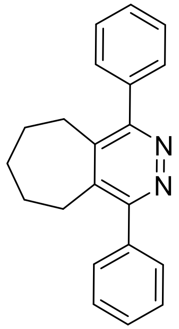

8

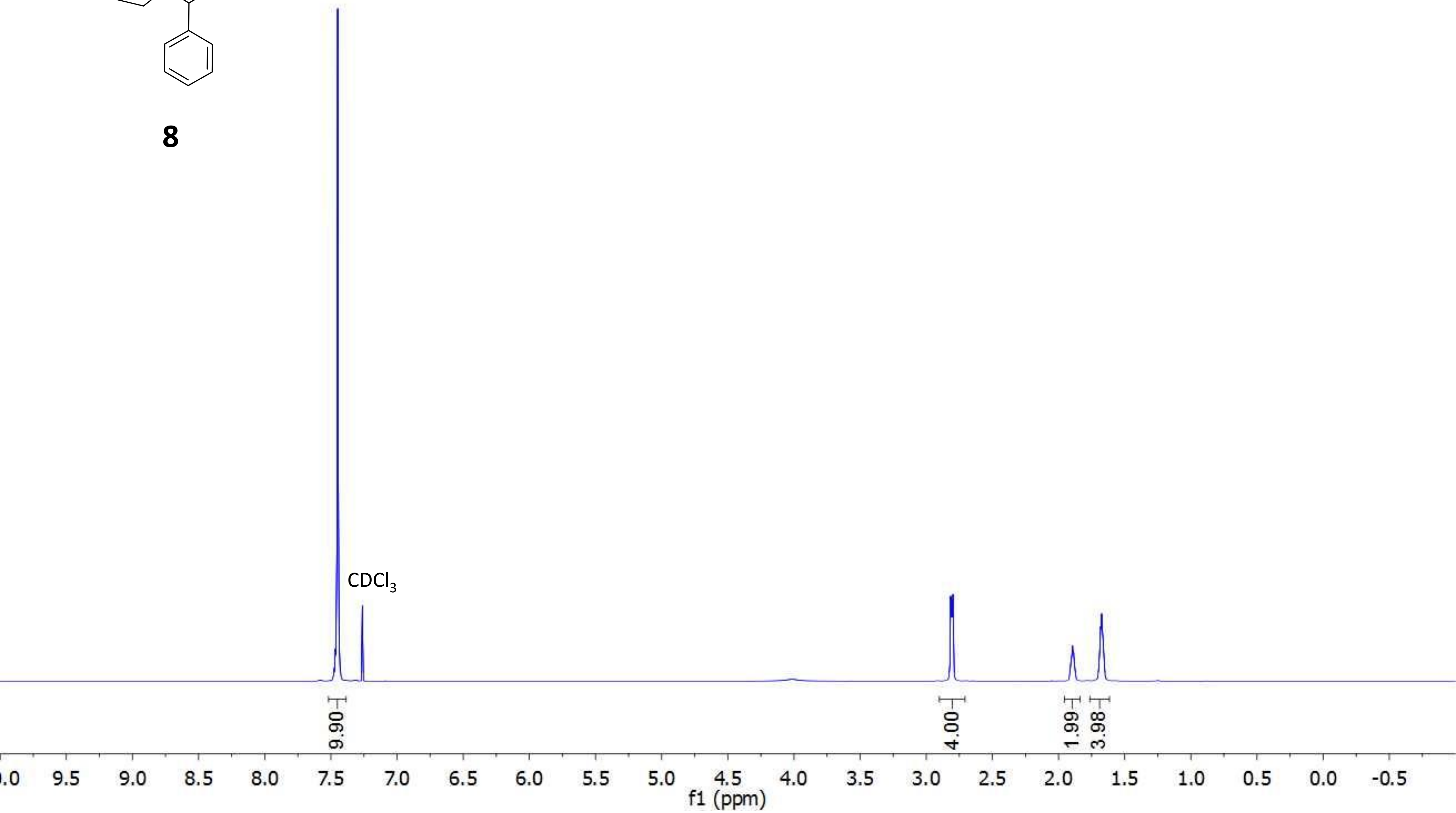

^{13}C NMR (150 MHz, $CDCl_3$)

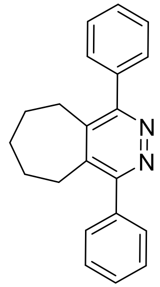

8

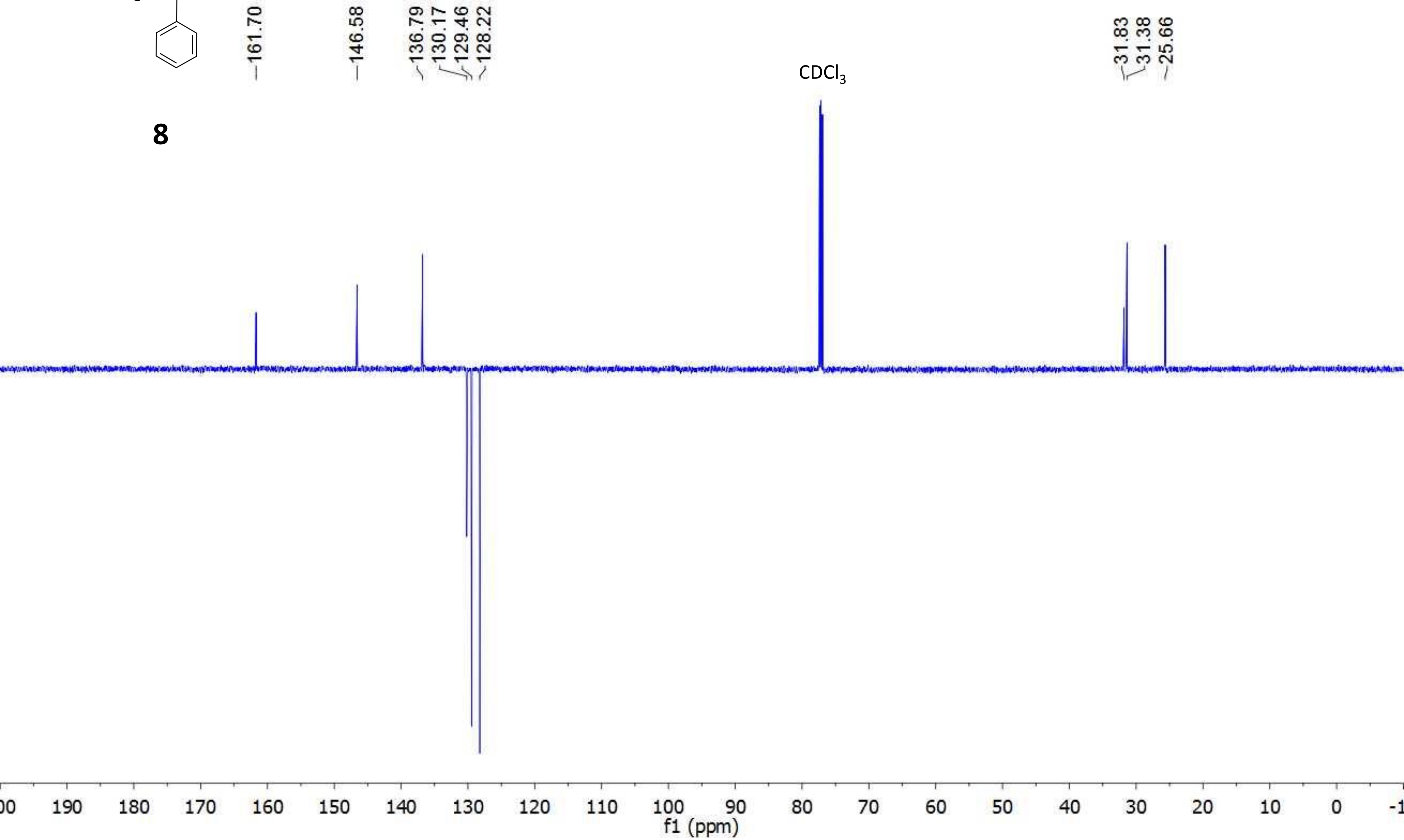

^{1}H NMR (600 MHz, $CDCl_3$)

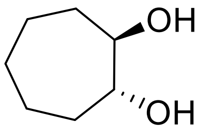

10

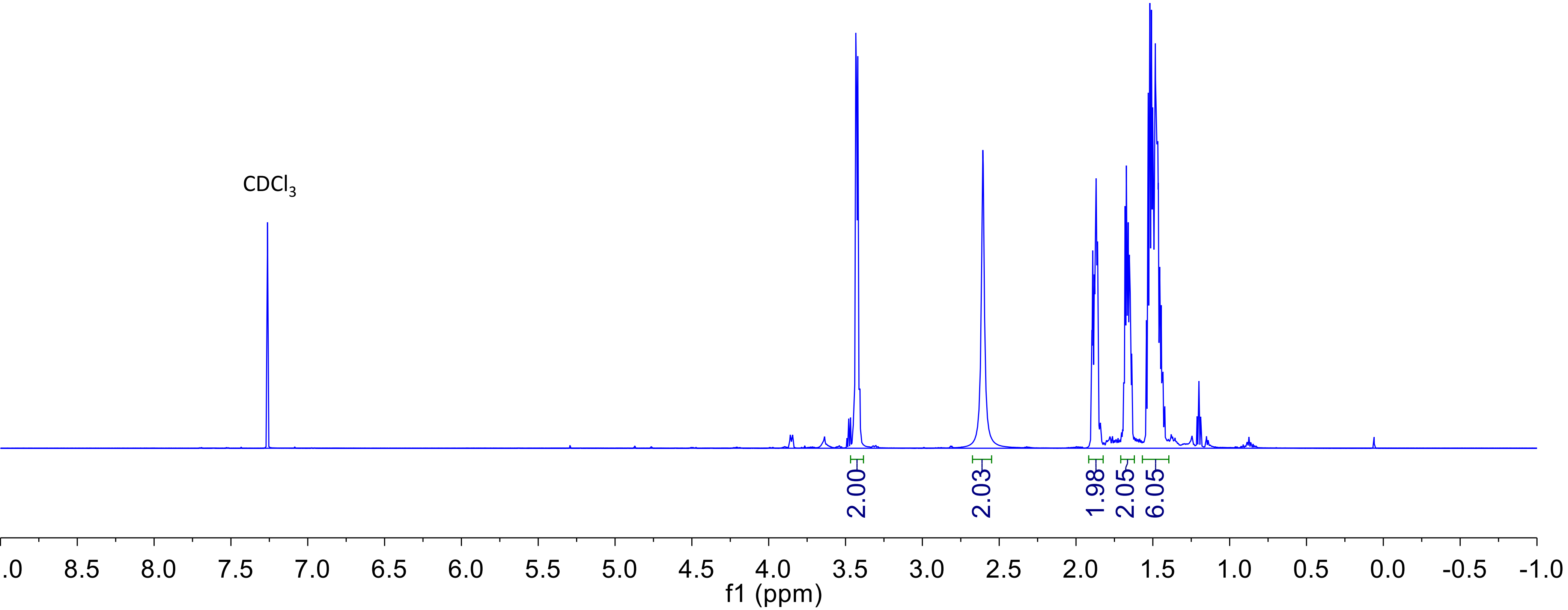

^{13}C NMR (100 MHz, $CDCl_3$)

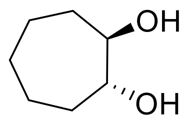

10

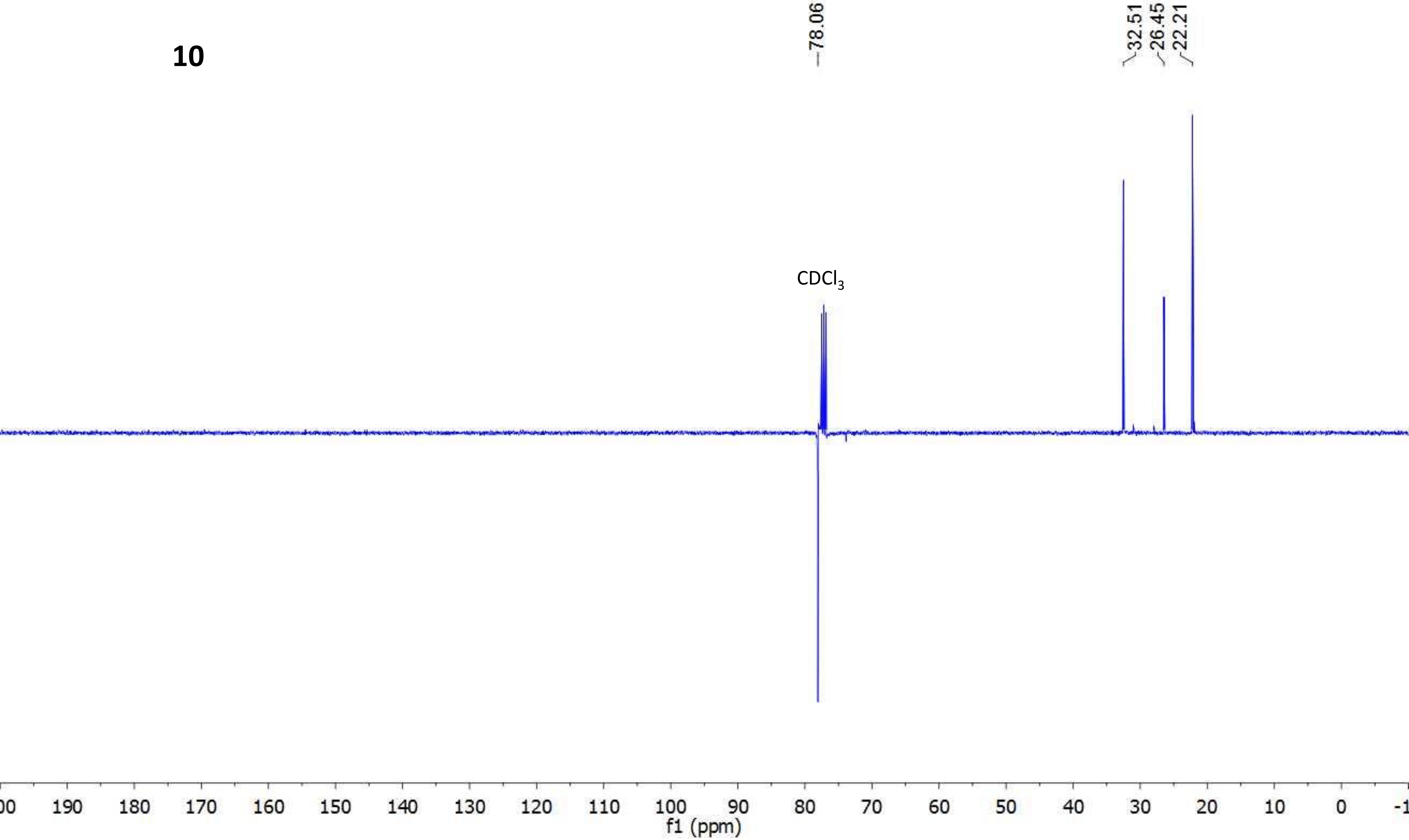

^1H NMR (400 MHz, $CDCl_3$)

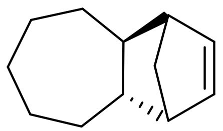

9

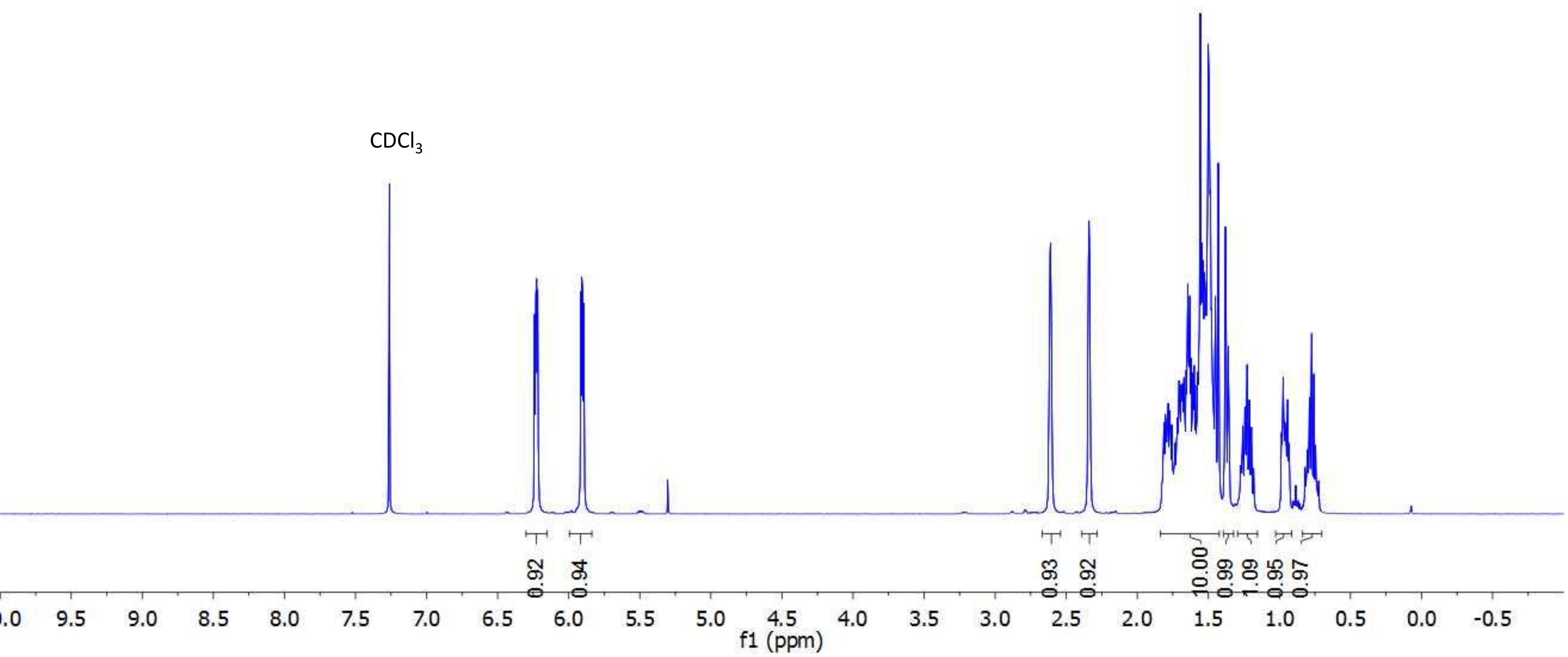

^{13}C NMR (100 MHz, $CDCl_3$)

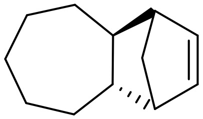

9

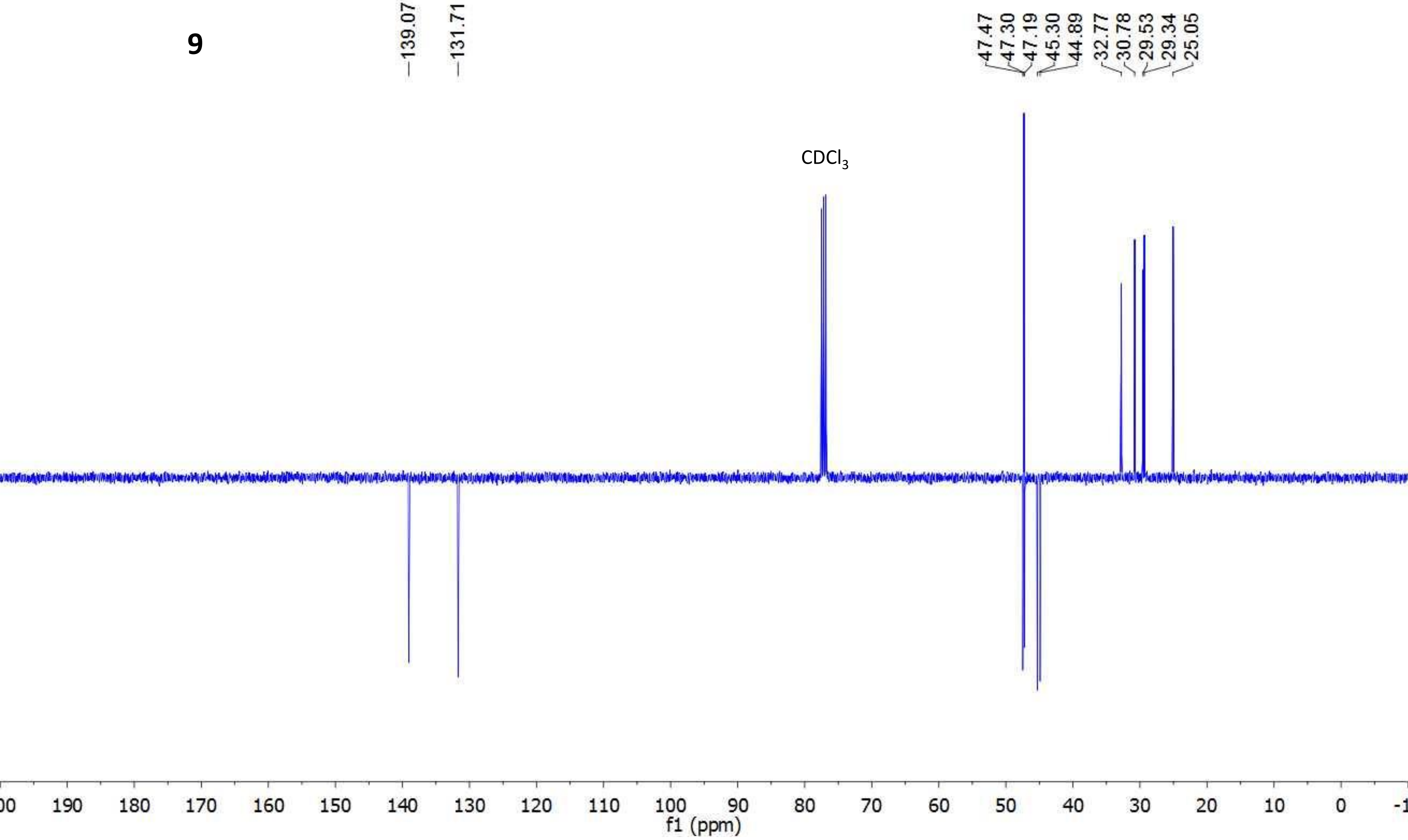

^{1}H NMR (600 MHz, $CDCl_3$)

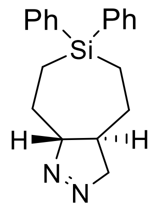

12

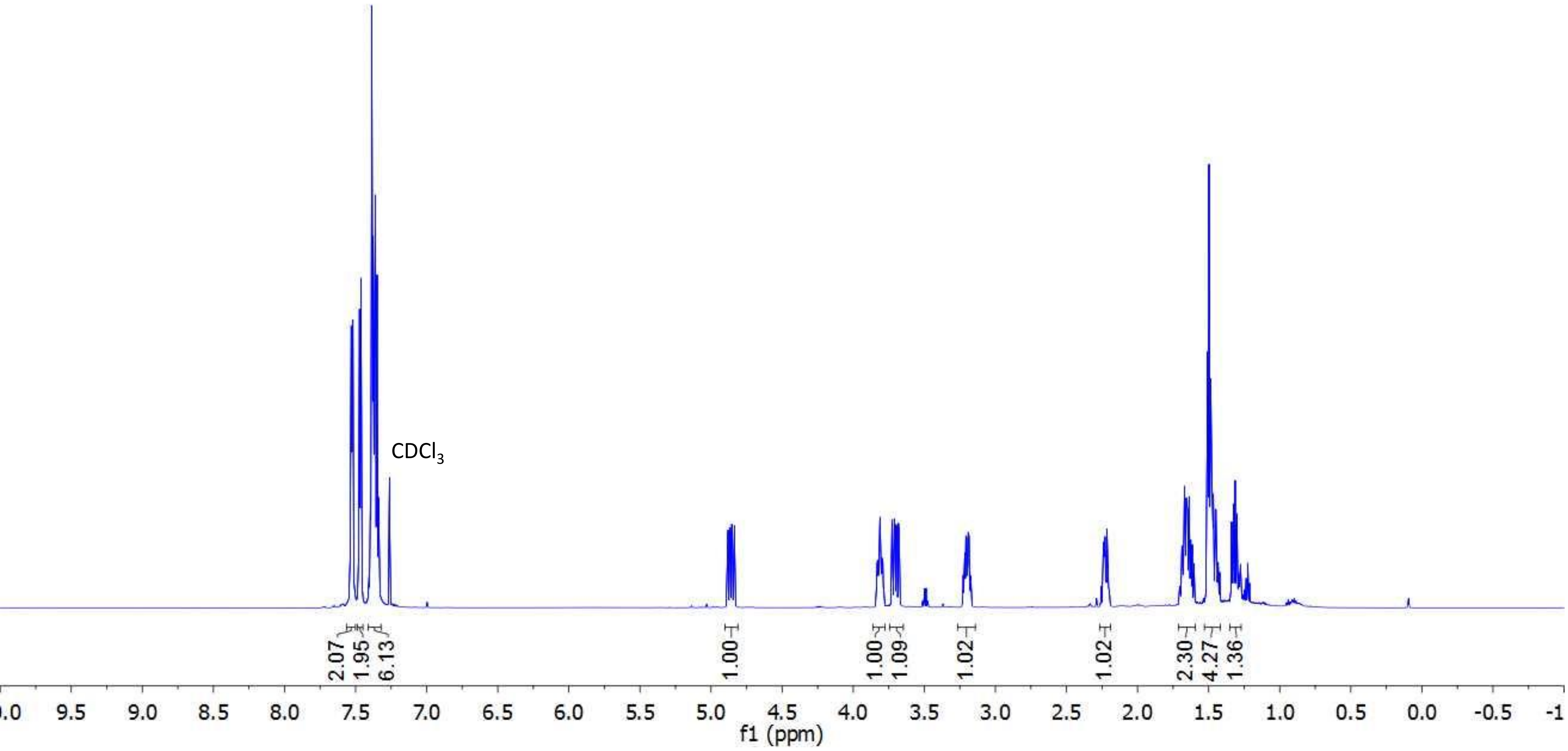

^{13}C NMR (100 MHz, $CDCl_3$)

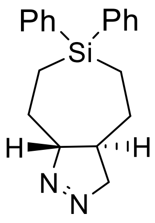

12

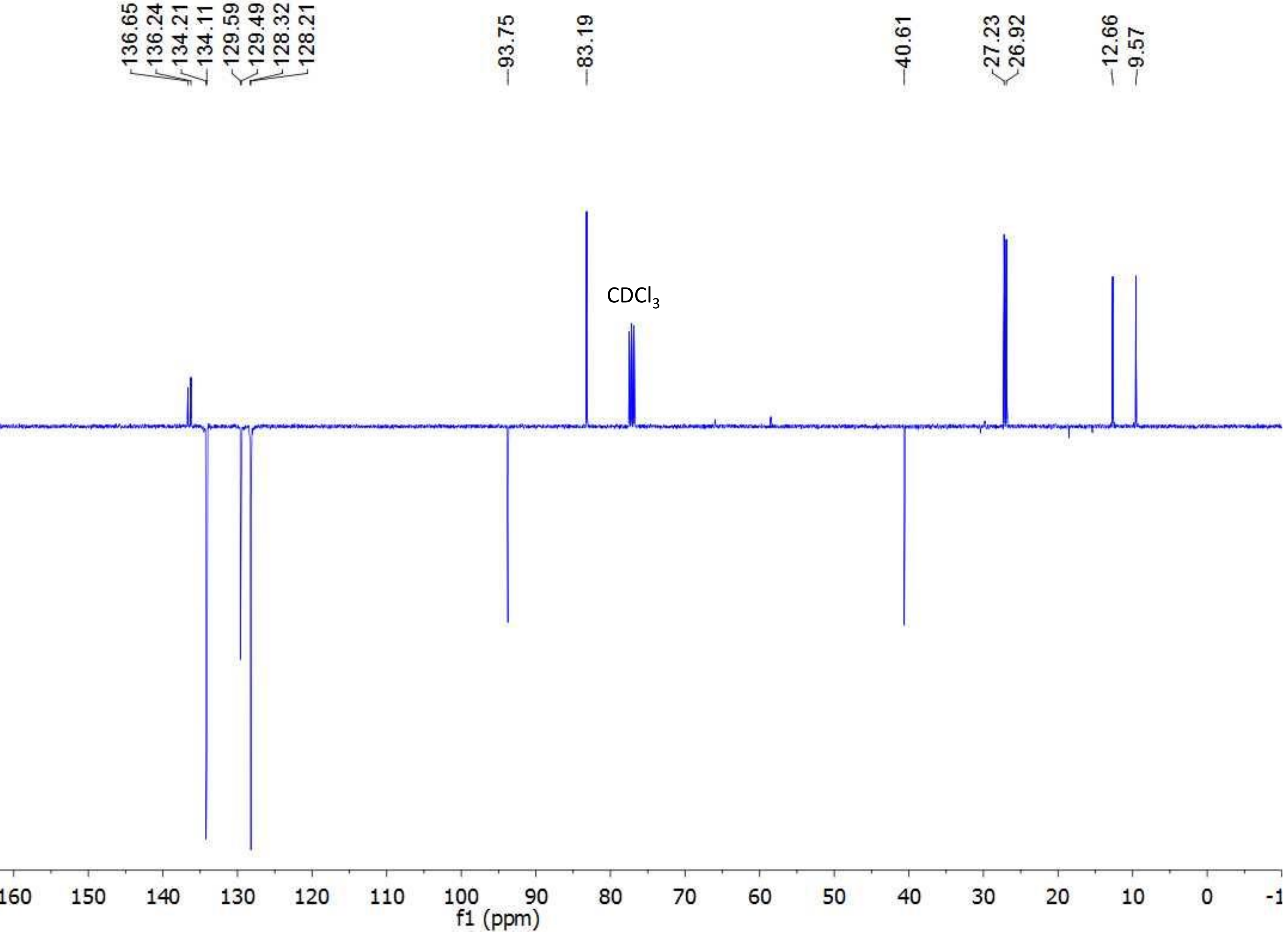

COSY (400 MHz, $CDCl_3$)

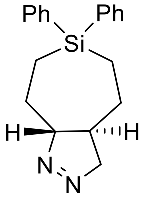

12

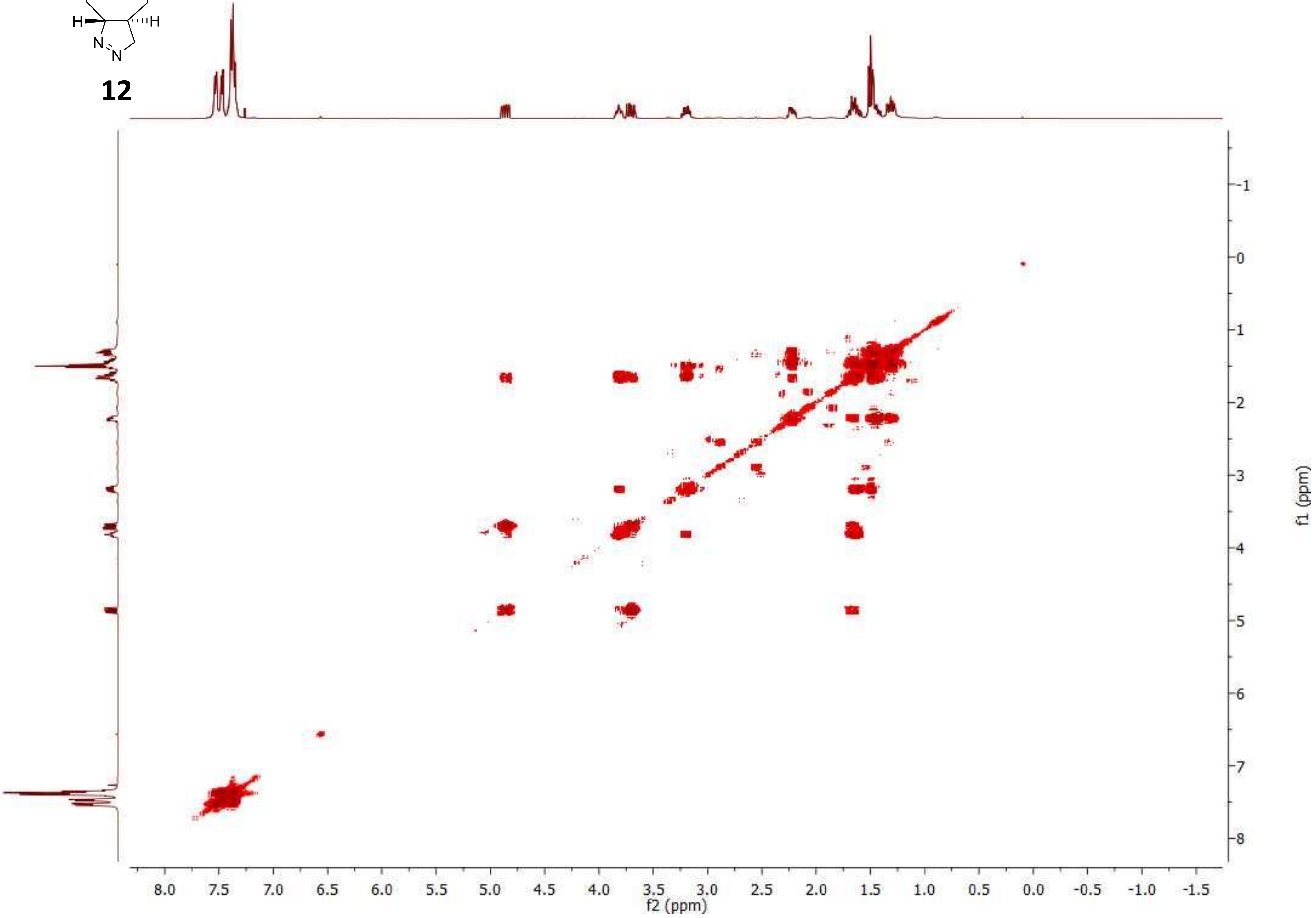

HMQC (400 MHz, $CDCl_3$)

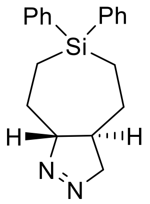

12

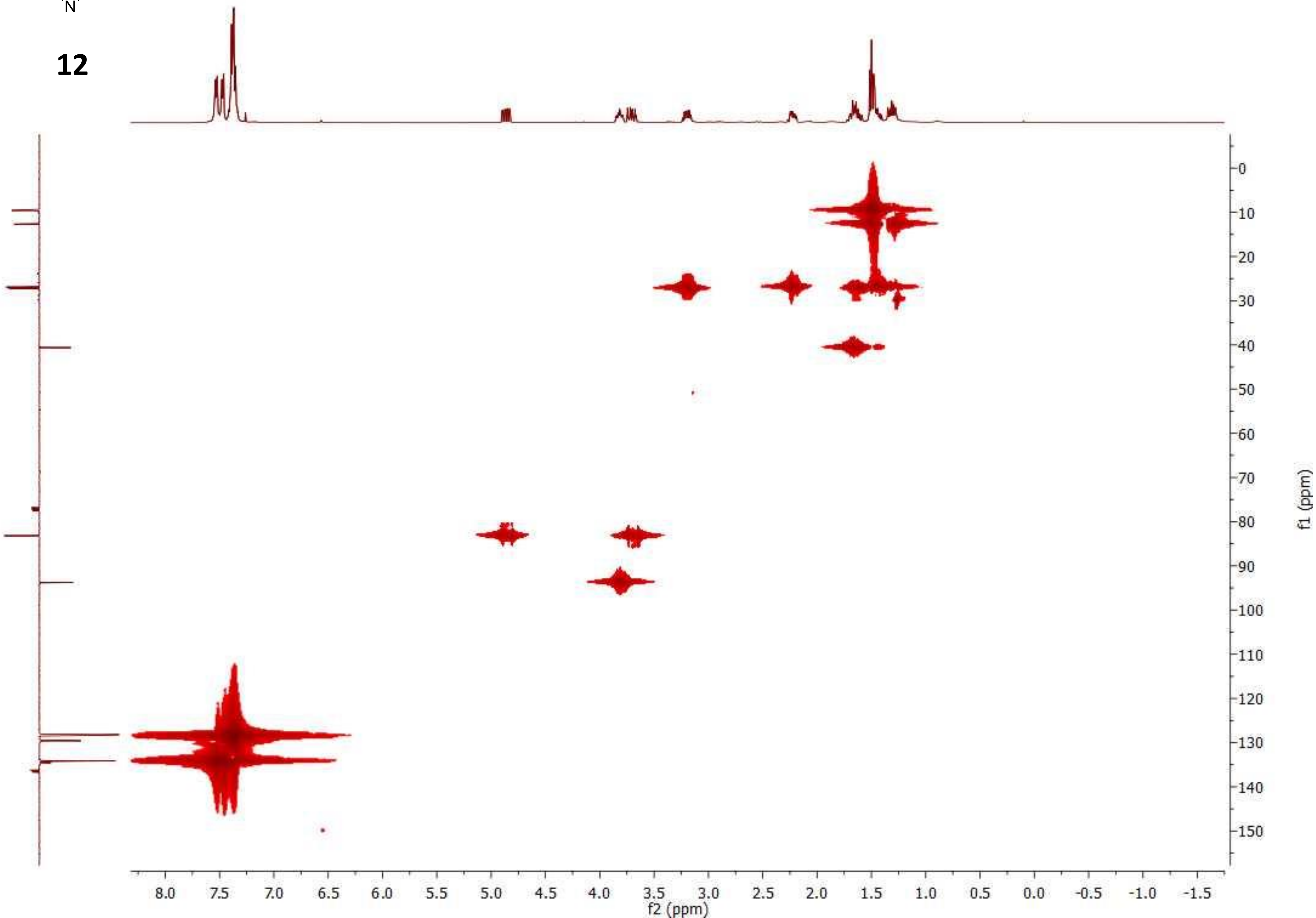

^{1}H NMR (600 MHz, $CDCl_3$)

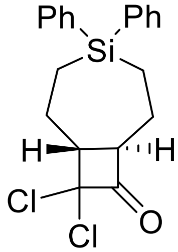

13

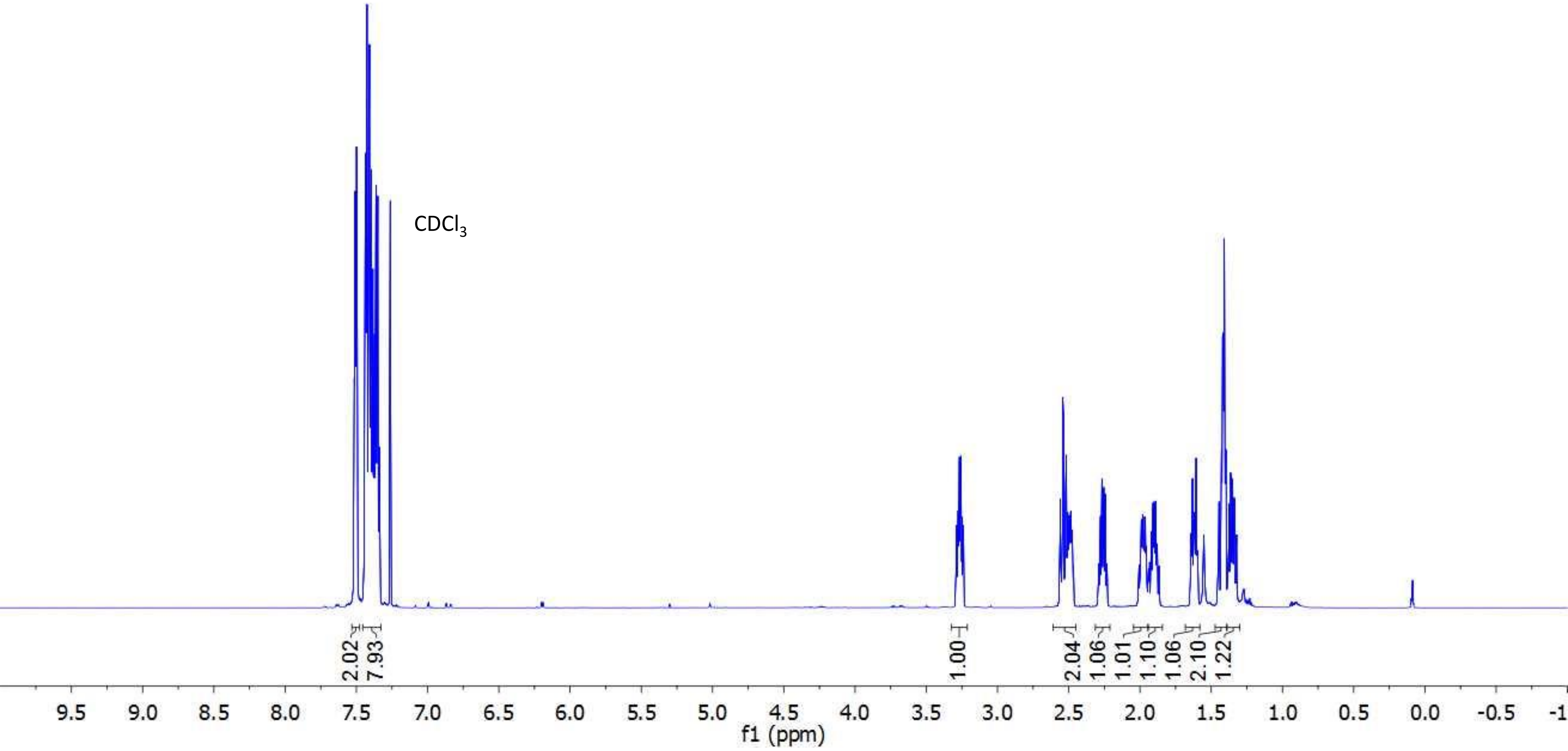

^{13}C NMR (150 MHz, $CDCl_3$)

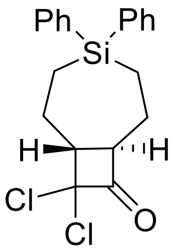

13

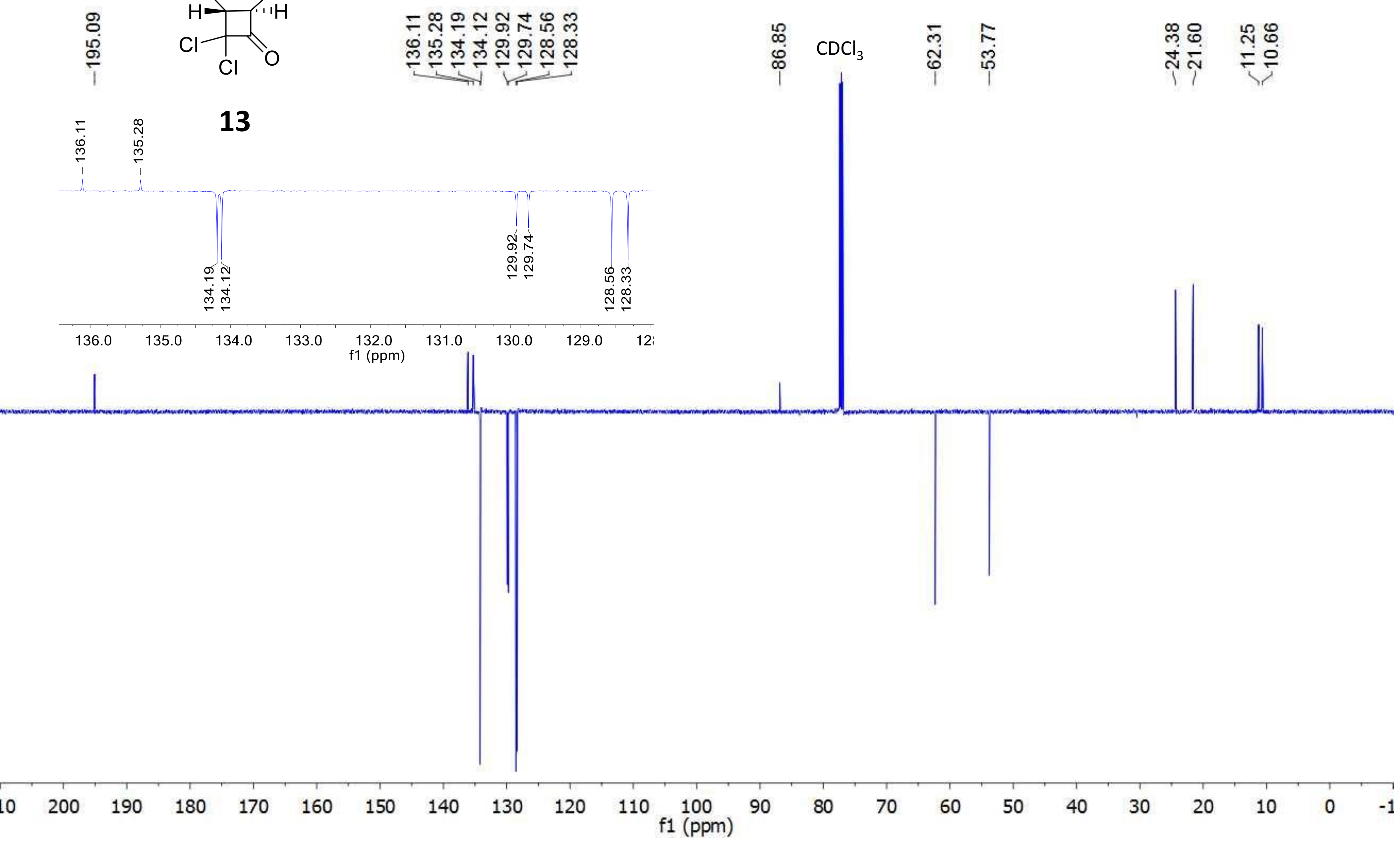

COSY (400 MHz, $CDCl_3$)

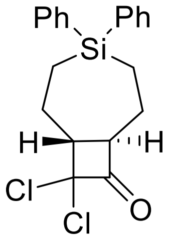

13

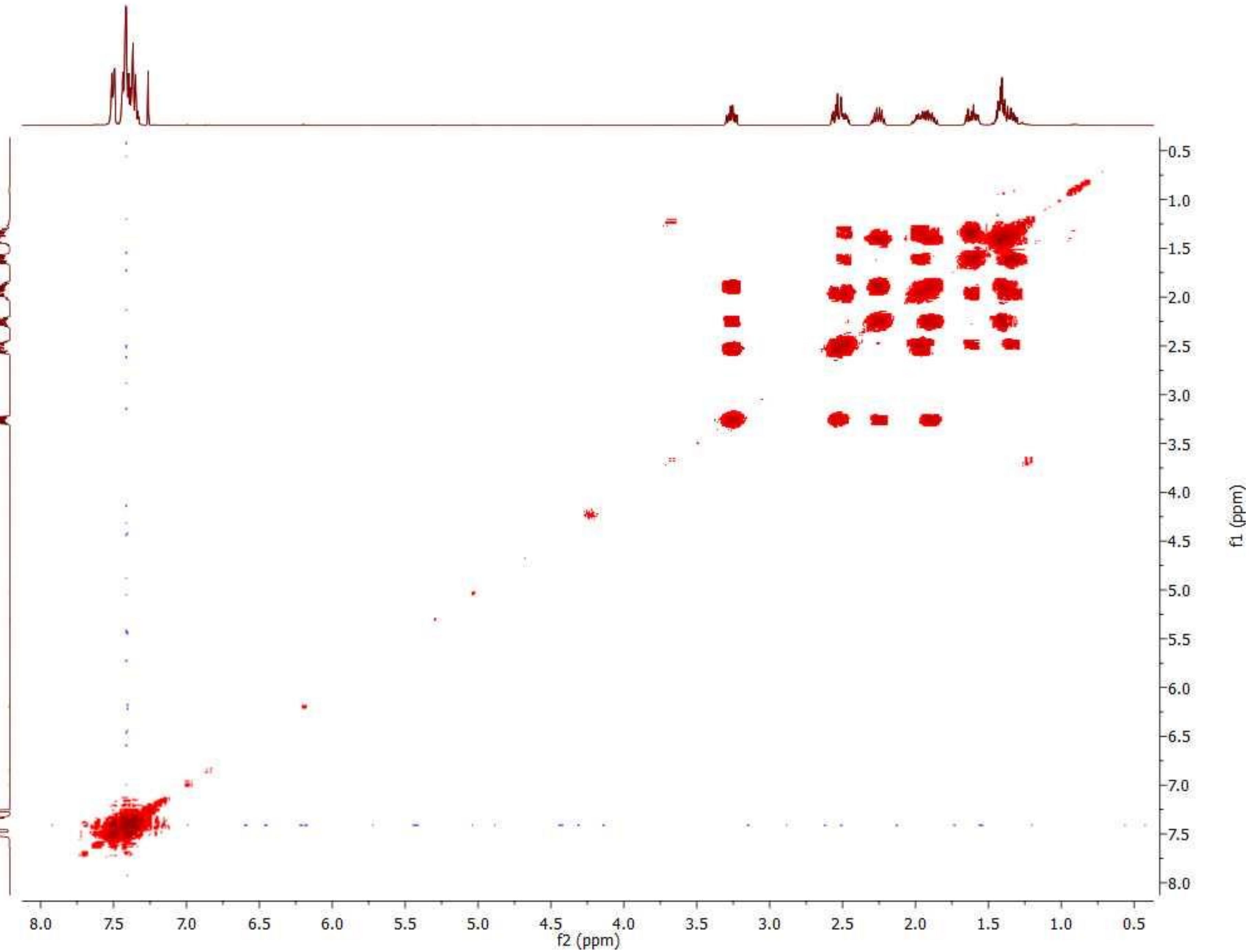

HMQC (400 MHz, $CDCl_3$)

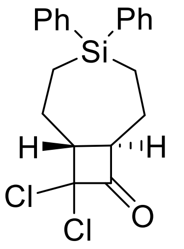

13

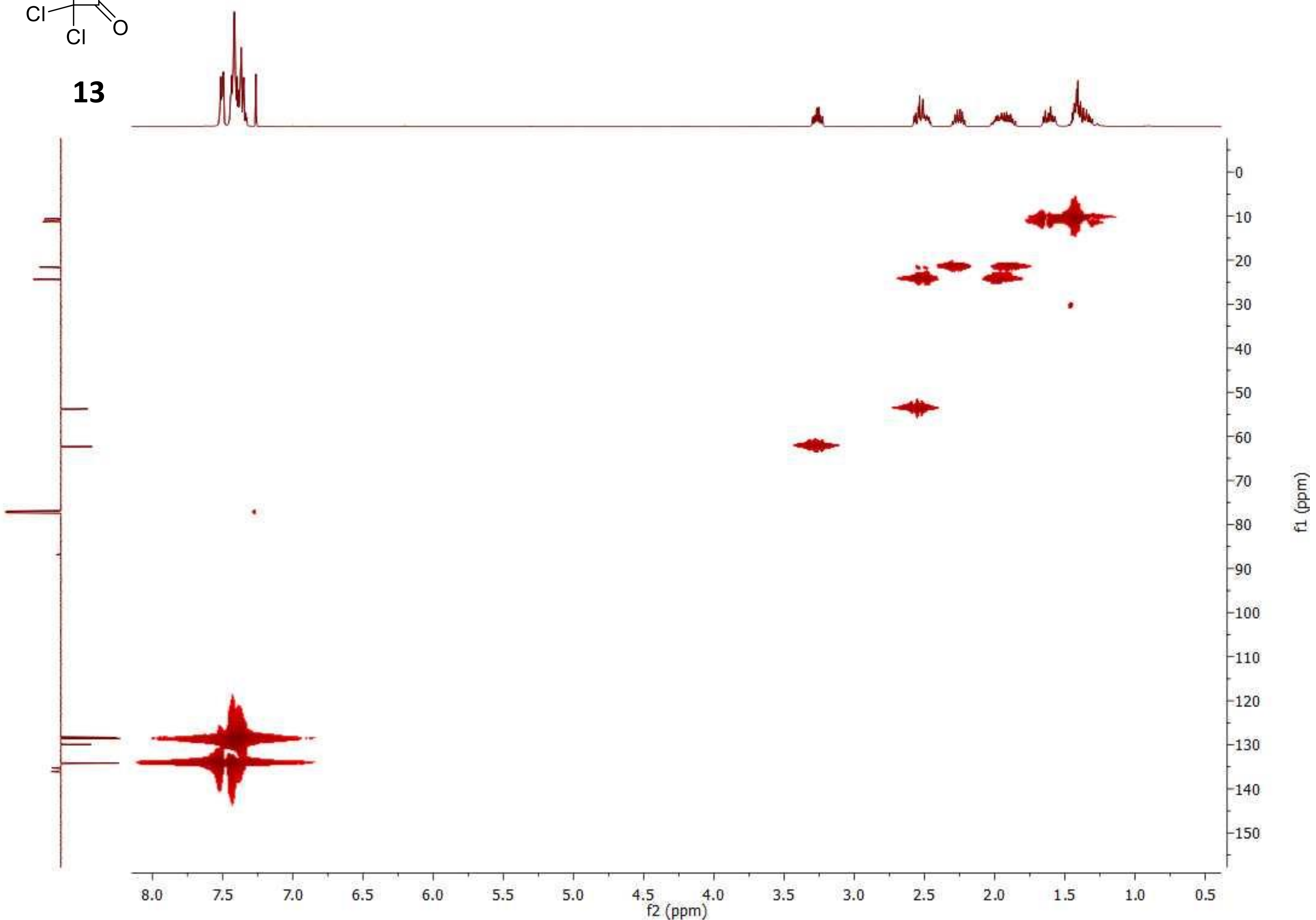

^{1}H NMR (600 MHz, DMSO-d$_6$)

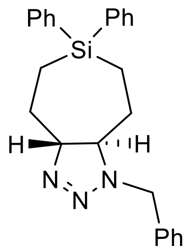

14

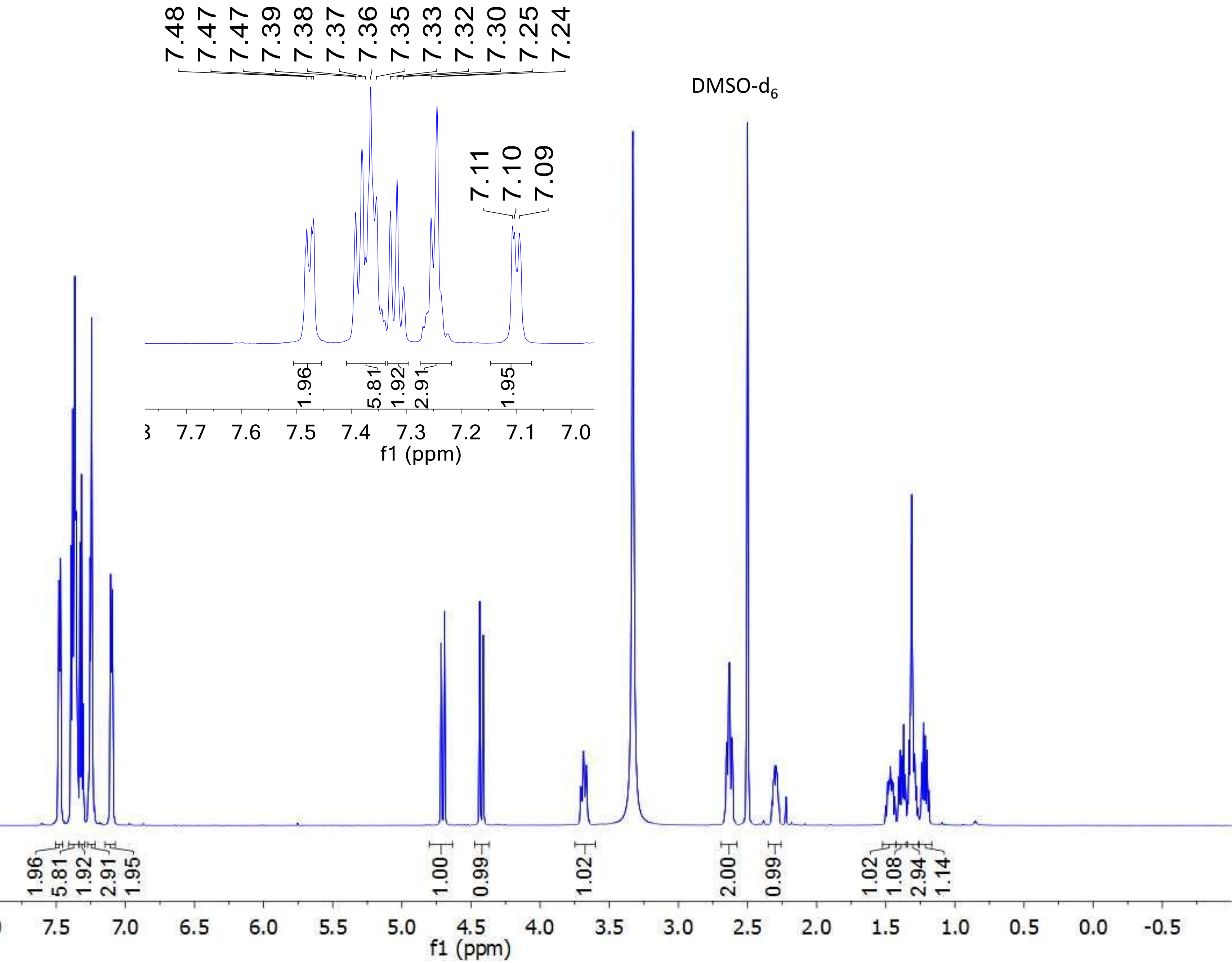

^{13}C NMR (150 MHz, DMSO-d_6)

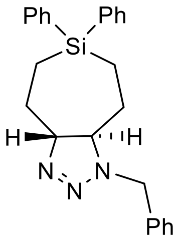

14

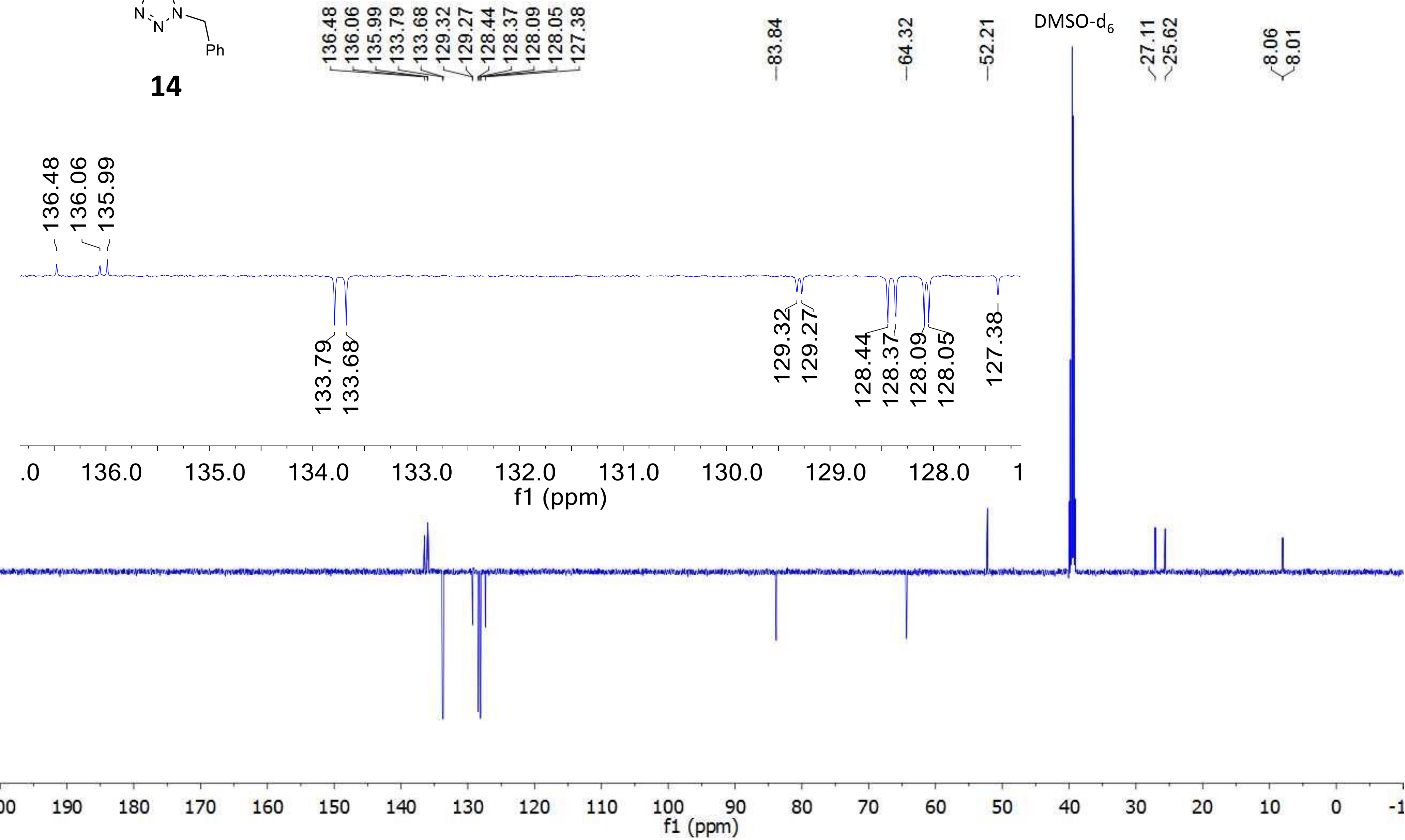

^{1}H NMR (400 MHz, $CDCl_3$)

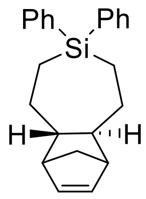

11

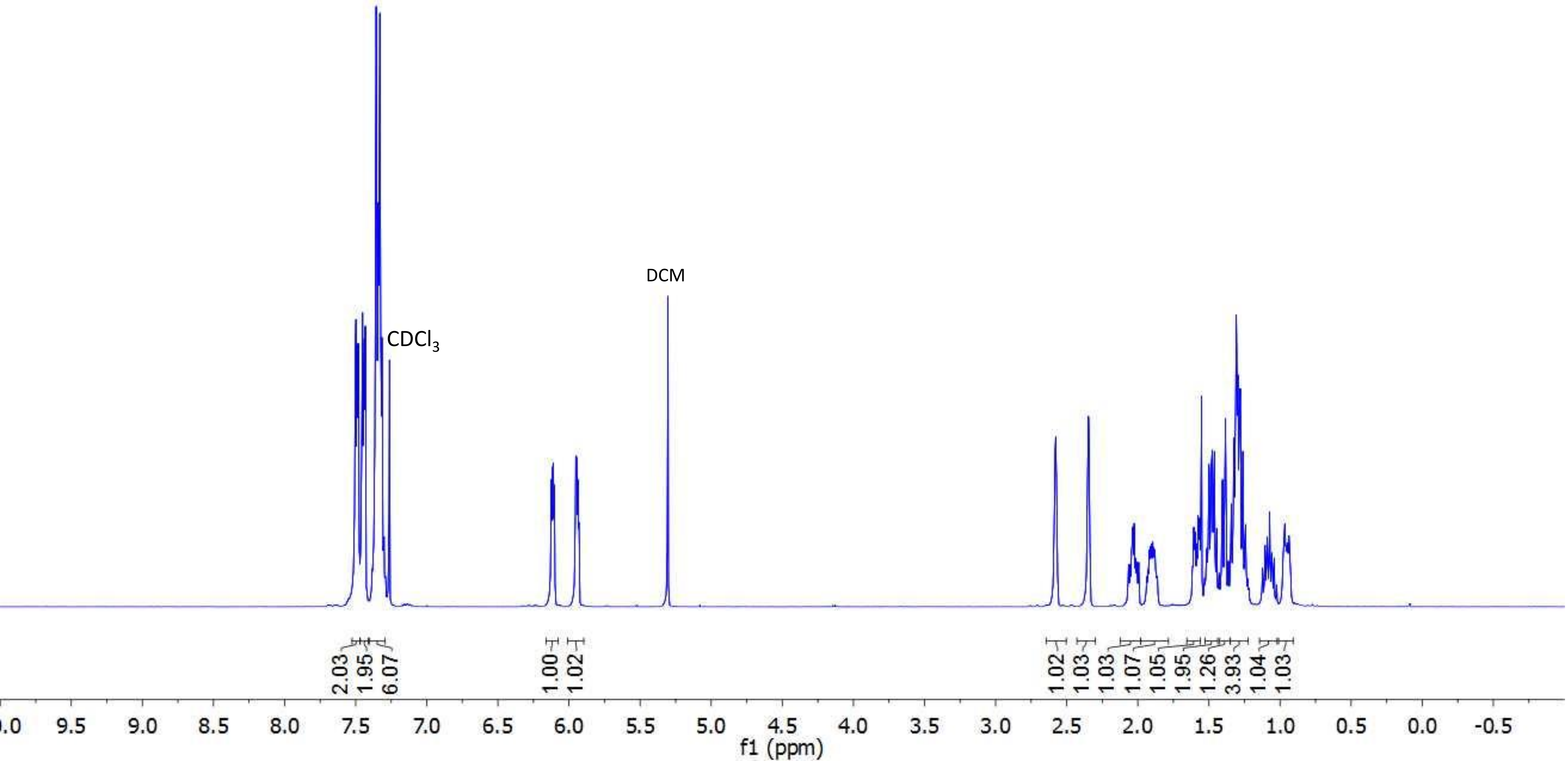

^{13}C NMR (150 MHz, C_6D_6)

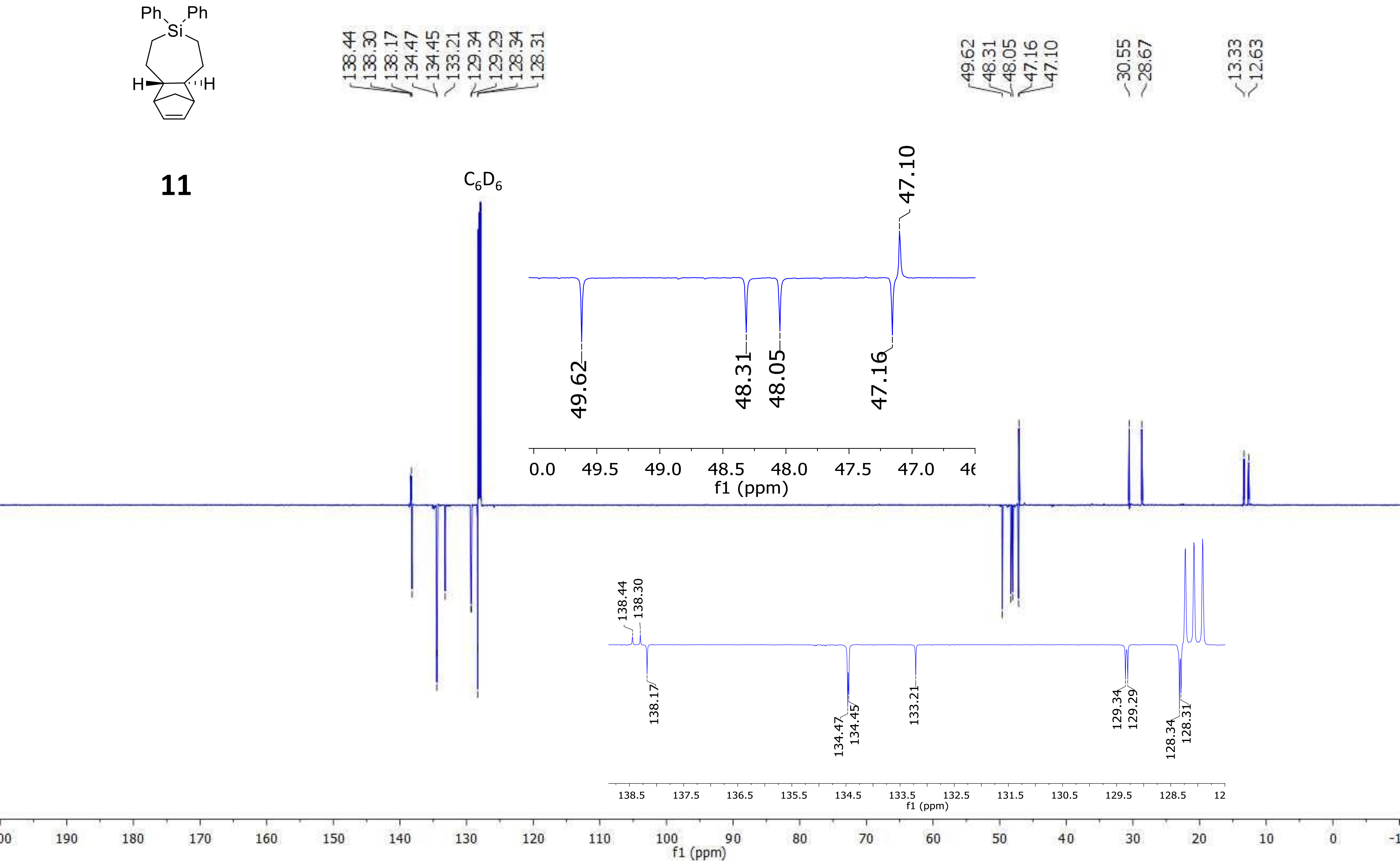

11

^{1}H NMR (600 MHz, $CDCl_3$)

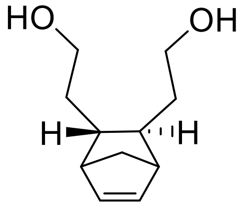

15

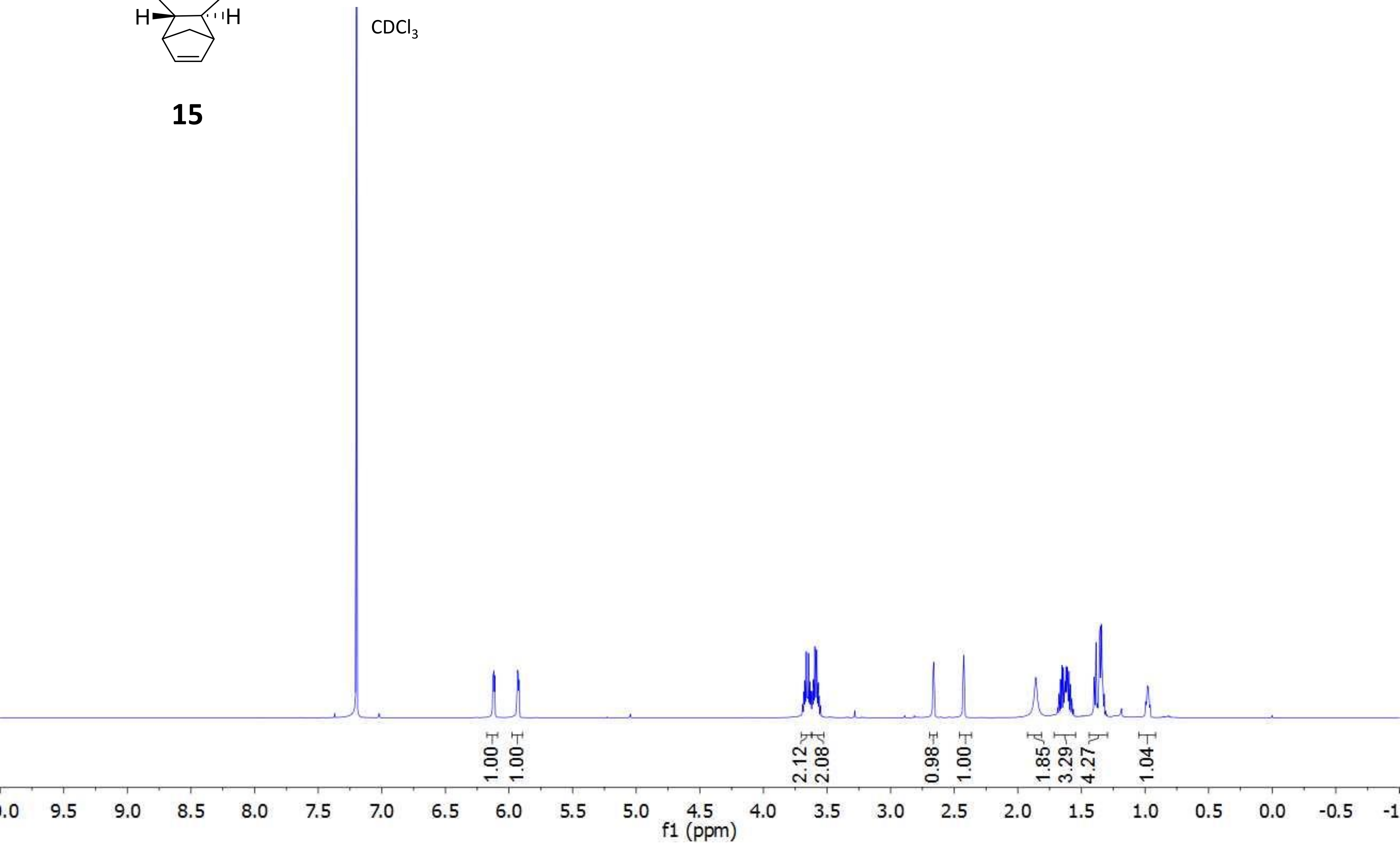

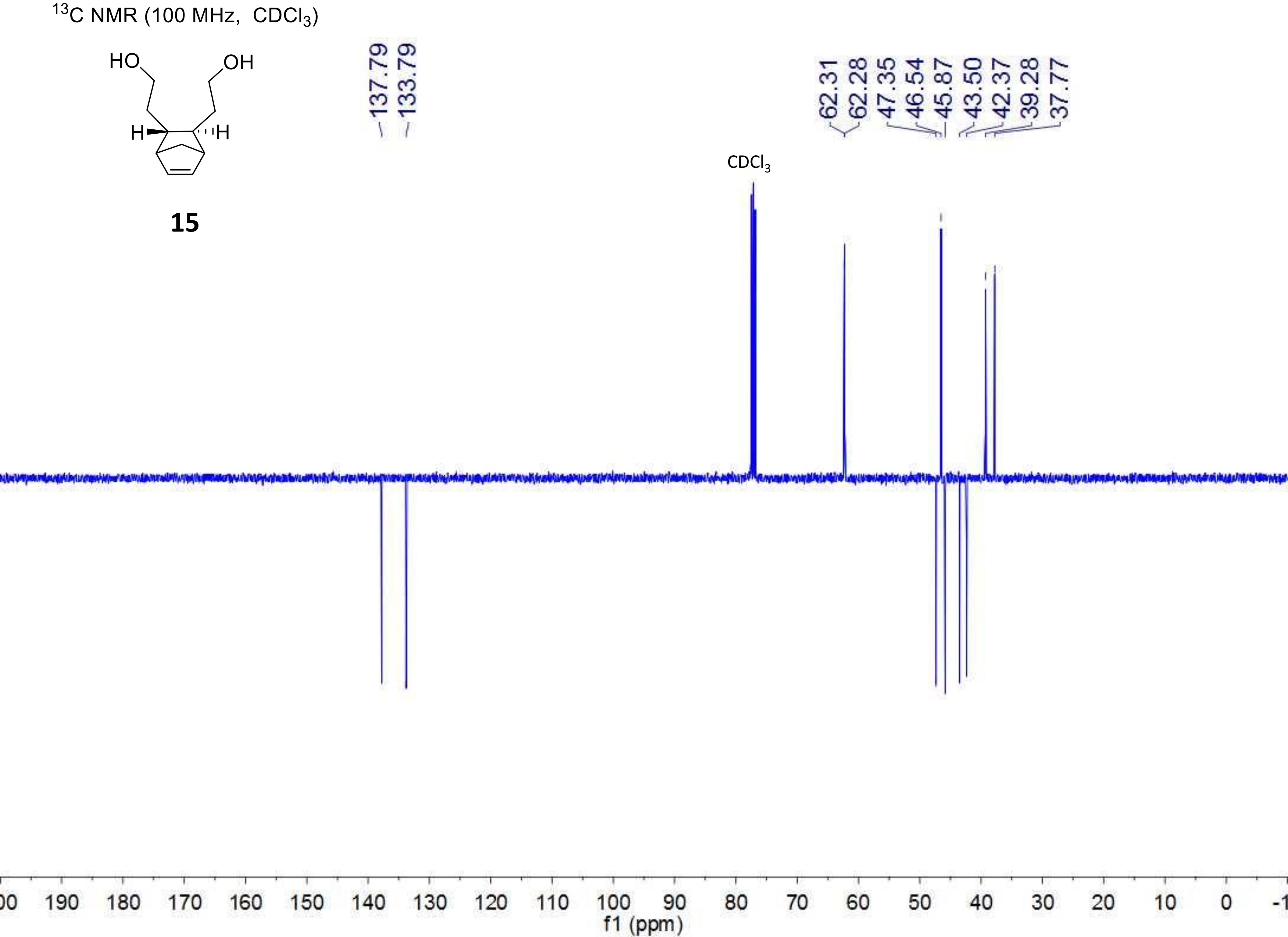

13C NMR (100 MHz, CDCl3)
HO
OH
H
H
15
137.79
133.79
62.31
62.28
47.35
46.54
45.87
43.50
42.37
39.28
37.77
CDCl3
200 190 180 170 160 150 140 130 120 110 100 90 80 70 60 50 40 30 20 10 0 -10
f1 (ppm)

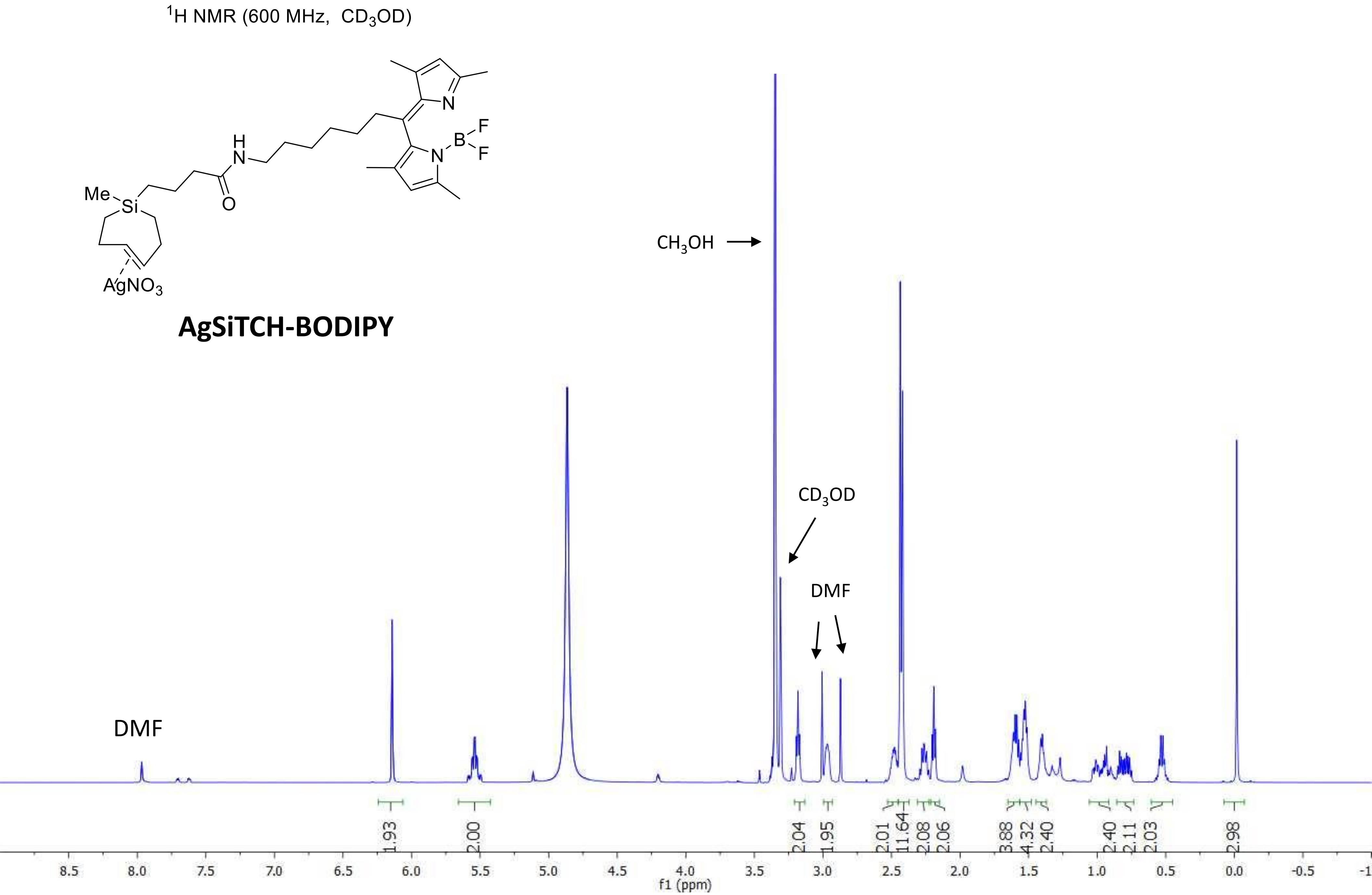

1H NMR (600 MHz, CD3OD)
AgSiTCH-BODIPY
CH3OH
CD3OD
DMF
DMF
1.93
2.00
2.04
1.95
2.01
11.64
2.08
2.06
3.88
4.32
2.40
2.40
2.11
2.03
2.98
f1 (ppm)

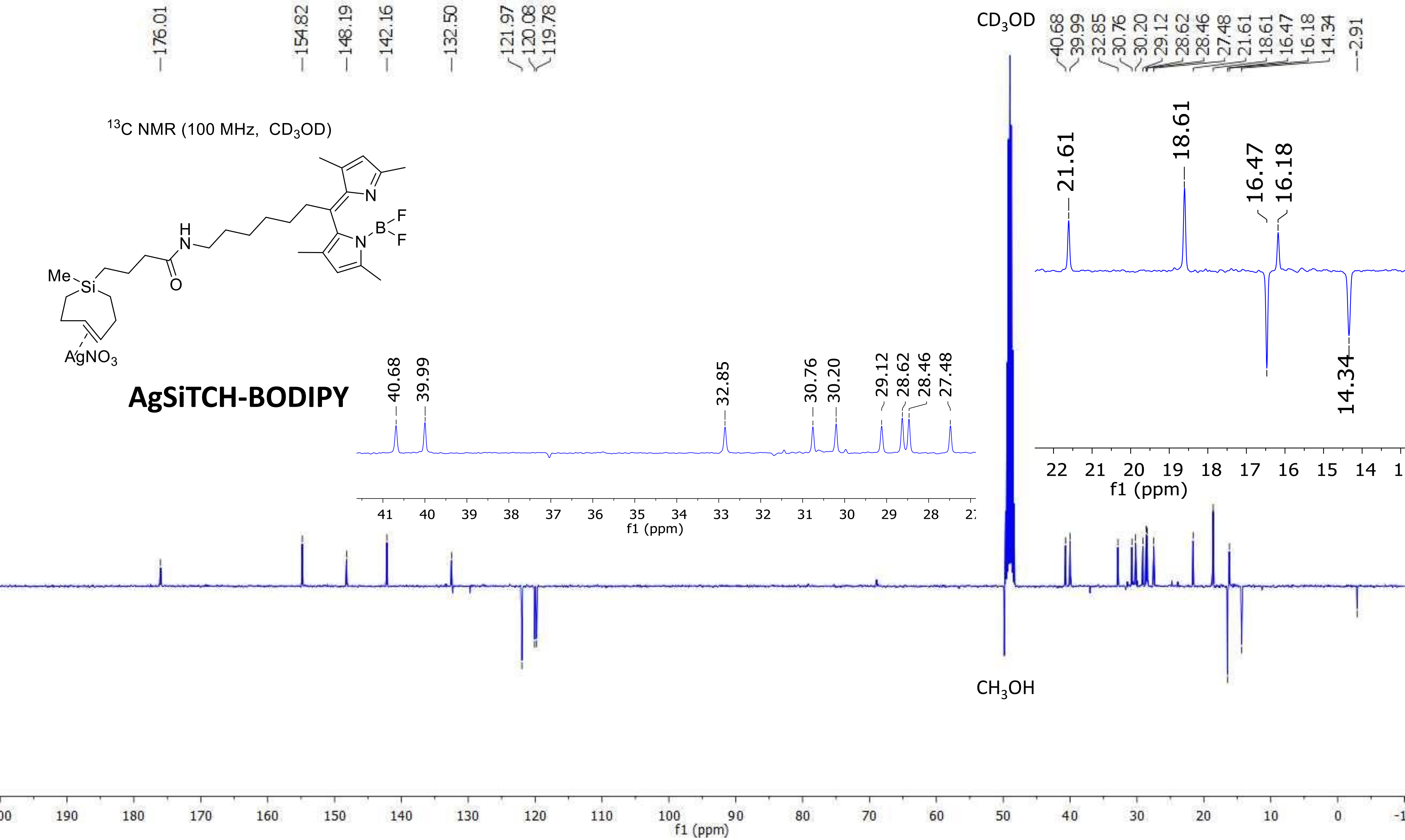

13C NMR (100 MHz, CD3OD)
AgSiTCH-BODIPY
Me
Si
AgNO3
H
N
O
N
B
F
F
N
CD3OD
CH3OH
176.01
154.82
148.19
142.16
132.50
121.97
120.08
119.78
40.68
39.99
32.85
30.76
30.20
29.12
28.62
28.46
27.48
21.61
18.61
16.47
16.18
14.34
-2.91
f1 (ppm)

^{1}H NMR (400 MHz, CD_3OD)

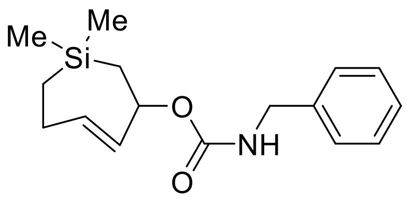

18

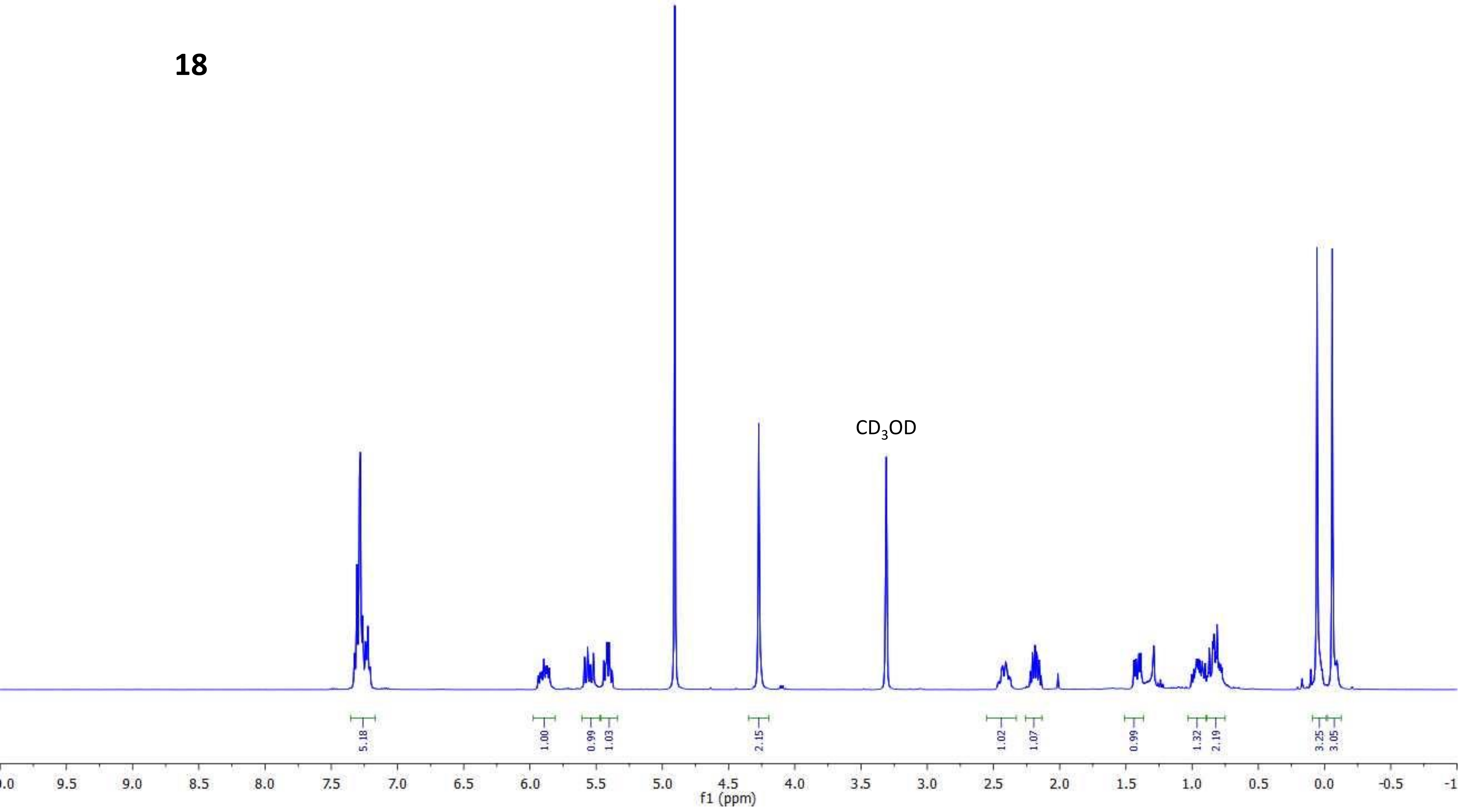

^{13}C NMR (100 MHz, CD_3OD)

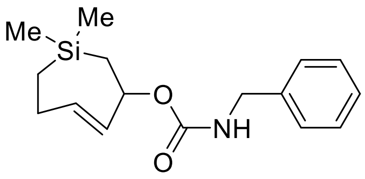

18

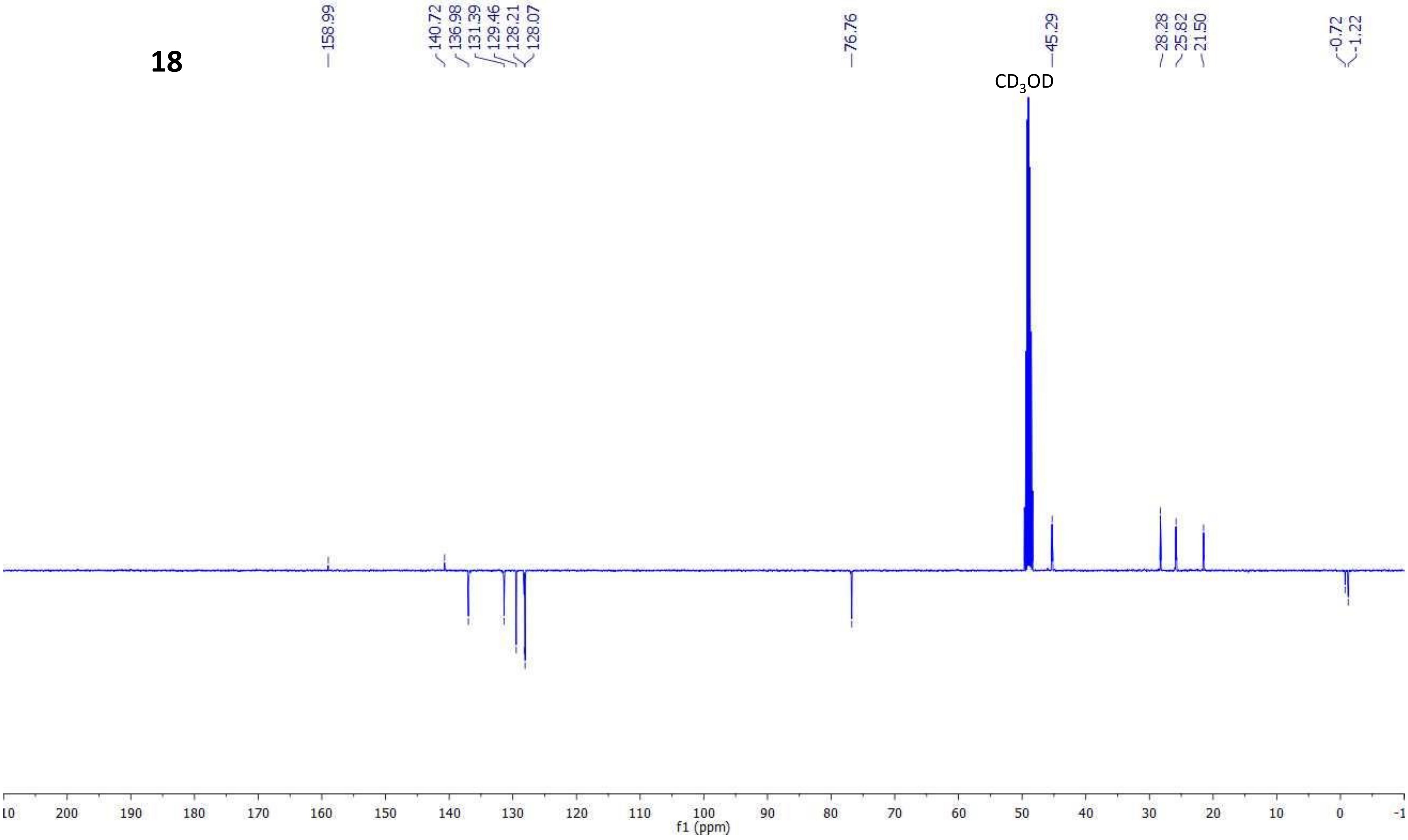

Supplement: Supplementary file 1 [file SC-009-C7SC04773H-s001.pdf]
