# Supplementary material for: Photochemical syntheses, transformations, and bioorthogonal chemistry of trans-cycloheptene and sila trans-cycloheptene Ag(i) complexes
Source: Chem Sci. 2018 Jan 8;9(7):1953–63. doi: 10.1039/c7sc04773h (PMC5892336; doi:10.1039/c7sc04773h)
Supplement: Supplementary file 2 [file SC-009-C7SC04773H-s002.pdf]

Electronic Supplementary Material (ESI) for Chemical Science.
This journal is © The Royal Society of Chemistry 2018

Supporting Information

Photochemical Syntheses, Transformations and Bioorthogonal Chemistry of *trans*-Cycloheptene and Sila *trans*-Cycloheptene Ag(I) Complexes

Yinzhi Fang,[a] Han Zhang,[a,#] Zhen Huang,[b,#] Samuel S. L. Scinto,[a] Jeffrey C. Yang,[b] Christopher W. Am Ende,[c] Olga Dmitrenko,[a] Douglas S. Johnson,[b] Joseph M. Fox[a,*]

[a] Brown Laboratories, Department of Chemistry and Biochemistry, University of Delaware, Newark, DE 19716

[b] Pfizer Worldwide Research and Development, Cambridge, Massachusetts 02139

[c] Pfizer Worldwide Research and Development, Groton, Connecticut 06340

Table of contents

S1. General Considerations **3**

S2. Preparation of Silver Nitrate Impregnated Silica Gel **3**

S3. Photoisomerization Apparatus for carbocyclic *trans*-cycloheptenes **3-5**

S4. Photoisomerization Apparatus for sila *trans*-cycloheptenes **6-7**

S5. Synthesis procedure for *cis*-cycloheptene substrates **7-20**

S6. Synthesis procedure for carbocyclic *trans*-cycloheptenes **20-21**

S7. General procedure for photoisomerization of silicon-containing *trans*-cycloheptene derivatives **22-29**

S8. Reactions of *trans*-cycloheptene•$AgNO_3$ complexes and Si-TCH **30-34**

S9. Transformations of Si-TCH derivatives **35-38**

S10. Kinetic measurements **38-43**

S11. General Method for in vitro Si-TCH / GFP-Tet-v.2.0 kinetics **44-45**

S12. General Method for in vivo Si-TCH / GFP-Tet-v.2.0 kinetics **45-49**

S13. IR and NMR comparisons of Si-TCH and Si-TCH silver complex **50-51**

S14. X-ray data **52-77**

S15. Computational studies **77-88**

S16. Cellular experiments **89-94**

S17. Stability Experiments **94-95**

S17. References **96**

Experimental Section

General Considerations

Anhydrous methylene chloride was dried through a column of alumina using a solvent purification system. Anhydrous THF was freshly distilled from Na^0/benzophenone ketyl. Commercial HMPA was dried over 4Å molecular sieves. All other reagents were purchased from commercial sources and used without further purification. Chromatography was performed on normal phase silica gel from Silicycle (40-63 μm, 230-400 mesh). All of the conversion of photoisomerization reactions were monitored by GC (GC-2010 Plus, Shimadzu). APT and CPD pulse sequences were used for ^{13}C NMR. When the APT pulse sequence was used for ^{13}C NMR, the secondary and quaternary carbons were phased to appear 'up' (u), and tertiary and primary carbons appear 'down' (dn). Data are represented as follows: chemical shift, multiplicity (br = broad, s = singlet, d = doublet, t = triplet, q = quartet, m = multiplet, dd = doublet of doublets). Stopped-Flow kinetics were measured using an SX18MV-R stopped-flow spectrophotometer (Applied Photophysics Ltd.) with temperature control. High resolution mass spectral data were taken with a Waters GCT Premier high-resolution time-of-flight mass spectrometer, or using a Thermo Q-Exactive Orbitrap instrument. Protein MS were conducted using a Waters Xevo G2-S QTof. Infrared (IR) spectra were obtained using FTIR spectrophotometers with films cast onto a NaCl, KBr or AgCl plate. Infrared (IR) spectra for compound **2a, 2b, 2c, 2d, 2d** (metal free)**, 2e,** and **2f** were collected from Bruker Tensor 27 and Nexus™ 670 FT-IR. Tetrazine-TAMRA conjugate **23** was prepared according to literature procedure.[7]

Preparation of Silver Nitrate Impregnated Silica Gel[1]

Flash silica gel (90 g, Silicycle cat # R12030B, 60 Å) was suspended in 100 mL of water in a 2 L round bottomed flask. The flask was covered with aluminum foil and a silver nitrate (10 g) solution in water (10 mL) was added. The resulting mixture was thoroughly mixed. Water was evaporated under reduced pressure on the rotavap (bath temperature ~ 85 °C) using a bump trap with a coarse fritted disk. To remove the remaining traces of water, toluene (2 x 200 mL) was added and subsequently evaporated by rotary evaporation. The silver nitrate impregnated silica was then dried under high vacuum overnight at rt.

Photoisomerization Apparatus for carbocyclic *trans*-cycloheptenes

Photoisomerizations were carried out using a Southern New England Ultraviolet Company Rayonet® reactor model RPR-100 or RPR-200, equipped with 8 low-pressure mercury lamps (2537 Å). Photoisomerizations were carried out in a PTFE tubing (also named FEP tubing, 1/8" OD x 0.063" ID, 14.9 m in length, flanged with a thermoelectric flanging tool), which was wrapped around a test tube rack (8.0 cm ×10.0 cm× 24.0cm). Biotage® SNAP cartriges (Biotage part No. FSK0-1107) were used to house silica gel and the $AgNO_3$-impregnated silica gel. The PTFE tubing was connected to the Biotage® SNAP cartriges and to a pump at the other end. The pump was purchased from Fluid Metering, Inc. (FMI pump model RP-D equipped with pump head FMI R405). An inline thermometer was connected between the FMI pump and the FEP tubing. A long-necked round bottomed flask was connected to the FMI pump and the Biotage®

SNAP cartriges via PTFE tubing. The round bottomed flask was settled into a cryobath (Thermo Scientific NESLAB CB-80 cryobath). The temperature of cryobath was set to –50 °C when using the photoapparatus.

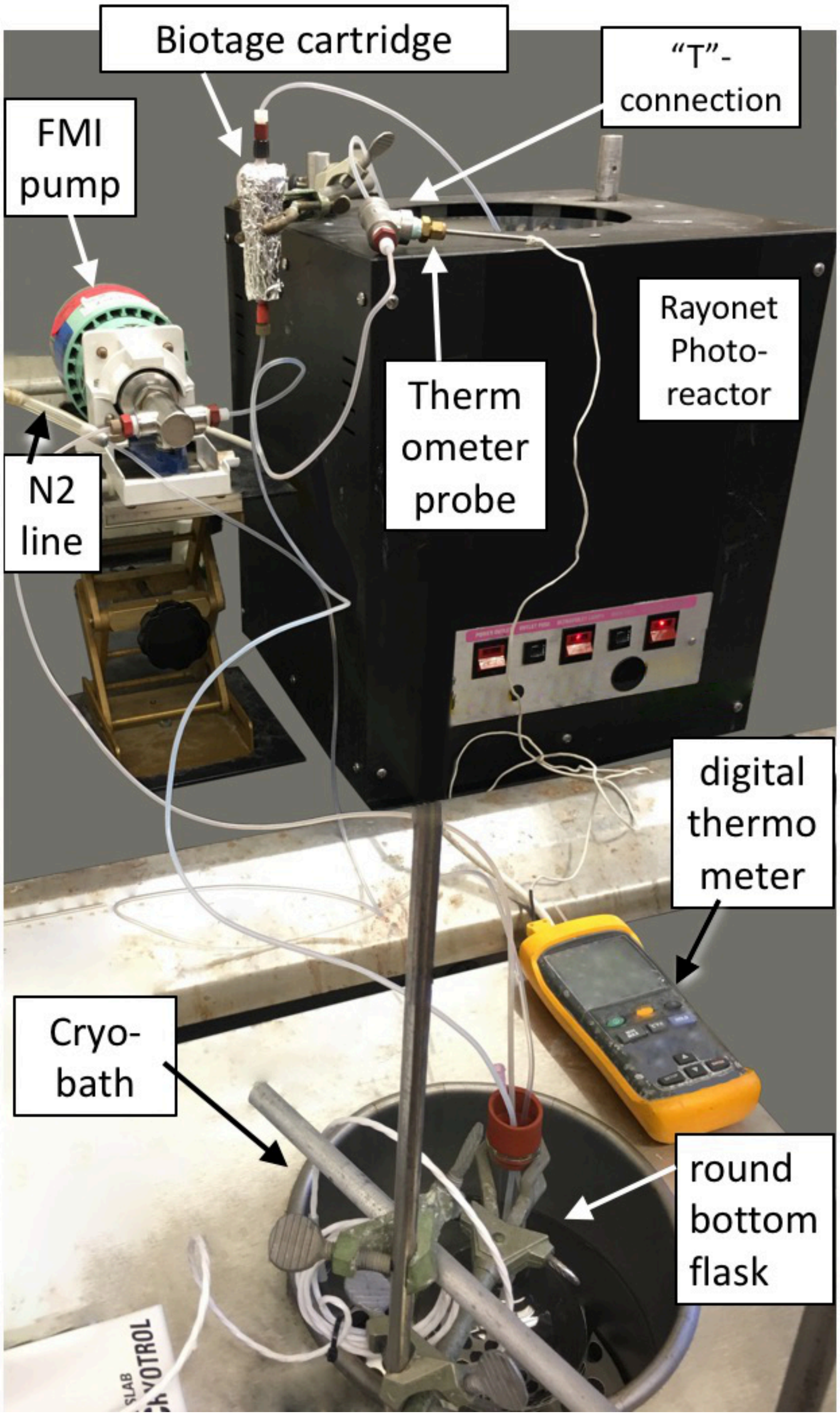

Fig.S1 Setup for the general photosynthesis of carbocyclic *trans*-cycloheptene

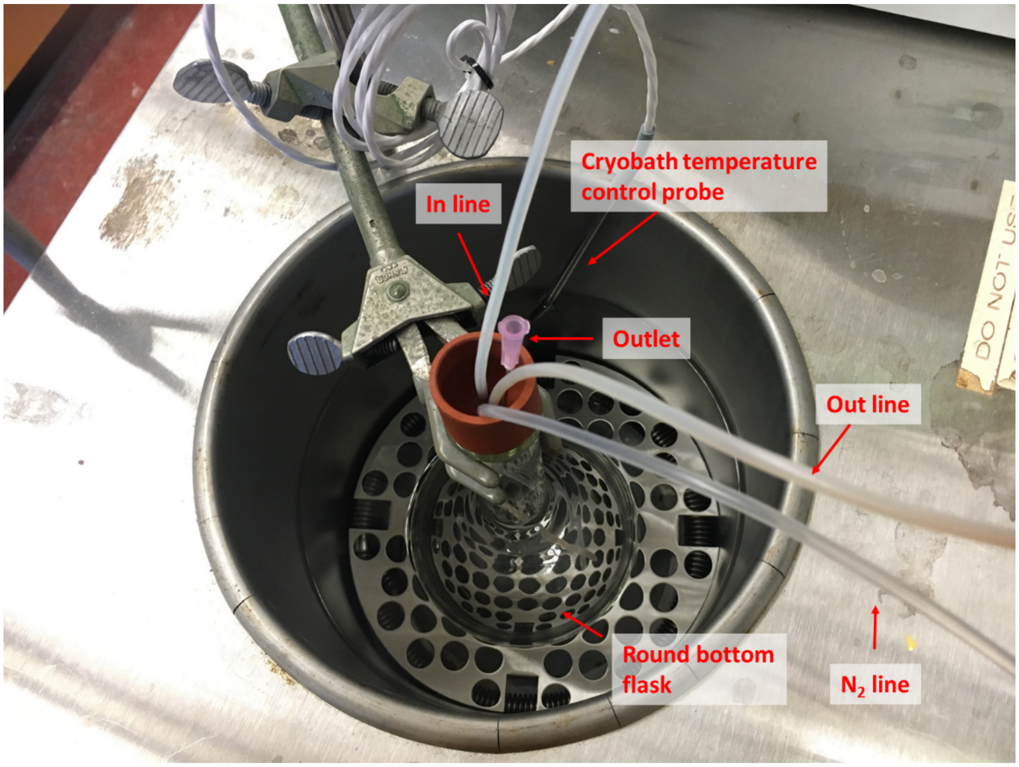

Fig.S2 Cryobath setup

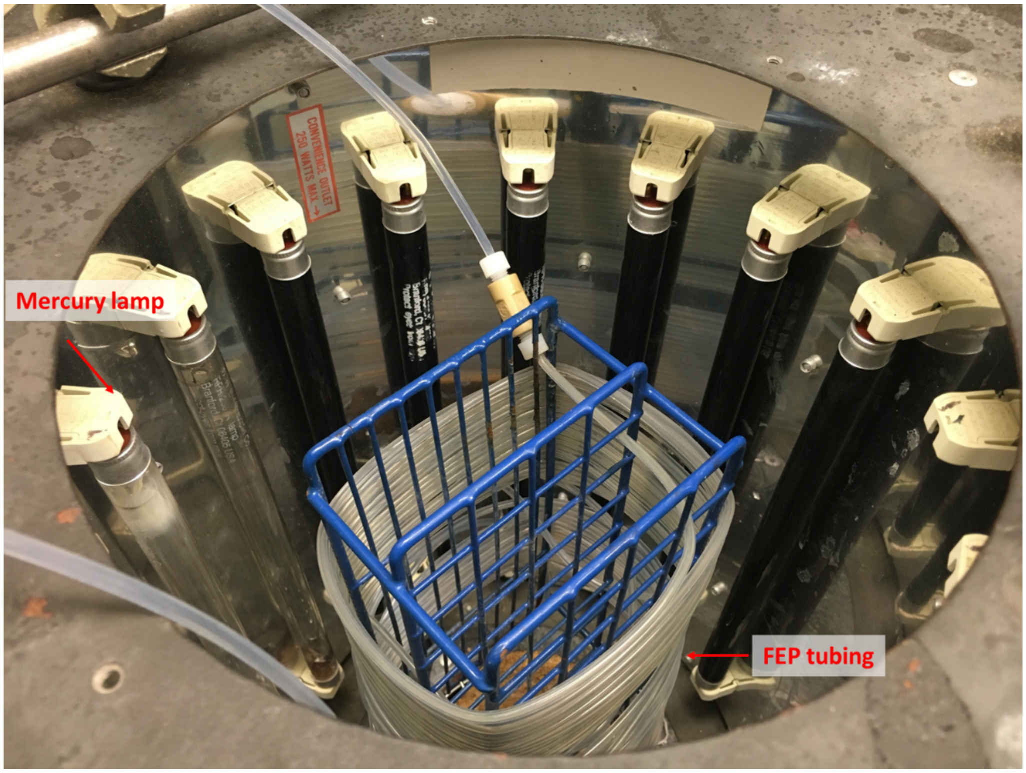

Fig.S3. Inside of Rayonet® reactor

Photoisomerization Apparatus for sila *trans*-cycloheptenes

Photoisomerizations were carried out using a Southern New England Ultraviolet Company Rayonet® reactor model RPR-100 or RPR-200, equipped with 8 low-pressure mercury lamps (2537 Å). Photoisomerizations were carried out in a quartz flask (Southern New England Ultraviolet Company). Biotage® SNAP cartriges (Biotage part No. FSK0-1107) were used to house silica gel and the $AgNO_3$-impregnated silica gel. The bottom of the column was interfaced to PTFE tubing (1/8" OD x 0.063" ID, flanged with a thermoelectric flanging tool), equipped with flangeless nylon fittings (1/4-28 thread, IDEX part no. P-582), using a female luer (1/4-28 thread, IDEX part no. P-628). The top of the column was interfaced using a male luer (1/4-28 thread, IDEX part no. P-675). Pump used for recirculating solvents through the photoreactor was purchased from Fluid Metering, Inc. (FMI pump model RP-D equipped with pump head FMI R405). Adapters for interfacing the FMI pump to the PTFE tubing were purchased from IDEX (part no. U-510).

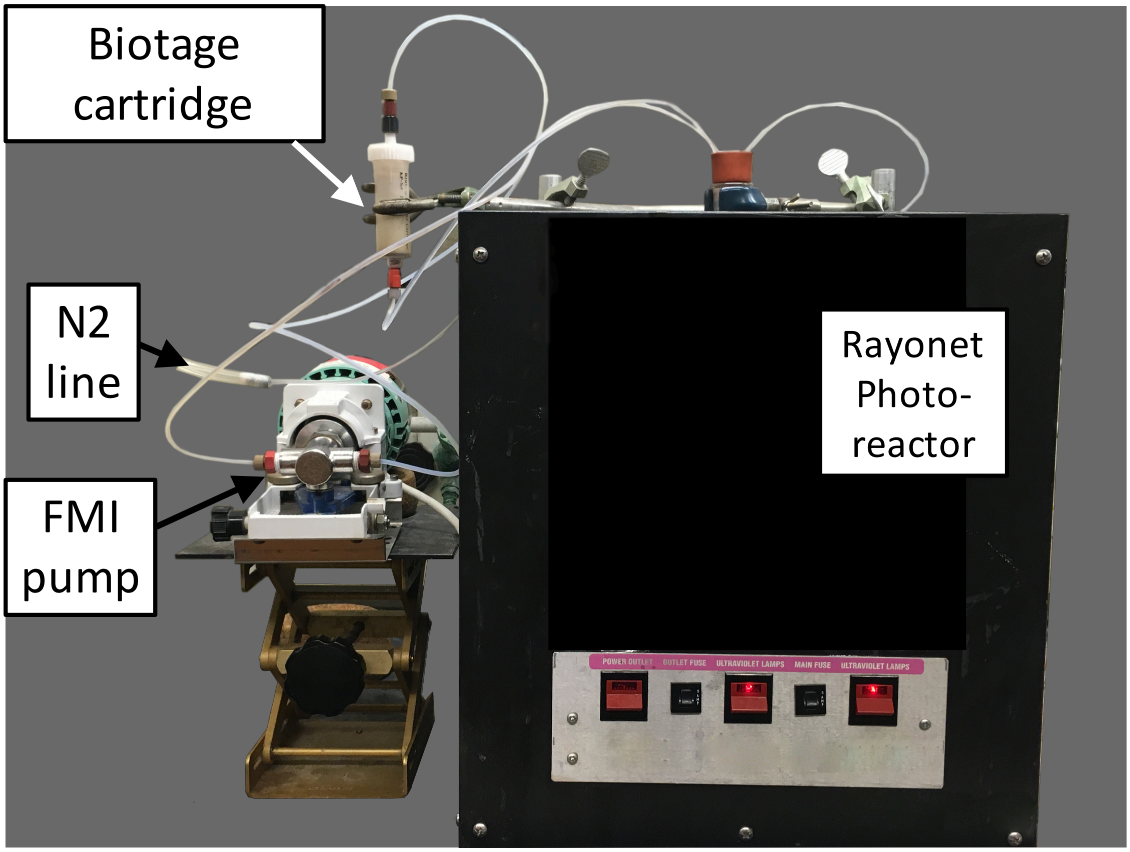

Fig.S4 Setup for the general photosynthesis of sila *trans*-cycloheptenes

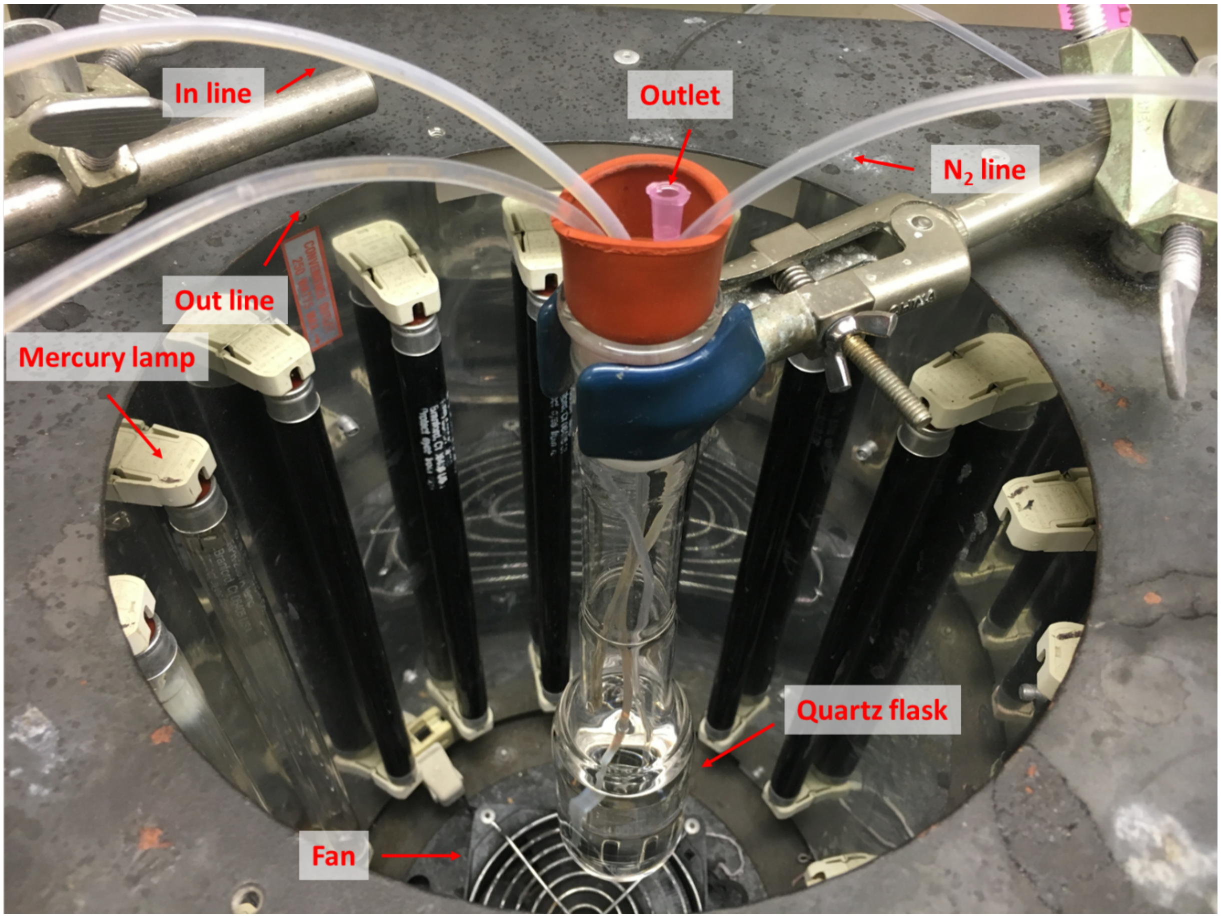

Fig. S5. Inside of Rayonet® reactor

Synthesis procedure for *cis*-cycloheptene substrates:

2-(But-3-en-1-yl)hex-5-en-1-ol

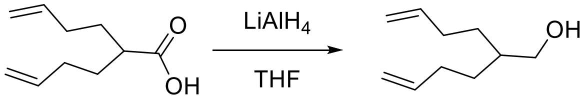

A dry two-neck round-bottomed flask equipped with a reflux condenser was sequentially charged with anhydrous THF (5.30 mL) and $LiAlH_4$ (271 mg, 7.14 mmol, 2.00 equiv). The mixture was chilled by an ice bath, and a solution of 2-(but-3-en-1-yl) hex-5-enoic acid[2] (600 mg, 3.57 mmol, 1.00 equiv) in anhydrous THF (5.30 mL) was added dropwise with a syringe. The reaction mixture was then heated to reflux overnight. The reaction mixture was then allowed to cool to rt and then to ice bath temperature, and 20 mL of 15% NaOH solution was added dropwise. The resulting mixture was filtered and the filter cake was washed with ethyl acetate (20 mL). The aqueous phase was extracted with ethyl acetate (3 × 20 mL). The resulting solution was dried with Na_2SO_4, filtered and concentrated on the rotary evaporator. Purification by

column chromatography with 5% ethyl acetate in hexanes yielded 2-(but-3-en-1-yl)hex-5-en-1-ol (431 mg, 2.80 mmol, 78%) as a clear oil. ^{1}H NMR (600 MHz, $CDCl_3$) δ: 5.82 (ddt, J = 16.9, 10.2, 6.6 Hz, 2H), 5.04-5.01 (m, 2H), 4.97-4.95 (m, 2H), 3.58 (d, J = 5.3 Hz, 2H), 2.09 (dt, J = 14.4, 7.2 Hz, 4H), 1.57-1.53 (m, 1H), 1.50-1.44 (m, 2H), 1.43-1.37 (m, 2H), 1.35 (s, 1H); ^{13}C NMR (100 MHz, $CDCl_3$) δ:139.0 (dn), 114.6 (u), 65.3 (u), 39.5 (dn), 31.2 (u), 30.2 (u); FTIR (KBr/thin film) 2925, 1641, 1384, 1050, 908, 668 cm^{-1}; HRMS (CI+) *m/z*: $[M+H]^+$ calcd. for $C_{10}H_{19}O^+$, 155.1436 found 155.1420.

(4*Z*)-Cyclohept-4-en-1-ylmethanol

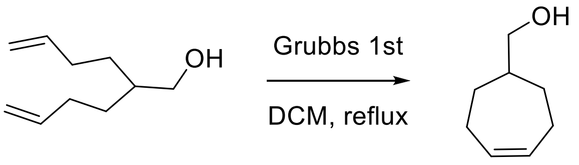

A 2 L round-bottomed flask equipped with a reflux condenser was charged with 1 L of methylene chloride, followed by addition of 2-(but-3-en-1-yl)hex-5-en-1-ol (1.00 g, 6.49 mmol, 1.00 equiv). The solution was heated to reflux temperature. Grubbs 1st generation catalyst (267 mg, 0.324 mmol, 0.05 equiv) was added. The reaction mixture was allowed to reflux for 3 hours. The solvent was removed with a rotary evaporator. Purification by column chromatography with 8% ethyl acetate in hexanes yielded 430 mg (3.41 mmol, 54%) of the title compound as a green oil. ^{1}H NMR (400 MHz, $CDCl_3$) δ: 5.79-5.72 (m, 2H), 3.42 (d, J = 6.5 Hz, 2H), 2.29-2.17 (m, 3H), 2.07-2.00 (m, 2H), 1.80-1.75 (m, 2H), 1.72-1.64 (m, 1H), 1.15-1.06 (m, 2H); ^{13}C NMR (100 MHz, $CDCl_3$) δ: 132.1 (dn), 68.2 (u), 44.9 (dn), 29.6 (u), 26.9 (u); FTIR (KBr/thin film) 3323, 3018, 2916, 2840, 1442, 1250, 1067, 1044, 1008, 939, 703, 631 cm^{-1}; HRMS (CI+) *m/z*: $[M+H]^+$ calcd. for $C_8H_{15}O^+$, 127.1123 found 127.1105.

4-(Di(but-3-en-1-yl)(methyl)silyl)butanenitrile

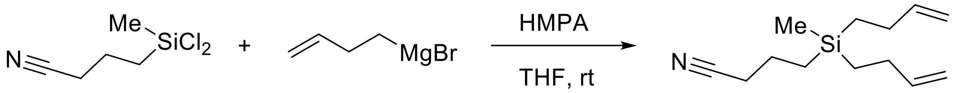

A dry round-bottomed flask was charged with Mg powder (1.24 g, 51.7 mmol, 3.00 equiv) and dry THF (125 mL) under nitrogen atmosphere. 4-Bromo-1-butene (5.60 mL, 55.2 mmol, 3.21 equiv) was introduced to the flask dropwise via syringe. The reaction mixture was allowed to stir at rt. After magnesium powder was consumed and the formation of the Grignard reagent was complete, HMPA (15.0 mL, 86.0 mmol, 5.00 equiv, dried over 4Å molecular sieve) was added, followed by 4-(dichloro(methyl)silyl)butanenitrile (2.70 mL, 17.2 mmol, 1.00 equiv). The reaction mixture was stirred at rt overnight. Afterwards, THF was removed via rotary evaporation. Saturated aq. NH_4Cl (80 mL) and ethyl acetate (80 mL) were added and the aqueous layer was extracted three times with ethyl acetate. The organics were combined, dried with anhydrous $MgSO_4$, filtered, and concentrated via rotary evaporation. Purification by flash column chromatography (1% diethyl ether/hexane) afforded the title compound as colorless oil (2.14 g, 9.66 mmol, 56% yield). ^{1}H NMR (600 MHz, $CDCl_3$) δ: 5.86 (ddt, J=16.5, 10.1, 6.3 Hz, 2H), 5.00 (dd, J=17.0, 1.7 Hz, 2H), 4.91 (dd, J=10.1, 1.3 Hz, 2H), 2.36 (t, J=6.9 Hz, 2H), 2.05

(ddd, *J*=9.9, 8.8, 6.4 Hz, 4H), 1.68-1.63 (m, 2H), 0.71-0.68 (m, 2H), 0.67-0.64 (m, 4H) , 0.01 (s, 3H); ^{13}C NMR (150 MHz, $CDCl_3$) δ:141.3, 119.8, 113.2, 27.9, 21.1, 20.7, 13.8, 12.8, -5.2; FTIR (KBr/thin film) 3077, 2975, 2908, 2245, 1714, 1639, 1414, 1253, 1175, 1080, 994, 906, 805 cm^{-1}; HRMS (CI+) *m/z*: $[M+H]^+$ calcd. for $C_{13}H_{24}NSi^+$, 222.1678 found 222.1697.

(*Z*)-*Si*-(3-Cyanopropyl)-*Si*-methyl-5-sila-cycloheptene

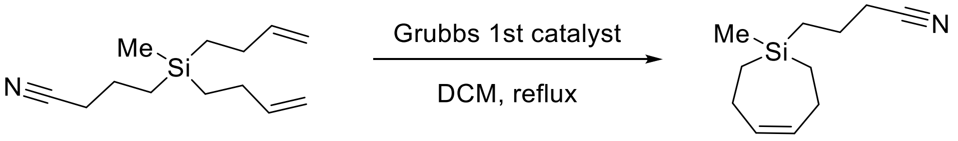

4-(Di(but-3-en-1-yl)(methyl)silyl)butanenitrile (400 mg, 1.81 mmol, 1.00 equiv) was dissolved in dry CH_2Cl_2 (120 mL). Grubbs' 1st generation catalyst (74 mg, 0.0903 mmol, 0.0500 equiv) was added as a solution in dry CH_2Cl_2 (37 mL) and the solution was heated to reflux for 5 hours. After cooling to rt, the reaction mixture was concentrated via rotary evaporation. Purification by flash column chromatography (2% diethyl ether/hexane) afforded the title compound (299 mg, 1.55 mmol, 85% yield) as colorless oil. ^{1}H NMR (400 MHz, $CDCl_3$) δ: 5.80-5.72 (m, 2H), 2.35 (t, *J* =7.0 Hz, 2H), 2.24-2.19 (m, 4H), 1.70-1.62 (m, 2H), 0.72-0.67 (m, 2H), 0.65-0.61 (m, 4H), 0.03 (s, 3H); ^{13}C NMR (100 MHz, $CDCl_3$) δ: 132.6, 119.9, 21.05, 21.02, 20. 5, 14.7, 12.5, -4.0; FTIR (KBr/thin film) 3016, 2909, 2876, 2855, 2245, 1466, 1425, 1406, 1252, 1170, 933, 795, 699 cm^{-1}; HRMS (LIFDI-TOF) *m/z*: $[M]^+$ calcd. for $C_{11}H_{19}NSi^+$, 193.1281 found 193.1282.

(*Z*)-*Si*-(4-Oxobutyl)-*Si*-methyl-5-silacycloheptene

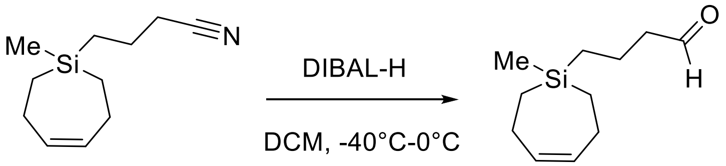

A dry round-bottomed flask was charged with a solution of (*Z*)-*Si*-(3-cyanopropyl)-*Si*-methyl-5-silacycloheptene (656 mg, 3.39 mmol, 1.00 equiv) in CH_2Cl_2 (4.5 mL) under an atmosphere of nitrogen. The flask was cooled by a bath of dry ice/acetone (–78 °C), and DIBAL-H (4.1 mL of a 1.0 M solution in CH_2Cl_2, 4.1 mmol, 1.2 equiv) was slowly added via syringe. The dry ice/acetone bath was then replaced with a –40 °C bath (dry ice/acetonitrile), and stirring was continued for 1 hour. The cold bath was then replaced by an ice bath (0 °C). At 0 °C, H_2O (0.14 mL) and 15% NaOH (0.14 mL) were sequentially added dropwise. Additional water (0.34 mL) was added, and the ice bath was removed and the mixture allowed to stir for 15 min at rt. Some anhydrous magnesium sulfate was added and the mixture was stirred for another 15 min. The salts formed in the mixture were filtered via a Büchner funnel and rinsed with methylene chloride. The methylene chloride solutions were combined and concentrated. Purification by flash column chromatography (2% diethyl ether/hexane) afforded the title compound (422 mg, 2.15 mmol, 63% yield) as colorless oil. ^{1}H NMR (400 MHz, $CDCl_3$) δ: 9.75 (t, *J* =1.8 Hz, 1H),

5.80-5.72 (m, 2H), 2.45 (td, J =7.2, 1.8 Hz, 2H), 2.23-2.18 (m, 4H), 1.68-1.60 (m, 2H), 0.64-0.54 (m, 6H), 0.02 (s, 3H); ^{13}C NMR (100 MHz, $CDCl_3$) δ: 203.2 (dn), 132.7 (dn), 47.7 (u), 21.1 (u), 16.7 (u), 14.8 (u), 12.6 (u), -4.0 (dn); FTIR (KBr/thin film) 2910, 2876, 2855, 1727, 1466, 1407, 1251, 1169, 1153, 934, 795, 697 cm^{-1}; HRMS (LIFDI-TOF) *m/z*: $[M]^+$ calcd. for $C_{11}H_{20}OSi^+$, 196.1283 found 196.1301.

(*Z*) 4-(1-methyl-2,3,6,7-tetrahydro-1H-silepin-1-yl)butanoic acid

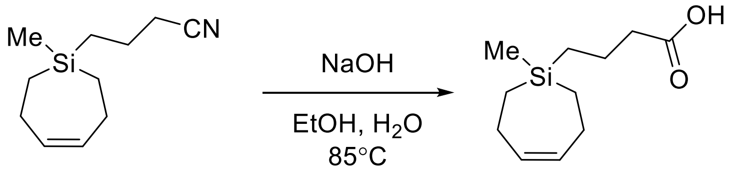

A round-bottomed flask was charged with a solution of (*Z*)-*Si*-(3-cyanopropyl)-*Si*-methyl-5-silacycloheptene (225 mg, 1.16 mmol, 1.00 equiv) in ethanol (11.6 mL). NaOH solution (2.32 g in 2.32 mL H_2O, 58.0 mmol, 50.0 equiv) was slowly added. The reaction was then refluxed for 4 hours. Afterwards, ethanol was removed by rotary evaporation, and the remainder was acidified to pH~2 with 3.0 M HCl solution. The mixture was extracted with 3×40 mL diethyl ether. The combined organics were dried with anhydrous $MgSO_4$, filtered, and concentrated. Purification by flash column chromatography (30% ethyl acetate/hexane) afforded the title compound (188 mg, 0.89 mmol, 76% yield) as light yellow oil. ^{1}H NMR (400 MHz, $CDCl_3$) δ: 5.80-5.72 (m, 2H), 2.37 (t, J =7.4 Hz, 2H), 2.26-2.16 (m, 4H), 1.67-1.62 (m, 2H), 0.71-0.56 (m, 6H), 0.02 (s, 3H); ^{13}C NMR (100 MHz, $CDCl_3$) δ: 180.6 (u), 132.7 (dn), 38.0 (u), 21.1 (u), 19.4 (u), 14.7 (u), 12.6 (u), -4.0 (dn); FTIR (KBr/thin film) 3017, 2909, 1709, 1410, 1292, 1251, 1232, 1169, 933, 794, 698 cm^{-1}; HRMS (LIFDI-TOF) *m/z*: $[M]^+$ calcd. for $C_{11}H_{20}O_2Si^+$ 212.1233, found 212.1231.

(*Z*)-*Si*-(4-Hydroxybutyl)-*Si*-methyl-5-silacycloheptene

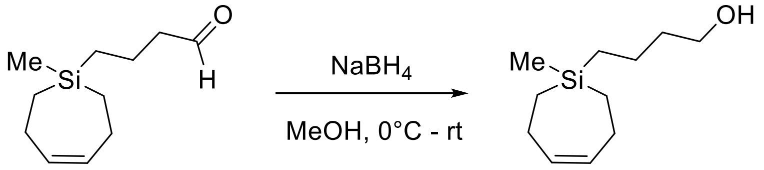

A 25 mL round-bottomed flask was charged with (*Z*)-*Si*-(4-Oxobutyl)-*Si*-methyl-5-silacycloheptene (422 mg, 2.15 mmol, 1.00 equiv) and methanol (11 mL). The flask was cooled by an ice bath (0 °C), and the mixture was magnetically stirred. Sodium borohydride (81 mg, 2.15 mmol, 1.00 equiv) was added slowly in small portions as a solid to the reaction mixture. The ice bath was removed, and the mixture was allowed to stir while warming to room temperature for 1 h. Water (3 mL) and 3M HCl (3 mL) were sequentially and cautiously added dropwise to the mixture. Methanol was removed by rotary evaporation, and the remainder was thrice extracted with diethyl ether. The combined organics were dried with anhydrous Na_2SO_4,

filtered, and concentrated. Purification by flash column chromatography (10%-20% diethyl ether/hexane) afforded the title compound (403 mg, 2.03 mmol, 95% yield) as colorless oil. ^{1}H NMR (400 MHz, $CDCl_3$) δ: 5.80-5.73 (m, 2H), 3.65 (t, J =6.4 Hz, 2H), 2.23-2.19 (m, 4H), 1.63-1.56 (m, 2H), 1.41-1.33 (m, 2H), 1.24 (br s, 1H), 0.64-0.53 (m, 6H), 0.01 (s, 3H); ^{13}C NMR (150 MHz, $CDCl_3$) δ: 132.7 (dn), 62.8 (u), 36.8 (u), 21.2 (u), 20.0 (u), 14.8 (u), 12.8 (u), -3.9 (dn); FTIR (KBr/thin film) 3323, 3016, 2909, 2874, 1647, 1406, 1378, 1250, 1169, 1060, 934, 795, 698, 435 cm^{-1}; HRMS (CI+) *m/z*: $[M+H]^+$ calcd. for $C_{11}H_{23}OSi^+$ 199.1518, found 199.1522.

Di(but-3-en-1-yl)diphenylsilane

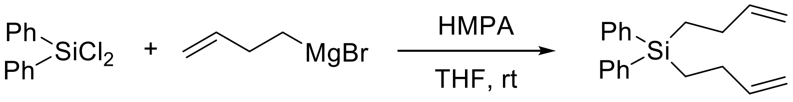

A flame-dried round-bottomed flask was charged with Mg powder (2.85 g, 119 mmol, 3.50 equiv) and dry THF (200 mL) under nitrogen atmosphere. 4-Bromo-l-butene (12.3 mL, 121 mmol, 3.56 equiv) was introduced to the flask dropwise via syringe. The reaction mixture was allowed to stir at rt. After magnesium powder was consumed and the formation of the Grignard reagent was complete, HMPA (29.6 mL, 170 mmol, 5.00 equiv, dried over 4Å molecular sieve) was added, followed by dichlorodiphenylsilane (7.15 mL, 34.0 mmol, 1.00 equiv). The reaction mixture was stirred at rt overnight. Afterwards, THF was removed via rotary evaporation. Saturated aq. NH_4Cl (100 mL) and ethyl acetate (100 mL) were added and the aqueous layer was extracted three times with ethyl acetate. The organics were combined, dried with anhydrous $MgSO_4$, filtered, and concentrated via rotary evaporation. Purification by flash column chromatography (hexane) afforded the title compound as colorless oil (7.03 g, 24.0 mmol, 71%). ^{1}H NMR (600 MHz, $CDCl_3$) δ: 7.54-7.49 (m, 4H), 7.43-7.33 (m, 6H), 5.95-5.83 (m, 2H), 5.03-4.82 (m, 4H), 2.15-2.06 (m, 4H), 1.25-1.17 (m, 4H); ^{13}C NMR (100 MHz, $CDCl_3$) δ: 141.4 (dn), 135.8 (u), 135.0 (dn), 129.4 (dn), 128.0 (dn), 113.1 (u), 27.9 (u), 11.8 (u) ; FTIR (NaCl/thin film) 3068, 2998, 2976, 2907, 1638, 1427, 1180, 1110, 997, 900, 738, 700, 484 cm^{-1}; HRMS (LIFDI-TOF) *m/z*: $[M]^+$ calculated for $C_{20}H_{24}Si^+$ 292.1642; Found 292.1659.

(*Z*)-Si,-Si-diphenyl-5-sila-cycloheptene

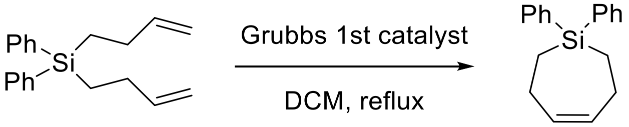

Di(but-3-en-1-yl)diphenylsilane (7.03 g, 24.0 mmol, 1.00 equiv) was dissolved in CH_2Cl_2 (120 mL). Grubbs 1st generation catalyst (594 mg, 0.722 mmol, 0.030 equiv) was added as a solution in CH_2Cl_2 (1.7 L) and the mixture was refluxed under nitrogen for 1 hour. The mixture was cooled to rt, and the reaction mixture was concentrated via rotary evaporation. Purification by flash column chromatography (hexane) afforded the title compound (4.50 g, 71% yield) as colorless solid, mp 59 °C. ^{1}H NMR (600 MHz, $CDCl_3$) δ: 7.56-7.50 (m, 4H), 7.41-7.32 (m, 6H), 5.90-5.82 (m, 2H), 2.40-2.30 (m, 4H), 1.29-1.22 (m, 4H); ^{13}C NMR (100 MHz, $CDCl_3$) δ: 137.2 (u), 134.7 (dn), 132.7 (dn), 129.3 (dn), 128.0 (dn), 21.1 (u), 11.4 (u); FTIR (KBr/thin film)

3067, 3017, 2910, 2856, 1427, 1149, 1115, 931, 787, 698, 515 cm^{-1}; HRMS (LIFDI-TOF) *m/z*: $[M]^+$ calculated for $C_{18}H_{20}Si^+$ 264.1329; Found 264.1338.

(3-Chloropropyl)(methyl)bis(2-methylallyl)silane

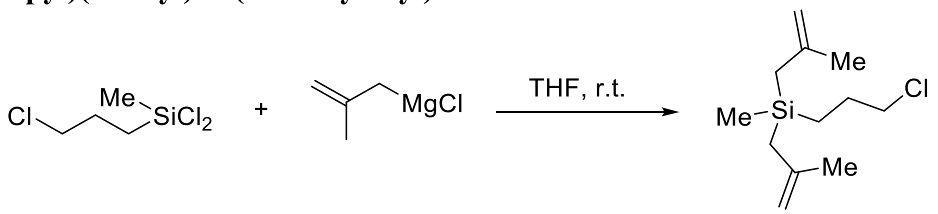

3-Methallylmagnesium chloride solution (12.5 mmol, 0.5M in THF, 2.50 equiv) was added to a flask that had been flame dried and cooled under nitrogen. 3-Chloropropyldichloromethylsilane (958 mg, 5.0 mmol, 1.00 equiv) was added dropwise. The reaction was stirred for 6 hours at rt. Afterwards, THF was removed via rotary evaporation. Saturated NH_4Cl aqueous solution and diethyl ether were added, and aqueous layer was extracted with diethyl ether for 3 times. The combined organic layers were dried with anhydrous $MgSO_4$, filtered, and concentrated via rotary evaporation. Purification by flash column chromatography (hexane) afforded the desired (3-chloropropyl)(methyl)bis(2-methylallyl)silane as colorless oil (950 mg, 83% yield). ^1H NMR (400 MHz, $CDCl_3$) δ: 4.62 (s, 2H), 4.51 (s, 2H), 3.50 (t, J = 6.9 Hz, 2H), 1.83 – 1.75 (m, 2H), 1.72 (s, 6H), 1.59 (s, 4H), 0.71 – 0.67 (m, 2H), 0.07 (s, 3H); ^{13}C NMR (100 MHz, $CDCl_3$) δ: 143.2 (u), 109.2 (u), 48.1 (u), 27.6 (u), 25.7 (u), 25.5 (dn), 11.8 (u), -4.3 (dn); FTIR (KBr /thin film) 3074, 2954, 2914, 1637, 1440, 1373, 1279, 1252, 1164, 1000, 872, 840 cm^{-1}; HRMS (CI+) *m/z*: $[M-CH_3]^+$ calculated for $C_{11}H_{20}ClSi^+$ 215.1017; Found 215.1022.

3,3'-((3-Chloropropyl)(methyl)silanediyl)bis(2-methylpropan-1-ol)

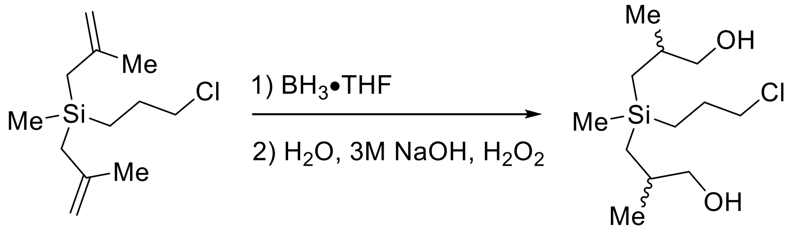

A 25 mL flame-dried round-bottomed flask was charged with (3-chloropropyl)(methyl)bis(2-methylallyl)silane (231 mg, 1.00 mmol, 1.00 equiv) and 0.500 mL dry THF. The flask was cooled by an ice bath (0 °C). Borane THF complex solution (1.0 M in THF) (2.00 mL, 2.00 mmol, 2.00 equiv) was added dropwise to the reaction mixture. The ice bath was removed, and the mixture was allowed to stir while warming to room temperature for 2 hours. Then 2 mL H_2O was added to the mixture, followed by 0.48 mL 3M NaOH solution and 0.48 mL 30% H_2O_2. The mixture was stirred at 35 °C for 4 h. Afterwards, 10 mL H_2O and 10 mL ethyl acetate were added, and the aqueous layer was saturated with K_2CO_3 and extracted with ethyl acetate 3 times. The combined organic layer were dried with anhydrous $MgSO_4$, filtered, and concentrated via rotary evaporation. Purification by flash column chromatography (20%-30% Ethyl acetate/hexane) afforded the title compound as colorless oil (236 mg, 88% yield).

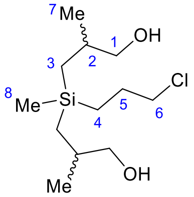

^{1}H NMR (600 MHz, $CDCl_3$) δ: 3.49 (t, *J* = 6.9 Hz, 2H), 3.41 – 3.33 (m, 4H), 2.06-2.01 (m, 2H), 1.78-1.72 (m, 4H), 0.94 (d, *J* = 6.7 Hz, 6H), 0.76 (dd, *J* = 14.8, 4.6 Hz, 1H), 0.74 – 0.70 (m, 1H), 0.68-0.64 (m, 2H), 0.40 – 0.32 (m, 2H), 0.062-0.055 (3H); ^{13}C NMR (150 MHz, $CDCl_3$) δ: **C1** [70.8 (u), 70.6 (u)], **C6** [48.2 (u)], **C2** [32.3 (dn), 32.21 (dn), 32.17 (dn)], **C5** [27.8 (u)], **C7** [19.95 (dn), 19.93 (dn), 19.78 (dn), 19.73 (dn)], **C3** [18.68 (u), 18.53 (u), 18.41 (u), 18.34 (u)], **C4** [13.41 (u), 13.09 (u), 12.90 (u)], **C8** [-3.04 (dn), -3.15 (dn), -3.30 (dn)]. FTIR (KBr /thin film) 3327, 2953, 1457, 1413, 1377, 1254, 1217, 1171, 1083, 1035, 852, 813, 660 cm^{-1}; HRMS (CI+) *m/z*: $[M\text{-}CH_3]^+$ calculated for $C_{11}H_{24}ClO_2Si^+$ 251.1229; Found 251.1241.

3,3'-((3-Chloropropyl)(methyl)silanediyl)bis(2-methylpropanal)

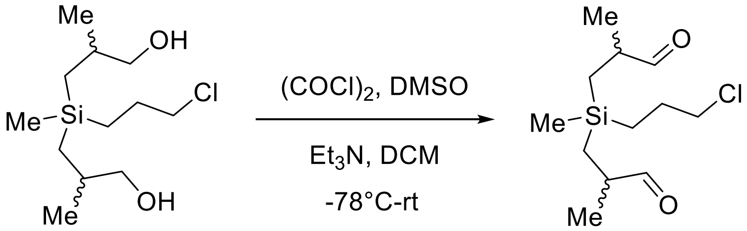

A 100 mL flame-dried round-bottomed flask was charged with oxalyl chloride (0.440 mL, 5.14 mmol, 3.00 equiv) and 15.6 mL dry CH_2Cl_2. The flask was cooled to –78 °C. Then dry DMSO (0.42 mL, 5.93 mmol, 3.50 equiv) in 5 mL dry CH_2Cl_2 was added dropwise to the flask. The mixture was stirred at –78 °C for 30 min. Then 3,3'-((3-chloropropyl)(methyl)silanediyl)bis(2-methylpropan-1-ol) (457 mg, 1.71 mmol, 1.00 equiv) in 3 mL dry CH_2Cl_2 was added to the mixture. The reaction was stirred at –78 °C for 20 min. Then dry triethylamine (2.38 mL, 17.1 mmol, 10.0 equiv), stir at –78 °C for 15 min. The reaction was then allowed to warm up to rt and stir for another 5 hours. The reaction was then quenched with saturated aq. NH_4Cl (20 mL). The aqueous layer was extract with 20 mL CH_2Cl_2 three times. The collected organic layer was dried with anhydrous $MgSO_4$, filtered, and concentrated via rotary evaporation. Purification by flash column chromatography (2%-10% Ethyl acetate/hexane) afforded the title compound as colorless oil (370 mg, 82% yield).

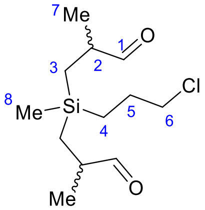

^{1}H NMR (600 MHz, $CDCl_3$) δ: 9.57 – 9.56 (m, 2H)， 3.51 (t, J = 6.7 Hz, 2H), 2.45-2.38 (m, 2H), 1.79-1.74 (m, 2H), 1.16 (d, J = 7.1 Hz, 6H), 1.07-1.05 (m, 2H), 0.71-0.69 (m, 2H), 0.56-0.53 (m, 2H), 0.09-0.08 (m, 3H); ^{13}C NMR (150 MHz, $CDCl_3$) δ: **C1** [204.4 (dn)], **C6** [47.8 (u)], **C2** [42.3 (dn)], **C5** [27.4 (u)], **C7** [16.73 (dn), 16.69 (dn)], **C3** [15.16 (u), 15.05 (u)], **C4** [12.7 (u), 12.5 (u), 12.3 (u)], **C8** [-3.49 (dn), -3.62 (dn), -3.69 (dn)]. FTIR (KBr /thin film) 2956, 2932, 1720, 1454, 1414, 1255, 1179, 1123, 1011, 813 cm^{-1}; HRMS (CI+) *m/z*: $[M-CH_3]^+$ calculated for $C_{11}H_{20}ClO_2Si^+$ 247.0916; Found 247.0929.

(3-Chloropropyl)(methyl)bis(2-methylbut-3-en-1-yl)silane

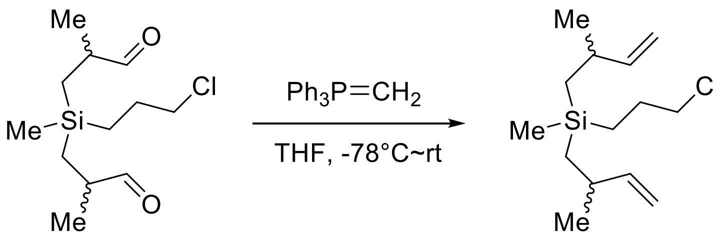

A solution of n-BuLi (2.5 M, 1.85 mL, 4.63 mmol, 3.30 equiv) in hexanes was added dropwise to a cooled (–78 °C) solution of methylphosphonium iodide (1.98 g, 4.91 mmol, 3.50 equiv) in dry THF (10.0 mL) and the mixture was allowed to warm to 0°C. After stirring for 50 min at 0°C, a solution of 3,3'-((3-chloropropyl)(methyl)silanediyl)bis(2-methylpropanal) (369 mg, 1.40 mmol, 1.00 equiv) in dry THF (2.00 mL) was added. The reaction mixture was stirred at rt for another 2 hours. The reaction mixture was partitioned between 20 mL CH_2Cl_2 and 20 mL saturated solution of $NaHCO_3$. The aqueous layer was extracted with 2×20mL CH_2Cl_2. The organic layer was combined, dried over $MgSO_4$, and concentrated. The crude residue was purified by flash chromatography (1% diethyl ether/hexane) to afford the title compound as colorless oil (276 mg, 76% yield).

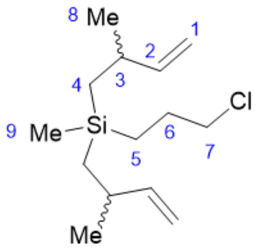

^{1}H NMR (600 MHz, $CDCl_3$) δ: 5.72 (ddd, J = 17.5, 10.1, 7.7 Hz, 2H), 4.94 (d, J = 17.1 Hz, 2H), 4.84 (d, J = 10.2 Hz, 2H), 3.49 (t, J = 7.0 Hz, 2H), 2.30 (dt, J = 13.8, 6.9 Hz, 2H), 1.78-173 (m, 2H), 1.03 (d, J = 6.7 Hz, 6H), 0.71-0.68 (m, 2H), 0.65-0.58 (m, 4H), 0.029 (s, 3H); ^{13}C NMR (150 MHz, $CDCl_3$) δ: **C2** [147.0 (dn)], **C1** [111.3 (u)], **C7** [48.2 (u)], **C3** [34.4 (dn)], **C6** [27.8 (u)], **C8** [24.40 (dn), 24.37 (dn)], **C4** [22.70 (u), 22.68 (u), 22.65 (u)], **C5** [13.06 (u), 13.04 (u)], **C9** [-3.11 (dn), -3.14 (dn)]. FTIR (KBr /thin film) 2956, 2925, 2898, 1639, 1456, 1370, 1253, 1121, 995, 910, 810 cm^{-1}; HRMS (CI+) *m/z*: $[M-C_5H_9]^+$ calculated for $C_9H_{18}ClSi^+$ 189.0861; Found 189.0869.

1-(3-Chloropropyl)-1,3,6-trimethyl-2,3,6,7-tetrahydro-1*H*-silephine

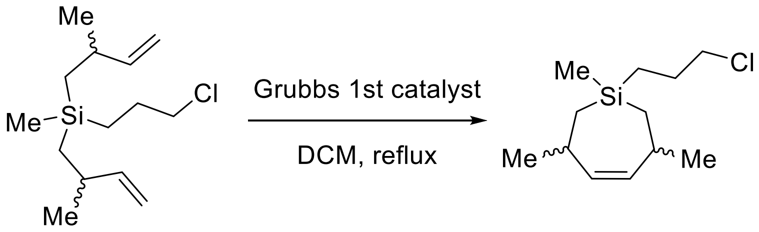

(3-chloropropyl)(methyl)bis(2-methylbut-3-en-1-yl)silane (389 mg, 1.50 mmol, 1.00 equiv) was dissolved in dry CH_2Cl_2 (144 mL). Grubbs 1st generation catalyst (61 mg, 0.074 mmol, 0.050 equiv) was added as a solution in CH_2Cl_2 (10 mL) and the mixture was refluxed under nitrogen for 4 hours. The mixture was cooled to rt, and the reaction mixture was concentrated via rotary evaporation. Purification by flash column chromatography (hexane) afforded the title compound (341 mg, 98% yield) as colorless oil.

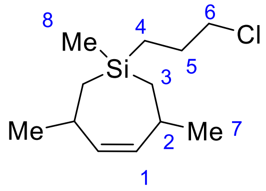

^{1}H NMR (600 MHz, $CDCl_3$) δ: **C1**[5.29 (m, 0.95H), 5.23 (m, 1.05H)], **C6**[3.54 (t, *J*=7.0 Hz, 0.47 H), 3.50 (t, *J*=7.0 Hz, 0.99 H), 3.46 (t, *J*=7.0 Hz, 0.52H)], **C2** [2.62-2.54 (m, 2H)], **C5**[1.81-1.68 (m, 2H)], **C7**[1.09-1.06 (m, 6H)], **C3**[0.90-0.59 (m, 4H)], **C4**[0.54-0.46 (m, 2H)], **C8**[0.08 (s, 0.86H), 0.01 (s, 1.48 H), -0.08(s, 0.60H)];
^{13}C NMR (150 MHz, $CDCl_3$) δ: **C1** [137.8 (dn), 137.7 (dn), 135.6 (dn), 135.5 (dn)], **C6** [48.3 (u), 48.2 (u), 48.1 (u)], **C2** [30.6 (dn), 30.4 (dn), 27.84 (dn), 27.54 (dn)], **C5** [27.91 (u), 27.68 (u), 27.52 (u)], **C7** [26.93 (dn), 26.89 (dn), 26.48 (dn), 26.41 (dn)], **C3** [22.3 (u), 22.2 (u), 21.6 (u)], **C4** [13.7 (u), 13.2 (u), 11.8 (u)], **C8** [-2.5 (dn), -3.4 (dn), -4.1 (dn)]. FTIR (KBr /thin film) 2952, 2900, 2869, 1646, 1455, 1370, 1252, 1096, 1013, 816, 727, 703 cm^{-1}; HRMS (CI+) *m/z*: $[M-CH_3]^+$ calculated for $C_{11}H_{20}ClSi^+$ 215.1017; Found 215.1029.

3-(1,3,6-Trimethyl-2,3,6,7-tetrahydro-1*H*-silepin-1-yl)propyl acetate

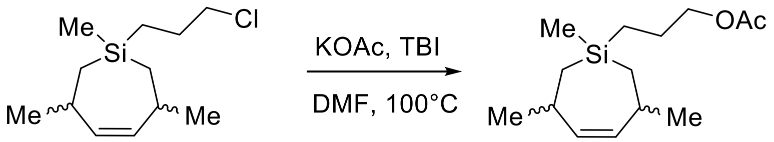

A 10 mL flame-dried round-bottomed flask was charged with KOAc (98 mg, 1.0 mmol, 2.0 equiv) and tetrabutylammonium iodide (9 mg, 0.03 mmol, 0.050 equiv). A solution of 1-(3-chloropropyl)-1,3,6-trimethyl-2,3,6,7-tetrahydro-1*H*-silephine (116 mg, 0.5 mmol, 1.0 equiv) in

dry DMF (1 mL) was added to the mixture. The reaction was heated up to 100 °C and allowed to stir overnight. The mixture was cooled and DMF was removed under reduced pressure. Brine (10 mL) and diethyl ether (10 mL) were added. The aqueous layer was extracted twice with 10 mL diethyl ether. The organic layers were dried over $MgSO_4$ and concentrated. The crude residue was purified by flash chromatography (1% diethyl ether/hexane) to afford 3-(1,3,6-trimethyl-2,3,6,7-tetrahydro-1*H*-silepin-1-yl)propyl acetate as colorless oil (122 mg, 96% yield).

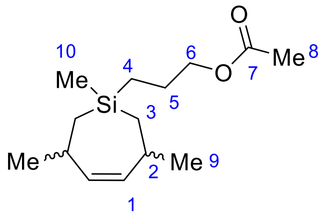

^{1}H NMR (600 MHz, $CDCl_3$) δ: **C1**[5.28 (m, 0.97H), 5.21 (m, 0.99H)], **C6**[4.04 (t, *J*=7.0 Hz, 0.42 H), 4.00 (t, *J*=7.0 Hz, 1.02 H), 3.97 (t, *J*=7.0 Hz, 0.56H)], **C2** [2.61-2.49 (m, 2H)], **C8**[2.06-2.03 (m, 3H)], **C5**[1.65-1.53 (m, 2H)], **C9**[1.08-1.05 (m, 6H)], **C3-C4**[0.89-0.78 (m, 1H), 0.74-0.59 (m, 2H), 0.52-0.40 (m, 3H)], **C10** [0.07 (s, 0.83H), 0.00 (s, 1.53 H), -0.10 (s, 0.65H)];
^{13}C NMR (150 MHz, $CDCl_3$) δ: **C7** [171.3 (u)], **C1** [137.83 (dn), 137.68 (dn), 135.56 (dn), 135.49 (dn)], **C6** [67.34 (u), 67.30 (u), 67.23 (u)], **C2** [30.6 (dn), 30.4 (dn), 27.83 (dn), 27.53(dn)],)], **C9** [26.93 (dn), 26.90 (dn), 26.49 (dn), 26.41 (dn)], **C5** [23.4 (u), 23.2 (u), 23.0 (u)], **C3** [22.3 (u), 22.2 (u), 21.6 (u)], **C8** [21.2 (dn)], **C4** [11.8 (u), 11.2 (u), 9.9 (u)], **C10** [-2.5 (dn), -3.4 (dn), -4.2 (dn)].
FTIR (KBr /thin film) 2953, 2924, 2899, 2870, 1743, 1455, 1364, 1236, 1047, 844, 817, 705 cm^{-1}; HRMS (CI+) *m/z*: $[M+H]^+$ calcd. for $C_{14}H_{27}O_2Si^+$ 255.1775, found 255.1767.

3-(1, 3, 6-Trimethyl-2,3,6,7-tetrahydro-1*H*-silepin-1-yl)propan-1-ol

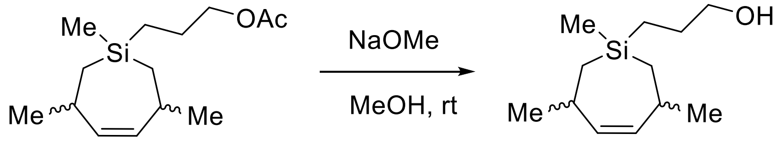

A 10 mL round-bottomed flask was charged with sodium methoxide (2 mg, 0.041 mmol, 0.1 equiv). A solution of 3-(1,3,6-trimethyl-2,3,6,7-tetrahydro-1*H*-silepin-1-yl)propyl acetate (105 mg, 0.41 mmol, 1.0 equiv) in MeOH (4 mL) was added. The reaction mixture was allowed to stir at rt overnight. After the reaction, 10 mL brine and 10 mL diethyl ether was added, and the aqueous layer was extracted with diethyl ether 3 times. The combined organic layers were dried with anhydrous $MgSO_4$, filtered, and concentrated via rotary evaporation. Purification by flash column chromatography (30% diethyl ether/*n*-hexane) afforded the desired 3-(1, 3, 6-trimethyl-2,3,6,7-tetrahydro-1*H*-silepin-1-yl)propan-1-ol as colorless oil (82 mg, 94% yield).

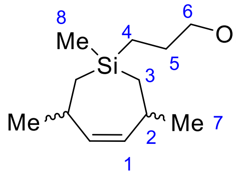

^{1}H NMR (600 MHz, $CDCl_3$) δ: **C1**[5.29 (m, 0.96H), 5.22 (m, 0.99H)], **C6**[3.63 (t, *J* =6.8 Hz, 0.43 H), 3.59 (t, *J* =6.8 Hz, 1.02 H), 3.56 (t, *J* =6.8 Hz, 0.57H)], **C2** [2.63-2.53 (m, 2H)], **C5**[1.62-1.51 (m, 2H)], 1.48 (br, 1H), **C7**[1.07-1.06 (m, 6H)], **C3**-**C4**[0.90-0.79 (m, 1H), 0.75-0.60 (m, 2H), 0.53-0.40 (m, 3H)], **C8**[0.07 (s, 0.84H), 0.00 (s, 1.51 H), -0.09(s, 0.64H)]; ^{13}C NMR (150 MHz, $CDCl_3$) δ: **C1** [137.8 (dn), 137.7 (dn), 135.6 (dn), 135.5 (dn)], **C6** [66.0 (u)], **C2** [30.6 (dn), 30.4 (dn), 27.86 (dn), 27.57 (dn)], **C5** [27.36 (u), 27.13 (u)], **C7** [26.98 (dn), 26.94 (dn), 26.51 (dn), 26.44 (dn)], **C3** [22.4 (u), 22.3 (u), 21.7 (u)], **C4** [11.6 (u), 11.0 (u), 9.7 (u)], **C8** [-2.5 (dn), -3.4 (dn), -4.1 (dn)].
FTIR (KBr /thin film) 3323, 2952, 2924, 2900, 2869, 1454, 1250, 1096, 1054, 1012, 843, 816, 704 cm^{-1}; HRMS (CI+) *m/z*: $[M-H]^+$ calcd. for $C_{12}H_{23}OSi^+$ 211.1513, found 211.1504.

But-3-en-1-yl(chloromethyl)dimethylsilane

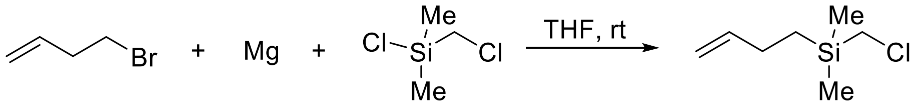

A dry round-bottomed flask was charged with Mg powder (468 mg, 19.5 mmol, 1.30 equiv) and dry THF (42 mL) under nitrogen atmosphere. 4-Bromo-1-butene (1.98 mL, 19.5 mmol, 1.30 equiv) was introduced to the flask dropwise via syringe. After magnesium powder was consumed and the formation of the Grignard reagent was complete, chloro(chloromethyl)dimethylsilane (1.98 mL, 15.0 mmol, 1.00 equiv) was added. The reaction mixture was allowed to stir at rt for 20 hours. Afterwards, THF was removed via rotary evaporation. 30 mL saturated NH_4Cl aqueous solution and 30 mL pentane were added, and aqueous layer was twice extracted with 30 mL portions of pentane. The organics were combined, dried with anhydrous $MgSO_4$, filtered, and concentrated via rotary evaporation. Purification by flash column chromatography (pentane) afforded the title compound as colorless oil (1.86 g, 11.4 mmol, 76% yield). ^{1}H NMR (400 MHz, $CDCl_3$) δ: 5.87 (ddt, *J* = 16.5, 10.1, 6.2 Hz, 1H), 5.04-4.99 (m, 1H), 4.94-4.90 (m, 1H), 2.79 (s, 2H), 2.14-2.08 (m, 2H), 0.79-0.75 (m, 2H), 0.13 (s, 6H); ^{13}C NMR (100 MHz, $CDCl_3$) δ: 141.1 (dn), 113.4 (u), 30.4 (u), 27.8 (u), 12.9 (u), -4.39 (dn); FTIR (KBr /thin film) 3441, 1643, 1254, 1095, 911, 821, 673 cm^{-1}; HRMS (CI+) *m/z*: $[M-Cl]^+$ calcd. for $C_7H_{15}Si^+$ 127.0943, found 127.0939.

1-(But-3-en-1-yldimethylsilyl)but-3-en-2-ol

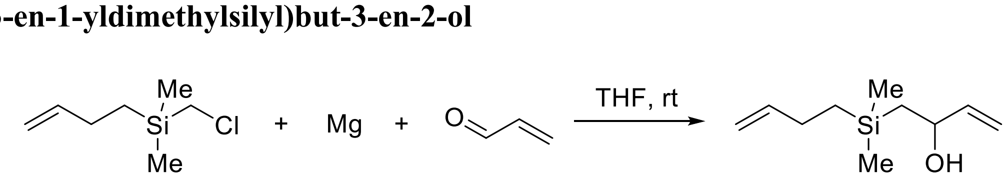

A dry round-bottomed flask was charged with magnesium powder (310 mg, 12.9 mmol, 1.05 equiv) under nitrogen atmosphere. But-3-en-1-yl(chloromethyl)dimethylsilane (2.00 g, 12.3 mmol, 1.00 equiv) was introduced to the flask as a dry THF (3.00 mL) solution via syringe. After initiation of the Grignard reaction, 7 mL of additional anhydrous THF was added to the mixture. The reaction was stirred at rt for 3 hours. After the Grignard reagent had formed, acrolein (0.821 mL, 12.3 mmol, 1.00 equiv) was added dropwise at 0°C. The reaction was stirred at rt for

another hour. Afterwards, THF was removed via rotary evaporation. Saturated aq. NH_4Cl (20 mL) and diethyl ether (20 mL) were added to the mixture. The aqueous layer was extracted with three 20 mL portions of diethyl ether. The organics were combined, dried with anhydrous $MgSO_4$, filtered, and concentrated via rotary evaporation. Purification by flash column chromatography (5% diethyl ether/hexane) afforded the title compound as colorless oil (1.24 g, 6.70 mmol, 55% yield). ^{1}H NMR (600 MHz, $CDCl_3$) δ: 5.91-5.84 (m, 2H), 5.17 (d, *J* = 17.2 Hz, 1H), 5.02 (d, *J* = 10.3 Hz, 1H), 4.98 (dd, *J* = 17.1, 1.5 Hz, 1H), 4.89 (d, *J* = 10.1 Hz, 1H), 4.28-4.25 (m, 1H), 2.06 (dt, *J* = 11.4, 6.5 Hz, 2H), 1.58 (br, 1H), 1.00 (dd, *J* = 14.4, 7.2 Hz, 1H), 0.91 (dd, *J* = 14.3, 7.3 Hz, 1H), 0.66-0.63 (m, 2H), 0.041 (s, 3H), 0.037 (s, 3H); ^{13}C NMR (150 MHz, $CDCl_3$) δ:143.6 (dn), 141.7 (dn), 113.6 (u), 112.9 (u), 71.6 (dn), 28.0 (u), 25.1(u), 15.1 (u), -2.34 (dn), -2.36 (dn); FTIR (NaCl /thin film) 3407, 2953, 2909, 1639, 1413, 1249, 991, 901, 837 cm^{-1}; HRMS (CI+) *m/z*: $[M-OH]^+$ calcd. for $C_{10}H_{19}Si^+$ 167.1256, found 167.1255.

1,1-dimethyl-2,3,6,7-tetrahydro-1H-silepin-3-ol

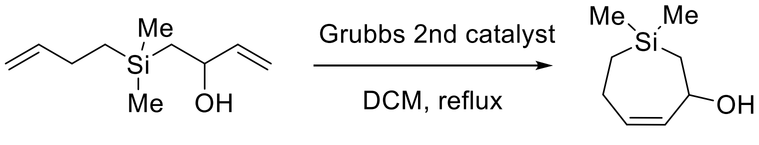

1-(but-3-en-1-yldimethylsilyl)but-3-en-2-ol (719 mg, 3.89 mmol, 1.00 equiv) was dissolved in dry CH_2Cl_2 (327 mL). Grubbs 2nd generation catalyst (99 mg, 0.117 mmol, 0.0300 equiv) was added as a solution in dry CH_2Cl_2 (10 mL) and the mixture was refluxed under nitrogen for 1 hours. The mixture was cooled to rt, and the reaction mixture was concentrated via rotary evaporation. Purification by flash column chromatography (5%-15% diethyl ether/hexane) afforded the title compound (504 mg, 83% yield) as yellow oil. ^{1}H NMR (400 MHz, $CDCl_3$) δ: 5.70-5.61 (m, 2H), 4.74 (d, *J* = 12.3 Hz, 1H), 2.26-2.11 (m, 2H), 1.70-1.65 (m, 1H), 1.12-1.06 (m, 1H), 1.01 (dd, *J* = 13.2, 2.8 Hz, 1H), 0.67 (ddd, *J* = 14.4, 7.0, 3.2 Hz, 1H), 0.53-0.46 (m, 1H), 0.11 (s, 3H), 0.00 (s, 3H); ^{13}C NMR (100 MHz, $CDCl_3$) δ: 138.5 (dn), 129.8 (dn), 67.2 (dn), 27.1 (u), 21.8 (u), 13.0 (u), -1.3 (dn), -2.5 (dn) ; FTIR (KBr /thin film) 3332, 2952, 2911, 2878, 2854, 1708, 1648, 1416, 1250, 1179, 1010, 924, 836, 800, 697, cm^{-1}; HRMS (CI+) *m/z*: $[M+H]^+$ calcd. for $C_8H_{17}OSi^+$ 157.1049, found 157.1035.

(*Z*) 4-(1-methyl-2,3,6,7-tetrahydro-1H-silepin-1-yl)butyl (4-nitrophenyl) carbonate

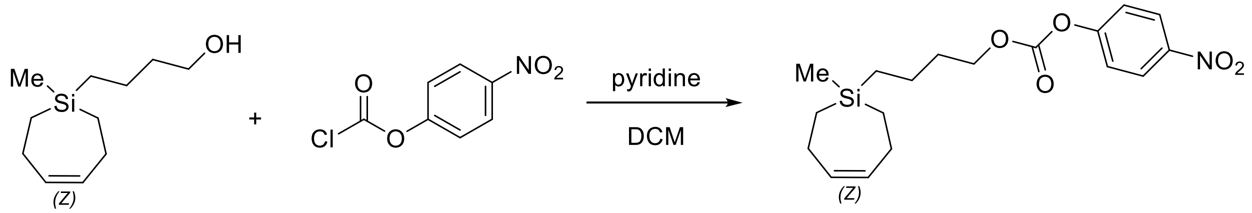

A round-bottomed flask was charged with 4-(1-methyl-2,3,6,7-tetrahydro-1*H*-silepin-1-yl)butan-1-ol (50 mg, 0.250 mmol, 1.00 equiv), and was then purged with nitrogen. Anhydrous methylene chloride (1.50 mL) and pyridine (50 mg, 0.63 mmol, 2.5 equiv) were sequentially added to the flask. A solution of 4-nitrophenyl chloroformate (102 mg, 0.510 mmol, 2.0 equiv) in anhydrous methylene chloride (1.00 mL) was added to the mixture via syringe and the resulting solution was allowed to stir for 30 minutes at rt. To the reaction was added saturated (aq.) NH_4Cl, and the layers were separated. The aqueous layer was extracted twice with methylene chloride. The organic layers were combined, dried with $MgSO_4$, filtered and concentrated via rotary evaporator. Flash chromatography (1%-10% diethyl ether/hexane) afforded the title compound (81 mg, 88% yield) as colorless oil. ^{1}H NMR (600 MHz, $CDCl_3$) δ: 8.27 (d, *J* = 9.1 Hz, 2H), 7.38 (d, *J* = 9.1 Hz, 2H), 5.76 (t, *J* = 4.5 Hz, 2H), 4.29 (t, *J* = 6.6 Hz, 2H), 2.25-2.17 (m, 4H), 1.80-1.75 (m, 2H), 1.46-1.41 (m, 2H), 0.67-0.57 (m, 6H), 0.027 (s, 3H); ^{13}C NMR (150 MHz, $CDCl_3$) δ:155.7 (u), 152.7 (u), 145.5 (u), 132.7 (dn), 125.4 (dn), 121.9 (dn), 69.4 (u), 32.4 (u), 21.2 (u), 20.0 (u), 14.6 (u), 12.7 (u), -3.93 (dn); FTIR (KBr /thin film) 2909, 2874, 1767, 1526, 1348, 1258, 1216, 860, 796, 699 cm^{-1}; HRMS (CI+) *m/z*: $[M+H]^+$ calculated for $C_{18}H_{26}NO_5Si^+$ 364.1580; Found 364.1576.

(Z)4-(1-methyl-2,3,6,7-tetrahydro-1*H*-silepin-1-yl)butyl (2-(2-((6-chlorohexyl)oxy)ethoxy)ethyl)carbamate

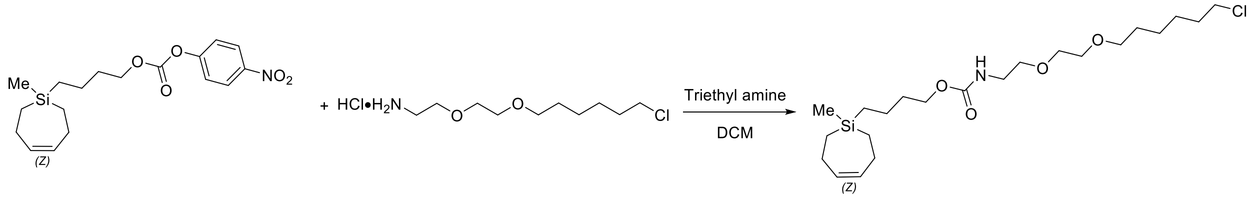

To a solution of (Z)4-(1-methyl-2,3,6,7-tetrahydro-1*H*-silepin-1-yl)butyl (4-nitrophenyl) carbonate (149 mg, 0.407 mmol, 1.00 equiv) and 2-[2-(6-chloro-hexyloxy)-ethoxy]-ethylammonium hydrochloride[5] (159 mg, 0.611 mmol, 1.50 equiv) in methylene chloride (3.7 mL) was added triethylamine (170 µl, 1.22 mmol, 3.00 equiv) in one portion. The reaction mixture was stirred at rt for 2 hours. The mixture was poured over 10 mL EtOAc and washed with 10 mL brine. The organic layer was dried with anhydrous $MgSO_4$, filtered and concentrated via rotary evaporator. The crude residue was purified by silica gel chromatography (15% diethyl ether/hexane, then 5% acetone/hexane) to afford the title compound as a mixture with nitrophenol. CH_2Cl_2 was added and the mixture was washed with 2×20 mL NaOH (1.00 M) to afford title compound (173 mg, 95% yield) as colorless oil. ^{1}H NMR (600 MHz, $CDCl_3$) δ: 5.78-5.73 (m, 2H), 5.15 (br s, 1H), 4.04 (t, *J* = 6.3 Hz, 2H), 3.66-3.59 (m, 2H), 3.56-3.52 (m, 6H), 3.46 (t, *J* = 6.7 Hz, 2H), 3.38-3.35 (m, 2H), 2.20 (dt, *J* = 10.3, 4.1 Hz, 4H), 1.80-1.75 (m, 2H), 1.63-1.58 (m, 4H), 1.48-1.43 (m, 2H), 1.40-1.32 (m, 4H), 0.65-0.53 (m, 6H), 0.00 (s, 3H); ^{13}C NMR (150 MHz, $CDCl_3$) δ: 157.0 (u), 132.73 (dn), 71.4 (u), 70.4 (u), 70.3 (u), 70.2 (u), 64.8 (u), 45.2 (u), 40.9 (u), 33.0 (u). 32.7 (u), 29.6 (u), 26.8(u), 25.6 (u), 21.2 (u), 20.1 (u), 14.6 (u), 12.8 (u), -3.9 (dn); FTIR (NaCl/thin film) 2934, 2860, 1722, 1521, 1251, 1119, 796, 698 cm^{-1}; HRMS (ESI+) *m/z*: $[M+H]^+$ calcd. for $C_{22}H_{43}NClO_4Si^+$ 448.2650, found 448.2634.

(*Z*) 2,5-dioxopyrrolidin-1-yl 4-(1-methyl-2,3,6,7-tetrahydro-1H-silepin-1-yl)butanoate

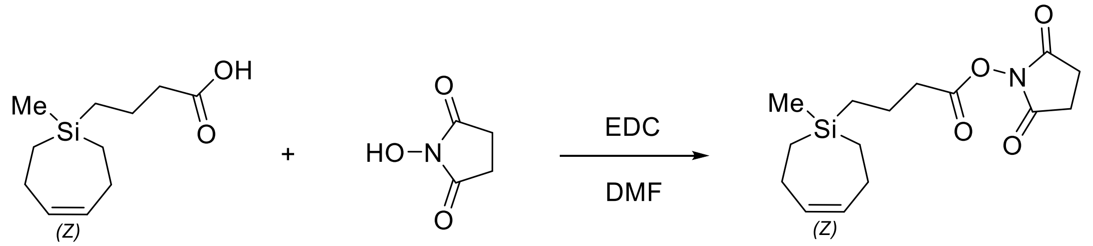

To a solution of (*Z*)4-(1-methyl-2,3,6,7-tetrahydro-1H-silepin-1-yl)butanoic acid (66 mg, 0.310 mmol, 1.00 equiv) and *N*-hydroxysuccinimide (72 mg, 0.625 mmol, 2.00 equiv) in DMF (1.80 mL) was added *N*-(3-Dimethylaminopropyl)-*N*′-ethylcarbodiimide hydrochloride (126 mg, 0.625 mmol, 2.00 equiv) in one portion. The reaction was stirred at rt for 14 hours. DMF solution was concentrated via rotary evaporation. Flash chromatography (10%-30% ethyl acetate/hexane) afforded the title compound (88 mg, 91% yield) as white solid, mp 41-43 °C. ^{1}H NMR (600 MHz, $CDCl_3$) δ: 5.74 (t, *J* = 4.5 Hz, 2H),2.86-2.73 (m, 4H), 2.61 (t, *J* = 7.2 Hz, 2H), 2.23-2.17 (m, 4H), 1.76-1.71 (m, 2H), 0.66-0.57 (m, 6H), 0.02 (s, 3H); ^{13}C NMR (150 MHz, $CDCl_3$) δ:169.3 (u), 168.5 (u), 132.6 (dn), 34.6 (u), 25.7 (u), 21.1 (u), 19.4 (u), 14.4 (u), 12.5 (u), -4.1 (dn); FTIR (KBr /thin film) 3015, 2908, 2876, 1814, 1784, 1740, 1430, 1366, 1207, 1069, 796, 648 cm^{-1}; HRMS (LIFDI-TOF) *m/z*: $[M]^+$ calcd. for $C_{15}H_{23}NO_4Si^+$, 309.1396 found 309.1407.

Synthesis procedure for carbocyclic *trans*-cycloheptenes
***trans*-Cycloheptene•$AgNO_3$(1a)**

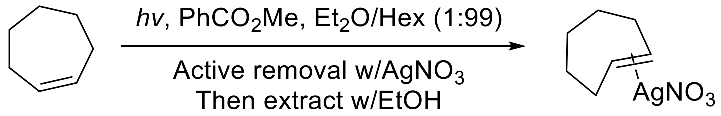

In a round bottomed flask, *cis*-cycloheptene (500 mg, 5.20 mmol, 1.00 equiv) and methyl benzoate (1.43 g, 10.4 mmol, 2.0 equiv) were dissolved in 2% diethyl ether/hexane (500 mL). The round bottomed flask was immersed in a cooling bath (NESLAB CB 80 with a CRYOTROL controller, bath temperature was set to -50 °C) and connected via PTFE tubing successively to an FMI "Q" pump, a three-way tee that was equipped with a thermometer probe, a coil of FEP tubing (total length: 1m; ID: 1/16 inch; OD 1/8 inch) and a 25g Biotage® column as illustrated in Figure S1. The FEP tubing coil was placed in a Rayonet® RPR-100 reactor. The bottom of the column was packed with dry silica gel (6 cm in height), and the top of the column was packed with silver nitrate impregnated silica (11.5 g of 10 wt% of $AgNO_3$ on SiO_2, 1.30 equiv). The column was flushed with 200 mL of the reaction solvent. The pump was turned on and the rate of circulation was adjusted to approx. 100 mL per minute. The temperature at the three-way tee was maintained at 0 ˚C. The lamp (254 nm) was then turned on, and photoisomerization of the stirring mixture was carried out for 6 hours. Afterwards, the sensitizer was flushed from the column with 300 mL of 10% ether in hexanes. The column was then dried by a stream of compressed nitrogen, and all of the silica gel in the cartridge was taken out and dissolved in 200 mL acetonitrile (HPLC grade). The acetonitrile solution was lyophilized, affording pale yellow semisolid consisting of *trans*-cycloheptene•$AgNO_3$ complex and free $AgNO_3$. Yields are

determined by titration 10 mL (out of 200 mL) acetonitrile solution of *trans*-cycloheptene•$AgNO_3$ with diphenyl tetrazine (run 1, 2.60 mmol, 50% yield; run 2, 2.91 mmol, 56% yield). The *trans*-cycloheptene •$AgNO_3$ complex was collected and stored as solution in ethanol (200 mL) at -20 °C. ^{1}H NMR (400 MHz, CD_3OD) δ: 5.57-5.54 (m, 2H), 2.55-2.50 (m, 2H), 2.28-2.19 (m, 2H), 1.75-1.56 (m, 6H); ^{13}C NMR (100 MHz, CD_3OD) δ: 120.2 (dn), 32.3 (u), 31.0 (u), 25.5 (u); FTIR (AgCl /thin film) 2934, 1756, 1717, 1558, 1302, 1077, 809, 718 cm^{-1}; HRMS (CI+) *m/z*: $[M-AgNO_3]^+$ calcd. for $C_7H_{13}^+$ 97.1017, found 97.1021.

(4*E*)-Cyclohept-4-en-1-ylmethanol •$AgNO_3$(1b)

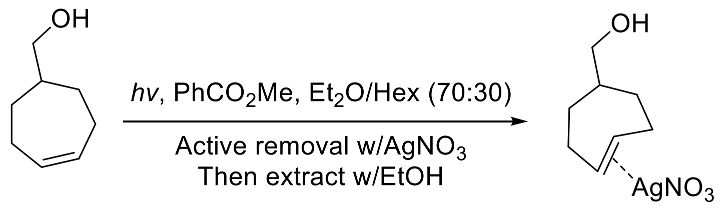

The (4*Z*)-Cyclohept-4-en-1-ylmethanol (100 mg, 0.79 mmol, 1.00 equiv) and methyl benzoate (216 mg, 1.59 mmol, 2.0 equiv) were dissolved in 100 mL of solvent (70% diethyl ether/hexane) in a round bottomed flask. The round bottomed flask was immersed in a cooling bath (NESLAB CB 80 with a CRYOTROL controller, bath temperature was set to -50 °C) and connected via PTFE tubing successively to an FMI "Q" pump, a three-way tee that was equipped with a thermometer probe, a coil of FEP tubing (total length: 1m; ID: 1/16 inch; OD 1/8 inch) and a 10g Biotage® column as illustrated in Figure 1. The FEP tubing coil was placed in a Rayonet® RPR-100 reactor. The bottom of the column was packed with dry silica gel (4.60 cm in height), and the top of the column was packed with silver nitrate impregnated silica (2.70 g of 10 wt% of $AgNO_3$ on SiO_2, 2.00 equiv). The column was flushed with 200 mL of the reaction solvent. The pump was turned on and the rate of circulation was adjusted to approx. 100 mL per minute. The temperature at the three-way tee was maintained at 0 ˚C. The lamp (254 nm) was then turned on, and photoisomerization of the stirring mixture was carried out for 2 hours. Afterwards, the sensitizer was flushed from the column with 100 mL of 10% ether in hexanes. The column was then dried by a stream of compressed nitrogen, and then flushed with 100 mL ethanol. The ethanol solution was concentrated via rotary evaporation, affording 306 mg of a dark brown viscous oil consisting of (4*E*)-cyclohept-4-en-1-ylmethanol •$AgNO_3$ complex (run 1: 0.474 mmol by NMR analysis, 60% yield; run 2: 0.545 mmol by NMR analysis, 69% yield) and free $AgNO_3$. ^{1}H NMR (400 MHz, CD_3OD) δ: 5.59 (ddd, *J* = 17.5, 9.9, 5.7 Hz, 1H), 5.31 (ddd, *J* = 17.6, 10.8, 2.6 Hz, 1H), 3.31-3.20 (m, 2H), 2.78-2.73 (m, 1H), 2.45-2.28 (m, 2H), 2.22-2.12 (m, 1H), 2.00-1.88 (m, 2H), 1.61-1.54 (m, 2H), 1.23-1.15 (m, 1H), 1.06-0.96 (m, 1H); ^{13}C NMR (100 MHz, CD_3OD) δ: 121.6 (dn), 118.6 (dn), 68.9 (u), 41.0 (dn), 37.2 (u), 36.4 (u), 35.3 (u), 26.8 (u); FTIR (AgCl /thin film) 3401, 2928, 1715, 1324, 1076, 1042, 1028, 816, 668 cm^{-1}; HRMS (CI+) *m/z*: $[M-OH-AgNO_3]^+$ calcd. for $C_8H_{13}^+$ 109.1017, found 109.1021.

General procedure for photoisomerization of silicon-containing *trans*-cycloheptene derivatives

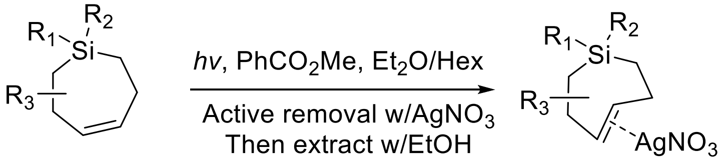

The (*Z*)-sila cycloheptene derivative (100 mg) and methyl benzoate (2.0 equiv) were dissolved in 100 mL of solvent in a quartz flask into which N_2 was sparged. The quartz flask was placed in a Rayonet® reactor and connected via PTFE tubing to a column (Biotage® SNAP cartridge, 10g) and an FMI pump. The bottom of the column was packed with silica gel, and the top of the column was packed with silver nitrate impregnated silica (2.0 equiv). The column was flushed with 7:3 hexane: diethyl ether. The pump was turned on and the rate of circulation was adjusted to approximately 100 mL per minute. The lamp was then turned on, and irradiation (254 nm) of the mixture was carried out for the indicated time. The conversion of the starting material was monitored by GC with dodecane used as standard. The column was washed with additional solvent (100 mL) and then dried by a stream of compressed nitrogen. The SNAP cartridge was then flushed with 150 mL of ethanol to afford an ethanol solution of (*E*)-sila cycloheptene•$AgNO_3$ derivative. The ethanol solution was concentrated via rotary evaporation, affording the corresponding (*E*)-sila cycloheptene•$AgNO_3$ derivative and free $AgNO_3$. The NMR yield of the *trans*-cycloheptene•$AgNO_3$ complex was determined by comparing the integration of the *trans*-alkene protons to mesitylene that was added as an NMR standard.

(*E*)-*Si*-(3-Cyanopropyl)-*Si*-methyl-5-silacycloheptene•$AgNO_3$(2b)

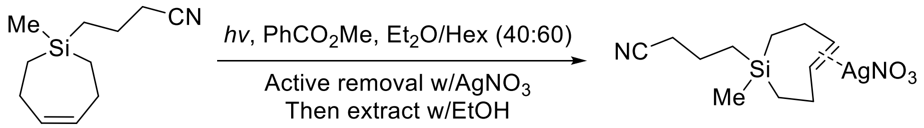

(Z)-*Si*-(3-Cyanopropyl)-*Si*-methyl-5-silacycloheptene (100 mg, 0.517 mmol, 1.00 equiv) and methyl benzoate (145 mg, 1.06 mmol, 2.05 equiv) were placed in a quartz flask and dissolved in 100 mL of 2:3 Et_2O: hexanes that had been degassed through three freeze/pump/thaw cycles. The SNAP cartridge was packed with dry silica gel and silver nitrate impregnated silica (1.76 g, 10wt% $AgNO_3$, 2.0 equiv). Dodecane (87 mg, 0.51 mmol, 1.0 equiv) was added to the flask to allow for GC monitoring. The solution in the quartz flask was then irradiated (254 nm) under continuous flow conditions (100 mL/min) for 3 hours with N_2 sparging, at which point GC analysis indicated that the reaction was complete. The SNAP cartridge was flushed with 200 mL of 1:4 Et_2O/hexanes and then dried with compressed nitrogen. The SNAP cartridge was then flushed with 225 mL of ethanol to afford an ethanol solution of (*E*)-*Si*-(3-cyanopropyl)-*Si*-methyl-5-silacycloheptene•$AgNO_3$. The ethanol solution was concentrated via rotary evaporation, affording 230 mg of a dark brown viscous oil containing the *trans*-cycloheptene•$AgNO_3$ complex and free $AgNO_3$. ^{1}H NMR analysis with an internal standard

(mesitylene) indicated that the yield of the title compound was 0.408 mmol (79% yield). A repetition of this experiment on the same scale gave 0.388 mmol (75% yield). ^{1}H NMR (400 MHz, CD_3OD) δ: 5.64-5.55 (m, 2H), 2.61-2.54 (m, 4H), 2.37-2.26 (m, 2H), 1.72-1.64 (m, 2H), 1.13-1.00 (m,2H), 0.94-0.81 (m, 2H), 0.76-0.67 (m, 2H), 0.051 (s, 3H); ^{13}C NMR (100 MHz, CD_3OD) δ: 122.5 (u), 120.1 (dn), 119.7 (dn), 28.6 (u), 28.5 (u), 21.14 (u), 21.09 (u), 18.5 (u), 16.0 (u), -3.1 (dn); FTIR (ATR) 3430, 2940, 2880, 2270, 1730, 1560, 1370, 1270, 1030, 924, 856, 810, 727, cm^{-1}; HRMS (CI+) *m/z*: $[M+H-AgNO_3]^+$ calcd. for $C_{11}H_{20}NSi^+$ 194.1365, found 194.1372.

(*E*)-*Si*-(3-Cyanopropyl)-*Si*-methyl-5-silacycloheptene (7b): without concentration

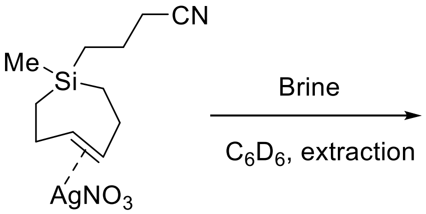

To 0.398 mmol of (*E*)-*Si*-(3-cyanopropyl)-*Si*-methyl-5-silacycloheptene•$AgNO_3$ was added 2 mL C_6D_6, followed by generous excess of brine (3 mL). The mixture was shaked for a couple of seconds. The precipitate (AgCl) was filtered off and the C_6D_6 layer was separated, producing an organic solution of (*E*)-*Si*-(3-cyanopropyl)-*Si*-methyl-5-silacycloheptene. ^{1}H NMR and ^{13}C NMR were taken, indicating that the solution contained 98% *trans* isomer and 2% *cis* isomer. ^{1}H NMR (400 MHz, C_6D_6) δ: 5.52 (ddd, J = 16.9, 9.7, 4.9 Hz, 1H), 5.42 (ddd, J = 17.0, 9.2, 5.2 Hz, 1H), 2.23-2.05 (m, 4H), 1.46 (t, J = 7.0 Hz, 2H), 1.04-0.97 (m, 2H), 0.69-0.64 (m, 2H), 0.49-0.40 (m, 2H), 0.21-0.16 (m, 2H), -0.24 (s, 3H); ^{13}C NMR (100 MHz, C_6D_6) δ: 135.6, 134.8, 119.5, 27.6, 27.0, 20.8, 20.5, 20.1, 19.8, 15.6, -2.8.

(*E*)-*Si*-(4-Hydroxybutyl)-*Si*-methyl-5-silacycloheptene•$AgNO_3$ (2c)

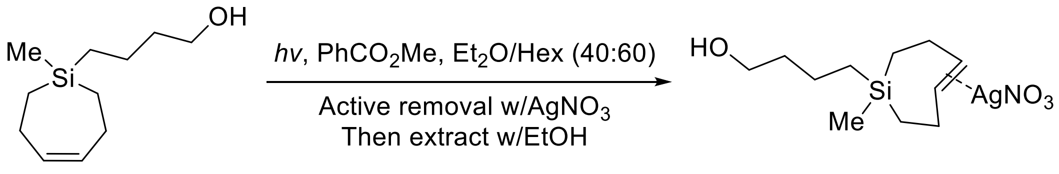

(*Z*)-*Si*-(4-Hydroxybutyl)-*Si*-Methyl-5-silacycloheptene (100 mg, 0.510 mmol, 1.00 equiv) and methyl benzoate (138 mg, 1.02 mmol, 2.00 equiv) were placed in a quartz flask and dissolved in 100 mL of 2:3 Et_2O: hexanes that had been degassed through three freeze/pump/thaw cycles. The SNAP cartridge was packed with dry silica gel and silver nitrate impregnated silica (1.73 g, 10wt% $AgNO_3$, 2.0 equiv). Dodecane (86 mg, 0.51 mmol, 1.0 equiv) was added to the flask to allow for GC monitoring. The solution in the quartz flask was then irradiated (254 nm) under continuous flow conditions (100 mL/min) for 3 hours with N_2 sparging, at which point GC analysis indicated that the reaction was complete. The SNAP cartridge was flushed with 200 mL of 1:4 Et_2O/hexanes and then dried with compressed nitrogen. The SNAP cartridge was then flushed with 225 mL of ethanol to afford an ethanol solution of (*E*)-*Si*-(4-Hydroxybutyl)-*Si*-methyl-5-silacycloheptene•$AgNO_3$. The ethanol solution was concentrated via rotary

evaporation, affording 226 mg of tan viscous oil containing the *trans*-cycloheptene•$AgNO_3$ complex and free $AgNO_3$. ^{1}H NMR analysis with an internal standard (mesitylene) indicated that the yield of the title compound was 0.377 mmol (74% yield). A repetition of this experiment on the same scale gave 0.357 mmol (70% yield). ^{1}H NMR (400 MHz, CD_3OD) δ: 5.60-5.49 (m, 2H), 3.55 (t, J = 6.4 Hz, 2H), 2.59 – 2.46 (m, 2H), 2.36 – 2.25 (m, 2H), 1.59-1.52 (m,2H), 1.42-1.34 (m, 2H), 1.11-0.98 (m, 2H), 0.92-0.78 (m, 2H), 0.61-0.53 (m, 2H), 0.020 (s, 3H); ^{13}C NMR (100 MHz, CD_3OD) δ: 123.8 (dn), 123.6 (dn), 62.5 (u), 37.5 (u), 28.7 (u), 28.6 (u), 21.1 (u), 19.0 (u), 16.4 (u), -2.69 (dn); FTIR (ATR) 3382, 2932, 2873, 1559, 1400, 1280, 1130, 1027, 857, 801, 727 cm^{-1}; HRMS (CI+) *m/z*: $[M+H-AgNO_3]^+$ calcd. for $C_{11}H_{23}OSi^+$ 199.1518, found 199.1522.

(*E*)-*Si*-(4-Hydroxybutyl)-*Si*-methyl-5-silacycloheptene: concentration gives an E/Z mixture

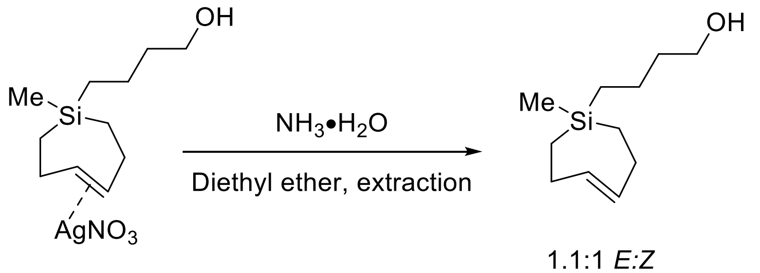

1.1:1 *E:Z*

To 0.24 mmol (189 mg mixed with excess $AgNO_3$) of (*E*)-*Si*-(4-Hydroxybutyl)-*Si*-methyl-5-silacycloheptene•$AgNO_3$ was added 10 mL ammonia solution, extracted with 3×10mL diethyl ether. The organic layers were separated, combined and dried over $MgSO_4$. The diethyl ether solution was concentrated via rotary evaporation, filtered through a plug (2.2 cm in height) of silica gel in a pipet (Fisherbrand^{TM} disposal large-volume pasteur pipets, 4 ml capacity) and rinsed with 15 ml 40% diethyl ether/hexane. The filtrate was concentrated via rotary evaporation, affording 44.2 mg of the (*E*)-*Si*-(4-Hydroxybutyl)-*Si*-methyl-5-silacycloheptene as a colorless oil, mixed with its cis isomer (1.1:1 *E:Z*) as determined by ^{1}H NMR analysis.

(*E*)-*Si*-(4-Hydroxybutyl)-*Si*-methyl-5-silacycloheptene (7c): without concentration

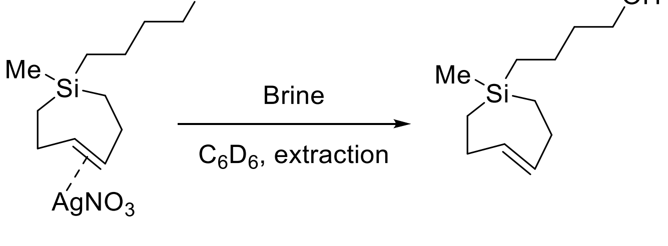

To 0.38 mmol of (*E*)-*Si*-(4-Hydroxybutyl)-*Si*-methyl-5-silacycloheptene •$AgNO_3$ was added 2 mL C_6D_6, followed by 2 ml of brine. The mixture was shaked for a couple of seconds. The precipitate (AgCl) was filtered off and the C_6D_6 layer was separated and dried over $MgSO_4$, producing a C_6D_6 solution of (*E*)-*Si*-(4-Hydroxybutyl)-*Si*-methyl-5-silacycloheptene. ^{1}H NMR and ^{13}C NMR were taken, indicating that the solution contained 98% *trans* isomer and 2% *cis* isomer. ^{1}H NMR (400 MHz, C_6D_6) δ: 5.62-5.50 (m, 2H), 3.40 (t, J = 6.4 Hz, 2H), 2.32-2.13 (m,

4H), 1.47-1.40 (m, 2H), 1.32-1.22 (m, 2H), 0.98 (br, 1H), 0.91-0.76 (m, 2H), 0.67-0.54 (m, 2H), 0.45-0.32 (m, 2H), -0.08 (s, 3H); ^{13}C NMR (100 MHz, C_6D_6) δ: 135.4 (dn), 134.9 (dn), 62.4 (u), 37.1 (u), 27.6 (u), 27.4 (u), 20.6 (u), 20.2 (u), 20.1 (u), 16.1 (u), -2.5 (dn). HRMS (ESI+) *m/z*: $[M+H]^+$ calcd. for $C_{11}H_{23}OSi^+$ 199.1513, found 199.1507

(*E*)-1,1-diphenyl-2,3,6,7-tetrahydro-1H-silepine •AgNO₃(2a)

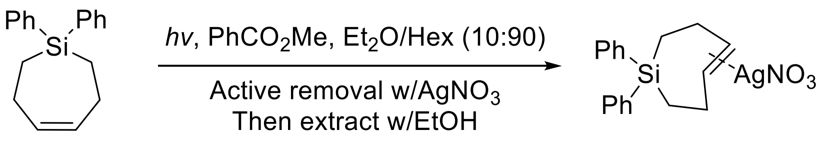

(*Z*)-*Si, Si*-diphenyl-5-silacycloheptene (100 mg, 0.378 mmol, 1.00 equiv) and methyl benzoate (285 mg, 1.89 mmol, 5.0 equiv) were placed in a quartz flask and dissolved in 100 mL of 1:24 Et_2O: hexanes that had been degassed through three freeze/pump/thaw cycles. The SNAP cartridge was packed with dry silica gel and silver nitrate impregnated silica (1.29 g, 10wt% $AgNO_3$, 2.0 equiv). Dodecane (64 mg, 0.38 mmol, 1.0 equiv) was added to the flask to allow for GC monitoring. The solution in the quartz flask was then irradiated (254 nm) under continuous flow conditions (100 mL/min) for 6 hours with N_2 sparging, at which point GC analysis indicated that the reaction was complete. The SNAP cartridge was flushed with 100 mL of 1:9 Et_2O/hexanes and then dried with compressed nitrogen. The SNAP cartridge was then flushed with 100 mL of ethanol to afford an ethanol solution of (*E*)-*Si*, *Si*-diphenyl-5-silacycloheptene•$AgNO_3$. The ethanol solution was concentrated via rotary evaporation, affording 182 mg of a dark brown viscous oil containing the *trans*-cycloheptene•$AgNO_3$ complex and free $AgNO_3$. ^{1}H NMR analysis with an internal standard (mesitylene) indicated that the yield of the title compound was 0.213 mmol (56% yield). A repetition of this experiment on the same scale gave 0.190 mmol (50% yield). ^{1}H NMR (400 MHz, CD_3OD) δ: 7.48-7.46 (m, 4H), 7.37-7.34 (m, 6H), 5.57-5.55 (m, 2H), 2.68-2.61 (m, 2H), 2.45-2.36 (m, 2H), 1.67 (ddd, J = 14.7, 8.5, 4.5 Hz, 2H), 1.52-1.45 (m, 2H); ^{13}C NMR (100 MHz, CD_3OD) δ: 137.9 (u), 135.2 (dn), 130.2 (dn), 129.1 (dn), 120.2 (dn), 28.6 (u), 17.9 (u); FTIR (ATR) 3380, 3060, 2930, 2880, 1720, 1560, 1430, 1280, 1110, 1030, 997, 852, 741, 698, 613, 534, cm^{-1}; HRMS (CI+) *m/z*: $[M+H-AgNO_3]^+$ calcd. for $C_{18}H_{21}Si^+$ 265.1413, found 265.1424.

(*E*)-1,1-dimethyl-2,3,6,7-tetrahydro-1H-silepin-3-ol•AgNO₃(2e)

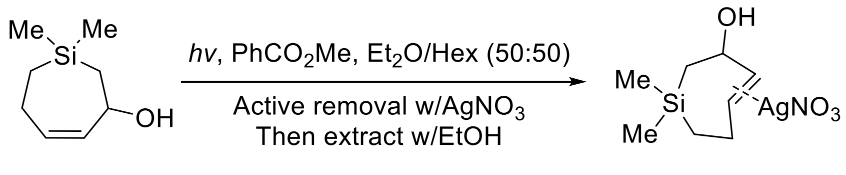

(*Z*)-1,1-dimethyl-2,3,6,7-tetrahydro-1H-silepin-3-ol (108 mg, 0.691 mmol, 1.00 equiv) and methyl benzoate (188 mg, 1.38 mmol, 2.00 equiv) were placed in a quartz flask and dissolved in 108 mL of 45:55 Et_2O: hexanes that had been degassed through three freeze/pump/thaw cycles. The SNAP cartridge was packed with dry silica gel and silver nitrate impregnated silica (2.35 g,

10wt% $AgNO_3$, 2.0 equiv). Dodecane (118 mg, 0.691 mmol, 1.00 equiv) was added to the flask to allow for GC monitoring. The solution in the quartz flask was then irradiated (254 nm) under continuous flow conditions (100 mL/min) for 1 hour with N_2 sparging, at which point GC analysis indicated that the reaction was complete. The SNAP cartridge was flushed with 100 mL of 3:7 Et_2O/hexanes and then dried with compressed nitrogen. The SNAP cartridge was then flushed with 100 mL of ethanol to afford an ethanol solution of (*E*)-*Si*, *Si*-diphenyl-5-silacycloheptene•$AgNO_3$. The ethanol solution was concentrated via rotary evaporation, affording 305 mg of brown viscous oil containing the *trans*-cycloheptene•$AgNO_3$ complex and free $AgNO_3$. ^{1}H NMR analysis with an internal standard (mesitylene) indicated that the yield of the title compound was 0.459 mmol (66% yield). A repetition of this experiment on the same scale gave 0.449 mmol (65% yield).

Major diastereomer : ^{1}H NMR (600 MHz, CD_3OD) δ: 5.72 (ddd, *J* = 16.0, 10.3, 5.3 Hz, 1H), 5.49 (dd, *J* = 16.9, 9.0 Hz, 1H), 4.53 (td, *J* = 9.1, 6.4 Hz, 1H), 2.48-2.37 (m, 2H), 1.38 (dd, *J* = 14.1, 6.2 Hz, 1H), 0.97 (ddd, *J* = 14.7, 10.6, 6.1 Hz, 1H), 0.90-0.84 (m, 2H); 0.08 (s, 3H), 0.00 (s, 3H); ^{13}C NMR (150 MHz, CD_3OD) δ: 124.2 (dn), 114.7 (dn), 73.4 (dn), 29.8 (u), 26.6 (u), 18.9 (u), -0.96 (dn), -1.3 (dn);
Minor diastereomer: ^{1}H NMR (600 MHz, CD_3OD) δ: 5.93-5.87 (m, 1H), 5.67 (dd, *J* = 16.7, 2.5 Hz, 1H), 4.75-4.73 (m, 1H), 2.64-2.59 (m, 1H), 2.40-2.31 (m, 1H), 1.29 (dd, *J* = 14.5, 5.7 Hz, 1H), 1.06-1.02 (m, 1H), 0.94 (dd, *J* = 14.5, 4.0 Hz, 1H), 0.82-0.77 (m, 1H), 0.06 (s, 3H), 0.01 (s, 3H); ^{13}C NMR (150 MHz, CD_3OD) δ: 123.3 (dn), 114.6 (dn), 69.4 (dn), 31.0 (u), 28.8 (u), 19.9 (u), -0.25 (dn), -1.46 (dn) ; FTIR (ATR as a mixture of two diastereomers) 3203, 2946, 2872, 1571, 1389, 1304, 1291, 1248, 1170, 1105, 1059, 1038, 863, 829, 662cm^{-1}; HRMS (CI+) *m/z*: $[M+H-AgNO_3]^+$ calcd. for $C_8H_{17}OSi^+$ 157.1049, found 157.1047.

(*E*)-3-(1,3,6-trimethyl-2,3,6,7-tetrahydro-*1* H-silepin-1-yl)propan-1-ol•$AgNO_3$ (2d) and (*E*)-3-(1,3,6-trimethyl-2,3,6,7-tetrahydro-*1* H-silepin-1-yl)propan-1-ol (7d)

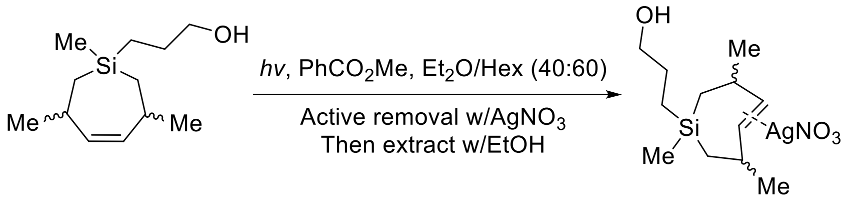

(*Z*)- 3-(1,3,6-trimethyl-2,3,6,7-tetrahydro-*1* H-silepin-1-yl)propan-1-ol (101 mg, 0.470 mmol, 1.00 equiv) and methyl benzoate (323 mg, 2.35 mmol, 5.00 equiv) were placed in a quartz flask and dissolved in 100 mL of 2:3 Et_2O: hexanes that had been degassed through three freeze/pump/thaw cycles. The SNAP cartridge was packed with dry silica gel and silver nitrate impregnated silica (1.60 g, 10wt% $AgNO_3$, 2.0 equiv). Dodecane (80 mg, 0.47 mmol, 1.00 equiv) was added to the flask to allow for GC monitoring. The solution in the quartz flask was then irradiated (254 nm) under continuous flow conditions (100 mL/min) for 6 hours with N_2 sparging, at which point GC analysis indicated that the reaction was complete. The SNAP cartridge was flushed with 100 mL of 1:4 Et_2O/hexanes and then dried with compressed nitrogen. The SNAP cartridge was then flushed with 150 mL of ethanol to afford an ethanol solution of (*E*)-3-(1,3,6-trimethyl-2,3,6,7-tetrahydro-*1* H-silepin-1-yl)propan-1-ol •$AgNO_3$. The

ethanol solution was concentrated via rotary evaporation, affording 213 mg of tan viscous oil containing the *trans*-cycloheptene•$AgNO_3$ complex and free $AgNO_3$. ^1H NMR analysis with an internal standard (mesitylene) indicated that the yield of the title compound was 0.320 mmol (68% yield). A repetition of this experiment on the same scale gave 0.282 mmol (60% yield). To 0.32 mmol of the title compound was added 5 ml diethyl ether and 5 ml ammonia solution. The aqueous layer was further extracted with 5 ml diethyl ether twice. The organics were combined and dried over $MgSO_4$. The diethyl ether solution was concentrated via rotary evaporation, affording 65 mg of **7d** (mixture of diastereomers) as a colorless oil.

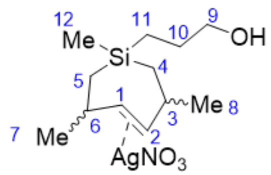

^1H NMR (600 MHz, CD_3OD) δ: **C1-C2** [6.02-5.93 (m, 0.83H), 5.32-5.23 (m, 1.01H)], **C9** [3.53-3.45 (m, 2H)], **C3C6** [2.94-2.57 (m, 2H)], **C10** [1.55-1.45 (m, 2H)], **C7C8** [1.30-1.19 (m, 6H)], **C5C4C11** [1.15-1.05 (m, 2H), 0.77-0.41 (m, 4H)], **C12** [0.10 (s, 0.80H), 0.04 (s, 1.38 H), -0.06 (s, 0.73H)];
^{13}C NMR (100 MHz, CD_3OD) δ: **C1C2** [122.98 (dn), 122.80 (dn), 122.69 (dn), 122.56 (dn), 121.81 (dn), 121.45 (dn), 121.22 (dn), 120.83 (dn)], **C9** [65.82 (u), 65.78 (u), 65.7(u)], **C3C6** [39.4 (dn), 39.3 (dn), 37.2 (dn), 36.8 (dn), 33.9 (dn), 33.7 (dn), 32.5 (dn), 32.23 (dn)], **C10** [29.09 (u), 28.83 (u)], **C5C4** [27.81(u), 27.71(u), 27.68(u), 27.60(u), 27.54(u), 27.18(u), 27.13(u)], **C7C8** [23.59 (dn), 23.57 (dn), 23.51 (dn), 23.49 (dn), 21.42 (dn), 21.35 (dn), 20.00(dn), 19.76 (dn)], **C11** [13.08 (u), 12.58 (u), 12.38 (u), 11.60 (u)], **C12** [-1.94 (dn), -2.30 (dn), -2.90 (dn), -3.17 (dn)].
FTIR (ATR as a mixture of diastereomers) 3421, 2955, 2926, 2868, 1559, 1408, 1375, 1283, 1254, 1172, 1141, 1032, 861, 801, cm^{-1}; HRMS (CI+) *m/z*: $[M+H-AgNO_3]^+$ calcd. for $C_{12}H_{25}OSi^+$ 213.1675, found 213.1665.

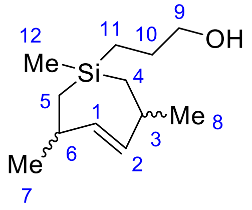

^1H NMR (600 MHz, C_6D_6) δ: **C1-C2** [5.94-5.85 (m, 0.88H), 5.29-5.18 (m, 1.10H)], **C9** [3.44-3.39 (m, 2H)], **C3C6** [2.72-2.51, 2.41-2.33 (m, 2H)], 1.64 (br s, 1 H), **C10** [1.44-1.35 (m, 2H)], **C7C8** [1.22-1.18, 1.14-1.09 (m, 6H)], **C5C4C11** [1.06-0.81 (m, 2H), 0.58-0.14 (m, 4H)], **C12** [-0.03 (s, 0.80H), -0.05 (s, 0.82H), -0.07 (s, 0.51H), -0.14 (s, 0.80H)];
^{13}C NMR (150 MHz, C_6D_6) δ: **C1C2** [138.2 (dn), 138.1 (dn), 137.5 (dn), 135.3 (dn), 135.0 (dn), 134.2 (dn)], **C9** [65.8 (u), 65.7 (u), 65.6(u)], **C3C6** [38.04 (dn), 38.02 (dn), 36.0 (dn), 35.3 (dn), 32.7 (dn), 32.4 (dn), 31.4 (dn), 31.1 (dn)], **C10** [31.6 (u), 30.1 (u), 29.8 (u)], **C5C4** [29.6(u), 29.4(u), 29.3(u), 29.2(u), 27.73(u), 27.72(u), 27.68(u), 27.66 (u)], **C7C8** [22.8 (dn), 22.72 (dn),

22.66 (dn), 22.62 (dn), 20.1 (dn), 20.0 (dn), 19.0 (dn), 18.5 (dn)], **C11** [12.7 (u), 12.3 (u), 12.2 (u), 11.4 (u)], **C12** [-1.6 (dn), -1.9 (dn), -2.5 (dn), -2.7 (dn)].
FTIR (ATR as a mixture of diastereomers) 3315, 2953, 2924, 2866, 1624, 1453, 1374, 1250, 1051, 1009, 982, 862, 828, 788 cm^{-1}; HRMS (ESI+) *m/z*: $[M+H]^+$ calcd. for $C_{12}H_{25}OSi^+$ 213.1669, found 213.1668.

(*E*) 2,5-dioxopyrrolidin-1-yl 4-(1-methyl-2,3,6,7-tetrahydro-1*H*-silepin-1-yl)butanoate•$AgNO_3$(2f)

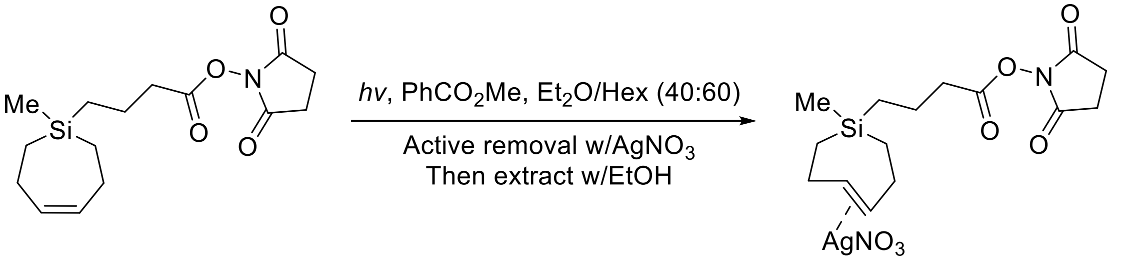

(Z) 2,5-dioxopyrrolidin-1-yl 4-(1-methyl-2,3,6,7-tetrahydro-1*H*-silepin-1-yl)butanoate (118 mg, 0.380 mmol, 1.00 equiv) and methyl benzoate (261 mg, 1.90 mmol, 5.00 equiv) were placed in a quartz flask and dissolved in 80 mL of 40% Et_2O/hexanes. The SNAP cartridge was packed with dry silica gel and silver nitrate impregnated silica (1.29 g, 10wt% $AgNO_3$, 2.0 equiv). Dodecane (65 mg, 0.38 mmol, 1.0 equiv) was added to the flask to allow for GC monitoring. The solution in the quartz flask was then irradiated (254 nm) under continuous flow conditions (100 mL/min) for 2.5 hours, at which point GC analysis indicated that the reaction was complete. The SNAP cartridge was flushed with 100 mL of 30% Et_2O/hexanes and then dried with compressed nitrogen. The SNAP cartridge was then flushed with 100 mL of ethanol to afford an ethanol solution of (*E*) 2,5-dioxopyrrolidin-1-yl 4-(1-methyl-2,3,6,7-tetrahydro-1*H*-silepin-1-yl)butanoate •$AgNO_3$. The ethanol solution was concentrated via rotary evaporation, affording 183 mg of brown viscous oil containing the *trans*-cycloheptene•$AgNO_3$ complex and free $AgNO_3$. ^{1}H NMR analysis with an internal standard (mesitylene) indicated that the yield of the title compound was 0.217 mmol (57% yield). A repetition of this experiment on the same scale gave 0.186 mmol (49% yield). ^{1}H NMR (400 MHz, CD_3CN) δ: 5.48-5.37 (m, 2H), 2.72 (s, 4H), 2.54 (t, *J* = 6.9 Hz, 2H), 2.38-2.33 (m, 2H), 2.20-2.10 (m, 2H), 1.62-1.54 (m, 2H), 0.97-0.84 (m, 2H), 0.78-0.65 (m, 2H), 0.60-0.47 (m, 2H), -0.10 (s, 3H); ^{13}C NMR (100 MHz, CD_3CN) δ: 171.3 (u), 169.9 (u), 120.9 (dn), 120.7 (dn), 34.8 (u), 28.3 (u), 28.2 (u), 26.3 (u), 20.1 (u), 18.22 (u), 18.18 (u), 15.3 (u), -2.9 (dn); FTIR (ATR) 3448, 2995, 2935, 2875, 1734, 1559, 1365, 1279, 1206, 1068, 1031, 858, 811, 728 cm^{-1}; HRMS (CI+) *m/z*: $[M+H-AgNO_3]^+$ calcd. for $C_{15}H_{24}NO_4Si^+$ 310.1474, found 310.1467.

(*E*) 4-(1-methyl-2,3,6,7-tetrahydro-1H-silepin-1-yl)butyl (2-(2-((6-chlorohexyl)oxy)ethoxy)ethyl)carbamate•$AgNO_3$(2g)

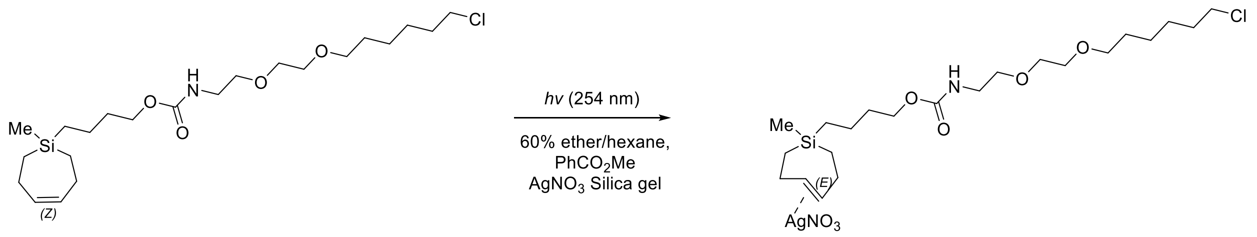

(*Z*) 4-(1-methyl-2,3,6,7-tetrahydro-1H-silepin-1-yl)butyl (2-(2-((6-chlorohexyl)oxy)ethoxy)ethyl)carbamate (101 mg, 0.226 mmol, 1.00 equiv) and methyl benzoate (155 mg, 1.13 mmol, 5.00 equiv) were placed in a quartz flask and dissolved in 43 mL of 60% Et_2O/hexanes. Dodecane (38 mg, 0.23 mmol, 1.0 equiv) was added to the flask to allow for GC monitoring. The solution in the quartz flask was then irradiated (254 nm) under continuous flow conditions (100 mL/min) for 2 hours, at which point GC analysis indicated that the reaction was complete. The SNAP cartridge was flushed with 100 mL of 20% Et_2O/hexanes and then dried with compressed nitrogen. The SNAP cartridge was then flushed with 100 mL of EtOH to afford an ethanol solution of (*E*) 4-(1-methyl-2,3,6,7-tetrahydro-1H-silepin-1-yl)butyl (2-(2-((6-chlorohexyl)oxy)ethoxy)ethyl)carbamate•$AgNO_3$. The ethanol solution was concentrated via rotary evaporation, affording light yellow viscous oil containing the *trans*-cycloheptene•$AgNO_3$ complex and free $AgNO_3$. ^{1}H NMR analysis with an internal standard (mesitylene) indicated that the yield of the title compound was 0.098 mmol (43% yield). A repetition of this experiment on the same scale gave 0.095 mmol (42% yield). ^{1}H NMR (600 MHz, CD_3OD) δ: 5.61-5.53 (m, 2H), 4.04 (t, J =6.3 Hz, 2H), 3.61-3.55 (m, 6H), 3.53 (t, J =5.6 Hz, 2H), 3.49 (t, J =6.6 Hz, 2H), 3.28 (t, J =5.5 Hz, 2H), 2.55-2.52 (m, 2H), 2.33-2.27 (m, 2H), 1.80-1.75 (m, 2H), 1.66-1.58 (m, 4H), 1.47 (dt, J =14.4, 7.1 Hz, 2H), 1.42-1.36 (m, 4H), 1.09-1.05 (m, 1H), 1.03-0.98 (m, 1H), 0.88 (dt, J =14.8, 7.5 Hz, 1H), 0.82 (dt, J =14.8, 7.6 Hz, 1H), 0.63-0.54 (m, 2H), 0.02 (s, 3H); ^{13}C NMR (100 MHz, CD_3OD) δ: 159.1, 120.0, 119.8, 72.1, 71.1, 71.00, 70.97, 65.4, 45.7,41.6, 33.8, 33.6, 30.4, 28.6, 27.6, 26.3, 21.0, 18.7, 18.6, 16.1, -2.8; FTIR (AgCl /thin film) 2936, 2871, 1695, 1540, 1331, 1116, 1033, 858, 810, 729, 668 cm^{-1}; HRMS (ESI+) *m/z*: $[M+H-AgNO_3]^+$ calcd. for $C_{22}H_{43}NClO_4Si^+$ 448.2650, found 448.2649.

Reactions of *trans*-cycloheptene•$AgNO_3$ complexes and Si-TCH

1,4-Diphenyl-6,7,8,9-tetrahydro-5H-cyclohepta[*d*]pyridazine(8)

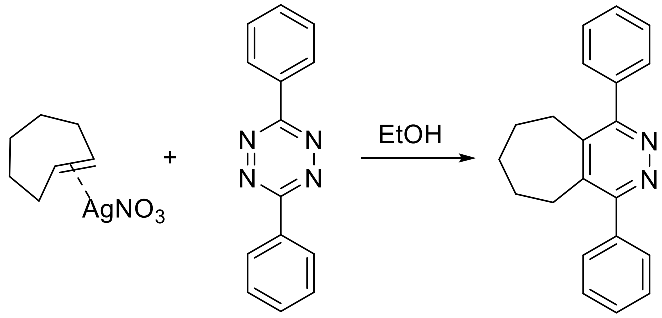

3,6-diphenyl-s-tetrazine (234 mg, 1.00 mmol, 1.20 equiv) in 10 mL methylene chloride was added to an ethanolic solution of *trans*-cycloheptene•$AgNO_3$ (0.833 mmol, in 60.0 mL EtOH, 1.00 equiv) with stirring at rt. Nitrogen evolved immediately upon mixing and a black precipitate was formed. After stirring for 30 minutes, the reaction mixture was filtered and the filtrate was concentrated down onto silica gel using a rotary evaporator and loaded onto a flash column. Column chromatography using a gradient (0-50%) of ethyl acetate in hexanes followed by 5% methanol in methylene chloride as eluents afforded 246 mg (0.820 mmol, 98%) of the title compound as a white solid. A repetition on the same scale gave 247 mg (0.825 mmol, 99%). mp 120 °C (decomposition). ^1H NMR (600 MHz, $CDCl_3$) δ: 7.48-7.42 (m, 10H), 2.83-2.78 (m, 4H), 1.93-1.86 (m, 2H), 1.72-1.62 (m, 4H); ^{13}C NMR (150 MHz, $CDCl_3$) δ: 161.7 (u), 146.6 (u), 136.8 (u), 130.2 (dn), 129.5 (dn), 128.2 (dn), 31.8 (u), 31.4(u), 25.7 (u). FTIR (NaCl/thin film) 2926, 2854, 1377, 1350, 1287, 1026, 832, 763, 704 cm^{-1}; HRMS (LIFDI-TOF) *m/z*: $[M]^+$ for $C_{21}H_2N_2^+$, 300.1621 found 300.1605.

rel-(1*R*,2*R*)-Cycloheptane-1,2-diol(10)

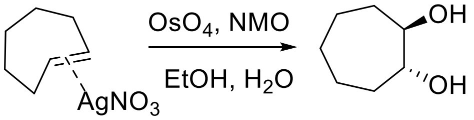

N-Methylmorpholine *N*-oxide monohydrate (115 mg, 0.852 mmol, 1.30 equiv) and 4 wt% OsO_4 in aqueous solution (125 µL, 20.5 µmol, 0.0313 equiv) were sequentially added to an ethanolic solution of *trans*-cycloheptene•$AgNO_3$ (50.0 mL of a 13.1 mM solution in EtOH, 0.654 mmol, 1.00 equiv) The resulting mixture was allowed to stir for 2 hours at rt. The mixture was diluted with saturated $NaHSO_3$ aqueous solution (50 mL) and filtered, and filtrate was extracted with ethyl acetate (8 × 25 mL), and the organics were combined, dried over $MgSO_4$ and concentrated by rotary evaporation. Purification by column chromatography with a gradient (0-5%) of methanol in methylene chloride afforded the 70 mg (0.54 mmol, 82%) of the title compound as a pale yellow solid. A repetition on the same scale gave 71 mg (0.54 mmol, 83%). mp 53-54 °C. ^1H NMR (600 MHz, $CDCl_3$) δ: 3.45-3.41 (m, 2H), 2.61 (br s, 2H), 1.90-1.84 (m, 2H), 1.69-1.64 (m, 2H), 1.54-1.42 (m, 6H); ^{13}C NMR (100 MHz, $CDCl_3$) δ: 78.1 (dn), 32.5 (u), 26.5 (u), 22.2

(u). FTIR (KBr /thin film) 3374, 2930, 2861, 1459, 1264, 1058, 1025, 561 cm^{-1}; HRMS (LIFDI-TOF) *m/z*: $[M]^+$ calcd. for $C_7H_{14}O_2^+$, 130.0988 found 130.0975.

***rel*-(1*R*,4*S*,4a*R*,9a*R*)-4,4a,5,6,7,8,9,9a-Octahydro-1*H*-1,4-methanobenzo[7]annulene(9)**

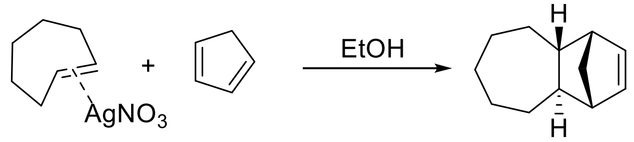

Freshly cracked cyclopentadiene (412 mg, 6.24 mmol, 10.0 equiv) was added to 50.0 mL of a 12.5 mM ethanol solution that contained *trans*-cycloheptene•$AgNO_3$ (0.624 mmol, 1.00 equiv). The mixture was allowed to stir at rt for 17 hours. The reaction mixture was then filtered and concentrated using rotary evaporator. Purification by flash chromatography using hexane afforded 83 mg (0.51 mmol, 82%) of the title compound as a clear oil. A repetition on the same scale gave 81 mg (0.50 mmol, 80%). ^{1}H NMR (400 MHz, $CDCl_3$) δ: 6.23 (dd, *J* =5.8, 3.0 Hz, 1H), 5.90 (dd, *J* =5.7, 2.9 Hz, 1H), 2.61 (m, 1H), 2.34 (m, 1H), 1.85-1.40 (m, 10H), 1.40-1.33 (m, 1H), 1.30-1.17 (m, 1H), 1.00-0.92 (m, 1H), 0.83-0.72 (m, 1H); ^{13}C NMR (100 MHz, $CDCl_3$) δ: 139.1 (dn), 131.7 (dn), 47.5 (dn), 47.3 (u), 47.2 (dn), 45.3 (dn), 44.9 (dn), 32.8 (u), 30.8 (u), 29.5 (u), 29.3 (u), 25.1 (u) ; FTIR (KBr /thin film) 2926, 2855, 1709, 1452, 1177, 1046, 657 cm^{-1};HRMS (LIFDI-TOF) *m/z*: $[M]^+$ calcd. for $C_{12}H_{18}^+$, 162.1403 found 162.1404.

6,6-diphenyl-3,3a,4,5,6,7,8,8a-octahydrosilepino[4,5-c]pyrazole(12)

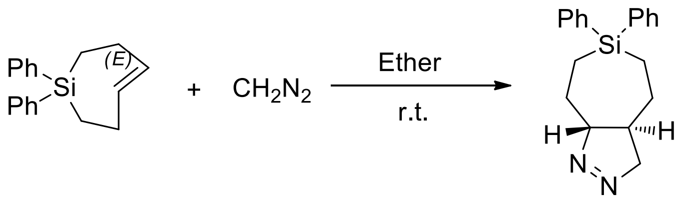

(*E*)-*Si*, *Si*-Diphenyl-5-silacycloheptene•$AgNO_3$ (0.306 mmol, 1.00 equiv) was suspended in diethyl ether (5 mL) and ammonium hydroxide (5 mL). The aqueous layer was extracted with diethyl ether (2 × 5 mL). The organics were combined, dried with anhydrous Na_2SO_4 and filtered. Diazald (656 mg, 3.06 mmol, 10.00 equiv) was taken up in 98 mL of absolute ethanol in a Lombardi flask[3]. Behind a blast shield, KOH (857 mg, 15.3 mmol, 50.0 equiv) in 0.730 mL of water was added dropwise, and the resulting diazomethane was bubbled into the flask containing the sila *trans*-cycloheptene using a stream of nitrogen. After the diazomethane solution had changed from yellow to colorless, nitrogen was bubbled for an additional 15 min. The ether solution was concentrated via rotary evaporation, and the residue was purified by flash column chromatography (10% ethyl acetate/hexane) to afford 90 mg (96% yield) of the title compound as a pale yellow oil. A similar experiment that began with 0.221 mmol of starting material gave 59 mg (87% yield) of the title compound. ^{1}H NMR (600 MHz, $CDCl_3$) δ: 7.53-7.52 (m, 2H), 7.48-7.46 (m, 2H), 7.41-7.34 (m, 6H), 4.86 (ddd, *J* =17.5, 9.3, 2.5 Hz, 1H), 3.81 (m, 1H), 3.70 (ddd, *J* =17.5, 9.6, 3.0 Hz, 1H), 3.23-3.17 (m, 1H), 2.25-2.20 (m, 1H), 1.70-1.60 (m, 2H), 1.51-1.42 (m, 4H), 1.34-1.29 (m, 1H); ^{13}C NMR (100 MHz, $CDCl_3$) δ: 136.7 (u), 136.2 (u), 134.2

(dn), 134.1(dn), 129.6 (dn), 129.5 (dn), 128.3 (dn), 128.2 (dn), 93.8 (dn), 83.2 (u), 40.6 (dn), 27.2 (u), 26.9 (u), 12.7 (u), 9.6 (u) ; FTIR (KBr /thin film) 3067, 2919, 2858, 1452, 1427, 1409, 1186, 1111, 802, 742, 726, 537, 475 cm^{-1}; HRMS (LIFDI-TOF) *m/z*: $[M]^+$ calculated for $C_{19}H_{22}N_2Si^+$ 306.1547; Found 306.1578.

(*rel*-1R,7R)-9,9-dichloro-4,4-diphenyl-4-silabicyclo[5.2.0]nonan-8-one(13)

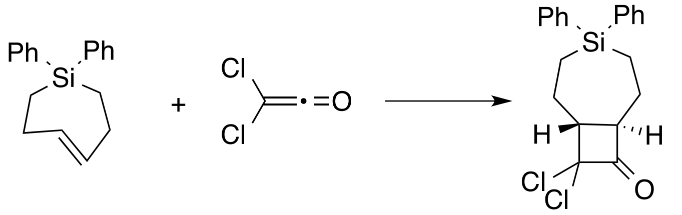

In a round-bottomed flask, (*E*)-*Si*, *Si*-diphenyl-5-silacycloheptene•$AgNO_3$ (0.31 mmol, 1.00 equiv) was combined with CH_2Cl_2 (2 mL) and conc. ammonium hydroxide (2 mL). The aqueous layer was extracted twice with CH_2Cl_2, and the organics were combined and dried over Na_2SO_4. The organic solution was purified through a plug of C2 silica gel[4] (4 cm height, 2.55 cm dimensions) eluting with 5% diethyl ether/hexane solvent (30 mL). Without evaporating to dryness, the eluate was concentrated to an approximate volume of 1 mL. The solution was added to a separate flask that had been charged with CH_2Cl_2 (1 mL) and triethylamine (1.25 mmol, 174 µl, 4.00 equiv) under an N_2 atmosphere. Dichloroacetyl chloride (0.937 mmol, 90 µL, 3.00 equiv) in CH_2Cl_2 (1 mL) was then added dropwise at rt, and the resulting mixture was allowed to stir at rt for 1.5 hours. The mixture was washed with sat. aq. $NaHCO_3$ (10 mL), and the aqueous layer was extracted with CH_2Cl_2 (3×10 mL). The organics were combined, dried, and purified by flash column chromatography (30% diethyl ether/hexane) to afford 88 mg (75% yield) of the title compound as a yellow solid A similar experiment that began with 0.29 mmol of starting material gave 84 mg (78% yield) of the title compound. mp 83-85 °C. ^1H NMR (600 MHz, $CDCl_3$) δ: 7.51-7.50 (m, 2H), 7.44-7.34 (m, 8), 3.29-3.24 (m, 1H), 2.57-2.47 (m, 2H), 2.29-2.23 (m, 1H), 2.01-1.94 (m, 1H), 1.94-1.87 (m, 1H), 1.62 (dt, J=14.8, 5.6 Hz, 1H), 1.45-1.32 (m, 3H); ^{13}C NMR (150 MHz, $CDCl_3$) δ: 195.1 (u), 136.1 (u), 135.3 (u), 134.2 (dn), 134.1 (dn), 129.9 (dn), 129.7 (dn), 128.6 (dn), 128.3 (dn), 86.9 (u), 62.3 (dn), 53.8 (dn), 24.4 (u), 21.6 (u), 11.3 (u), 10.7 (u); FTIR (KBr /thin film) 3068, 2926, 2875, 1802, 1733, 1428, 1113, 824, 701, 532 cm^{-1};HRMS (LIFDI-TOF) *m/z*: $[M]^+$ calculated for $C_{20}H_{20}Cl_2OSi^+$ 374.0655; Found 374.0668.

(3aR,8aR)-1-benzyl-6,6-diphenyl-1,3a,4,5,6,7,8,8a-octahydrosilepino[4,5-d][1,2,3]triazole(14)

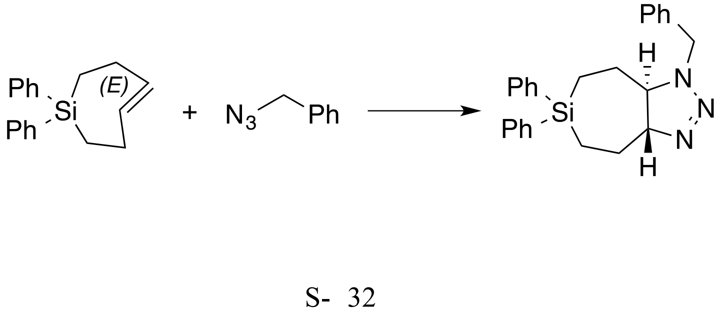

In a 20 mL vial, (*E*)-*Si*, *Si*-diphenyl-5-silacycloheptene•$AgNO_3$ (0.250 mmol, 1.0 equiv) was combined with diethyl ether (2 mL) and conc. ammonium hydroxide (2 mL). The aqueous layer was extracted with 2×2mL diethyl ether, and the organics were combined and benzyl azide (0.50 mmol, 66.4 µl, 2.00 equiv) was then added to the CH_2Cl_2 solution. The mixture was stirred at rt for 30 min. The CH_2Cl_2 solution was concentrated via rotary evaporation, and the residue was purified on silica gel (deactivated with 10% triethylamine/hexane, and rinsed with pure hexane). Flash chromatography (15% Diethyl ether/hexane) afforded 95 mg (95% yield) of the title compound as a white solid. A similar experiment that began with 0.232 mmol of starting material gave 90 mg (98% yield) of the title compound. mp 108-110°C. ^{1}H NMR (600 MHz, DMSO-d_6) δ: 7.48-7.47 (m, 2H), 7.39-7.30 (m, 8H), 7.26-7.24 (m, 3H), 7.11-7.09 (m, 2H), 4.7 (d, *J* =15.0 Hz, 1H), 4.42 (d, *J* =15.0 Hz, 1H), 3.71-3.66 (m, 1H), 2.65-2.61 (m, 2H), 2.30 (ddd, *J* =13.0, 10.4, 6.7 Hz, 1H), 1.50-1.43 (m, 1H), 1.41-1.36 (m, 1H), 1.34-1.27 (m, 3H), 1.21 (dt, *J* =15.3, 7.7 Hz, 1H); ^{13}C NMR (150 MHz, DMSO-d_6) δ: 136.5 (u), 136.1 (u), 136.0 (u), 133.8 (dn), 133.7 (dn), 129.32 (dn), 129.27 (dn), 128.44 (dn), 128.37 (dn), 128.09 (dn), 128.05 (dn), 127.38 (dn), 83.8 (dn), 64.3 (dn), 52.2 (u), 27.1 (u), 25.6 (u), 8.1 (u), 8.0 (u); FTIR (KBr /thin film) 3066, 2924, 2865, 1454, 1427, 1114, 720, 700, 536 cm^{-1}; HRMS (LIFDI-TOF) *m/z*: [M]$^+$ calculated for $C_{25}H_{27}N_3Si^+$ 397.1969; Found 397.1980.

(5aR,9aR)-3,3-diphenyl-2,3,4,5,5a,6,9,9a-octahydro-1H-6,9-methanobenzo[d]silepine(11)

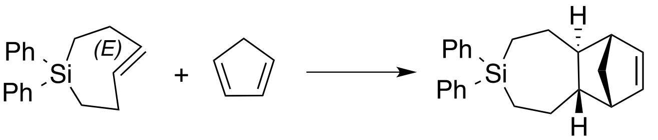

(*E*)-*Si*, *Si*-Diphenyl-5-silacycloheptene•$AgNO_3$ (0.305 mmol, 1.0 equiv) was suspended in diethyl ether (2 mL) and ammonium hydroxide (2 mL). The aqueous layer was extracted with 2×2 mL diethyl ether. The organics were combined dried with anhydrous Na_2SO_4 and filtered. Then freshly cracked cyclopentadiene (205 mg, 3.05 mmol, 10.0 equiv) was added to this ether solution of (*E*)-*Si*, *Si*-Diphenyl-5-silacycloheptene. The mixture was allowed to stir at rt for 1 hour. Afterwards, the ether solution was concentrated via rotary evaporation, the residue was purified by flash column chromatography (1% diethyl ether/hexane) to afford 100 mg (99% yield) of the title compound as a colorless oil. A repetition on the same scale gave 92 mg (91% yield). ^{1}H NMR (400 MHz, $CDCl_3$) δ: 7.50-7.48 (m, 2H), 7.46-7.43 (m, 2H), 7.36-7.29 (m, 6H), 6.11 (dd, *J* = 5.6, 3.0 Hz, 1H), 5.94 (dd, *J* = 5.6, 2.8 Hz, 1H), 2.58 (s, 1H), 2.35 (s, 1H), 2.06-1.99 (m, 1H), 1.94-1.86 (m, 1H), 1.61-1.56 (m, 1H), 1.53-1.44 (m, 2H), 1.43-1.36 (m, 1H), 1.34-1.22 (m, 4H), 1.12-1.02 (m, 1H), 0.98-0.93 (m, 1H); ^{13}C NMR (150 MHz, C_6D_6) δ: 138.4 (u), 138.3 (u), 138.2 (dn), 134.5 (dn), 134.4 (dn), 133.2 (dn), 129.34 (dn), 129.30 (dn), 128.34 (dn), 128.31 (dn), 49.6 (dn), 48.3 (dn), 48.0 (dn), 47.2 (dn) , 47.1 (u), 30.6 (u), 28.7 (u), 13.3 (u), 12.6 (u); FTIR (KBr /thin film) 3066, 2956, 2912, 1456, 1427, 1407, 1330, 1115, 795, 738, 715, 539, 482 cm^{-1}; HRMS (LIFDI-TOF) *m/z*: [M]$^+$ calculated for $C_{23}H_{26}Si^+$ 330.1798; Found 330.1798.

***rel*-2,2'-((1*R*,2*S*,3*S*,4*S*)-bicyclo[2.2.1]hept-5-ene-2,3-diyl)bis(ethan-1-ol)(15)**

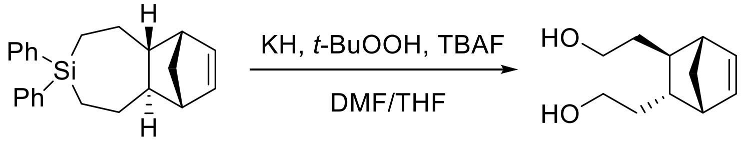

Potassium hydride (494 mg, 6.18 mmol, purchased as a suspension in mineral oil and rinsed with hexane prior to use, 12.0 equiv) was suspended in anhydrous DMF (3 mL), and the flask cooled by an ice bath (0 °C). *tert*-Butyl hydroperoxide (1.13 mL, 5.5 M in decane, 6.20 mmol, 12.0 equiv) was added dropwise. The mixture was allowed to warm to rt. *rel*-(5a*S*,6*R*,9*S*,9a*S*)-3,3-diphenyl-2,3,4,5,5a,6,9,9a-octahydro-1*H*-6,9-methanobenzo[*d*]silepine (170 mg, 0.515 mmol, 1.00 equiv) in anhydrous DMF (4 mL) was added to the mixture dropwise. After 10 min, *n*-Bu_4NF solution (1.0 M in THF, 2.10 mL, 2.10 mmol, 4.08 equiv) was added. The reaction was heated at 70 °C overnight. After the mixture was cooled to rt, excess sodium thiosulfate pentahydrate (3.00 g, 12.1 mmol) was added. After stirring for 30 min, the resulting mixture was filtered and solvent was removed by rotary evaporator. The solid residue was dissolved by methylene chloride and the resulting solution was filtered, and concentrated by rotary evaporation. Purification by column chromatography with a gradient (30%-100%) of ethyl acetate in hexanes yielded title compound (71 mg, 0.396 mmol, 76%) as a white solid, mp 61-63 °C. ^{1}H NMR (600 MHz, $CDCl_3$) δ: 6.18 (dd, *J* =5.8, 3.1 Hz, 1H), 5.99 (dd, *J* =5.8, 2.9 Hz, 1H), 3.80-3.60 (m, 4H), 2.73 (s, 1H), 2.49 (s, 1H), 1.92 (br s, 2H), 1.76-1.62 (m, 3H), 1.49-1.36 (m, 4H), 1.06-1.02 (m, 1H); ^{13}C NMR ($CDCl_3$, 100 MHz) δ: 137.8 (dn), 133.8 (dn), 62.31 (u), 62.28 (u), 47.4 (dn), 46.5 (u), 45.9 (dn), 43.5 (dn), 42.4 (dn), 39.3 (u), 37.8 (u).FTIR (KBr /thin film) 3322, 2959, 2929, 2873, 1454, 1339, 1053, 1011, 914, 714 cm^{-1}; HRMS (LIFDI-TOF) *m/z*: $[M]^+$ calculated for $C_{11}H_{18}O_2^+$ 182.1301; Found 182.1286.

Transformations of Si-TCH derivatives

(*E*)-N-(7-(1-(difluoroboranyl)-3,5-dimethyl-1H-pyrrol-2-yl)-7-(3,5-dimethyl-2H-pyrrol-2-ylidene)heptyl)-4-(1-methyl-2,3,6,7-tetrahydro-1H-silepin-1-yl)butanamide•$AgNO_3$(AgSiTCH-BODIPY)

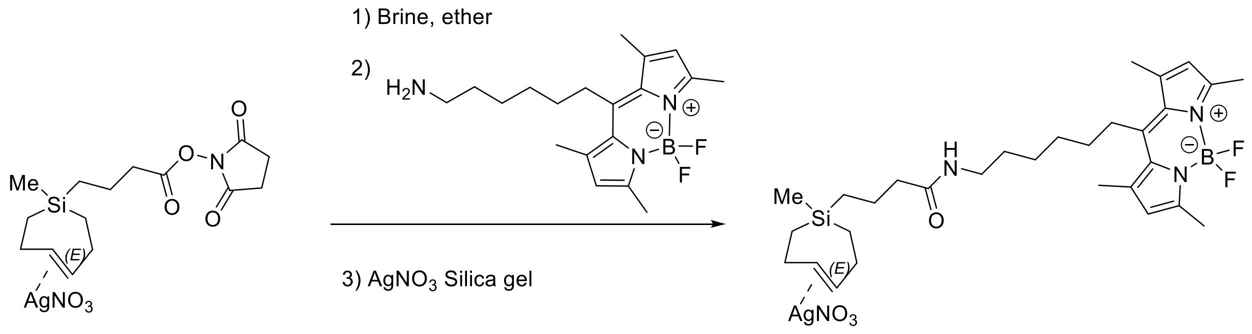

(*E*)2,5-dioxopyrrolidin-1-yl 4-(1-methyl-2,3,6,7-tetrahydro-1*H*-silepin-1-yl)butanoate•$AgNO_3$ (0.110 mmol, 7.20 equiv) was treated with 1.5 mL CH_2Cl_2 and 2.0 mL brine. CH_2Cl_2 layer was separated. The aqueous layer was extract with 2×1.5 mL CH_2Cl_2. The organic layer was combined and dried with Na_2SO_4 and filtered. With care not to concentrate to dryness, the solution was concentrated down to approximately 1.5 mL via rotary evaporation. (Z)-7-(1-(difluoroboryl)-3,5-dimethyl-1H-pyrrol-2-yl)-7-(3,5-dimethyl-2H-pyrrol-2-ylidene)heptan-1-amine (BODIPY amine, 5.3 mg, 0.015 mmol, 1.00 equiv) and triethylamine (41 mg, 0.40 mmol, 26 equiv) were added. The reaction was stirred at rt for 1.5 hour. The reaction mixture was quickly loaded on to flash chromatography, (15%-50% ethyl acetate/hexane) afforded the (*E*)-N-(7-(1-(difluoroboranyl)-3,5-dimethyl-1H-pyrrol-2-yl)-7-(3,5-dimethyl-2H-pyrrol-2-ylidene)heptyl)-4-(1-methyl-2,3,6,7-tetrahydro-1H-silepin-1-yl)butanamide as a solution in ca. 50% ethyl acetate/hexane. The solution was allowed to flow through a plug of 4 g silica gel (impregnated with 10% w/w $AgNO_3$), which was packed in an 11g Biotage® SNAP cartridge on top of a bed of unmodified silica gel. The SNAP cartridge was eluted with 100 mL 50% ethyl acetate/hexane over 2 hours. Then the silica gel column was washed with 100 mL 60% ethyl acetate/hexane, followed by 100 mL EtOH to give the desired EtOH solution of (*E*)-N-(7-(1-(difluoroboranyl)-3,5-dimethyl-1H-pyrrol-2-yl)-7-(3,5-dimethyl-2H-pyrrol-2-ylidene)heptyl)-4-(1-methyl-2,3,6,7-tetrahydro-1H-silepin-1-yl)butanamide•$AgNO_3$. The ethanol solution was concentrated via rotary evaporation, affording a sticky orange semisolid. ^1H NMR analysis with an internal standard (mesitylene) indicated that the yield of the title compound was 0.0104 mmol (68% yield).

^1H NMR (600 MHz, CD_3OD) δ: 6.15 (s, 2H), 5.59-5.49 (m, 2H), 3.19 (t, *J* = 6.8 Hz, 2H), 3.00-2.98 (m, 2H), 2.52-2.46 (m, 2H), 2.44 (s, 12 H), 2.29-2.23 (m, 2H), 2.19 (t, *J* = 7.2 Hz, 2H), 1.66-1.51 (m, 8H), 1.44-1.39 (m, 2H), 1.04-0.94 (m, 2H), 0.86-0.76 (m, 2H), 0.58-0.49 (m, 2H), -0.011 (s, 3H); ^{13}C NMR (100 MHz, CD_3OD): 176.0 (u), 154.8 (u), 148.2 (u), 142.2 (u), 132.5 (u), 122.0 (dn), 120.1 (dn), 119.8 (dn), 40.7 (u), 40.0 (u), 32.9 (u), 30.8 (u), 30.2 (u), 29.1 (u), 28.6 (u), 28.5 (u), 27.5 (u), 21.6 (u), 18.6 (u), 16.5 (dn), 16.2 (u), 14.3 (dn), -2.9 (dn). HRMS (ESI+) *m/z*: $[M\text{-}AgNO_3\text{-}F]^+$ calcd. for $C_{30}H_{46}BFN_3OSi^+$, 522.3482 found 522.3482.

(*E, eq*)-1,1-Dimethyl-2,3,6,7-tetrahydro-1H-silepin-3-ol (equatorial isomer)

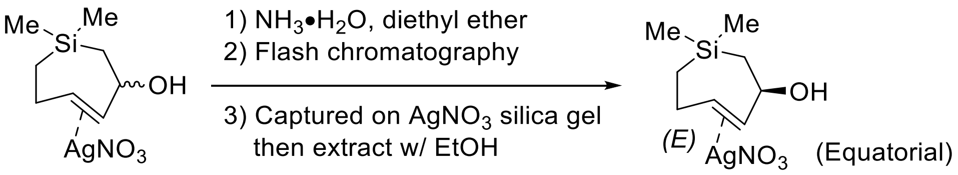

(*E*)-1,1-dimethyl-2,3,6,7-tetrahydro-1H-silepin-3-ol•$AgNO_3$ (0.812 mmol) was treated with 10 mL conc. ammonia solution and 10 mL diethyl ether. The aqueous layer was extract with 3×10 mL diethyl ether. Organics were combined and dried over $MgSO_4$, filtered and concentrated via rotary evaporation. Purification by column chromatography (1-4% ethyl acetate/ hexane) gave the equatorial diastereomer of (*E*)-1,1-dimethyl-2,3,6,7-tetrahydro-1H-silepin-3-ol and its *cis*-isomer as colorless mixture. The mixture was dissolved in 100 mL 45% diethyl ether/hexane. The solution was allowed to flow for 2 hours through a plug of 2.2 g of $AgNO_3$ impregnated silica gel (10% w/w $AgNO_3$), which was packed in an 11g Biotage® SNAP cartridge on top of a bed of unmodified silica gel. The cartridge was then eluted with 100 mL 45% diethyl ether/hexane followed by 100 mL ethanol to give an ethanol solution of (*E*)-1,1-dimethyl-2,3,6,7-tetrahydro-1H-silepin-3-ol•$AgNO_3$ (major). The ethanol solution was concentrated via rotary evaporation to give the title compound (*E, eq*)-1,1-dimethyl-2,3,6,7-tetrahydro-1H-silepin-3-ol•$AgNO_3$ (0.205 mmol) as white semisolid. ^{1}H NMR (600 MHz, CD_3OD) δ: 5.72 (ddd, J = 16.0, 10.3, 5.3 Hz, 1H), 5.49 (dd, J = 16.9, 9.0 Hz, 1H), 4.53 (td, J = 9.1, 6.4 Hz, 1H), 2.48-2.37 (m, 2H), 1.38 (dd, J = 14.1, 6.2 Hz, 1H), 0.97 (ddd, J = 14.7, 10.6, 6.1 Hz, 1H), 0.90-0.84 (m, 2H); 0.08 (s, 3H), 0.00 (s, 3H); ^{13}C NMR (150 MHz, CD_3OD) δ: 124.2 (dn), 114.7 (dn), 73.4 (dn), 29.8 (u), 26.6 (u), 18.9 (u), -0.96 (dn), -0.13 (dn); FTIR (AgCl/thin film) 3246, 2946, 1762, 1652, 1559, 1316, 1005, 862, 830, 785; HRMS (CI+) *m/z*: $[M-AgNO_3+H]^+$ calcd. for $C_8H_{17}OSi^+$, 157.1043 found 157.1055.

(*E*)-1,1-Dimethyl-2,3,6,7-tetrahydro-1 H-silepin-3-yl benzylcarbamate (18)

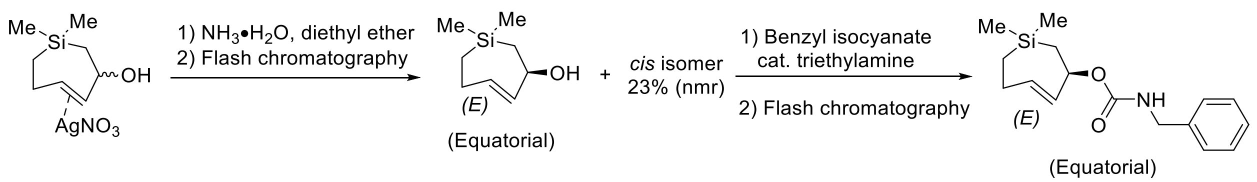

(*E*)-1,1-dimethyl-2,3,6,7-tetrahydro-1H-silepin-3-ol•$AgNO_3$ (0.832 mmol) was treated with conc. ammonium hydroxide (10 mL) and diethyl ether (10 mL). The aqueous layer was extracted with 3×10 mL diethyl ether. The organics were combined and dried over $MgSO_4$, filtered and concentrated via rotary evaporation. Purification by column chromatography (1-4% ethyl acetate/ hexane) gave (*E*)-1,1-dimethyl-2,3,6,7-tetrahydro-1H-silepin-3-ol (equatorial) and its cis

isomer as a colorless mixture (78 mg, 23% cis by nmr). To this mixture in dry methylene chloride (4 mL) was added benzyl isocyanate (0.61 mL, 661 mg, 4.96 mmol, 10.0 equiv.) and triethylamine (14μL, 0.10 mmol, 0.20 equiv). The solution was stirred under a nitrogen atmosphere at rt for 6 hours. The volatiles were removed. Purification by column chromatography (1-4% ethyl acetate/ hexane) gives the title compound (90 mg, 0.31 mmol, 81% yield based on theoretical yield of the equatorial isomer) as colorless oil. ^{1}H NMR (400 MHz, CD_3OD) δ: 7.32-7.21 (m, 5H), 5.94-5.85 (m, 1H), 5.55 (dd, J = 17.2, 9.1 Hz, 1H), 5.41 (td, J = 9.3, 6.2 Hz, 1H), 4.27 (s, 2H), 2.42 (td, J = 13.6, 3.7 Hz, 1H), 2.22-2.14 (m, 1H), 1.41 (dd, J = 14.0, 5.9 Hz, 1H), 1.00-0.90 (m, 1H), 0.88-0.78 (m, 2H), 0.06 (s, 3H), -0.06 (s, 3H); ^{13}C NMR (100 MHz, CD_3OD) δ: 159.0 (u), 140.7 (u), 137.0 (dn), 131.4 (dn), 129.5 (dn), 128.2 (dn), 128.1 (dn), 76.8 (dn), 45.3 (u), 28.3 (u), 25.8 (u), 21.5 (u), -0.7 (dn), -1.2 (dn); FTIR (NaCl /thin film) 3335, 2950, 2924, 1697, 1522, 1497, 1251, 838, 698 cm^{-1}; HRMS (ESI+) *m/z*: $[M+H]^+$ calcd. For $C_{16}H_{24}NO_2Si^+$, 290.1576 found 290.1566.

(Z)-7-(1-(difluoroboranyl)-3,5-dimethyl-1H-pyrrol-2-yl)-7-(3,5-dimethyl-2H-pyrrol-2-ylidene)heptan-1-amine (16)

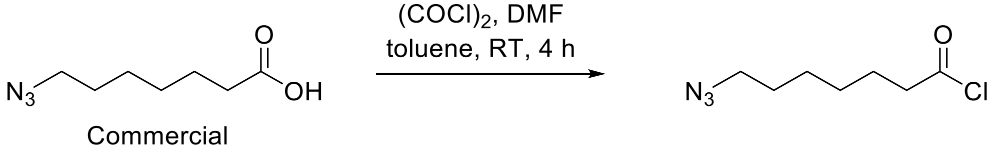

To a solution of 7-azidoheptanoic acid (5.0 g, 29 mmol, 1.00 equiv) in toluene (50 mL) was added oxalyl chloride (3.78 mL, 43.8 mmol, 1.51 equiv) followed by DMF (0.1 mL). The mixture was stirred at room temperature for 4 h and then concentrated under reduced pressure. The crude 7-azidoheptanoyl chloride was used directly in the subsequent step.

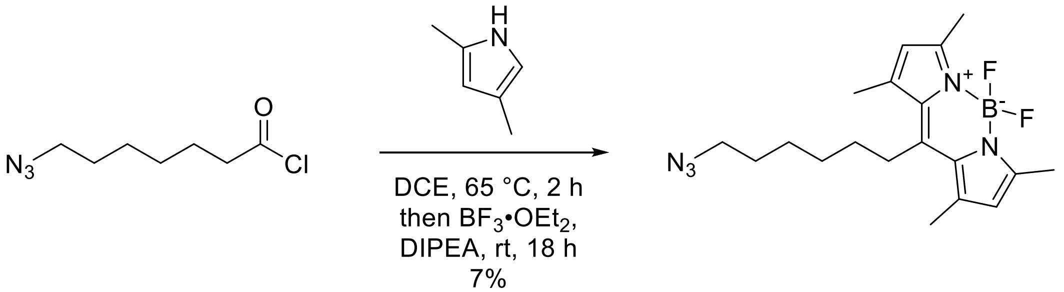

To a solution of crude 7-azidoheptanoyl chloride (4.0 g, 23 mmol, 1.00 equiv) in 1,2-dichloroethene (60 mL) was added 2,4-dimethylpyrrole (5.07 mL, 49.1 mmol, 2.13 equiv). The reaction was stirred at 65 °C for 2 h and then cooled to room temperature. To the reaction was added BF_3•OEt_2 (16.58 g, 118.8 mmol, 5.16 equiv) was added dropwise followed by the addition of DIPEA (16.77 mL, 93.46 mmol, 4.06 equiv) dropwise and the mixture was degassed by bubbling argon through the mixture for 15 minutes and then stirred at room temperature for 18 h. The reaction was diluted with water and extracted with CH_2Cl_2 (2x). The organic fractions were combined, washed with water and brine, dried with Na_2SO_4, filtered and concentrated under reduced pressure. The crude residue was purified by silica gel chromatography (EtOAc/Hexane) to afford BODIPY-N_3 (650 mg, 7%) as a red solid. ^{1}H NMR (400MHz, $CDCl_3$) δ: 6.05 (s, 2H), 3.28 (t, J = 6.8 Hz, 2H), 3.03 - 2.83 (m, 2H), 2.51 (s, 6H), 2.40 (s, 6H), 1.71 - 1.57 (m, 4H), 1.57 - 1.37 (m, 4H);^{13}C NMR (101MHz, $CDCl_3$) δ: 153.80, 146.19, 140.17, 131.37, 121.58, 51.32, 31.71, 29.86, 28.85, 28.25, 26.56, 16.36, 14.41 (t, J = 2.9 Hz); HRMS calcd for $C_{19}H_{26}BFN_5$ $[M-F]^+$ 454.2265, found 454.2258.

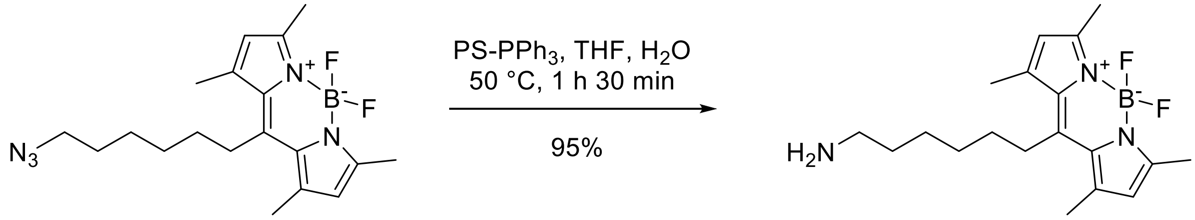

To a 2-dram vial containing BODIPY-N3 (150 mg, 0.40 mmol) and polymer-supported triphenylphosphine (574 mg, 1.4 – 2.0 mmol/g loading, Alfa Aesar) was added THF (2 mL) and water (0.1 mL). The reaction was heated to 50 °C for 1.5 hours and then cooled to room temperature. The reaction mixture was filtered through celite and concentrated under reduced pressure to yield a dark red solid (132 mg, 95% yield) that was used directly without additional purification. ^{1}H NMR (400 MHz, $CDCl_3$) δ: 6.05 (s, 2H), 3.00 - 2.85 (m, 2H), 2.71 (app. br. s., 2H), 2.51 (s, 6H), 2.41 (s, 6H), 1.70 - 1.57 (m, 4H), 1.56 - 1.44 (m, 4H), 1.44 - 1.34 (m, 2H); ^{13}C NMR (101 MHz, $CDCl_3$) δ: 153.75, 146.45, 140.21, 131.40, 121.55, 41.98, 33.48, 31.88, 30.27, 28.41, 26.71, 16.38, 14.43 (t, J = 2.9 Hz); HRMS (ESI+) *m/z*: $[M+H]^+$ calcd. For $C_{19}H_{29}BF_2N_3^+$, 348. 2417 found 348.2418.

General Procedure for the stopped-flow kinetic analysis of Si-TCH and 3,6-diphenyl-*s*-tetrazine.

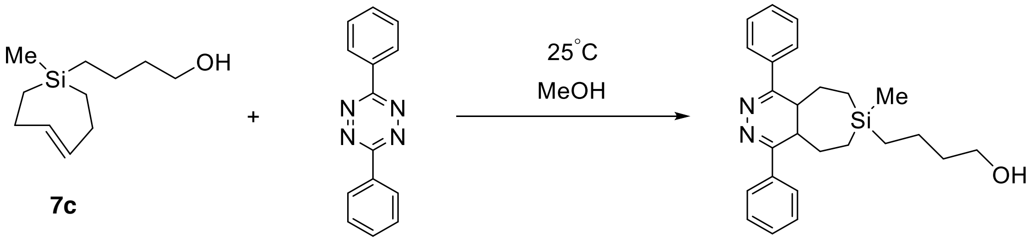

7c

The reaction between Si-TCH **7c (**Ag-free) and 3,6-diphenyl-*s*-tetrazine was monitored by SX 18MV-R stopped-flow spectrophotometer (Applied Photophysics Ltd.) at 295 nm under pseudo-first order conditions. The Si-TCH (200 µM in methanol, concentration of *trans*-isomer) and tetrazine (20 µM in methanol) were mixed in a stopped-flow spectrophotometer resulting in a final concentration of 100 µM Si-TCH and 10 µM tetrazine. Analysis was carried out in triplicate at 298 K. For each run, data was collected over 20 seconds. k_{obs} was determined by nonlinear regression analysis of the data points using Prism software (v. 6.00, GraphPad Software Inc.). The average from of triple sets of measurements was reported as k_2=4360 (+/-430) $M^{-1}s^{-1}$.

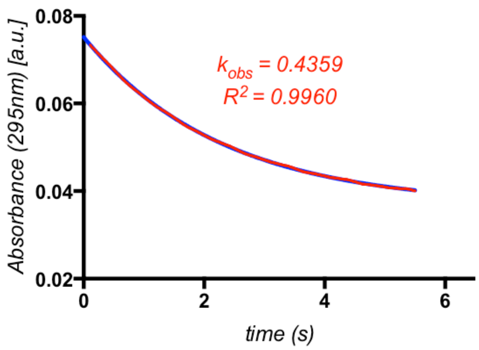

Fig S6. Reaction of **7c** with 3,6-diphenyl-*s*-tetrazine at 298 K monitored at 295 nm.

General Procedure for the stopped-flow kinetic analysis of sTCO (anti-isomer), Si-TCH and tetrazine TAMRA (23).

The kinetics for the reactions between sTCO or Si-TCH and the tetrazine TAMRA were measured under pseudo-first order conditions with 10 equivalents of sTCO or Si-TCH, in water/methanol (9:1) at 298 K by following the fluorescence increase of the tetrazine TAMRA at 576 nm over time using an SX 18MV-R stopped-flow spectrophotometer (Applied Photophysics Ltd.). The excitation wavelength of the tetrazine TAMRA was 556 nm.

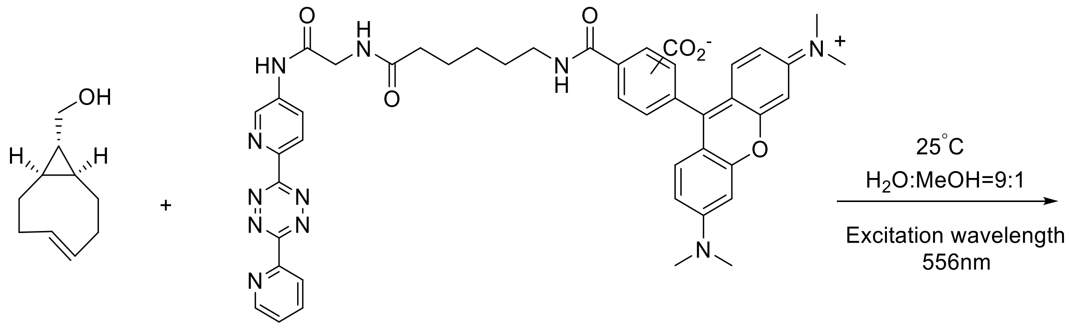

For the reaction between sTCO and tetrazine TAMRA, solutions were prepared for the sTCO (100 µM in 80% water/20% MeOH) and the tetrazine TAMRA (10 µM in water) and thermostatted in the syringes of the spectrophotometer before measuring. An equal volume of each was mixed by the stopped flow device resulting in a final concentration of 5 µM tetrazine TAMRA and 50 µM sTCO. Data was recorded for 0.05 seconds and performed in triplicate at 298 K. k_{obs} was determined by nonlinear regression analysis of the data points using Prism software (v. 6.00, GraphPad Software Inc.). Triplicate measurements were performed on two independent samples. The mean k_2 was measured to be 8,340,000 (+/- 667,000) $M^{-1}s^{-1}$.

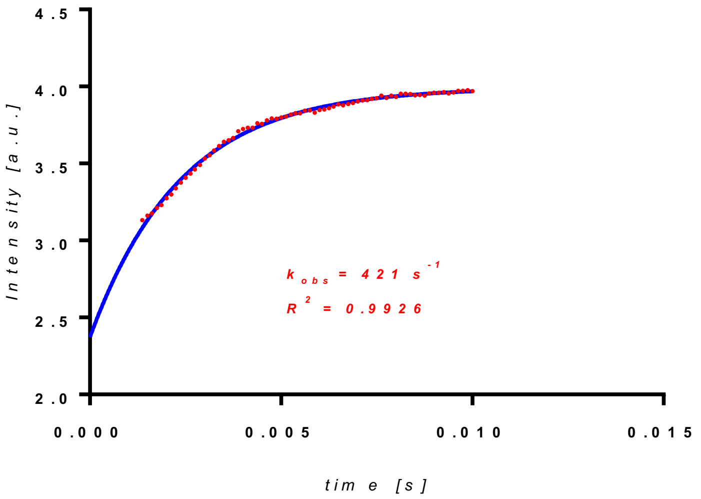

Fig.S7. Stopped-flow monitored reaction of tetrazine-TAMRA (5 µM) with s-TCO (50 µM) in 9:1 water: MeOH. Triplicate measurements for two independent samples were measured.

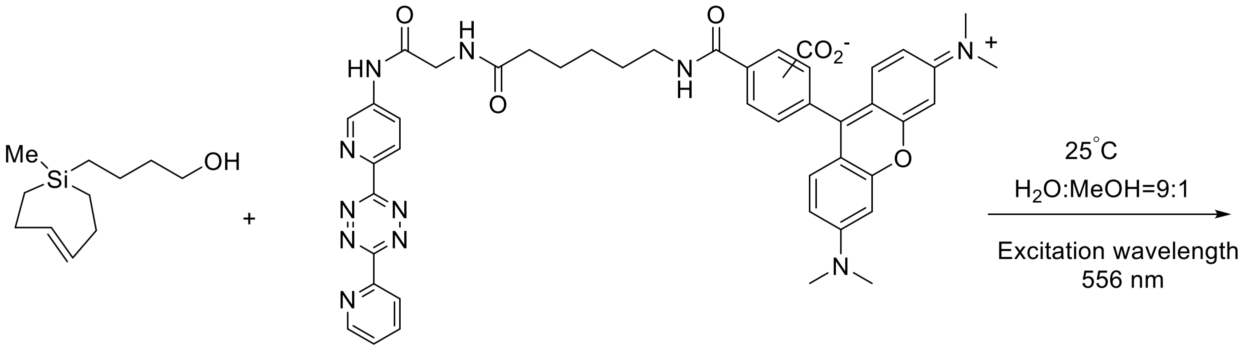

7c

The general procedure for the stopped-flow analysis of Si-TCH (**7c**) was followed using Si-TCH (**7c**)(60 µM, 80 µM, 100 µM and 120 µM in 80% water/methanol) and tetrazine TAMRA (6 µM, 8 µM, 10 µM and 12 µM in water). Final concentrations were 3 µM, 4 µM, 5 µM and 6 µM for the tetrazine TAMRA and 30 µM, 40 µM, 50 µM and 60 µM for the Si-TCH (concentration of *trans*-isomer). The k_{obs} was determined by nonlinear regression analysis of the data points using Prism software (v. 7.00, GraphPad Software Inc.). Triplicate measurements for two independent samples at each concentration were measured, and the average of the observed rates *k*' were plotted against the concentration of Si-TCH to obtain the bimolecular rate constant k_2 from the slope of the plot. This mean k_2 was measured as 11,400,000 (+/-1,100,000) $M^{-1}s^{-1}$.

a)

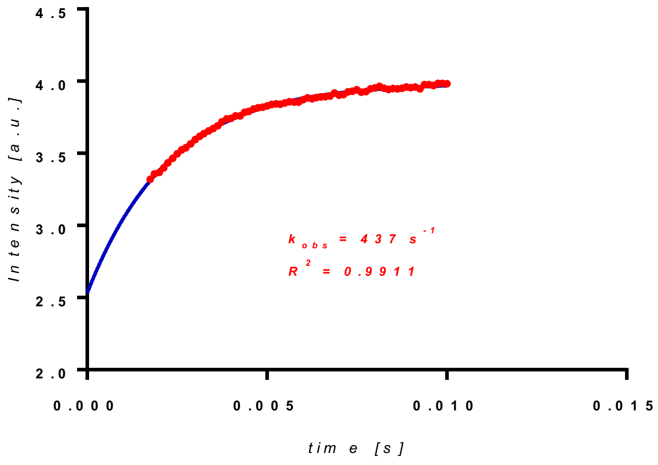

b)

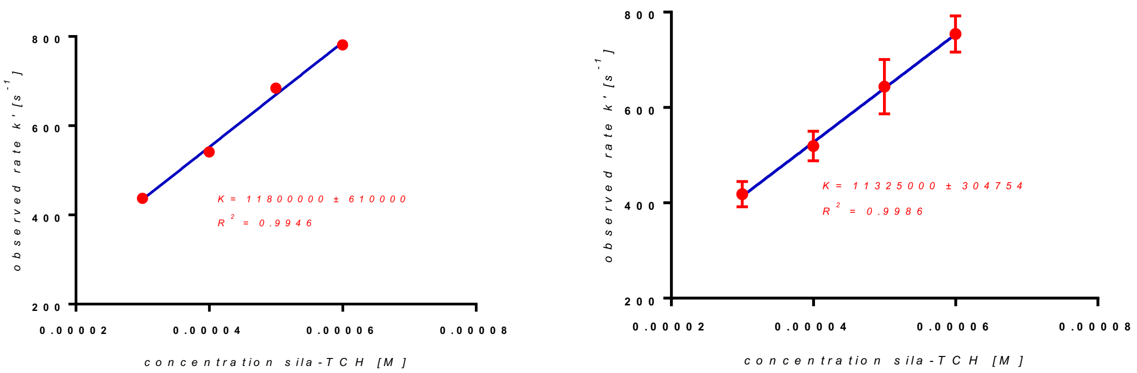

Fig S8. Determination of the bimolecular rate constant *k* from the stopped-flow monitored reaction of Si-TCH and tetrazine TAMRA. (a) The exponential plot of the reaction of tetrazine TAMRA (final concentration 3 µM) with 10 equivalents of Si-TCH (final concentration 30 µM) at 298 K at 556 nm excitation wavelength. Similar data sets were collected at 4 µM, 5 µM and 6 µM in tetrazine-TAMRA, and at 40 µM, 50 µM, and 60 µM in Si-TCH. (b) Triplicate measurements for two independent samples at each concentration were measured, and the observed rates k_{obs} were plotted against the concentration of Si-TCH to obtain the bimolecular rate constant k_2 from the slope of the plot.

General Procedure for the stopped-flow kinetic analysis of (*E*)-1,1-dimethyl-2,3,6,7-tetrahydro-1 H-silepin-3-yl benzylcarbamate (18) and 3,6-pyridyl -*s*-tetrazine.

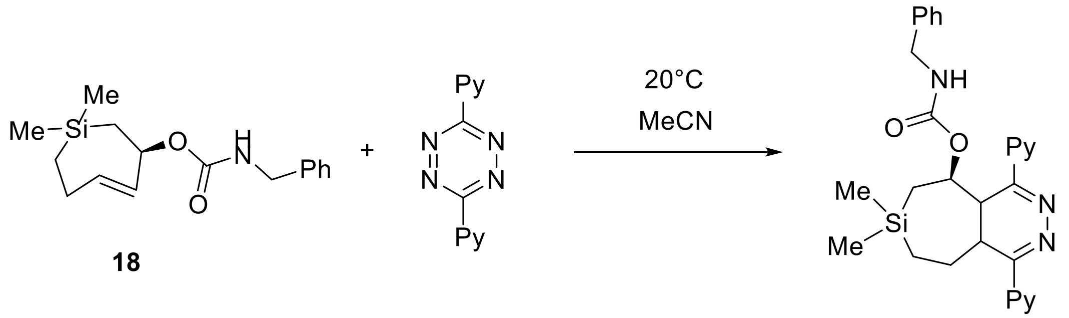

The reaction between (*E*)-1,1-dimethyl-2,3,6,7-tetrahydro-1 H-silepin-3-yl benzylcarbamate (18) and 3,6-dipyridyl-*s*-tetrazine was monitored using a SX 18MV-R stopped-flow spectrophotometer (Applied Photophysics Ltd.) at 290 nm under pseudo-first order conditions in acetonitrile at 20 °C. The Si-TCH (1000 µM in MeCN) and tetrazine (100 µM in MeCN) were mixed using the stopped flow instrument resulting in final concentrations of 500 µM Si-TCH and 50 µM tetrazine. Analysis was carried out in triplicate at 20 °C. For each run, data was collected over 200 seconds. The k_{obs} was determined by nonlinear regression analysis of the data points using Prism software (v. 6.00, GraphPad Software Inc.). From the average of triplicate sets of measurements a rate of k_2= 240 (+/- 30) $M^{-1}s^{-1}$ was determined.

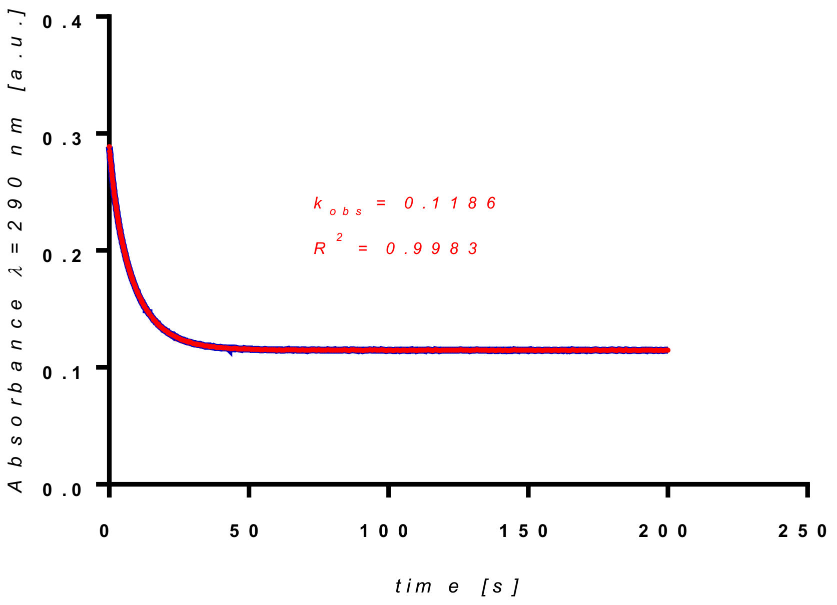

Fig S9. The reaction of (*E*)-1,1-dimethyl-2,3,6,7-tetrahydro-1 H-silepin-3-yl benzylcarbamate with 3,6-dipyridyl-*s*-tetrazine at 20 °C monitored at 290 nm.

General Procedure for the UV-Vis kinetic analysis of (*E*)- 3-(1,3,6-trimethyl-2,3,6,7-tetrahydro-*1* H-silepin-1-yl)propan-1-ol (7d) and 3,6-phenyl -*s*-tetrazine.

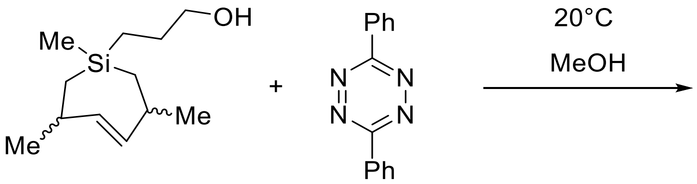

The reaction between (*E*)- 3-(1,3,6-trimethyl-2,3,6,7-tetrahydro-*1* H-silepin-1-yl)propan-1-ol and 3,6-phenyl-*s*-tetrazine was monitored using a UV-Vis spectrophotometer (HP8453) at 295 nm under pseudo-first order conditions in acetonitrile at 20 °C. The Si-TCH (0.7 mL, 1000 µM in methanol) was added to the tetrazine (0.7 mL, 100 µM in methanol) and quickly mixed in a 1 mL cuvette, resulting in final concentrations of 500 µM Si-TCH and 50 µM tetrazine. Analyses was carried out in triplicate at 20 °C. For each run, data was collected over 1100 seconds. k_{obs} was determined by nonlinear regression analysis of the data points using Prism software (v. 6.00, GraphPad Software Inc.). The average of triple sets of measurements was reported as k_2= 22 (+/- 4) $M^{-1}s^{-1}$.

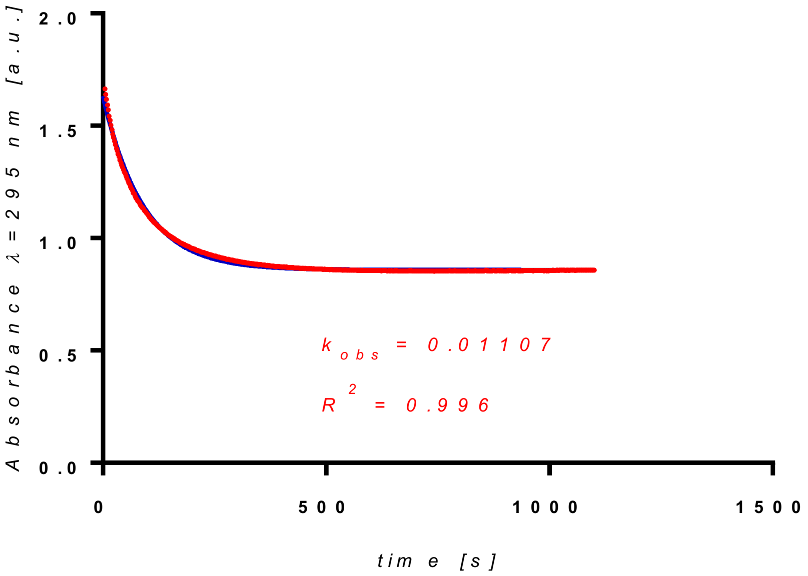

Fig.S10. Reaction of (*E*)- 3-(1,3,6-trimethyl-2,3,6,7-tetrahydro-*1* H-silepin-1-yl)propan-1-ol and 3,6-phenyl -*s*-tetrazine at 20 °C in methanol monitored at 295 nm.

General Method for *in vitro* Si-TCH / GFP-Tet-v.2.0 kinetics

The reaction between Si-TCH (**7c**) and GFP-tetrazine 2.0 was measured under pseudo-first order conditions using an SX 18MV-R stopped-flow spectrophotometer (Applied Photophysics Ltd.). A sample of 100 nM GFP-tetrazine 2.0 (Excitation 488 nm, Emission 506 nm) in PBS buffer was prepared for a final concentration of 50 nM. Si-TCH solutions (1.00, 1.98, 2.97, 3.92 µM) were prepared in 95% PBS/methanol from stock solutions (19.9, 39.6, 59.4, 78.5 µM in methanol). The final concentrations of **7c** were 0.500, 0.997, 1.49 and 1.96 µM. Data collection was taken in 0.02 or 0.04 sec increments for about 20 s to 40 s for each trial. The observed rate for each measurement, k_{obs} (Fig.S11), was determined by nonlinear regression analysis using Prism software resulting in rates constants of 0.0947, 0.239, 0.366 and 0.459 s^{-1} respectively and were then plotted against final concentration to determine the bimolecular rate constant k_2 (Fig.S12) of 250000 (+/- 15000) $M^{-1}s^{-1}$ from the slope of the plot.

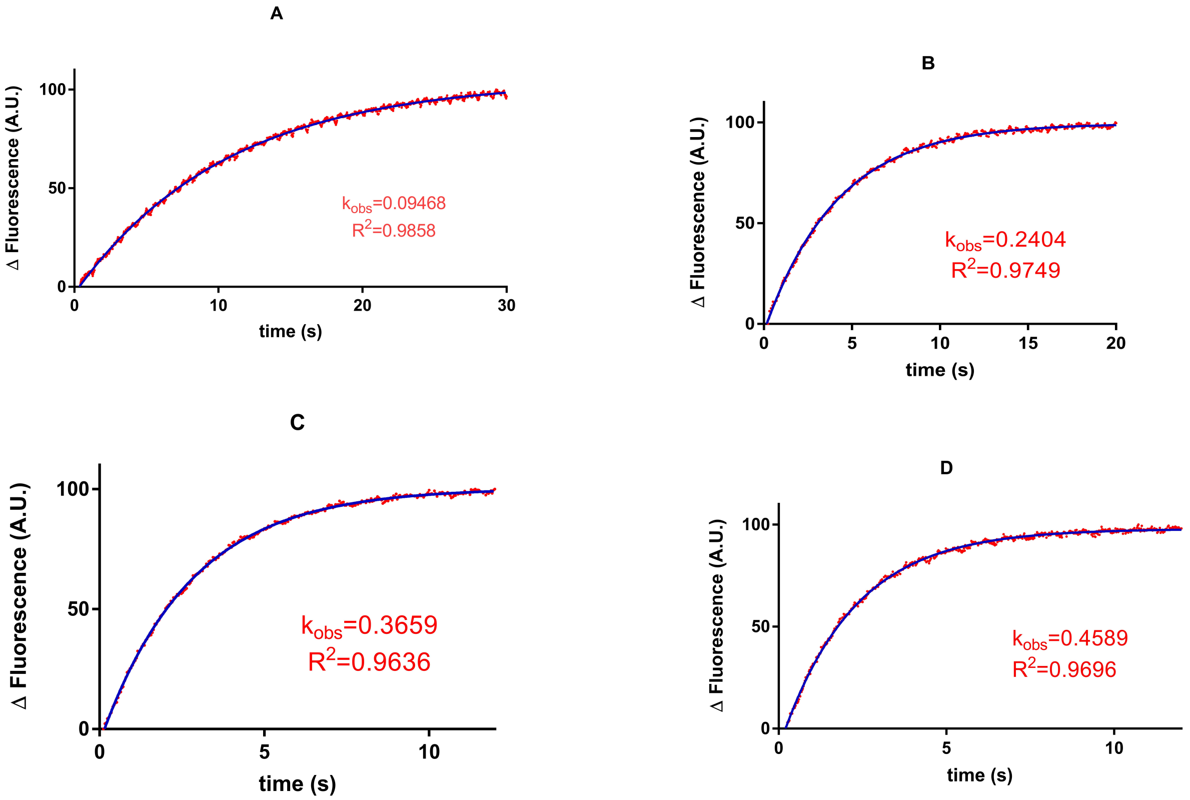

Fig. S11. Fluorescence kinetics of Si-TCH (**7c**) and GFP-tetrazine 2.0. After mixing, the final tetrazine concentration was 50 nM and the final Si-TCH concentrations are as follows: 0.500, 0.997, 1.49 and 1.96 µM. Raw data points and the fit curve for a pseudo first order rate equation calculated on Prism software are shown.

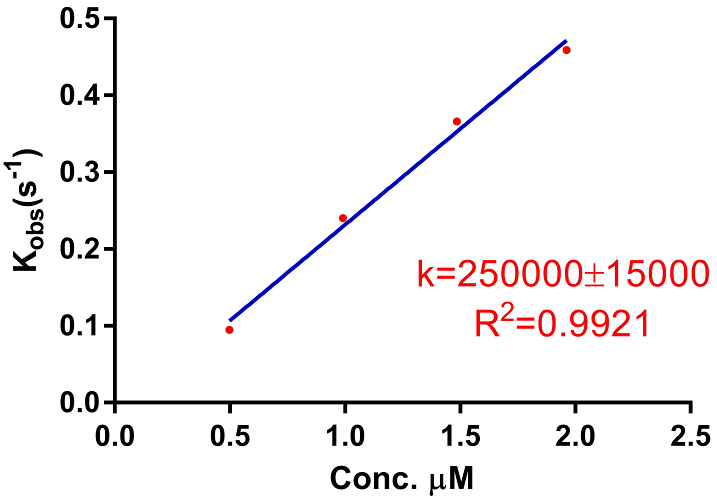

Fig.S12. in vitro bimolecular rate determination of Si-TCH (**7c**) solutions reacted with GFP-tetrazine 2.0 by plotting kobs vs final Si-TCH concentration

General Method for *in vivo* Si-TCH / GFP-Tet-v.2.0 kinetics

Purified sfGFP-TetV2.0 for *in vitro* kinetics

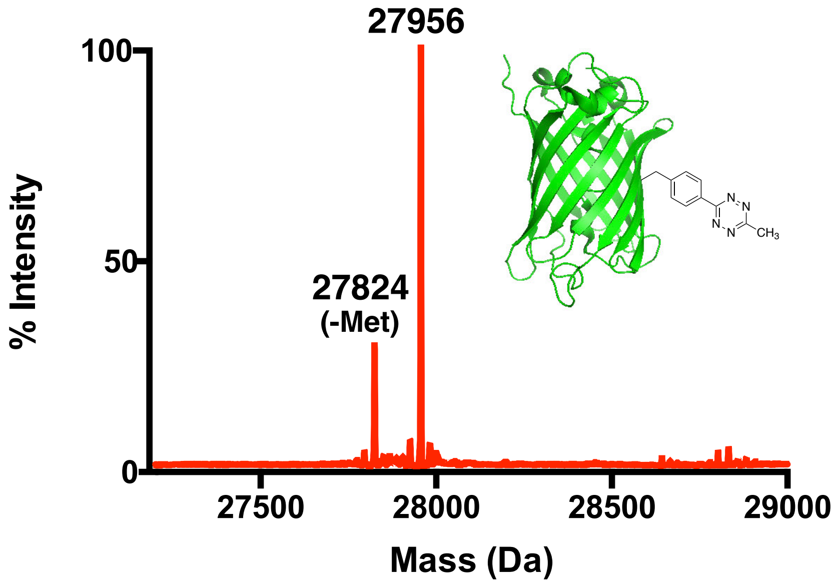

Fig.S13. Positive mode UPLC-ESI-MS deconvoluted mass spectrum of sfGFP-TetV2.0 with a major peak at 27956 ± 1 Da and a minor peak at 27824 ± 1 Da (loss of methionine)

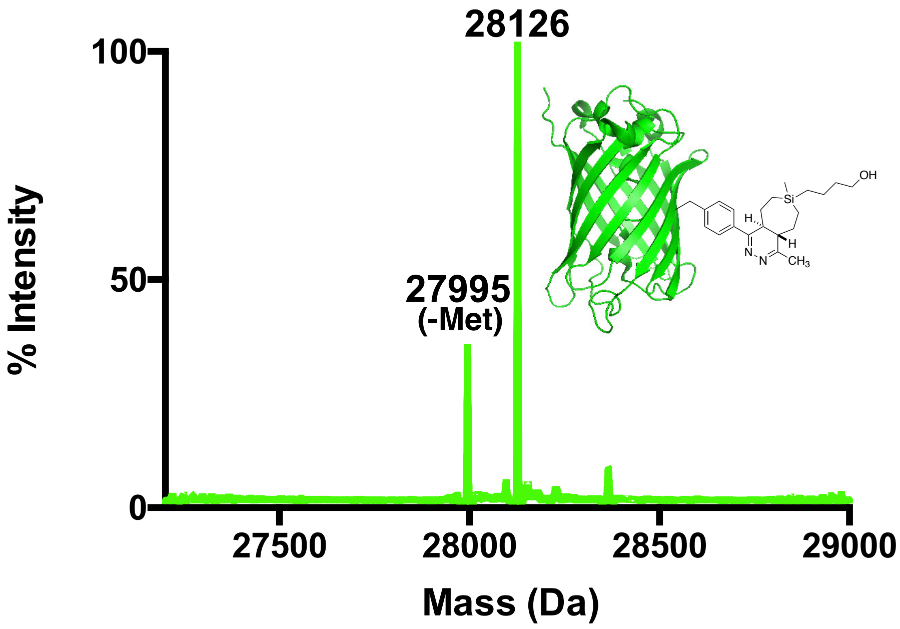

Fig. S14. Positive mode UPLC-ESI-MS deconvoluted mass spectrum of sfGFP-TetV2.0 with a major peak at 28126 $\pm$ 1 Da and a minor peak at 27995 $\pm$ 1 Da (loss of methionine). This shows an expected molecular weight difference of 170 Da at both major and minor peaks, indicating a successful conjugation with **7c**. Analysis was performed on the Xevo GS-2 QTof (Waters Corp.) system and MaxEnt was used for deconvolution of spectra

sfGFP-TetV2.0 used for *in vivo* experiments

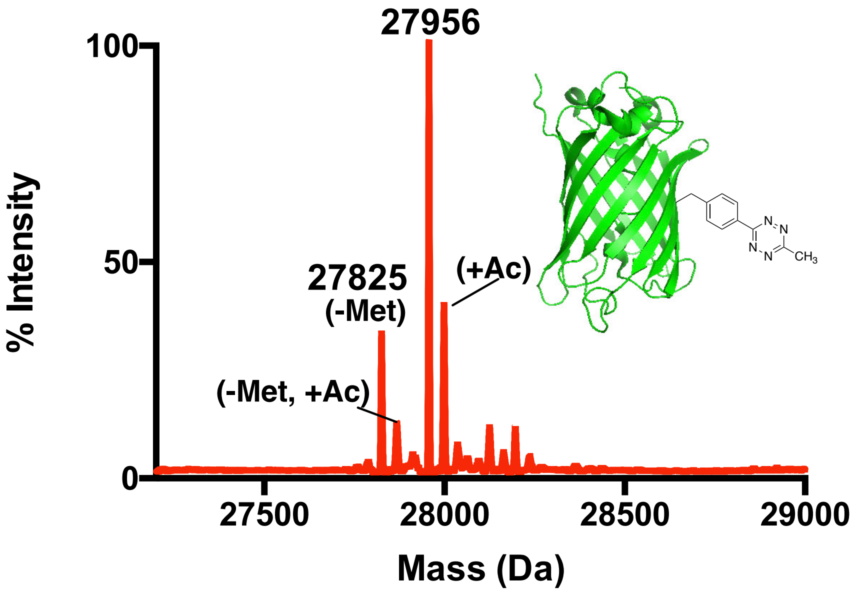

Fig. S15. Positive mode UPLC-ESI-MS deconvoluted mass spectrum of sfGFP-TetV2.0 used for *in vivo* experiments, with a major peak at 27956 $\pm$ 1 Da and a minor peak at 27824 $\pm$ 1 Da (loss

of methionine). Also observed, minor N-terminal acetylation (42 $\pm$ 1 Da), most likely due prolonged time to perform *in vivo* experiments prior to cell lysis and IMAC purification.

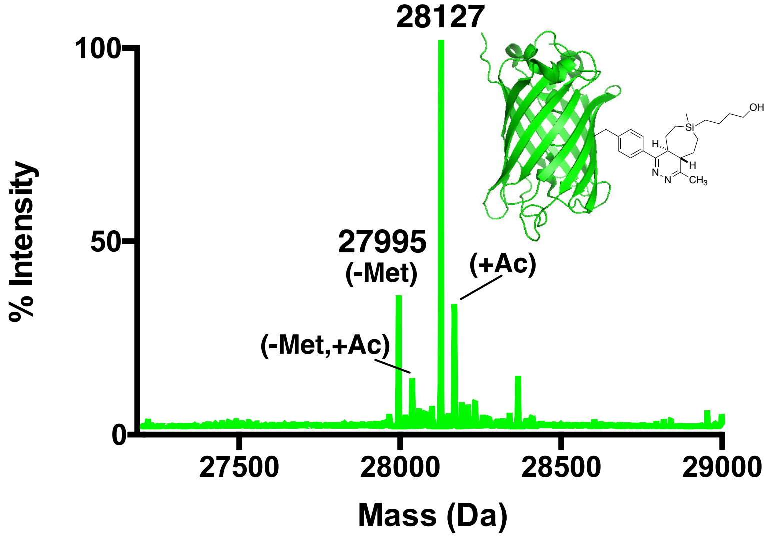

Fig.S16. Positive mode UPLC-ESI-MS deconvoluted mass spectrum of sfGFP-TetV2.0-SiTCH adduct from *in vivo* conjugation, with a major peak at 28127 $\pm$ 1 Da and a minor peak at 27824 $\pm$ 1 Da (loss of methionine). Also observed, minor N-terminal acetylation 42 $\pm$ 1 Da, most likely due prolonged time to perform *in vivo* experiments prior to cell lysis and IMAC purification. This shows an expected molecular weight difference of 170 Da at both major and minor peaks, indicating a quantitative and successful *in vivo* conjugation with Si-TCH. Analysis was performed on the Xevo GS-2 QTof (Waters Corp.) system and MaxEnt was used for deconvolution of spectra.

Pseudo-First Order Kinetics for *in vivo* conjugation of Si-TCH to sfGFP-TetV2.0

A 50 mL culture of E. coli overexpressing GFP-TetV2.0 was resuspended in PBS buffer and washed three times. Three pseudo-first order kinetic trials containing 100 μL of cell solution added to 2.85 mL PBS were initiated by adding 50 μL of stock Si-TCH (**7c**) methanol solutions (60, 50, 40 μM). Fluorescence increase was monitored, indicating conjugation of Si-TCH (**7c**) to GFP-TetV2.0 (Excitation 488 nm, Emission 506 nm, and 2s increments). For each trial, fluorescence was measured until a constant emission intensity was observed. Unimolecular rate constants were calculated for each concentration using Prism software (0.112, 0.079, 0.059 s^{-1} respectively) (**Fig. S17)** and a bimolecular constant was calculated by plotting each unimolecular rate constants against Si-TCH concentration (155,000 $\pm$ 20,000 $M^{-1}s^{-1}$) (**Fig. S18**)

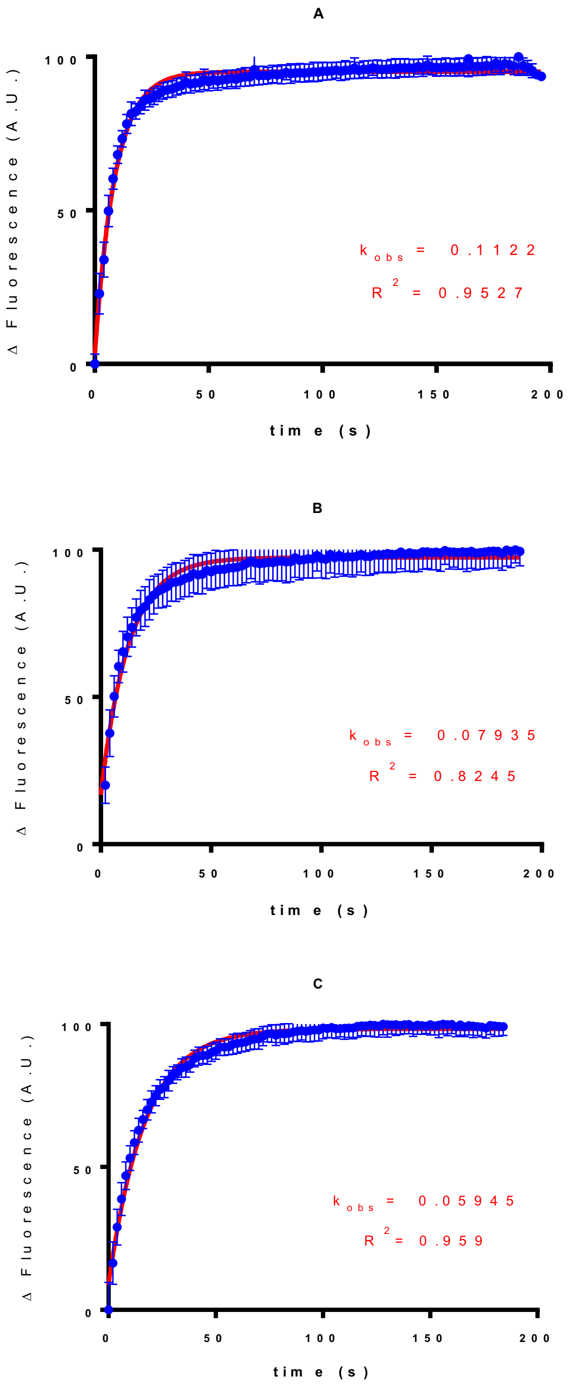

Fig. S17. Fluorescence kinetics of the *in vivo* reaction between Si-TCH and GFP-TetV2.0. Fluorescence was directly monitored from suspended bacterial cells in PBS. After injection, the final Si-TCH concentrations are as follows; **A**=1.0 μM, **B**=0.883 μM, **C**=0.666 μM. Raw data points (blue) and the fit curve (red) for a pseudo first order rate equation calculated on Prism software are shown.

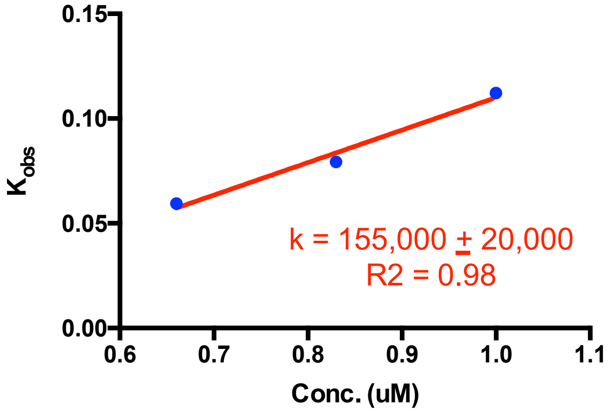

Fig. S18. *in vivo* bimolecular rate determination of three Si-TCH (**7c**) PBS/Methanol solutions reacted with GFP-TetV2.0 by plotting k_{obs} vs final Si-TCH (**7c**) concentration

***In Vivo* Mass Spec Analysis**

A series of reactions containing 50 μL of previously expressed GFP-TetV2.0 cell solution were added to 1.45 mL PBS. Reactions were initiated by adding Si-TCH•$AgNO_3$ complex **2c** to a final concentration of 1 μM and allowed to react for 10 minutes. After completion, cell solutions were pelleted, combined, washed three times, and resuspended in lysis buffer (50 mM Tris, NaCl 150 mM, sodium deoxycholate 0.5%, Triton X-100 1%, pH 7.5) and rocked for 30 minutes at room temperature. The lysed solution was spun down, the supernatant was purified by nickel affinity chromatography. Eluted fractions were applied to a 10 mL Amicon centrifugal filter (10,000 MWCO, EMD Millipore) and underwent three rounds of buffer exchange with PBS. The cell lysis solution was analyzed by LC-ESI-MS (**Fig. S15, S16**).

IR and NMR comparisons of Si-TCH and Si-TCH silver complexes

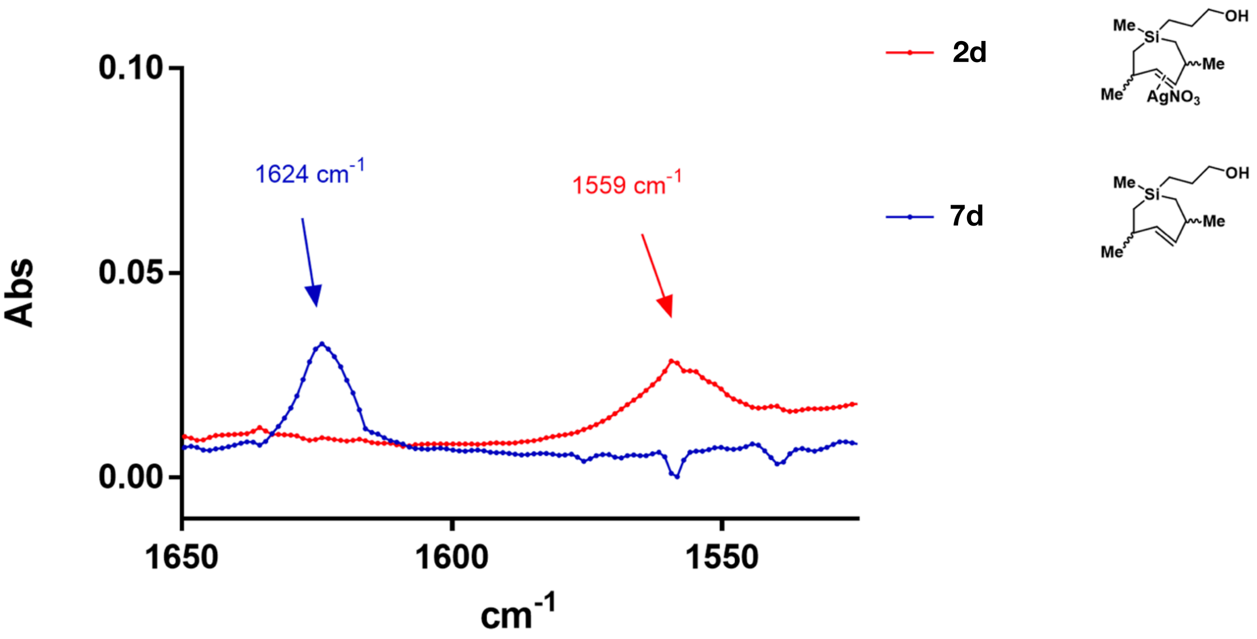

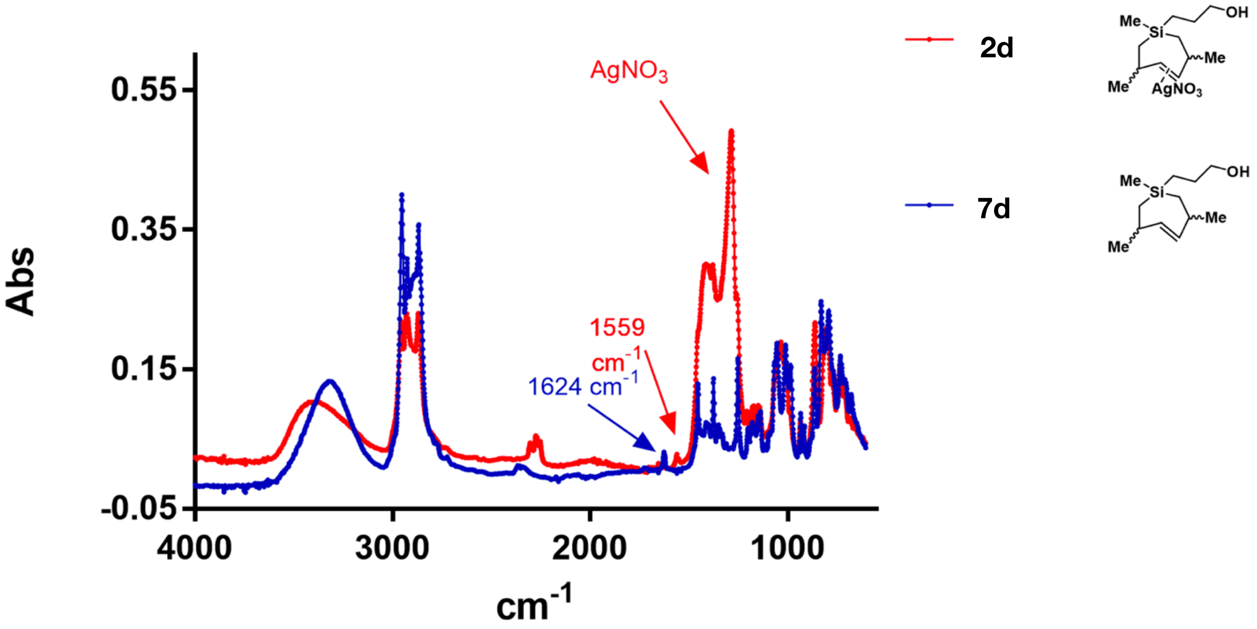

Fig.S19. Comparison of FT-IR (ATR) for the C=C stretch of compound **2d** and **7d**

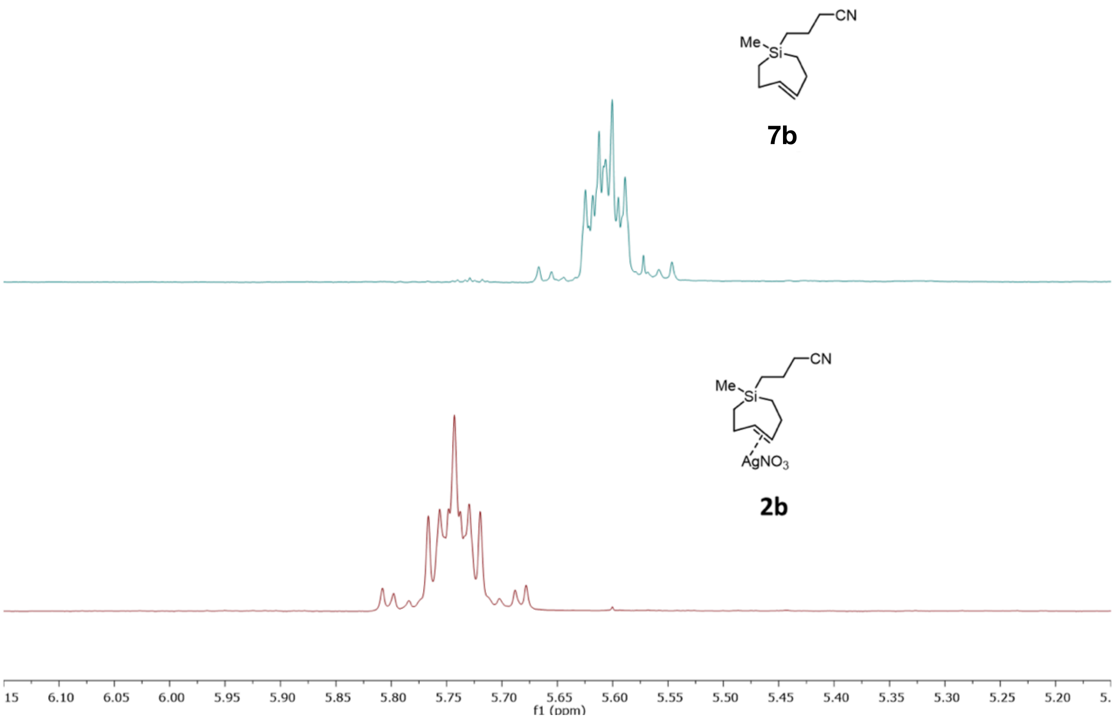

Fig.S20. Comparison of alkene resonances in ^{1}H NMR spectra (acetone-d_6, 400 MHz) of compound **2b** and **7b**.

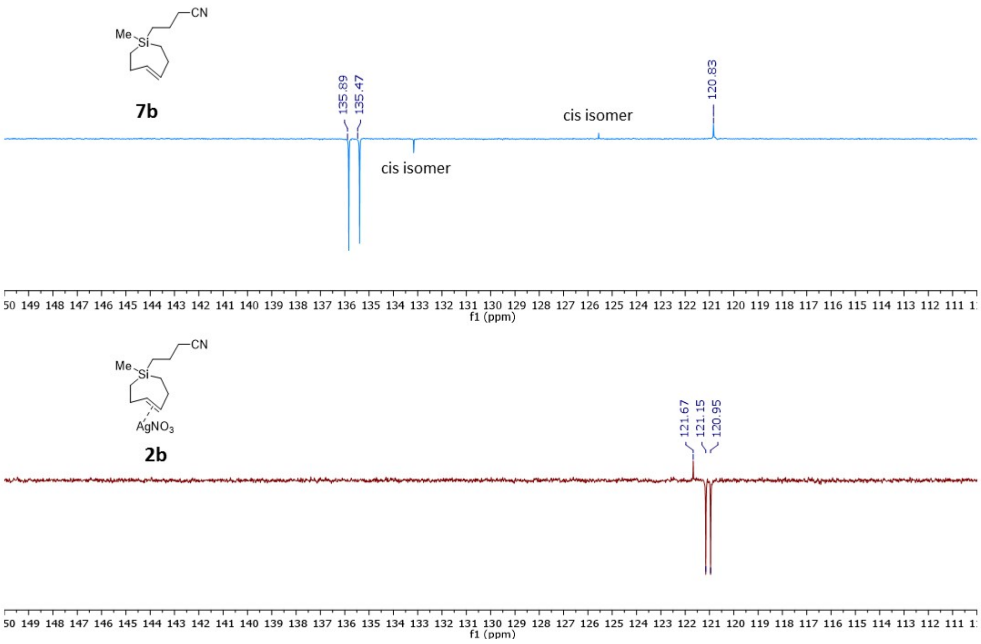

Fig.S21. Comparison of alkene resonances in ^{13}C NMR spectra (acetone-d_6, 100 MHz) of compound **2b** and **7b**.

X-ray data

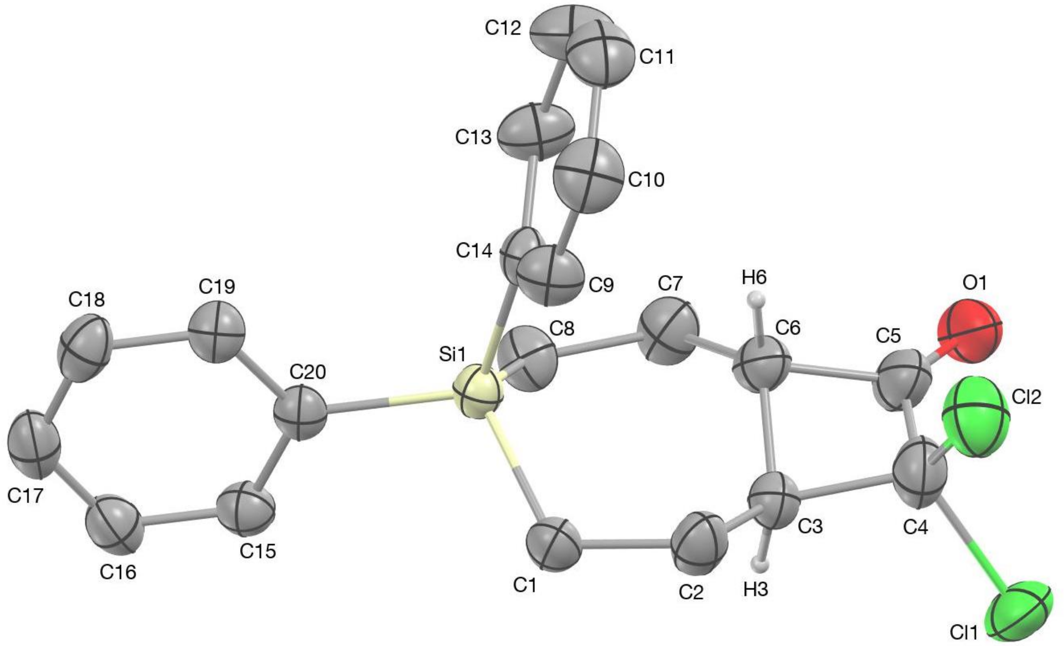

Fig.S22. X-ray structure of (*rel*-1R,7R)-9,9-dichloro-4,4-diphenyl-4-silabicyclo[5.2.0]nonan-8-one (**13**) Molecular diagram and crystallographic labeling scheme for compound **13** with ellipsoids at 50% probability. Minor disordered component for **13** and H-atoms other than the alkenyl H-atoms, depicted with arbitary radius, are omitted for clarity

X-ray structural analysis for compounds **13** (joef116) and **2a** (joef124): Crystals were mounted using viscous oil onto a plastic mesh and cooled to the data collection temperature. Data were collected on a Bruker-AXS APEX II DUO CCD diffractometer with Mo-Kα radiation (λ = 0.71073 Å) monochromated with graphite. Unit cell parameters were obtained from 36 data frames, 0.5^o ω, from three different sections of the Ewald sphere. The systematic absences in the diffraction data are uniquely consistent with $P2_1/c$. The data-sets were treated with multi-scan absorption corrections (Apex3 software suite, Madison, WI, 2005). The structures were solved using direct methods and refined with full-matrix, least-squares procedures on F^2 (Sheldrick, G.M. 2008. Acta Cryst. A64, 112-122). The *trans*-alkenyl ring in **2a** was disordered in two conformations, predominantly in the chair-like conformation, with refined site occupancy of 67/33. All non-hydrogen atoms were refined with anisotropic displacement parameters. All hydrogen atoms were treated as idealized contributions with geometrically calculated positions and with U_{iso} equal to 1.2U_{eq} of the attached atom. Atomic scattering factors are contained in various versions of the SHELXTL program library (Sheldrick, G., *op. cit.*).

Table 1. Sample and crystal data for 13

**Identification code**	joef116
**Chemical formula**	$C_{20}H_{20}Cl_2OSi$
**Formula weight**	375.35
**Temperature**	200(2) K

**Wavelength**	0.71073 Å	
**Crystal size**	0.148 x 0.502 x 0.606 mm	
**Crystal system**	monoclinic	
**Space group**	P 1 21/c 1	
**Unit cell dimensions**	a = 12.1989(17) Å	α = 90°
	b = 7.0792(10) Å	β = 94.322(2)°
	c = 21.834(3) Å	γ = 90°
**Volume**	1880.2(5) $Å^3$	
**Z**	4	
**Density (calculated)**	1.326 g/cm^3	
**Absorption coefficient**	0.413 mm^{-1}	
**F(000)**	784	

Table 2. Data collection and structure refinement for 13

**Theta range for data collection**	1.67 to 27.36°	
**Index ranges**	-15<=h<=15, -9<=k<=9, -28<=l<=28	
**Reflections collected**	21484	
**Independent reflections**	4243 [R(int) = 0.0480]	
**Coverage of independent reflections**	99.5%	
**Absorption correction**	multi-scan	
**Max. and min. transmission**	0.7456 and 0.6407	
**Structure solution technique**	direct methods	
**Structure solution program**	SHELXS-97 (Sheldrick 2008)	
**Refinement method**	Full-matrix least-squares on F^2	
**Refinement program**	SHELXL-2014/7 (Sheldrick, 2014)	
**Function minimized**	Σ w(F_o^2 - F_c^2)^2	
**Data / restraints / parameters**	4243 / 12 / 230	
**Goodness-of-fit on F^2**	1.013	
**Final R indices**	3168 data; I>2σ(I)	R1 = 0.0534, wR2 = 0.1253
	all data	R1 = 0.0765, wR2 = 0.1408
**Weighting scheme**	$w=1/[\sigma^2(F_o^2)+(0.0532P)^2+1.9253P]$ where $P=(F_o^2+2F_c^2)/3$	

**Largest diff. peak and hole**	0.370 and -0.478 eÅ^{-3}
**R.M.S. deviation from mean**	0.056 eÅ^{-3}

Table 3. Atomic coordinates and equivalent isotropic atomic displacement parameters (Å^2) for 13

U(eq) is defined as one third of the trace of the orthogonalized U_{ij} tensor.

	x/a	y/b	z/c	U(eq)
Si1	0.21036(5)	0.50672(9)	0.60939(3)	0.03091(17)
Cl1	0.27173(16)	0.7157(2)	0.34065(7)	0.0607(4)
Cl2	0.43713(11)	0.8294(3)	0.43653(7)	0.0625(4)
Cl1'	0.2944(4)	0.6736(6)	0.35993(14)	0.0607(4)
Cl2'	0.4240(3)	0.9343(5)	0.43813(15)	0.0625(4)
O1	0.1776(2)	0.0584(3)	0.42359(9)	0.0617(6)
C1	0.2613(2)	0.3662(4)	0.54416(11)	0.0403(6)
C2	0.3175(2)	0.4757(4)	0.49465(12)	0.0472(7)
C2'	0.3175(2)	0.4757(4)	0.49465(12)	0.0472(7)
C3	0.2494(3)	0.6319(6)	0.46624(18)	0.0363(8)
C3'	0.2824(7)	0.6780(12)	0.4881(4)	0.0363(8)
C4	0.2980(2)	0.7790(4)	0.42283(12)	0.0507(7)
C5	0.2152(3)	0.9159(4)	0.44294(12)	0.0547(8)
C6	0.2132(4)	0.8048(5)	0.50336(17)	0.0412(8)
C6'	0.1604(7)	0.7471(11)	0.4801(3)	0.0412(8)
C7	0.1066(2)	0.8110(4)	0.53415(13)	0.0473(7)
C7'	0.1066(2)	0.8110(4)	0.53415(13)	0.0473(7)
C8	0.0891(2)	0.6540(4)	0.58029(12)	0.0404(6)
C9	0.4331(2)	0.6043(4)	0.64592(12)	0.0421(6)
C10	0.5162(2)	0.7200(5)	0.67064(13)	0.0502(7)
C11	0.4925(2)	0.8904(4)	0.69590(13)	0.0510(7)
C12	0.3851(2)	0.9451(5)	0.69664(14)	0.0561(8)
C13	0.3018(2)	0.8315(4)	0.67167(13)	0.0469(7)
C14	0.32289(18)	0.6574(3)	0.64548(10)	0.0308(5)
C15	0.0728(2)	0.2146(3)	0.64947(11)	0.0371(5)
C16	0.0289(2)	0.0943(4)	0.69111(13)	0.0430(6)
C17	0.0697(2)	0.0960(4)	0.75171(13)	0.0442(6)
C18	0.1529(2)	0.2195(4)	0.77044(12)	0.0421(6)

	x/a	y/b	z/c	U(eq)
C19	0.19615(19)	0.3410(4)	0.72866(11)	0.0366(5)
C20	0.15723(18)	0.3409(3)	0.66688(10)	0.0316(5)

Table 4. Bond lengths (Å) for 13

Si1-C14	1.865(2)	Si1-C20	1.870(2)
Si1-C8	1.881(2)	Si1-C1	1.881(2)
Cl1-C4	1.854(3)	Cl2-C4	1.738(3)
Cl1'-C4	1.561(4)	Cl2'-C4	1.899(4)
O1-C5	1.174(3)	C1-C2'	1.533(4)
C1-C2	1.533(4)	C1-H1A	0.99
C1-H1B	0.99	C2-C3	1.490(5)
C2-H2A	0.99	C2-H2B	0.99
C2'-C3'	1.499(8)	C2'-H2'1	0.99
C2'-H2'2	0.99	C3-C6	1.551(5)
C3-C4	1.555(5)	C3-H3	1.0
C3'-C6'	1.564(10)	C3'-C4	1.618(8)
C3'-H3'	1.0	C4-C5	1.490(4)
C5-C6	1.538(4)	C5-C6'	1.617(8)
C6-C7	1.509(5)	C6-H6	1.0
C6'-C7'	1.464(8)	C6'-H6'	1.0
C7-C8	1.525(4)	C7-H7A	0.99
C7-H7B	0.99	C7'-C8	1.525(4)
C7'-H7'1	0.99	C7'-H7'2	0.99
C8-H8A	0.99	C8-H8B	0.99
C9-C10	1.382(4)	C9-C14	1.395(3)
C9-H9	0.95	C10-C11	1.367(4)
C10-H10	0.95	C11-C12	1.368(4)
C11-H11	0.95	C12-C13	1.375(4)
C12-H12	0.95	C13-C14	1.391(4)
C13-H13	0.95	C15-C16	1.383(4)
C15-C20	1.395(3)	C15-H15	0.95
C16-C17	1.378(4)	C16-H16	0.95
C17-C18	1.378(4)	C17-H17	0.95
C18-C19	1.386(3)	C18-H18	0.95
C19-C20	1.396(3)	C19-H19	0.95

Table 5. Bond angles (°) for 13

C14-Si1-C20	110.89(10)	C14-Si1-C8	111.14(11)

C20-Si1-C8	105.74(11)	C14-Si1-C1	110.32(11)
C20-Si1-C1	109.12(11)	C8-Si1-C1	109.50(12)
C2'-C1-Si1	117.21(18)	C2-C1-Si1	117.21(18)
C2-C1-H1A	108.0	Si1-C1-H1A	108.0
C2-C1-H1B	108.0	Si1-C1-H1B	108.0
H1A-C1-H1B	107.2	C3-C2-C1	113.8(2)
C3-C2-H2A	108.8	C1-C2-H2A	108.8
C3-C2-H2B	108.8	C1-C2-H2B	108.8
H2A-C2-H2B	107.7	C3'-C2'-C1	114.3(4)
C3'-C2'-H2'1	108.7	C1-C2'-H2'1	108.7
C3'-C2'-H2'2	108.7	C1-C2'-H2'2	108.7
H2'1-C2'-H2'2	107.6	C2-C3-C6	122.7(3)
C2-C3-C4	121.6(3)	C6-C3-C4	85.8(3)
C2-C3-H3	108.2	C6-C3-H3	108.2
C4-C3-H3	108.2	C2'-C3'-C6'	124.9(7)
C2'-C3'-C4	117.1(6)	C6'-C3'-C4	86.5(5)
C2'-C3'-H3'	108.7	C6'-C3'-H3'	108.7
C4-C3'-H3'	108.7	C5-C4-C3	87.6(2)
C5-C4-Cl1'	126.6(3)	C5-C4-C3'	84.3(3)
Cl1'-C4-C3'	124.5(4)	C5-C4-Cl2	119.4(2)
C3-C4-Cl2	116.4(2)	C5-C4-Cl1	111.8(2)
C3-C4-Cl1	112.4(2)	Cl2-C4-Cl1	108.16(16)
C5-C4-Cl2'	97.5(2)	Cl1'-C4-Cl2'	113.6(3)
C3'-C4-Cl2'	104.4(4)	O1-C5-C4	135.1(3)
O1-C5-C6	135.9(3)	C4-C5-C6	88.6(2)
O1-C5-C6'	130.6(4)	C4-C5-C6'	89.0(3)
C7-C6-C5	115.9(3)	C7-C6-C3	123.0(3)
C5-C6-C3	86.1(3)	C7-C6-H6	109.9
C5-C6-H6	109.9	C3-C6-H6	109.9
C7'-C6'-C3'	119.3(6)	C7'-C6'-C5	113.8(5)
C3'-C6'-C5	82.0(5)	C7'-C6'-H6'	112.8
C3'-C6'-H6'	112.8	C5-C6'-H6'	112.8
C6-C7-C8	116.1(2)	C6-C7-H7A	108.3
C8-C7-H7A	108.3	C6-C7-H7B	108.3
C8-C7-H7B	108.3	H7A-C7-H7B	107.4
C6'-C7'-C8	113.6(4)	C6'-C7'-H7'1	108.8
C8-C7'-H7'1	108.8	C6'-C7'-H7'2	108.8
C8-C7'-H7'2	108.8	H7'1-C7'-H7'2	107.7

C7'-C8-Si1	118.80(18)	C7-C8-Si1	118.80(18)
C7-C8-H8A	107.6	Si1-C8-H8A	107.6
C7-C8-H8B	107.6	Si1-C8-H8B	107.6
H8A-C8-H8B	107.0	C10-C9-C14	121.4(2)
C10-C9-H9	119.3	C14-C9-H9	119.3
C11-C10-C9	120.7(2)	C11-C10-H10	119.7
C9-C10-H10	119.7	C10-C11-C12	119.1(3)
C10-C11-H11	120.4	C12-C11-H11	120.4
C11-C12-C13	120.6(3)	C11-C12-H12	119.7
C13-C12-H12	119.7	C12-C13-C14	121.8(2)
C12-C13-H13	119.1	C14-C13-H13	119.1
C13-C14-C9	116.3(2)	C13-C14-Si1	121.83(18)
C9-C14-Si1	121.80(19)	C16-C15-C20	122.0(2)
C16-C15-H15	119.0	C20-C15-H15	119.0
C17-C16-C15	119.7(3)	C17-C16-H16	120.1
C15-C16-H16	120.1	C16-C17-C18	119.7(2)
C16-C17-H17	120.2	C18-C17-H17	120.2
C17-C18-C19	120.4(2)	C17-C18-H18	119.8
C19-C18-H18	119.8	C18-C19-C20	121.1(2)
C18-C19-H19	119.4	C20-C19-H19	119.4
C15-C20-C19	117.0(2)	C15-C20-Si1	120.37(18)
C19-C20-Si1	122.59(18)		

Table 6. Torsion angles (°) for 13

C14-Si1-C1-C2'	-52.8(2)	C20-Si1-C1-C2'	-174.9(2)
C8-Si1-C1-C2'	69.8(2)	C14-Si1-C1-C2	-52.8(2)
C20-Si1-C1-C2	-174.9(2)	C8-Si1-C1-C2	69.8(2)
Si1-C1-C2-C3	-54.9(3)	Si1-C1-C2'-C3'	-25.9(5)
C1-C2-C3-C6	63.1(4)	C1-C2-C3-C4	170.2(3)
C1-C2'-C3'-C6'	-50.9(8)	C1-C2'-C3'-C4	-156.7(4)
C2-C3-C4-C5	-152.1(3)	C6-C3-C4-C5	-25.8(3)
C2-C3-C4-Cl2	-30.2(4)	C6-C3-C4-Cl2	96.0(3)
C2-C3-C4-Cl1	95.4(4)	C6-C3-C4-Cl1	-138.4(3)
C2'-C3'-C4-C5	160.2(6)	C6'-C3'-C4-C5	32.5(5)
C2'-C3'-C4-Cl1'	29.1(8)	C6'-C3'-C4-Cl1'	-98.6(5)
C2'-C3'-C4-Cl2'	-103.5(6)	C6'-C3'-C4-Cl2'	128.7(4)

C3-C4-C5-O1	-161.4(4)	Cl1'-C4-C5-O1	-56.7(6)
C3'-C4-C5-O1	174.0(5)	Cl2-C4-C5-O1	79.5(5)
Cl1-C4-C5-O1	-48.2(5)	Cl2'-C4-C5-O1	70.2(4)
C3-C4-C5-C6	26.0(3)	Cl2-C4-C5-C6	-93.2(3)
Cl1-C4-C5-C6	139.2(2)	Cl1'-C4-C5-C6'	98.1(4)
C3'-C4-C5-C6'	-31.2(5)	Cl2'-C4-C5-C6'	-135.0(4)
O1-C5-C6-C7	36.3(6)	C4-C5-C6-C7	-151.2(3)
O1-C5-C6-C3	161.4(5)	C4-C5-C6-C3	-26.1(3)
C2-C3-C6-C7	-91.1(5)	C4-C3-C6-C7	143.6(3)
C2-C3-C6-C5	150.3(4)	C4-C3-C6-C5	25.0(3)
C2'-C3'-C6'-C7'	96.5(9)	C4-C3'-C6'-C7'	-142.6(6)
C2'-C3'-C6'-C5	-150.7(7)	C4-C3'-C6'-C5	-29.8(4)
O1-C5-C6'-C7'	-52.2(8)	C4-C5-C6'-C7'	151.1(5)
O1-C5-C6'-C3'	-170.7(5)	C4-C5-C6'-C3'	32.6(4)
C5-C6-C7-C8	160.0(3)	C3-C6-C7-C8	56.9(4)
C3'-C6'-C7'-C8	-66.6(8)	C5-C6'-C7'-C8	-160.8(4)
C6'-C7'-C8-Si1	53.2(5)	C6-C7-C8-Si1	14.9(4)
C14-Si1-C8-C7'	56.9(2)	C20-Si1-C8-C7'	177.3(2)
C1-Si1-C8-C7'	-65.2(2)	C14-Si1-C8-C7	56.9(2)
C20-Si1-C8-C7	177.3(2)	C1-Si1-C8-C7	-65.2(2)
C14-C9-C10-C11	0.4(4)	C9-C10-C11-C12	0.2(5)
C10-C11-C12-C13	-0.8(5)	C11-C12-C13-C14	0.7(5)
C12-C13-C14-C9	0.0(4)	C12-C13-C14-Si1	-178.0(2)
C10-C9-C14-C13	-0.5(4)	C10-C9-C14-Si1	177.5(2)
C20-Si1-C14-C13	-90.6(2)	C8-Si1-C14-C13	26.7(2)
C1-Si1-C14-C13	148.4(2)	C20-Si1-C14-C9	91.5(2)
C8-Si1-C14-C9	-151.2(2)	C1-Si1-C14-C9	-29.5(2)
C20-C15-C16-C17	-0.7(4)	C15-C16-C17-C18	0.8(4)
C16-C17-C18-C19	-0.3(4)	C17-C18-C19-C20	-0.3(4)
C16-C15-C20-C19	0.1(3)	C16-C15-C20-Si1	-177.83(19)

C18-C19-C20-C15	0.5(3)	C18-C19-C20-Si1	178.29(18)
C14-Si1-C20-C15	-179.75(18)	C8-Si1-C20-C15	59.7(2)
C1-Si1-C20-C15	-58.0(2)	C14-Si1-C20-C19	2.5(2)
C8-Si1-C20-C19	-118.1(2)	C1-Si1-C20-C19	124.2(2)

Table 7. Anisotropic atomic displacement parameters (Å^2) for 13.

The anisotropic atomic displacement factor exponent takes the form: $-2\pi^2[h^2 a^{*2} U_{11} + ... + 2 h k a^* b^* U_{12}]$

	U_{11}	U_{22}	U_{33}	U_{23}	U_{13}	U_{12}
Si1	0.0309(3)	0.0312(3)	0.0306(3)	0.0027(3)	0.0020(2)	0.0027(3)
Cl1	0.0935(11)	0.0581(9)	0.0300(8)	-0.0060(6)	0.0021(7)	0.0073(7)
Cl2	0.0538(6)	0.0699(10)	0.0641(6)	0.0096(8)	0.0063(4)	-0.0087(8)
Cl1'	0.0935(11)	0.0581(9)	0.0300(8)	-0.0060(6)	0.0021(7)	0.0073(7)
Cl2'	0.0538(6)	0.0699(10)	0.0641(6)	0.0096(8)	0.0063(4)	-0.0087(8)
O1	0.1082(18)	0.0360(10)	0.0393(10)	0.0090(9)	-0.0043(11)	0.0128(11)
C1	0.0515(15)	0.0311(13)	0.0391(13)	0.0010(10)	0.0088(11)	0.0037(11)
C2	0.0585(17)	0.0432(15)	0.0420(14)	0.0038(12)	0.0173(12)	0.0111(13)
C2'	0.0585(17)	0.0432(15)	0.0420(14)	0.0038(12)	0.0173(12)	0.0111(13)
C3	0.041(2)	0.036(2)	0.032(2)	0.0031(16)	0.0033(16)	0.0003(16)
C3'	0.041(2)	0.036(2)	0.032(2)	0.0031(16)	0.0033(16)	0.0003(16)
C4	0.0587(17)	0.0511(17)	0.0435(15)	0.0123(13)	0.0110(12)	0.0010(14)
C5	0.089(2)	0.0428(16)	0.0329(14)	0.0046(12)	0.0069(14)	0.0088(16)
C6	0.057(2)	0.035(2)	0.0312(19)	0.0026(15)	-0.0020(15)	0.0082(17)
C6'	0.057(2)	0.035(2)	0.0312(19)	0.0026(15)	-0.0020(15)	0.0082(17)
C7	0.0524(16)	0.0450(15)	0.0445(14)	0.0081(12)	0.0036(12)	0.0194(13)

C7'	0.0524(16)	0.0450(15)	0.0445(14)	0.0081(12)	0.0036(12)	0.0194(13)
C8	0.0337(12)	0.0418(14)	0.0447(14)	0.0066(11)	-0.0036(10)	0.0045(11)
C9	0.0352(13)	0.0402(14)	0.0507(15)	-0.0011(12)	0.0010(11)	0.0105(11)
C10	0.0298(13)	0.0635(19)	0.0565(17)	0.0042(15)	-0.0018(11)	0.0046(12)
C11	0.0423(15)	0.0574(18)	0.0518(16)	0.0005(14)	-0.0062(12)	-0.0090(13)
C12	0.0536(17)	0.0502(17)	0.0627(19)	-0.0208(15)	-0.0076(14)	0.0049(14)
C13	0.0350(13)	0.0525(16)	0.0528(16)	-0.0138(13)	0.0002(11)	0.0093(12)
C14	0.0295(11)	0.0351(12)	0.0277(11)	0.0066(9)	0.0021(8)	0.0039(9)
C15	0.0379(13)	0.0346(13)	0.0386(13)	-0.0011(11)	0.0020(10)	0.0017(10)
C16	0.0415(14)	0.0344(13)	0.0542(16)	0.0022(12)	0.0115(12)	-0.0021(11)
C17	0.0455(14)	0.0376(14)	0.0518(16)	0.0117(12)	0.0183(12)	0.0072(12)
C18	0.0440(14)	0.0487(15)	0.0340(12)	0.0083(11)	0.0051(10)	0.0138(12)
C19	0.0316(12)	0.0402(13)	0.0381(13)	0.0037(11)	0.0023(9)	0.0032(10)
C20	0.0294(11)	0.0308(12)	0.0351(12)	0.0029(10)	0.0051(9)	0.0069(9)

Table 8. Hydrogen atomic coordinates and isotropic atomic displacement parameters (Å^2) for 13.

	x/a	y/b	z/c	U(eq)
H1A	0.3139	0.2705	0.5618	0.048
H1B	0.1980	0.2971	0.5239	0.048
H2A	0.3874	0.5291	0.5132	0.057
H2B	0.3358	0.3865	0.4620	0.057
H2'1	0.3980	0.4716	0.5045	0.057
H2'2	0.3017	0.4113	0.4547	0.057
H3	0.1824	0.5752	0.4443	0.044
H3'	0.3224	0.7536	0.5214	0.044
H6	0.2751	0.8468	0.5328	0.049

x/a	y/b	z/c	U(eq)	
H6'	0.1126	0.6627	0.4529	0.049
H7A	0.0451	0.8069	0.5018	0.057
H7B	0.1023	0.9338	0.5555	0.057
H7'1	0.0343	0.8665	0.5205	0.057
H7'2	0.1517	0.9117	0.5549	0.057
H8A	0.0326	0.5674	0.5613	0.048
H8B	0.0579	0.7123	0.6163	0.048
H9	0.4513	0.4860	0.6288	0.051
H10	0.5906	0.6807	0.6701	0.06
H11	0.5499	0.9699	0.7127	0.061
H12	0.3678	1.0626	0.7146	0.067
H13	0.2279	0.8732	0.6723	0.056
H15	0.0446	0.2111	0.6077	0.044
H16	-0.0291	0.0107	0.6780	0.052
H17	0.0406	0.0125	0.7804	0.053
H18	0.1809	0.2214	0.8122	0.051
H19	0.2532	0.4258	0.7423	0.044

X-ray structure of (*E*)-1,1-diphenyl-2,3,6,7-tetrahydro-1H-silepine •$AgNO_3$ (2a)

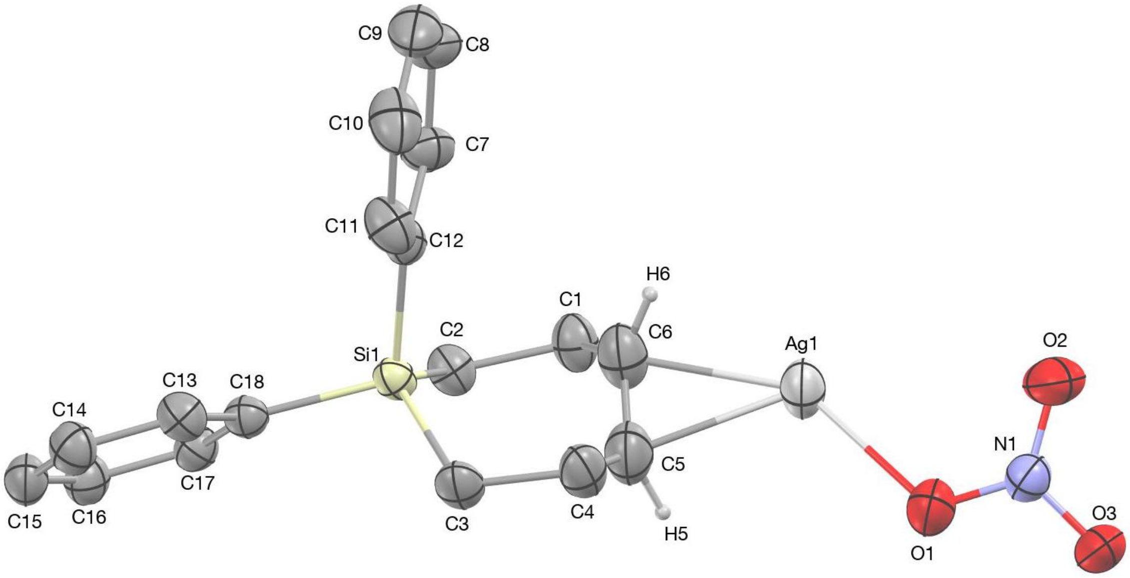

Fig.S23. X-ray structure of (*E*)-1,1-diphenyl-2,3,6,7-tetrahydro-1H-silepine (**2a**)

Table 9. Crystal data and structure refinement for 2a.

**Identification code**	joef124
**Empirical formula**	C18 H20 Ag N O3 Si
**Formula weight**	434.31
**Temperature**	200(2) K
**Wavelength**	0.71073 A
**Crystal system, space group**	Monoclinic, P2(1)/c
**Unit cell dimensions**	a = 16.5090(10) A alpha = 90 deg.<br>b = 8.4282(5) A beta = 114.6900(10) deg.<br>c = 13.9655(8) A gamma = 90 deg.
**Volume**	1765.53(18) A^3
**Z, Calculated density**	4, 1.634 Mg/m^3
**Absorption coefficient**	1.225 mm^-1

F(000) 880

Crystal size 0.394 x 0.345 x 0.194 mm

Theta range for data collection 2.716 to 27.574 deg.

Limiting indices -21<=h<=21, -10<=k<=10, -18<=l<=18

Reflections collected / unique 18866 / 4077 [R(int) = 0.0148]

Completeness to theta = 25.242 99.9 %

Absorption correction Semi-empirical from equivalents

Max. and min. transmission 0.7456 and 0.6940

Refinement method Full-matrix least-squares on F^2

Data / restraints / parameters 4077 / 0 / 217

Goodness-of-fit on F^2 1.009

Final R indices [I>2sigma(I)] R1 = 0.0369, wR2 = 0.1133

R indices (all data) R1 = 0.0395, wR2 = 0.1174

Extinction coefficient n/a

Largest diff. peak and hole 1.796 and -0.630 e.A^-3

Table 10. Atomic coordinates (x 10^4) and equivalent isotropic displacement parameters (A^2 x 10^3) for 2a.
U(eq) is defined as one third of the trace of the orthogonalized Uij tensor.

	x	y	z	U(eq)
Ag(1)	3989(1)	5314(1)	2962(1)	47(1)
C(1)	3696(2)	1563(3)	3408(2)	42(1)
C(2)	3345(2)	599(3)	4111(2)	36(1)
C(3)	2681(2)	3532(3)	5097(2)	40(1)
C(4)	2908(2)	4901(3)	4509(2)	45(1)
C(5)	3582(3)	4145(4)	4184(3)	56(1)
C(6)	3330(2)	3202(4)	3346(3)	53(1)
C(7)	1342(2)	931(3)	2219(2)	41(1)
C(8)	588(2)	1007(4)	1272(2)	54(1)
C(9)	-144(2)	1895(5)	1204(3)	55(1)
C(10)	-108(2)	2718(4)	2077(3)	54(1)
C(11)	654(2)	2641(4)	3031(2)	44(1)
C(12)	1391(2)	1725(3)	3125(2)	33(1)
C(13)	1419(2)	470(3)	5522(2)	37(1)
C(14)	1214(2)	-476(3)	6211(2)	41(1)
C(15)	1739(2)	-1773(3)	6673(2)	41(1)
C(16)	2465(2)	-2145(3)	6450(2)	38(1)
C(17)	2668(2)	-1189(3)	5766(2)	33(1)
C(18)	2148(2)	154(3)	5289(2)	31(1)
N(1)	4912(2)	8436(3)	2913(2)	38(1)
O(1)	4712(2)	7654(3)	3540(2)	57(1)
O(2)	4507(2)	8296(4)	1953(2)	67(1)
O(3)	5525(2)	9425(4)	3280(2)	64(1)
Si(1)	2420(1)	1528(1)	4396(1)	29(1)

Table 11. Bond lengths [A] and angles [deg] for 2a.

Ag(1)-C(6)	2.264(3)
Ag(1)-O(1)	2.271(2)
Ag(1)-C(5)	2.300(3)
Ag(1)-O(3)#1	2.318(3)
C(1)-C(6)	1.496(4)
C(1)-C(2)	1.561(4)
C(1)-H(1A)	0.9900
C(1)-H(1B)	0.9900
C(2)-Si(1)	1.901(3)
C(2)-H(2A)	0.9900
C(2)-H(2B)	0.9900
C(3)-C(4)	1.550(4)
C(3)-Si(1)	1.909(3)
C(3)-H(3A)	0.9900
C(3)-H(3B)	0.9900
C(4)-C(5)	1.508(5)
C(4)-H(4A)	0.9900
C(4)-H(4B)	0.9900
C(5)-C(6)	1.329(5)
C(5)-H(5)	0.9500
C(6)-H(6)	0.9500
C(7)-C(8)	1.388(4)
C(7)-C(12)	1.404(3)
C(7)-H(7)	0.9500
C(8)-C(9)	1.391(5)
C(8)-H(8)	0.9500
C(9)-C(10)	1.381(5)
C(9)-H(9)	0.9500
C(10)-C(11)	1.400(4)
C(10)-H(10)	0.9500
C(11)-C(12)	1.399(4)
C(11)-H(11)	0.9500
C(12)-Si(1)	1.882(2)
C(13)-C(14)	1.396(4)
C(13)-C(18)	1.396(4)
C(13)-H(13)	0.9500
C(14)-C(15)	1.377(4)
C(14)-H(14)	0.9500
C(15)-C(16)	1.394(4)
C(15)-H(15)	0.9500
C(16)-C(17)	1.394(4)
C(16)-H(16)	0.9500

C(17)-C(18)	1.408(4)
C(17)-H(17)	0.9500
C(18)-Si(1)	1.889(3)
N(1)-O(2)	1.229(3)
N(1)-O(1)	1.246(3)
N(1)-O(3)	1.244(3)
O(3)-Ag(1)#2	2.318(3)
C(6)-Ag(1)-O(1)	146.23(10)
C(6)-Ag(1)-C(5)	33.84(12)
O(1)-Ag(1)-C(5)	112.40(11)
C(6)-Ag(1)-O(3)#1	105.86(12)
O(1)-Ag(1)-O(3)#1	104.61(11)
C(5)-Ag(1)-O(3)#1	135.69(14)
C(6)-C(1)-C(2)	105.7(2)
C(6)-C(1)-H(1A)	110.6
C(2)-C(1)-H(1A)	110.6
C(6)-C(1)-H(1B)	110.6
C(2)-C(1)-H(1B)	110.6
H(1A)-C(1)-H(1B)	108.7
C(1)-C(2)-Si(1)	117.36(18)
C(1)-C(2)-H(2A)	108.0
Si(1)-C(2)-H(2A)	108.0
C(1)-C(2)-H(2B)	108.0
Si(1)-C(2)-H(2B)	108.0
H(2A)-C(2)-H(2B)	107.2
C(4)-C(3)-Si(1)	116.11(17)
C(4)-C(3)-H(3A)	108.3
Si(1)-C(3)-H(3A)	108.3
C(4)-C(3)-H(3B)	108.3
Si(1)-C(3)-H(3B)	108.3
H(3A)-C(3)-H(3B)	107.4
C(5)-C(4)-C(3)	102.5(2)
C(5)-C(4)-H(4A)	111.3
C(3)-C(4)-H(4A)	111.3
C(5)-C(4)-H(4B)	111.3
C(3)-C(4)-H(4B)	111.3
H(4A)-C(4)-H(4B)	109.2
C(6)-C(5)-C(4)	121.2(3)
C(6)-C(5)-Ag(1)	71.60(19)
C(4)-C(5)-Ag(1)	121.6(2)
C(6)-C(5)-H(5)	119.4
C(4)-C(5)-H(5)	119.4
Ag(1)-C(5)-H(5)	77.8
C(5)-C(6)-C(1)	122.0(3)
C(5)-C(6)-Ag(1)	74.6(2)

C(1)-C(6)-Ag(1)	121.1(2)
C(5)-C(6)-H(6)	119.0
C(1)-C(6)-H(6)	119.0
Ag(1)-C(6)-H(6)	75.0
C(8)-C(7)-C(12)	121.9(3)
C(8)-C(7)-H(7)	119.1
C(12)-C(7)-H(7)	119.1
C(9)-C(8)-C(7)	119.8(3)
C(9)-C(8)-H(8)	120.1
C(7)-C(8)-H(8)	120.1
C(10)-C(9)-C(8)	119.6(3)
C(10)-C(9)-H(9)	120.2
C(8)-C(9)-H(9)	120.2
C(9)-C(10)-C(11)	120.4(3)
C(9)-C(10)-H(10)	119.8
C(11)-C(10)-H(10)	119.8
C(12)-C(11)-C(10)	121.0(3)
C(12)-C(11)-H(11)	119.5
C(10)-C(11)-H(11)	119.5
C(11)-C(12)-C(7)	117.2(2)
C(11)-C(12)-Si(1)	123.0(2)
C(7)-C(12)-Si(1)	119.81(19)
C(14)-C(13)-C(18)	122.1(2)
C(14)-C(13)-H(13)	118.9
C(18)-C(13)-H(13)	118.9
C(15)-C(14)-C(13)	119.4(3)
C(15)-C(14)-H(14)	120.3
C(13)-C(14)-H(14)	120.3
C(14)-C(15)-C(16)	120.4(2)
C(14)-C(15)-H(15)	119.8
C(16)-C(15)-H(15)	119.8
C(17)-C(16)-C(15)	119.8(2)
C(17)-C(16)-H(16)	120.1
C(15)-C(16)-H(16)	120.1
C(16)-C(17)-C(18)	121.0(2)
C(16)-C(17)-H(17)	119.5
C(18)-C(17)-H(17)	119.5
C(13)-C(18)-C(17)	117.2(2)
C(13)-C(18)-Si(1)	120.13(19)
C(17)-C(18)-Si(1)	122.62(19)
O(2)-N(1)-O(1)	122.2(2)
O(2)-N(1)-O(3)	119.4(3)
O(1)-N(1)-O(3)	118.3(2)
N(1)-O(1)-Ag(1)	117.64(18)
N(1)-O(3)-Ag(1)#2	112.67(18)
C(12)-Si(1)-C(18)	107.90(11)

C(12)-Si(1)-C(2)	109.07(11)
C(18)-Si(1)-C(2)	108.62(11)
C(12)-Si(1)-C(3)	109.08(12)
C(18)-Si(1)-C(3)	105.61(11)
C(2)-Si(1)-C(3)	116.24(13)

Symmetry transformations used to generate equivalent atoms:
#1 -x+1,y-1/2,-z+1/2   #2 -x+1,y+1/2,-z+1/2

Table 12. Anisotropic displacement parameters (A^2 x 10^3) for 2a
The anisotropic displacement factor exponent takes the form:
-2 pi^2 [h^2 a*^2 U11 + ... + 2 h k a* b* U12]

	U11	U22	U33	U23	U13	U12
Ag(1)	60(1)	38(1)	54(1)	0(1)	35(1)	-8(1)
C(1)	49(1)	39(1)	48(1)	3(1)	30(1)	3(1)
C(2)	40(1)	34(1)	36(1)	3(1)	19(1)	6(1)
C(3)	56(2)	33(1)	35(1)	-7(1)	24(1)	-4(1)
C(4)	68(2)	30(1)	44(1)	-5(1)	30(1)	-6(1)
C(5)	70(2)	48(2)	59(2)	4(2)	36(2)	-10(2)
C(6)	72(2)	40(2)	62(2)	3(1)	43(2)	0(1)
C(7)	43(1)	38(1)	38(1)	-7(1)	12(1)	-3(1)
C(8)	55(2)	54(2)	39(1)	-6(1)	8(1)	-13(1)
C(9)	42(1)	63(2)	47(2)	15(1)	4(1)	-7(1)
C(10)	40(1)	57(2)	63(2)	24(2)	21(1)	12(1)
C(11)	47(1)	45(2)	45(1)	10(1)	23(1)	12(1)
C(12)	36(1)	29(1)	33(1)	2(1)	14(1)	0(1)
C(13)	39(1)	37(1)	37(1)	1(1)	18(1)	4(1)
C(14)	43(1)	45(2)	44(1)	-2(1)	26(1)	-2(1)
C(15)	50(1)	38(1)	41(1)	-1(1)	24(1)	-9(1)
C(16)	42(1)	29(1)	42(1)	3(1)	16(1)	-2(1)
C(17)	34(1)	30(1)	35(1)	-2(1)	14(1)	-1(1)
C(18)	34(1)	30(1)	29(1)	-2(1)	14(1)	-1(1)
N(1)	38(1)	36(1)	41(1)	-3(1)	18(1)	-4(1)
O(1)	69(1)	54(1)	54(1)	-3(1)	30(1)	-22(1)
O(2)	56(1)	97(2)	43(1)	-15(1)	16(1)	-28(1)
O(3)	69(2)	78(2)	44(1)	-15(1)	22(1)	-42(1)
Si(1)	36(1)	26(1)	27(1)	0(1)	14(1)	2(1)

Table 13. Hydrogen coordinates (x 10^4) and isotropic displacement parameters (A^2 x 10^3) for 2a.

	x	y	z	U(eq)
H(1A)	4356	1584	3729	50
H(1B)	3485	1087	2697	50
H(2A)	3856	388	4793	43
H(2B)	3130	-440	3768	43
H(3A)	2161	3857	5230	48
H(3B)	3191	3391	5790	48
H(4A)	2373	5252	3887	54
H(4B)	3167	5819	4980	54
H(5)	4199	4346	4585	67
H(6)	2904	3581	2687	64
H(7)	1839	324	2255	49
H(8)	572	454	671	64
H(9)	-666	1937	562	67
H(10)	-603	3340	2030	65
H(11)	671	3219	3623	53
H(13)	1051	1358	5201	44
H(14)	717	-227	6359	50
H(15)	1605	-2418	7146	49
H(16)	2821	-3049	6764	46
H(17)	3165	-1446	5619	40

Table 14. Torsion angles [deg] for 2a.

C(6)-C(1)-C(2)-Si(1)	13.1(3)
Si(1)-C(3)-C(4)-C(5)	-48.0(3)
C(3)-C(4)-C(5)-C(6)	79.8(4)
C(3)-C(4)-C(5)-Ag(1)	166.3(2)
C(4)-C(5)-C(6)-C(1)	-126.3(4)
Ag(1)-C(5)-C(6)-C(1)	117.4(3)
C(4)-C(5)-C(6)-Ag(1)	116.3(3)
C(2)-C(1)-C(6)-C(5)	66.6(4)
C(2)-C(1)-C(6)-Ag(1)	157.3(2)
C(12)-C(7)-C(8)-C(9)	0.2(5)
C(7)-C(8)-C(9)-C(10)	1.1(5)
C(8)-C(9)-C(10)-C(11)	-1.0(5)
C(9)-C(10)-C(11)-C(12)	-0.4(5)
C(10)-C(11)-C(12)-C(7)	1.8(4)
C(10)-C(11)-C(12)-Si(1)	-178.2(2)
C(8)-C(7)-C(12)-C(11)	-1.7(4)
C(8)-C(7)-C(12)-Si(1)	178.3(2)
C(18)-C(13)-C(14)-C(15)	-0.5(4)
C(13)-C(14)-C(15)-C(16)	-0.4(4)
C(14)-C(15)-C(16)-C(17)	0.8(4)
C(15)-C(16)-C(17)-C(18)	-0.2(4)
C(14)-C(13)-C(18)-C(17)	1.1(4)
C(14)-C(13)-C(18)-Si(1)	-177.4(2)
C(16)-C(17)-C(18)-C(13)	-0.7(4)
C(16)-C(17)-C(18)-Si(1)	177.74(19)
O(2)-N(1)-O(1)-Ag(1)	24.1(4)
O(3)-N(1)-O(1)-Ag(1)	-158.1(2)
O(2)-N(1)-O(3)-Ag(1)#2	-15.6(4)
O(1)-N(1)-O(3)-Ag(1)#2	166.6(2)
C(11)-C(12)-Si(1)-C(18)	75.2(2)
C(7)-C(12)-Si(1)-C(18)	-104.7(2)
C(11)-C(12)-Si(1)-C(2)	-167.0(2)
C(7)-C(12)-Si(1)-C(2)	13.1(2)
C(11)-C(12)-Si(1)-C(3)	-39.1(3)
C(7)-C(12)-Si(1)-C(3)	141.0(2)
C(13)-C(18)-Si(1)-C(12)	-54.5(2)
C(17)-C(18)-Si(1)-C(12)	127.1(2)
C(13)-C(18)-Si(1)-C(2)	-172.6(2)
C(17)-C(18)-Si(1)-C(2)	9.0(2)
C(13)-C(18)-Si(1)-C(3)	62.0(2)
C(17)-C(18)-Si(1)-C(3)	-116.4(2)

Symmetry transformations used to generate equivalent atoms:
#1 -x+1,y-1/2,-z+1/2   #2 -x+1,y+1/2,-z+1/2

X-ray structure of *rel*-(1*R*, 4*E*, p*R*)-Cyclooct-4-enol •$AgNO_3$ (3)

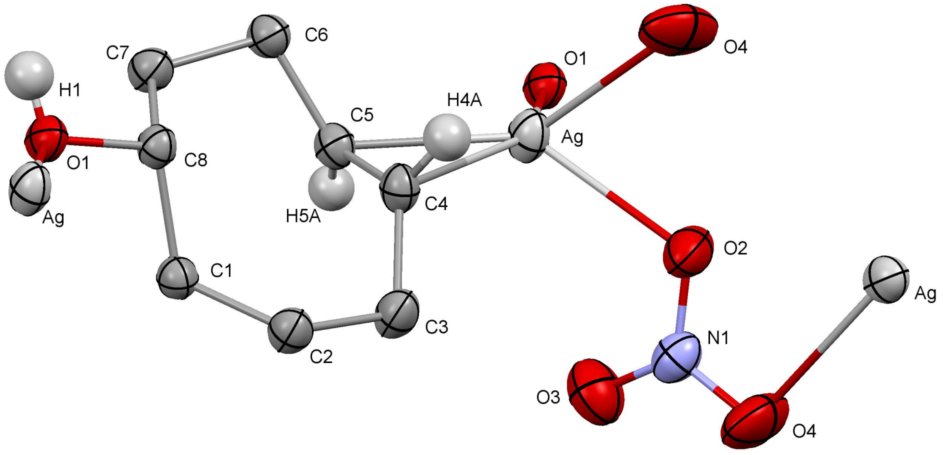

Fig.S24. X-ray structure of *rel*-(1*R*, 4*E*, p*R*)-Cyclooct-4-enol •$AgNO_3$ (3)

Table 15. Crystal data and structure refinement for *rel*-(1*R*, 4*E*, p*R*)-Cyclooct-4-enol •$AgNO_3$ (3)

Bond precision: C-C = 0.0020 A Wavelength=0.71073
Cell: a=7.786(3) b=8.024(3) c=16.081(5)
alpha=90 beta=90 gamma=90
Temperature: 200 K

**Calculated**	**Reported**
Volume 1004.7(6)	1004.6(6)
Space group P 21 21 21	P2(1)2(1)2(
Hall group P 2ac 2ab	NA
Moiety formula C8 H14 Ag N O4	NA
Sum formula C8 H14 Ag N O4	C8 H14 Ag N O4
Mr 296.07	296.07
Dx,g cm^{-3} 1.957	1.957
Z 4	4
Mu (mm^{-1}) 1.995	1.995
F000 592.0	592.0
F000' 588.67	

h,k,lmax 10,10,21 10,10,21
Nref 2491[1455] 2483
Tmin,Tmax 0.566,0.698 0.598,0.714
Tmin' 0.555

Correction method= # Reported T Limits: Tmin=0.598 Tmax=0.714
AbsCorr = MULTI-SCAN
Data completeness= 1.71/1.00 Theta(max)= 28.280
R(reflections)= 0.0162(2467) wR2(reflections)= 0.0442(2483)
S = 1.028 Npar= 131

Table 16. Atomic coordinates (x 10^4) and equivalent isotropic displacement parameters (A^2 x 10^3) for 3. U(eq) is defined as one third of the trace of the orthogonalized Uij tensor.

	x	y	z	U(eq)
Ag	1331(1)	11594(1)	9179(1)	33(1)
N(1)	5156(2)	12638(2)	8997(1)	34(1)
O(1)	-210(2)	3749(2)	8557(1)	30(1)
O(2)	4046(2)	12651(2)	9567(1)	45(1)
O(3)	4772(3)	12314(2)	8285(1)	53(1)
O(4)	6652(2)	12996(3)	9229(1)	65(1)
C(1)	1932(2)	5736(2)	8166(1)	26(1)
C(2)	3548(2)	6410(2)	8606(1)	30(1)
C(3)	3598(2)	8325(2)	8731(1)	34(1)
C(4)	1906(2)	8786(2)	9107(1)	28(1)
C(5)	499(2)	9036(2)	8615(1)	26(1)
C(6)	-1224(2)	8358(2)	8843(1)	28(1)
C(7)	-1285(2)	6580(2)	8478(1)	29(1)
C(8)	279(2)	5477(2)	8693(1)	25(1)

Table 17. Bond lengths [A] and angles [deg] for 3.

Ag-C(4)	2.3002(18)
Ag-O(1)#1	2.3305(14)
Ag-C(5)	2.3356(18)
Ag-O(2)	2.3622(16)
Ag-O(4)#2	2.593(2)
N(1)-O(3)	1.212(2)
N(1)-O(4)	1.256(2)
N(1)-O(2)	1.260(2)
O(1)-C(8)	1.454(2)
O(1)-Ag#3	2.3305(14)
O(4)-Ag#4	2.593(2)
C(1)-C(2)	1.541(2)
C(1)-C(8)	1.555(2)
C(2)-C(3)	1.550(3)
C(3)-C(4)	1.496(2)
C(4)-C(5)	1.366(2)
C(5)-C(6)	1.493(2)
C(6)-C(7)	1.543(3)
C(7)-C(8)	1.545(2)
C(4)-Ag-O(1)#1	143.66(5)
C(4)-Ag-C(5)	34.25(6)
O(1)#1-Ag-C(5)	110.06(6)
C(4)-Ag-O(2)	101.00(6)
O(1)#1-Ag-O(2)	107.93(6)
C(5)-Ag-O(2)	131.79(6)
C(4)-Ag-O(4)#2	98.93(7)
O(1)#1-Ag-O(4)#2	112.28(6)
C(5)-Ag-O(4)#2	121.43(7)
O(2)-Ag-O(4)#2	66.86(6)
O(3)-N(1)-O(4)	123.95(19)
O(3)-N(1)-O(2)	121.37(17)
O(4)-N(1)-O(2)	114.68(17)
C(8)-O(1)-Ag#3	120.52(10)
N(1)-O(2)-Ag	114.72(11)
N(1)-O(4)-Ag#4	103.48(12)
C(2)-C(1)-C(8)	118.25(14)
C(1)-C(2)-C(3)	115.34(14)
C(4)-C(3)-C(2)	105.98(15)
C(5)-C(4)-C(3)	120.58(16)
C(5)-C(4)-Ag	74.29(10)
C(3)-C(4)-Ag	115.71(12)

C(4)-C(5)-C(6)	121.67(15)
C(4)-C(5)-Ag	71.45(10)
C(6)-C(5)-Ag	118.28(11)
C(5)-C(6)-C(7)	105.69(13)
C(6)-C(7)-C(8)	114.88(13)
O(1)-C(8)-C(7)	107.86(13)
O(1)-C(8)-C(1)	105.20(13)
C(7)-C(8)-C(1)	116.95(13)

Symmetry transformations used to generate equivalent atoms: #1 x,y+1,z #2 x-1/2,-y+5/2,-z+2 #3 x,y-1,z #4 x+1/2,-y+5/2,-z+2

Table 18. Anisotropic displacement parameters (A^2 x 10^3) for 3.

The anisotropic displacement factor exponent takes the form: -2 pi^2 [h^2 a*^2 U11 + ... + 2 h k a* b* U12]

	U11	U22	U33	U23	U13	U12
Ag	32(1)	22(1)	45(1)	-2(1)	-5(1)	-1(1)
N(1)	31(1)	31(1)	41(1)	7(1)	-3(1)	-2(1)
O(1)	30(1)	22(1)	37(1)	0(1)	1(1)	-3(1)
O(2)	40(1)	57(1)	39(1)	-3(1)	3(1)	-16(1)
O(3)	71(1)	52(1)	38(1)	-4(1)	-1(1)	-1(1)
O(4)	31(1)	101(2)	63(1)	2(1)	-2(1)	-16(1)
C(1)	28(1)	24(1)	27(1)	-1(1)	1(1)	0(1)
C(2)	26(1)	26(1)	38(1)	-1(1)	-1(1)	2(1)
C(3)	27(1)	28(1)	47(1)	-2(1)	-3(1)	-2(1)
C(4)	31(1)	21(1)	32(1)	-2(1)	-4(1)	0(1)
C(5)	30(1)	19(1)	28(1)	1(1)	0(1)	1(1)
C(6)	28(1)	24(1)	33(1)	-1(1)	2(1)	1(1)
C(7)	26(1)	25(1)	37(1)	0(1)	0(1)	1(1)
C(8)	26(1)	20(1)	29(1)	0(1)	-1(1)	0(1)

Table 19. Hydrogen coordinates (x 10^4) and isotropic displacement parameters (A^2 x 10^3) for 3.

	x	y	z	U(eq)
H(1)	-1100(40)	3630(30)	8747(15)	35
H(1A)	2234	4653	7911	31
H(1B)	1647	6510	7707	31
H(2A)	4568	6078	8278	36
H(2B)	3641	5870	9157	36
H(3A)	4551	8640	9107	41

H(3B)	3760	8900	8192	41
H(4A)	1809	8905	9693	33
H(5A)	620	9656	8115	31
H(6A)	-2149	9055	8603	34
H(6B)	-1364	8328	9454	34
H(7A)	-1375	6664	7865	35
H(7B)	-2337	6021	8681	35
H(8A)	568	5631	9294	30

Table 20. Torsion angles [deg] for 3.

O(3)-N(1)-O(2)-Ag	9.3(2)
O(4)-N(1)-O(2)-Ag	-171.29(17)
C(4)-Ag-O(2)-N(1)	73.18(15)
O(1)#1-Ag-O(2)-N(1)	-84.55(14)
C(5)-Ag-O(2)-N(1)	56.04(16)
O(4)#2-Ag-O(2)-N(1)	168.28(16)
O(3)-N(1)-O(4)-Ag#4	-178.33(17)
O(2)-N(1)-O(4)-Ag#4	2.3(2)
C(8)-C(1)-C(2)-C(3)	-81.3(2)
C(1)-C(2)-C(3)-C(4)	49.4(2)
C(2)-C(3)-C(4)-C(5)	-83.8(2)
C(2)-C(3)-C(4)-Ag	-170.07(11)
O(1)#1-Ag-C(4)-C(5)	-14.48(15)
O(2)-Ag-C(4)-C(5)	-157.01(10)
O(4)#2-Ag-C(4)-C(5)	134.99(10)
O(1)#1-Ag-C(4)-C(3)	102.33(14)
C(5)-Ag-C(4)-C(3)	116.81(17)
O(2)-Ag-C(4)-C(3)	-40.20(13)
O(4)#2-Ag-C(4)-C(3)	-108.19(13)
C(3)-C(4)-C(5)-C(6)	136.73(17)
Ag-C(4)-C(5)-C(6)	-112.35(15)
C(3)-C(4)-C(5)-Ag	-110.93(16)
O(1)#1-Ag-C(5)-C(4)	170.92(10)
O(2)-Ag-C(5)-C(4)	30.94(13)
O(4)#2-Ag-C(5)-C(4)	-54.96(12)
C(4)-Ag-C(5)-C(6)	116.64(16)
O(1)#1-Ag-C(5)-C(6)	-72.44(13)
O(2)-Ag-C(5)-C(6)	147.58(11)
O(4)#2-Ag-C(5)-C(6)	61.68(14)
C(4)-C(5)-C(6)-C(7)	-87.69(19)
Ag-C(5)-C(6)-C(7)	-172.39(10)
C(5)-C(6)-C(7)-C(8)	50.30(18)

Ag#3-O(1)-C(8)-C(7)	-160.27(10)
Ag#3-O(1)-C(8)-C(1)	74.21(14)
C(6)-C(7)-C(8)-O(1)	161.22(13)
C(6)-C(7)-C(8)-C(1)	-80.56(18)
C(2)-C(1)-C(8)-O(1)	-125.48(16)
C(2)-C(1)-C(8)-C(7)	114.87(16)

Symmetry transformations used to generate equivalent atoms: #1 x,y+1,z #2 x-1/2,-y+5/2,-z+2 #3 x,y-1,z #4 x+1/2,-y+5/2,-z+2

Table 21. Hydrogen bonds for 3 [A and deg.].

D-H...A	d(D-H)	d(H...A)	d(D...A)	<(DHA)
O(1)-H(1)...O(4)#5	0.76(3)	1.98(3)	2.739(2)	172(3)

Symmetry transformations used to generate equivalent atoms: #1 x,y+1,z #2 x-1/2,-y+5/2,-z+2 #3 x,y-1,z #4 x+1/2,-y+5/2,-z+2 #5 x-1,y-1,z

Computational Studies

Gaussian reference:
Gaussian 09, Revision A.02,
M. J. Frisch, G. W. Trucks, H. B. Schlegel, G. E. Scuseria,
M. A. Robb, J. R. Cheeseman, G. Scalmani, V. Barone, B. Mennucci,
G. A. Petersson, H. Nakatsuji, M. Caricato, X. Li, H. P. Hratchian,
A. F. Izmaylov, J. Bloino, G. Zheng, J. L. Sonnenberg, M. Hada,
M. Ehara, K. Toyota, R. Fukuda, J. Hasegawa, M. Ishida, T. Nakajima,
Y. Honda, O. Kitao, H. Nakai, T. Vreven, J. A. Montgomery, Jr.,
J. E. Peralta, F. Ogliaro, M. Bearpark, J. J. Heyd, E. Brothers,
K. N. Kudin, V. N. Staroverov, R. Kobayashi, J. Normand,
K. Raghavachari, A. Rendell, J. C. Burant, S. S. Iyengar, J. Tomasi,
M. Cossi, N. Rega, J. M. Millam, M. Klene, J. E. Knox, J. B. Cross,
V. Bakken, C. Adamo, J. Jaramillo, R. Gomperts, R. E. Stratmann,
O. Yazyev, A. J. Austin, R. Cammi, C. Pomelli, J. W. Ochterski,
R. L. Martin, K. Morokuma, V. G. Zakrzewski, G. A. Voth,
P. Salvador, J. J. Dannenberg, S. Dapprich, A. D. Daniels,
O. Farkas, J. B. Foresman, J. V. Ortiz, J. Cioslowski,
and D. J. Fox, Gaussian, Inc., Wallingford CT, 2009.

Pre-reaction complex of trans-cyclooctene with 3,6-diphenyl-s-tetrazine optimized at the M06L/6-311+G(d,p) level of theory

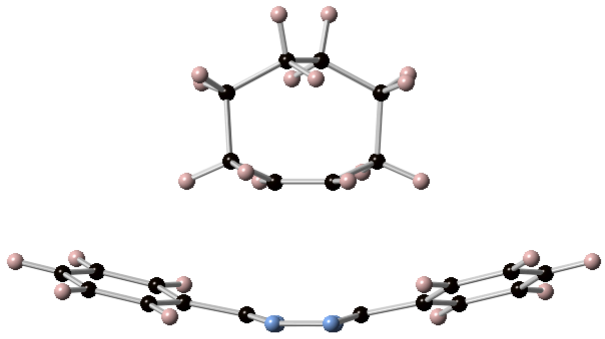

Electronic Energy -1071.83471322 au
Sum of electronic and zero-point Energies -1071.417219 au
Sum of electronic and thermal Energies -1071.394458 au
Sum of electronic and thermal Enthalpies -1071.393514 au
Sum of electronic and thermal Free Energies -1071.470561 au

Atomic Coordinates in Angstroms.

Atomic Number	X	Y	Z
6	-0.362610	3.919674	0.687390
6	0.362405	3.919724	-0.687105
6	-1.622304	1.656411	0.928082
6	1.622247	1.656563	-0.927932
6	-0.660249	1.200787	-0.108721
6	0.660217	1.200812	0.108836
6	-1.715074	3.194865	0.772151
6	1.714918	3.195011	-0.771898
1	-0.533840	4.960322	0.983711
1	0.533567	4.960397	-0.983384
1	-1.243578	1.413660	1.929022
1	1.243539	1.413857	-1.928890
1	-1.011492	1.220711	-1.144037
1	1.011458	1.220676	1.144153
1	0.308656	3.515095	1.455859
1	-0.308834	3.515133	-1.455591
1	-2.620560	1.216194	0.836712
1	2.620530	1.216400	-0.836588
1	-2.294937	3.596895	1.611212
1	2.294760	3.597131	-1.610930
1	-2.298775	3.423037	-0.129421
1	2.298597	3.423165	0.129694
7	0.668630	-1.982176	-1.170423
7	-0.668557	-1.982342	1.170202
6	1.277901	-1.783032	0.015315
6	-1.277837	-1.783080	-0.015512
7	0.640008	-1.987502	1.188272
7	-0.639935	-1.987386	-1.188493
6	2.712205	-1.497509	0.030578

6	3.340626	-1.130039	1.226341
6	3.456605	-1.534542	-1.154912
6	4.688684	-0.802207	1.232529
1	2.756662	-1.100195	2.139841
6	4.806018	-1.213071	-1.140018
1	2.959518	-1.814032	-2.077406
6	5.425128	-0.843028	0.051114
1	5.168590	-0.511683	2.161703
1	5.378212	-1.247400	-2.061405
1	6.479311	-0.584833	0.058644
6	-2.712151	-1.497612	-0.030738
6	-3.340592	-1.130031	-1.226457
6	-3.456545	-1.534811	1.154750
6	-4.688663	-0.802251	-1.232601
1	-2.756634	-1.100060	-2.139956
6	-4.805970	-1.213391	1.139900
1	-2.959443	-1.814388	2.077210
6	-5.425100	-0.843236	-0.051188
1	-5.168585	-0.511640	-2.161740
1	-5.378159	-1.247848	2.061285
1	-6.479293	-0.585080	-0.058683

TS for the reaction of *trans*-cyclooctene with 3,6-diphenyl-s-tetrazine
M06L/6-311+G(d,p)

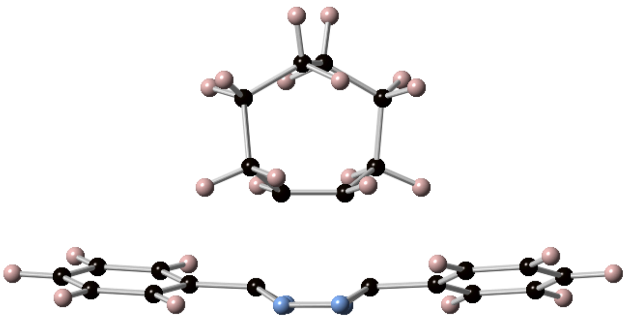

Electronic Energy -1071.81350424 au
Sum of electronic and zero-point Energies -1071.395018 au
Sum of electronic and thermal Energies -1071.373860 au
Sum of electronic and thermal Enthalpies -1071.372916 au
Sum of electronic and thermal Free Energies -1071.444919 au
Imaginary Frequency 513.5374i cm-1

Atomic Coordinates in Angstroms.

Atomic Number	X	Y	Z
6	2.983914	-0.600357	-0.489833
6	2.983882	0.600462	0.489979
6	0.709077	-1.835888	-0.240033
6	0.709013	1.835903	0.240014
6	0.152327	-0.589794	0.370744

6	0.152307	0.589806	-0.370791
6	2.234493	-1.859072	-0.040335
6	2.234451	1.859157	0.040471
1	4.023552	-0.889366	-0.674798
1	4.023503	0.889513	0.674968
1	0.474766	-1.850858	-1.311428
1	0.474613	1.850877	1.311389
1	0.319527	-0.480050	1.443788
1	0.319546	0.480060	-1.443835
1	2.605954	-0.280728	-1.470436
1	2.605886	0.280827	1.470566
1	0.279444	-2.741911	0.198584
1	0.279369	2.741897	-0.198650
1	2.627836	-2.729313	-0.577283
1	2.627698	2.729367	0.577541
1	2.450576	-2.043499	1.020175
1	2.450661	2.043673	-1.019995
7	-2.315259	1.207313	0.583782
7	-2.315251	-1.207451	-0.583862
6	-1.915484	1.021933	-0.728825
6	-1.915439	-1.022048	0.728720
7	-2.306113	-0.167996	-1.330339
7	-2.306086	0.167856	1.330259
6	-1.855206	2.208895	-1.602095
6	-1.556649	2.054010	-2.958721
6	-2.064616	3.492839	-1.089709
6	-1.473219	3.162724	-3.789689
1	-1.406988	1.054057	-3.354229
6	-1.983492	4.599505	-1.926097
1	-2.303172	3.605919	-0.037479
6	-1.687423	4.439008	-3.275993
1	-1.245247	3.031928	-4.842873
1	-2.155058	5.592235	-1.521838
1	-1.625123	5.305421	-3.926686
6	-1.855076	-2.208972	1.602001
6	-1.556350	-2.054018	2.958580
6	-2.064580	-3.492931	1.089699
6	-1.472797	-3.162692	3.789586
1	-1.406655	-1.054035	3.354010
6	-1.983336	-4.599558	1.926129
1	-2.303289	-3.606060	0.037510
6	-1.687065	-4.439001	3.275973
1	-1.244679	-3.031846	4.842733
1	-2.154965	-5.592306	1.521940
1	-1.624677	-5.305385	3.926697

Product of the reaction of trans-cyclooctene with 3,6-diphenyl-s-tetrazine
M06L/6-311+G(d,p)

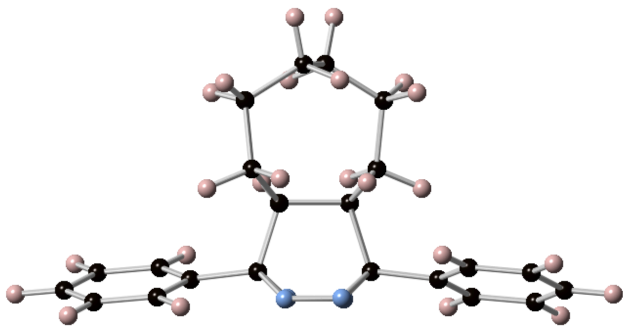

Electronic Energy -1071.85389431 au
Sum of electronic and zero-point Energies -1071.431939 au
Sum of electronic and thermal Energies -1071.411322 au
Sum of electronic and thermal Enthalpies -1071.410377 au
Sum of electronic and thermal Free Energies -1071.480812 au

Atomic Coordinates in Angstroms.

Atomic Number	X	Y	Z
6	0.212501	3.633694	-0.311927
6	-0.269423	3.485299	1.145961
6	1.367779	1.410864	-1.026075
6	-1.344232	1.128555	1.420794
6	0.789941	0.553243	0.100991
6	-0.740633	0.521349	0.153218
6	1.494587	2.891797	-0.690953
6	-1.523757	2.640811	1.372766
1	-0.478792	4.486224	1.536420
1	0.750872	1.280199	-1.924494
1	-0.720033	0.851270	2.280045
1	1.186728	0.904801	1.062221
1	-1.152551	1.035131	-0.725035
1	0.549642	3.107858	1.773455
1	2.363348	1.039694	-1.289402
1	-2.325267	0.679483	1.605625
1	1.941853	3.384393	-1.561496
1	-1.984987	2.943125	2.319546
1	2.226960	3.012106	0.119426
1	-2.262509	2.887391	0.597539
7	-0.570322	-1.692300	1.108465
7	0.693588	-1.443196	-1.257972
6	-1.187129	-0.981135	-0.019983
6	1.289021	-0.938299	-0.012944
7	-0.546921	-1.451761	-1.271116
7	0.669757	-1.660013	1.123886
6	-2.672988	-1.178201	-0.090905
6	-3.347140	-0.815570	-1.257733
6	-3.399261	-1.680877	0.988416
6	-4.727070	-0.951802	-1.344910

1	-2.780191	-0.444088	-2.106247
6	-4.780777	-1.817204	0.898761
1	-2.872850	-1.970846	1.891372
6	-5.448135	-1.452068	-0.265043
1	-5.240246	-0.671636	-2.259508
1	-5.336915	-2.212107	1.743040
1	-6.526028	-1.559389	-0.332566
6	2.781071	-1.093436	0.024133
6	3.446153	-0.936493	1.240891
6	3.520736	-1.355626	-1.128803
6	4.830279	-1.038726	1.305052
1	2.869505	-0.753404	2.142746
6	4.906450	-1.458378	-1.062218
1	3.001671	-1.486427	-2.072196
6	5.564641	-1.298694	0.152046
1	5.336496	-0.920261	2.257964
1	5.473127	-1.665555	-1.964433
1	6.645839	-1.379337	0.201440
1	0.385738	4.697200	-0.504175
1	-0.595057	3.354196	-1.002356

Pre-reaction complex of Si-TCH 16 with 3,6-diphenyl-s-tetrazine
M06L/6-311+G(d,p)

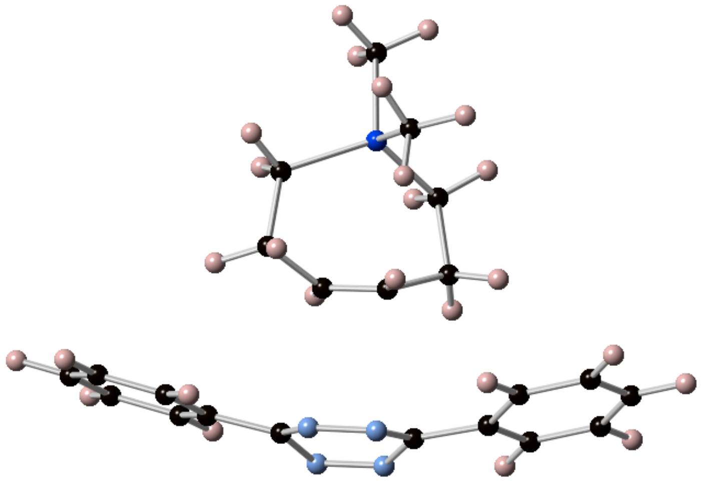

Electronic Energy= -1362.561286 au
Sum of electronic and zero-point Energies= -1362.128808
Sum of electronic and thermal Energies= -1362.101889
Sum of electronic and thermal Enthalpies= -1362.100945
Sum of electronic and thermal Free Energies= -1362.187796

N -2.0591609006,-0.9413334297,3.1197203745
N 0.5555954705,-1.5815544352,3.1807700955
C -1.4260781196,-1.5082030528,2.0723911318

C 0.0141793329,-0.6612848375,4.0043844321
N -0.1827576189,-2.0211778429,2.1932649087
N -1.3241220282,-0.5123835364,4.1134476945
C -2.1638618552,-1.7169830521,0.8269984103
C -1.5128236735,-2.2397121348,-0.2972675001
C -3.5094051985,-1.3428879626,0.7310042963
C -2.1985334099,-2.3840878221,-1.4946652004
H -0.4686149874,-2.5229693016,-0.2146105248
C -4.1893395275,-1.4917897263,-0.4687919732
H -4.0036762576,-0.9350538499,1.6058600718
C -3.5371129502,-2.0106963758,-1.5843031076
H -1.687983265,-2.7876517635,-2.3632146887
H -5.232844844,-1.201015105,-0.5366040798
H -4.0711785184,-2.1236759698,-2.522529566
C 0.8857326672,0.0674412395,4.9254607993
C 0.3625261715,1.0969202243,5.7170084975
C 2.2542900746,-0.2211840667,4.988707276
C 1.1949478946,1.8236753359,6.5553885133
H -0.6982577766,1.3159082613,5.6581497192
C 3.0802395686,0.5059664601,5.8336871691
H 2.6534611769,-1.0160679514,4.3685032092
C 2.5549391909,1.5300729547,6.6174765362
H 0.7832868894,2.6226368153,7.1635727355
H 4.1397953257,0.2757523089,5.8808342807
H 3.2046915883,2.0994118568,7.2746670609
C -0.2606226689,1.0051119402,0.9716692741
C 0.1978841782,1.6626860367,2.0430056687
C -1.4163033305,1.5253788627,0.1701734274
C 1.655825248,1.9688303,2.1620620225
H -0.4785007088,2.3525279485,2.5577361855
C -0.9326233213,2.9023941349,-0.3752785299
H -2.3225653223,1.6438166996,0.7753924591
H -1.6714229733,0.8441742811,-0.6469249871
C 1.8195336166,3.2361967769,1.2829195685
H 2.2511691251,1.1421937138,1.754297449
H 1.9980819624,2.1484056142,3.1866838474
H -1.3312953199,3.6934948044,0.2750546372
H -1.3519235182,3.1074275042,-1.368947862
Si 0.959879327,3.214017397,-0.4317991321
H 1.4085976558,4.0968134455,1.8299680426
H 2.8830348706,3.4713727153,1.1387767994
C 1.1815088862,4.9329608724,-1.1593756478
C 1.8213723668,1.9789884924,-1.5604960667
H 2.2379857194,5.2088393971,-1.2272993278
H 0.7632007134,4.9989284902,-2.1681923912
H 0.6852937899,5.6959666815,-0.5521161999
H 1.8851768238,0.9758613631,-1.1325514439
H 1.3038361387,1.8913905199,-2.5208367431
H 2.8447140101,2.3039395461,-1.7736649015
H 0.442338011,0.362650292,0.4361698798

TS for the reaction of Si-TCH 16 with 3,6-diphenyl-s-tetrazine M06L/6-311+G(d,p)

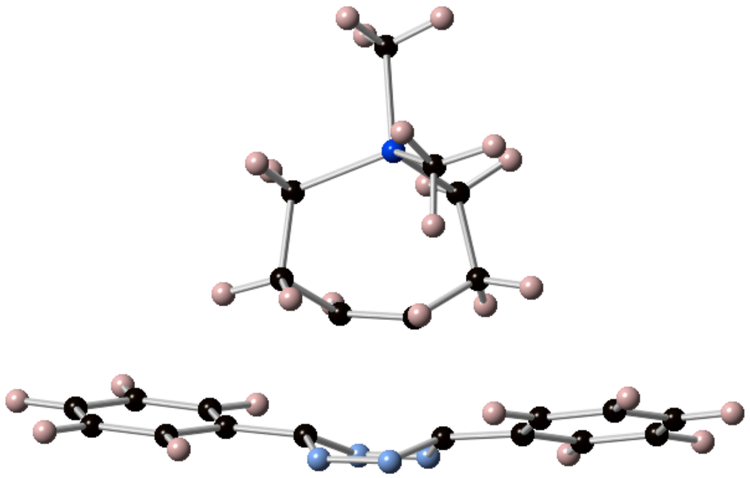

Electronic Energy= - 1362.5464985 a.u.
Sum of electronic and zero-point Energies= -1362.113021
Sum of electronic and thermal Energies= -1362.087746
Sum of electronic and thermal Enthalpies= -1362.086802
Sum of electronic and thermal Free Energies= -1362.167910
Im.Freq.= 451.9951*i* cm^{-1}

N 0.4932891429,-2.2104121901,-0.8792039395
N -0.8590980544,-1.7430719958,1.3891945116
C 1.1011664822,-1.6941969833,0.2485140962
C -1.4063263322,-1.510178564,0.1448557785
N 0.4191424075,-1.8434137425,1.4460977143
N -0.7845377625,-2.1092743997,-0.9356079031
C 2.5734832102,-1.7083223795,0.3032017031
C 3.2308730899,-1.2555846261,1.4512090191
C 3.3259058901,-2.1092813596,-0.8042798774
C 4.6172316929,-1.2004920636,1.4880233232
H 2.6419822362,-0.9548595518,2.3125400912
C 4.7130603801,-2.0570520541,-0.7606881274
H 2.8087141581,-2.4623212695,-1.6897127098
C 5.3630206074,-1.5990143962,0.3814459139
H 5.1196636181,-0.8474306152,2.3829948006
H 5.2904249787,-2.3741823288,-1.6234131979
H 6.4471002992,-1.5546009576,0.4108440862
C -2.8583651675,-1.292666216,0.0502956089
C -3.4612944927,-1.1739076833,-1.205715982
C -3.6428119786,-1.1647626737,1.2011549241
C -4.8249714034,-0.9369694777,-1.3085389847
H -2.850398822,-1.2822206155,-2.0963050915
C -5.0077756619,-0.9312034597,1.092593291
H -3.1693866205,-1.2590052529,2.1725719573

C -5.6028385293,-0.8155357285,-0.1600975887
H -5.2845302126,-0.849529667,-2.2879002013
H -5.6106118011,-0.8398656193,1.9906819574
H -6.6692804131,-0.6306976771,-0.2414978686
C 0.7386850556,0.4164807472,-0.0468867467
C -0.631414971,0.5521558179,-0.1837180291
C 1.6556026816,0.8355297417,-1.1652077887
C -1.3458300745,1.4128270591,0.8151260917
H -1.0317473969,0.5698227101,-1.2003942653
C 1.380537074,2.3308493244,-1.4561428818
H 1.4938123496,0.2247053975,-2.0609423579
H 2.6977912637,0.6898109927,-0.8674084177
C -1.0814010601,2.8791988552,0.4066220105
H -0.9396281134,1.2183438729,1.8154458336
H -2.421095473,1.2121637449,0.8543448244
H 0.6260189813,2.4057590847,-2.2515800569
H 2.2791388641,2.810248616,-1.8631345942
Si 0.7002352667,3.3993395347,-0.0309855433
H -1.6857164377,3.1086102611,-0.4826407683
H -1.4605160901,3.5503732952,1.1881301247
C 0.6056928824,5.164408968,-0.6613930628
C 1.8282210295,3.3548623534,1.4732732511
H 0.1746030381,5.8360257679,0.0868623252
H 1.5956577388,5.5546625648,-0.9151708288
H -0.0133689583,5.2391915506,-1.5604115187
H 1.8417131864,2.3891751981,1.9854691405
H 2.8607327749,3.5840919535,1.1915983659
H 1.5222376209,4.1048226702,2.2093578737
H 1.1182879459,0.6408283766,0.951138274

Pre-reaction complex of a conformationally strained ‘s-TCO’ derivative 17 with 3,6-diphenyl-s-tetrazine
M06L/6-311+G(d,p)

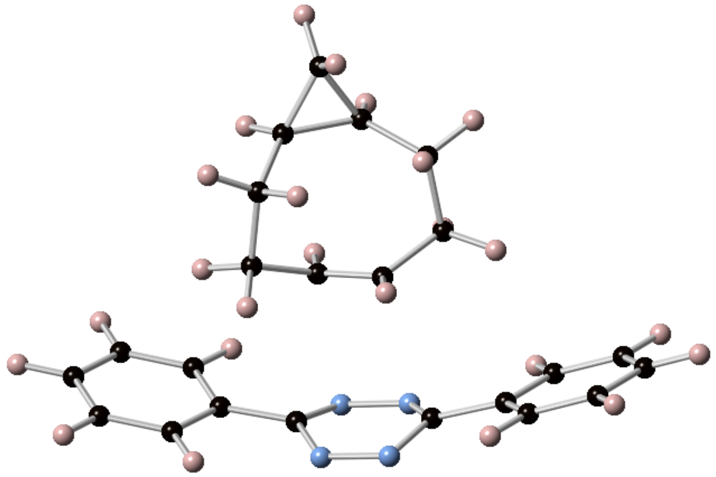

Electronic energy= -1109.9161079 a.u.
Sum of electronic and zero-point Energies= -1109.493766
Sum of electronic and thermal Energies= -1109.470604
Sum of electronic and thermal Enthalpies= -1109.469659
Sum of electronic and thermal Free Energies= -1109.547757

C 0.054192969,3.7299347992,-0.7456127781
C 1.6234847895,1.6861278156,-0.4922836044
C -1.7474223847,1.3062312677,0.9921391952
C 0.6314590783,1.0214505227,0.3983620029
C -0.6542024091,0.9344195456,0.042132319
C 1.3867613319,3.2008438823,-0.2551851695
C -1.5546452597,2.82747091,1.2258759726
H 1.444171095,1.4382005887,-1.5462440263
H -1.6724479441,0.7729185355,1.9470481042
H 0.8748858923,1.0224459608,1.4653370495
H -0.8928714781,0.9935708582,-1.0221555152
H 2.6629637436,1.4318900932,-0.2625224713
H -2.7400709625,1.0946099785,0.5809113593
H 2.1850029686,3.7778529939,-0.7392785219
H -0.6980853328,2.9592182096,1.8993898717
H 1.4911978324,3.399983206,0.8196555773
H -2.422549525,3.256878251,1.7429358462
N -0.4749651536,-2.3570630241,0.9775899458
N 0.8165469674,-1.9379514221,-1.3481177314
C -1.116593139,-2.0073723249,-0.1562605257
C 1.4336408153,-1.8642653695,-0.1512679687
N -0.4901459115,-2.0164822209,-1.3525405173
N 0.8312575101,-2.2895137475,0.980668929
C -1.2970064575,3.5891717691,-0.0580679988
C -0.5956854996,4.9126625342,-0.0784940079
H -0.1458267815,5.236576146,0.856346288
H -0.9913005955,5.7233860828,-0.6801220583
H -2.1164006492,3.5026795873,-0.7711289002
H -0.0271715324,3.7179189326,-1.8316892883
C 2.8453775315,-1.4854227097,-0.1008452944
C 3.4800855518,-1.3209464271,1.1359516026
C 3.5593709482,-1.2356210402,-1.2790979938
C 4.8053420268,-0.9147804551,1.1915722829
H 2.9174290693,-1.5127891116,2.0432856054
C 4.8861696145,-0.8339724754,-1.2163978494
H 3.0593141768,-1.3599079066,-2.2332442002
C 5.5122721026,-0.6709446394,0.0166893876
H 5.290251542,-0.7866495961,2.1538719168
H 5.4355064501,-0.6442696493,-2.1330635555
H 6.5490574249,-0.3525387681,0.061584715
C -2.5615128607,-1.7846778253,-0.1094002243
C -3.2482260801,-1.3681443886,-1.2564439196
C -3.2605788351,-1.9330442213,1.0945091843
C -4.6092655591,-1.105297139,-1.1965796971
H -2.6982949888,-1.2525081193,-2.1844974708

C -4.6216339214,-1.6706152053,1.1466211175
H -2.7203203765,-2.2513028631,1.9793026025
C -5.2996128109,-1.2549918078,0.0035519779
H -5.1353836454,-0.780673443,-2.0887036002
H -5.1576593501,-1.7886462324,2.0829938835
H -6.3639339884,-1.0467003375,0.048014152

TS for the reaction of a conformationally strained 's-TCO' derivative 17 with 3,6-diphenyl-s-tetrazine M06L/6-311+G(d,p)

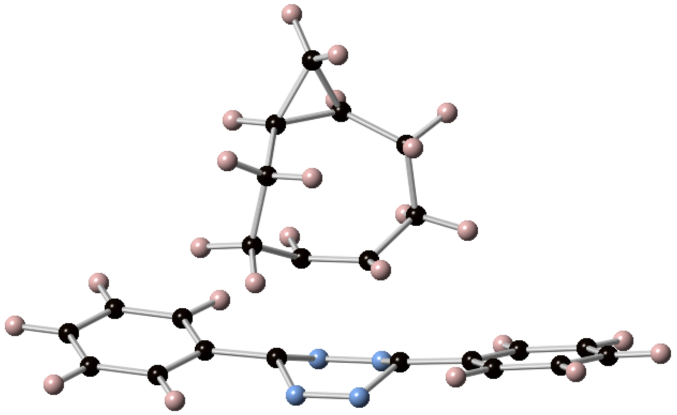

Electronic energy =-1109.8998725 a.u
Sum of electronic and zero-point Energies= -1109.477021
Sum of electronic and thermal Energies= -1109.455312
Sum of electronic and thermal Enthalpies= -1109.454367
Sum of electronic and thermal Free Energies= -1109.527447

C 2.7041979038,0.667192756,0.6145574515
C 0.5132134607,1.8336632982,-0.0909731517
C 0.476736118,-1.824205286,-0.2706943207
C -0.1536381013,0.5691660261,-0.5305936046
C -0.0899026652,-0.548591751,0.286452584
C 2.0297263589,1.6550568217,-0.3099010234
C 1.9418239586,-1.5474723807,-0.6604288473
H 0.3050287957,2.0273416448,0.9684905285
H -0.0919255723,-2.1563869045,-1.146911631
H -0.1274157326,0.3861939918,-1.6077521632
H 0.1416934093,-0.3536212969,1.334347619
H 0.1658498475,2.7005338159,-0.660094516
H 0.4266244597,-2.62757864,0.4711573112
H 2.5195335919,2.6293016693,-0.1917571495
H 1.9597450984,-0.9354332183,-1.570914408
H 2.2020900081,1.3692530924,-1.3556880636
H 2.4447420624,-2.4873691587,-0.9173813168
N -2.6557848603,-1.1868053987,-0.5050469428
N -2.5519187901,1.1953243343,0.7227371033
C -2.1377098476,-1.0347878155,0.7673172638
C -2.3027722494,1.050643522,-0.6276346695
N -2.4707745685,0.1335567067,1.4368203423

N -2.7325919856,-0.1249574506,-1.2195230146
C 2.6984000766,-0.8395000329,0.4379069205
C 3.9470082938,-0.0562979258,0.1734729563
H 4.2344177604,0.0716142569,-0.8666051222
H 4.7849331415,-0.1446701988,0.854971299
H 2.6907652173,-1.383770574,1.3814324167
H 2.6981772974,0.9840810318,1.6561168578
C -2.3064062913,2.2606744681,-1.4663393214
C -2.1892285691,2.1429113399,-2.8545696166
C -2.3880959968,3.5326045307,-0.8913501181
C -2.160841626,3.2777393533,-3.653437504
H -2.13535992,1.1536217986,-3.2973780863
C -2.3627510164,4.6650717029,-1.6954972085
H -2.4803528769,3.6158594861,0.186319205
C -2.248488927,4.5421090095,-3.0770208587
H -2.0734650156,3.1766807528,-4.7305370797
H -2.4347217478,5.6484656979,-1.2419923284
H -2.2295295721,5.4284531048,-3.7032260976
C -1.9650088783,-2.2488821397,1.5850009154
C -1.5442034836,-2.1362545088,2.9132191287
C -2.169149606,-3.5180716611,1.0359712089
C -1.3293890048,-3.274245726,3.678546126
H -1.3989320735,-1.1473057088,3.336708705
C -1.9573307781,-4.6537055513,1.807122182
H -2.4999095536,-3.5975220316,0.0059790511
C -1.534458213,-4.5366649582,3.1278836749
H -1.0045287453,-3.177766405,4.7098096238
H -2.1233878717,-5.6355442572,1.3752900844
H -1.3674849503,-5.4259196325,3.7273831451

Cellular experiments

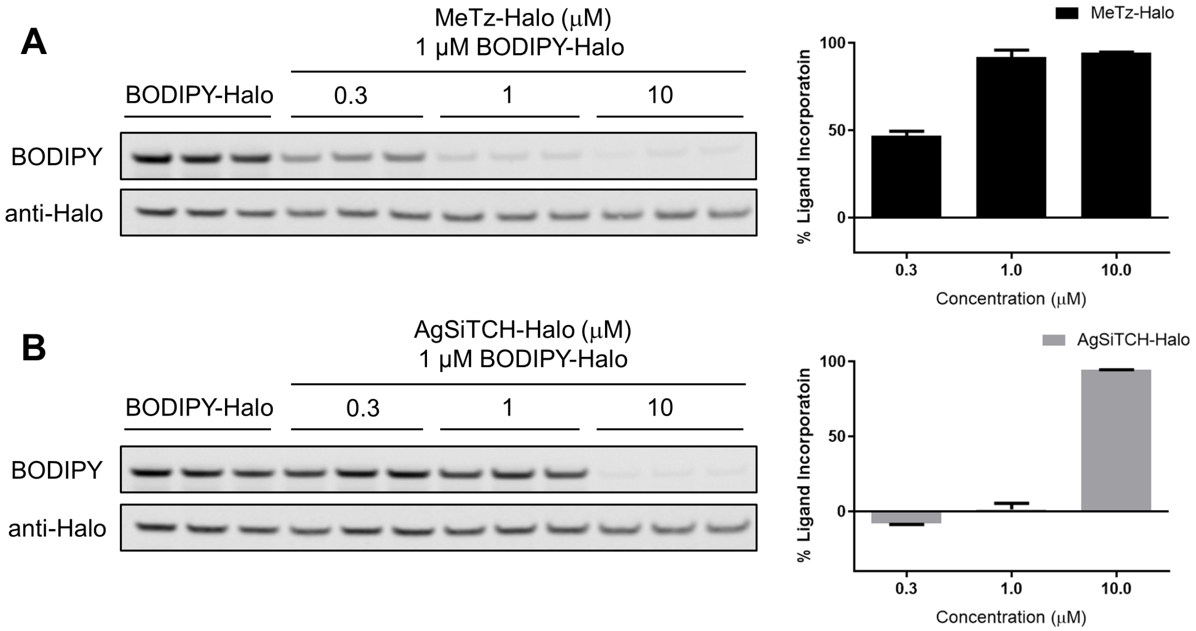

Fig.S25. Incorporation of MeTz-Halo and AgSiTCH-Halo into HaloTag protein, as measured using BODIPY-Halo as the probe. HEK 293T cells expressing HaloTag protein were treated with MeTz-Halo (A) or AgSiTCH-Halo (B) at 37°C for 30 min, followed by incubation with 1 µM BODIPY-Halo at 37°C for 30 min. The reactivity of MeTz-Halo and AgSi-TCH-Halo with HaloTag protein was analyzed with BODIPY in-gel fluorescence (top) and HaloTag Western blot (bottom). Control cells were treated only with 1 µM BODIPY-Halo. In-gel fluorescence signals were normalized by the corresponding HaloTag Western blot signals. The percentage of ligand incorporation was plotted as mean ± SEM from three biological replicates.

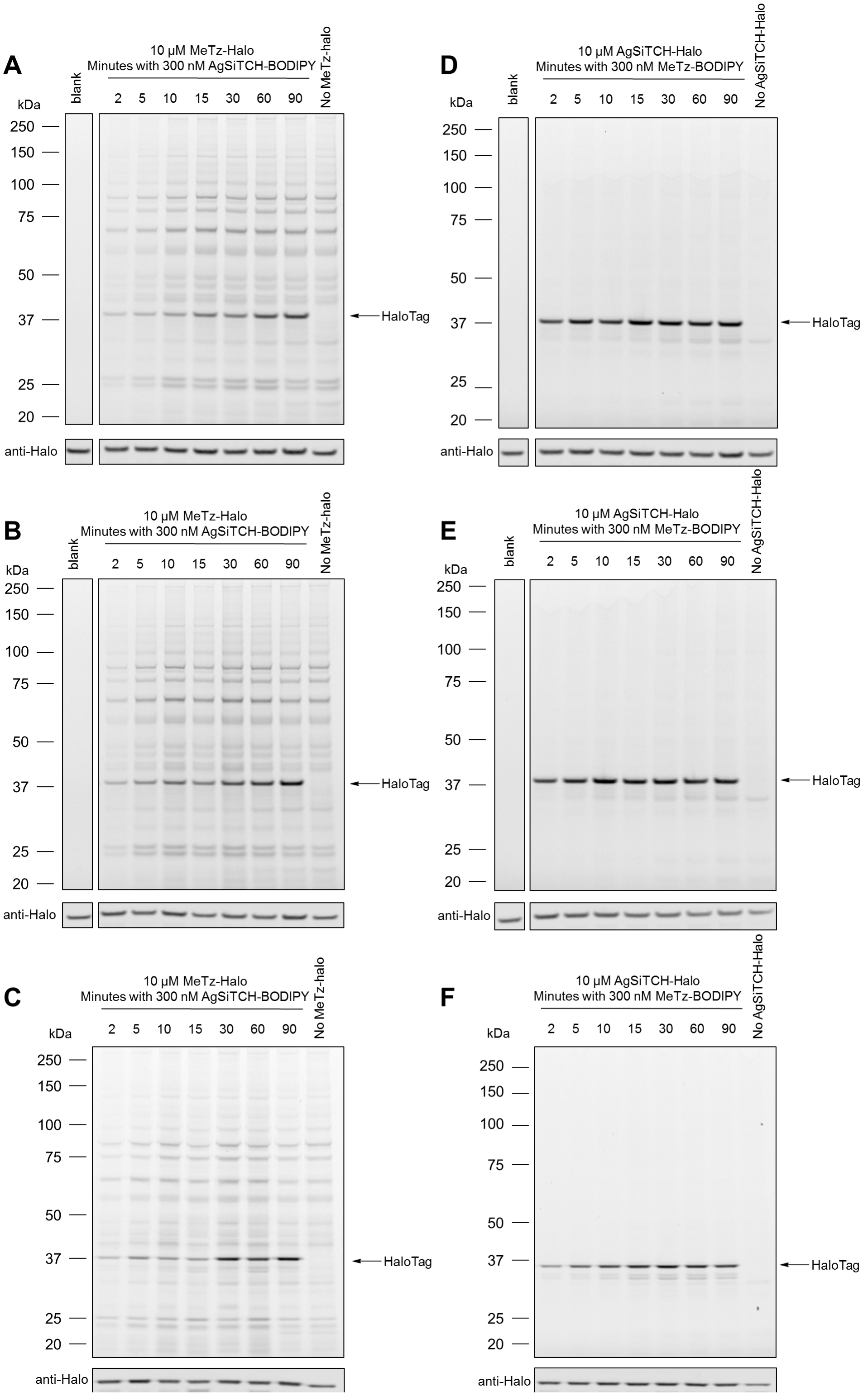

Fig. S26. Full gels for Scheme 8B in the main text. (A-C) Three biological replicates of treatment with 10 µM MeTz-Halo for 30 min, followed by treatment with 300 nM AgSiTCH-BODIPY for the indicated time. (D-F) Three biological replicates of treatment with 10 µM AgSiTCH-Halo for 30 min, followed by treatment with 300 nM MeTz-BODIPY for the indicated time. Arrows indicate the HaloTag band.

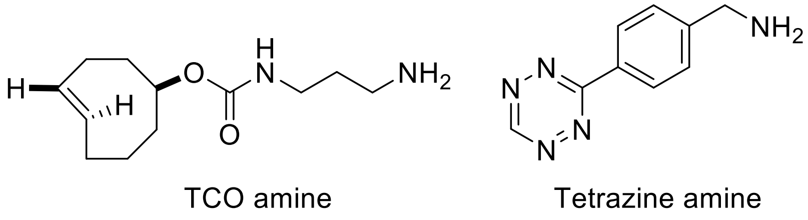

Fig. S27. Structures of reagents for quenching bioorthogonal reactions in live cells.

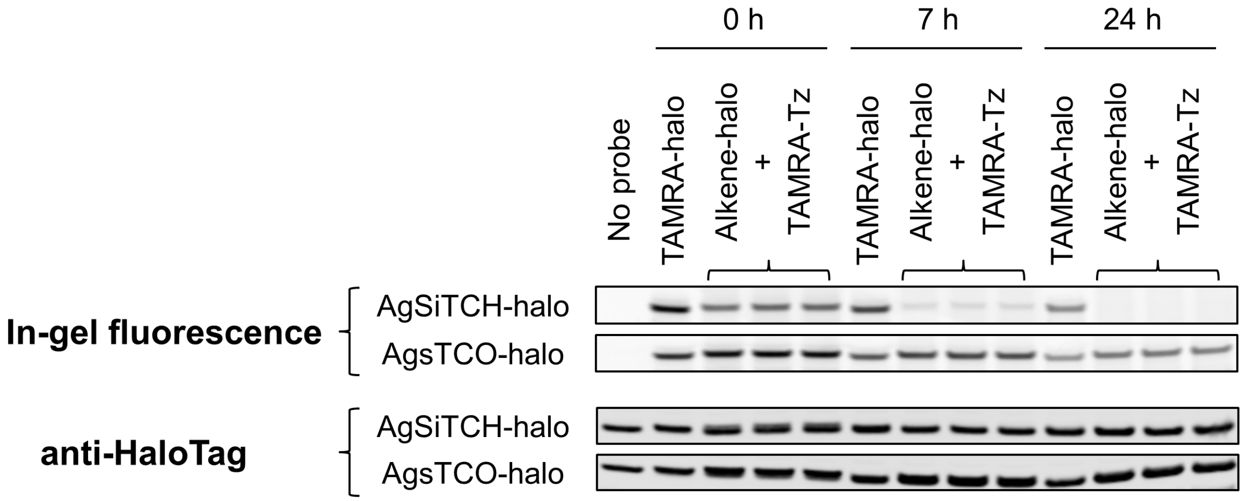

Fig. S28. In-gel fluorescence and Western blot data for Scheme 9B in the main text. Cellular stability of **Ag-sTCO-Halo** and **AgSiTCH-Halo**. In-gel fluorescent intensities were normalized by the corresponding Western blot signals, and were subsequently normalized by the value of **TAMRA-Halo** at time 0. Three biological replicates were done for bioorthogonal reactions. Data were plotted as mean ± SEM.

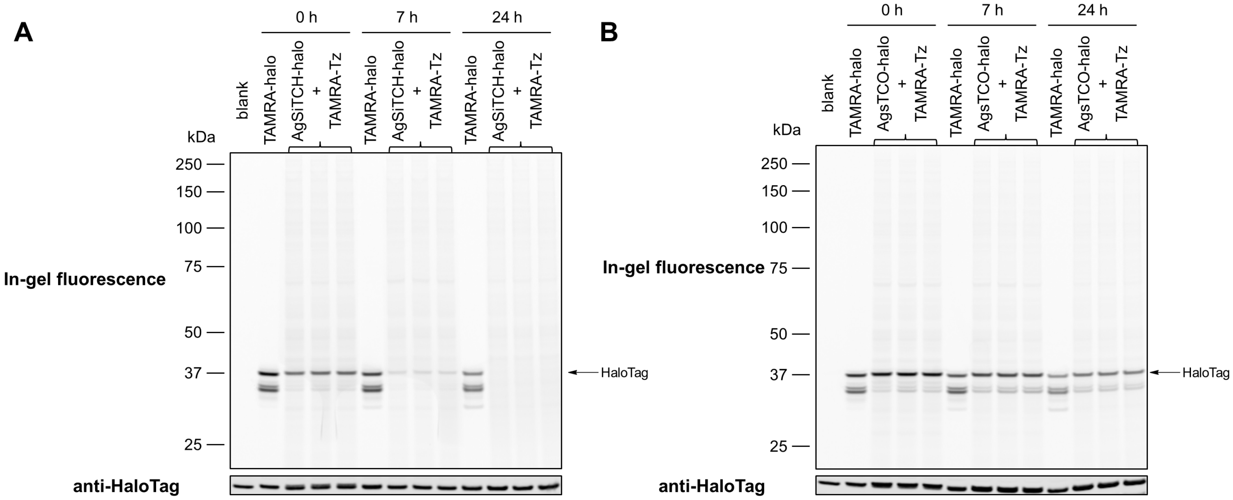

Fig. S29. Full gel images for of **Fig. S28**.

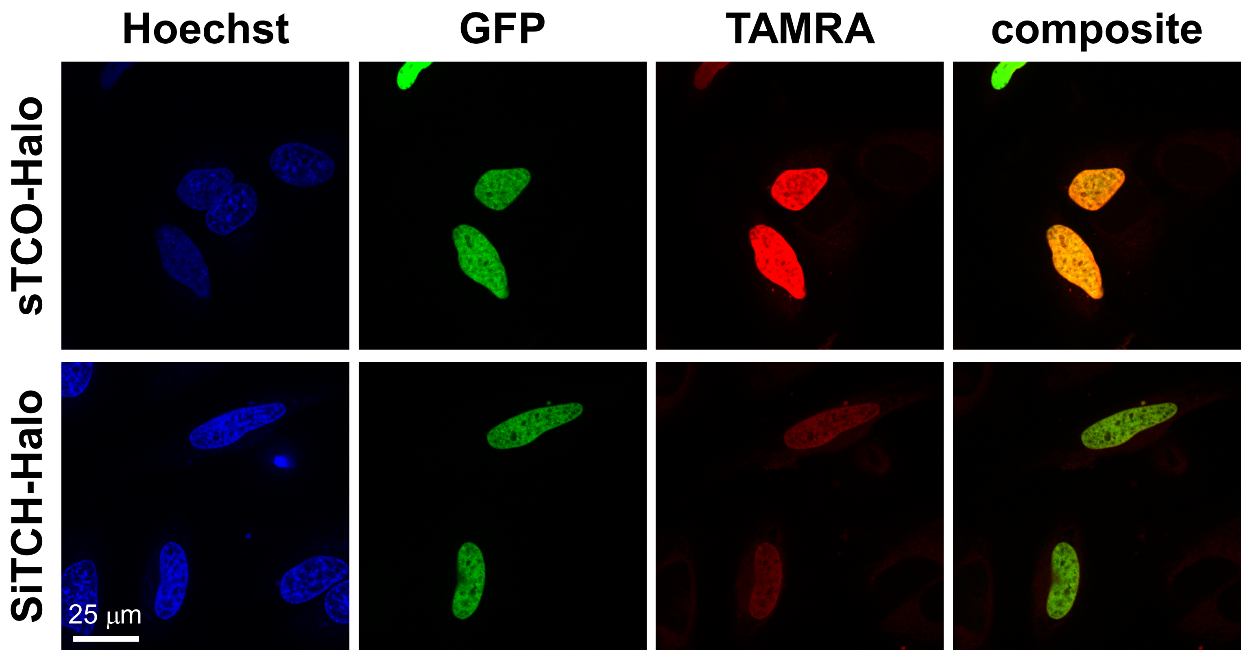

Fig. S30. Larger images for Scheme 9C in the main text.

Materials. The pHTN HaloTag CMV-neo vector was purchased from Promega. The molecular cloning of Halo-H2B-GFP vector was reported previously[6]. Polyethylenimine (PEI) was purchased from Polysciences, Inc.. Lipofectamine 2000 was purchased from Thermo Fisher Scientific. *Trans*-cyclooctene (TCO) amine and tetrazine amine were purchased from Click Chemistry Tools. MeTz-BODIPY and TAMRA-Tz were synthesized according to literature protocol[6]. Phosphate-based saline (PBS) was purchased from Mediatech, Inc.. Media and supplements for cell culture were purchased from Thermo Fisher Scientific unless otherwise noted. All reagents for cell treatment were prepared as 1000× solutions in anhydrous DMSO and aliquots were stored at -20°C.

Cell Culture and Transfection. HEK 293T cells were cultured in high-glucose Dulbecco's Modified Eagle Medium (DMEM) supplemented with 10% (v/v) heat-inactivated fetal bovine serum (certified), 1% (v/v) Glutamax Supplement, and 100 units/mL of penicillin-streptomycin in a humidified incubator with 5% CO_2 at 37°C. The day before transfection, cells were plated in poly-D-lysine coated 6-well plates at a density of $2–5×10^5$ cells per well. For transfection of cells in each well, 3 μg of PEI was diluted to 125 μL of serum-free DMEM, and 1 μg of plasmid was diluted to the same volume of serum-free DMEM. The two fractions were combined, and incubated at room temperature for 20 min. The PEI/plasmid complex was added to the wells, and cells were incubated at 37°C for 24 hours before treatment. HeLa cells were cultured in high-glucose Dulbecco's Modified Eagle Medium (DMEM) supplemented with 10% (v/v) heat-inactivated fetal bovine serum (certified) and 100 units/mL of penicillin-streptomycin in a humidified incubator with 5% CO_2 at 37°C. HeLa cells were transfected with lipofectamine 2000 (Thermo Fisher Scientific) according to manufacturer's protocol (details described below).

Evaluation of AgSiTCH Bioorthogonal Reactions in Live Cells. For labeling with HaloTag ligands, HEK 293T cells transfected with the pHTN HaloTag CMV-neo vector were incubated with 10 μM of MeTz-Halo or AgSiTCH-Halo in 1 mL of culture media for 30 min at 37°C. Excess HaloTag ligands were removed by replacing the media with 1 mL of fresh culture media twice, and cells were incubated for 30 min each time. The media were replaced with 1 mL of culture media containing 300 nM BODIPY fluorophores (AgSiTCH-BODIPY or MeTz-BODIPY), and the reaction between SiTCH and methyl tetrazine was allowed to proceed at 37°C for 1, 2, 5, 15, 30, 60, and 90 min. To quench the reaction, 100 μM TCO amine (for quenching MeTz-Halo) or tetrazine amine (for quenching SiTCH-Halo) were added to the cells. Separately, control cells were treated with 300 nM BODIPY-halo in 1 mL of culture media for 30 min at 37°C. Cells were scrapped and pelleted by centrifuging at 10,000×g at 37°C for 1 min. The buffer was removed and pellets were stored at -80°C until further analysis.

In-Gel Fluorescence and Western Blot. To the cell pellets were added 100 μL of phosphate-buffered saline (PBS) containing 0.25% sodium dodecyl sulfate (SDS), and the cells were lysed with sonication. The protein concentration was determined using bicinchoninic acid (BCA) assay (Thermo Fisher Scientific). The lysates were normalized by concentration, combined with NuPAGE sample reducing agent (Thermo Fisher Scientific) and lithium dodecyl sulfate (LDS) sample buffer (Thermo Fisher Scientific), and analyzed with 1.0 mm thick NuPAGE 4-12% bis-tris 15-well protein gels in 2-[N-morpholino]ethanesulfonic acid (MES) running buffer (Thermo Fisher Scientific). The gels were scanned on a Typhoon FLA 9500 Biomolecular Imager (GE Healthcare) with 473 nm laser excitation and 530±10 nm band pass emission filter. The gels were then transferred to a nitrocellulose membrane with an iBlot 2 dry blotting system (Thermo Fisher Scientific). The membranes were blocked with Odyssey tris-buffered saline (TBS) blocking buffer (LI-COR) at room temperature for 1 h, incubated with an anti-HaloTag polycolonal antibody (1:1,000, Promega, G9281) in the blocking buffer at 4°C overnight. They were then washed 3 times with TBS containing 0.1% tween-20, incubated with an IRDye 800CW goat anti-rabbit IgG antibody (LI-COR) at room temperature for 1 h, and washed 3 times with TBS containing 0.1% tween-20. The blots were imaged with a LI-COR Odyssey CLx imaging system.

Data Analysis and Quantification. In-gel fluorescence data were processed with ImageJ (v1.47, NIH) software. Western blot data were processed with Image Studio Lite (v4.0.21, LI-COR). Both sets of data were quantified with Image Studio Lite with background subtraction. In-gel fluorescence intensities were normalized by the corresponding HaloTag Western blot values. Data were plotted in Prism (v7.02,

GraphPad software Inc.) as mean ± SEM from three biological replicates. Timecourse data were fit with one phase decay function, where Y = (Y_0 - Plateau)*exp(-K*X) + Plateau.

Evaluation of Incorporation Percentage of HaloTag Ligands. HEK 293T cells transfected with the pHTN HaloTag CMV-neo vector in 6-well plates were pulse labeled with 0.3, 1, or 10 μM MeTz-Halo or AgSiTCH-Halo at 37°C for 30 min. Excess HaloTag ligand was removed by replacing the media with fresh growth media and incubating the cells at 37°C for 1 h. Cells were then chase labeled with 1 μM BODIPY-Halo at 37°C for 30 min. The cells were harvested, lysed and analysed by in-gel fluorescence and Western blot as described above.

Stability of SiTCH and sTCO in Live Cells. HEK 293T cells transfected with the pHTN HaloTag CMV-neo vector in 6-well plates were labeled with 10 μM AgSiTCH-Halo or Ag-sTCO-Halo at 37°C for 30 min. Control cells were incubated with 2 μM TAMRA-Halo at 37°C for 30 min. Excess HaloTag ligands were removed by replacing the media with 1 mL of fresh culture media twice, and cells were incubated for 30 min each time. After 0, 7, and 24 h, the media were replaced with 1 mL of culture media containing 2 μM TAMRA-Tz, and the reaction was allowed to proceed at 37°C for 1 h. The cells were harvested, lysed and analysed by in-gel fluorescence and Western blot as described above, with the modification that for in-gel fluorescence gels were scanned with 532 nm laser excitation and ≥575 nm long pass emission filter. For data analysis, in-gel fluorescence intensities were normalized by the corresponding HaloTag Western blot values, and the intensities at 0 h were defined as 1. Data were plotted as mean ± SEM from three biological replicates.

AgSiTCH Bioorthogonal Reactions for Live Cell Imaging. Cells were treated according to a reported protocol[6]. Briefly, HeLa cells were plated in 12-well poly-D-lysine coated glass bottom dishes (MatTek Corporation), and incubated overnight prior to transfection. Cells were transfected with 0.5 μg of Halo-H2B-GFP plasmid and 1.5 μg of lipofectamine 2000 (Thermo Fisher Scientific) according to manufacturer's protocol. On the next day, cells were treated with 10 μM of AgSiTCH-Halo or Ag-sTCO-Halo in 0.5 mL of culture media for 30 min at 37°C. Excess HaloTag ligands were removed by replacing the media with 0.5 mL of fresh culture media twice, and cells were incubated for 30 min each time. The cells were treated with 2 μM TAMRA-Tz for 5 min at 37°C, and the reactions were quenched with 100 μM TCO amine for 10 min. The cells were washed with 0.5 mL of fresh media for 1–3 hours, with 2–3 media changes. Prior to imaging, 8 μM of Hoechst 33342 was added to stain the nucleus.

Fluorescence Microscopy. Live cell images were acquired on a Zeiss Axio Observer.Z1 confocal microscope with a Yokagawa CSU-X1M 5000 spinning disk system and a Photometrics Evolve 512 Delta EM CCD camera. Zeiss Plan-Apochromatic 63×/1.4 oil immersion objectives were used. The microscope was equipped with an on-stage humidified chamber maintaining the cells at 37°C and 5% CO_2. Hoechst was excited with a 405 nm laser, and emission signals were collected between 440–480 nm. GFP was excited with a 488 nm laser, and emission signals were collected between 520–550 nm. TAMRA was excited with a 561 nm laser, and emission signals were collected between 620–670 nm. The microscope was operated with ZEN 2 (Blue edition) v.2.0.0.0 software (Carl Zeiss Microscopy). Images were processed in Fiji (ImageJ v1.50e, NIH) software.

Stability of 7b in 2-mercaptoethanol in MeOD-d_4

At 22 °C.

To a solution of **7b** (5.8 mg, 0.030 mmol, with 9% *cis* isomer) in MeOD-d_4 (1 ml) was added 2-mercaptoethanol (2.0 μL, 0.030 mmol) and 1,3,5-trimethoxylbenzene (1.6 mg, 0.01 mmol, as an NMR internal standard). The solution was transferred to an NMR tube. Another NMR sample without 2-mercaptoethanol was prepared as a control. The solutions were stored at room temperature (22 °C) and were monitored by ^{1}H NMR to observe the isomerization of **7b** to (*Z*)-*Si*-(3-cyanopropyl)-*Si*-methyl-5-sila-cycloheptene. After 2 hours, 3% of **7b** remained for the sample where mercaptoethanol was added. For the control experiment, 98% of **7b** remained after 2 h. Results were plotted using Prism software (V. 7.00, Graphpad Software Inc, Fig.S32).

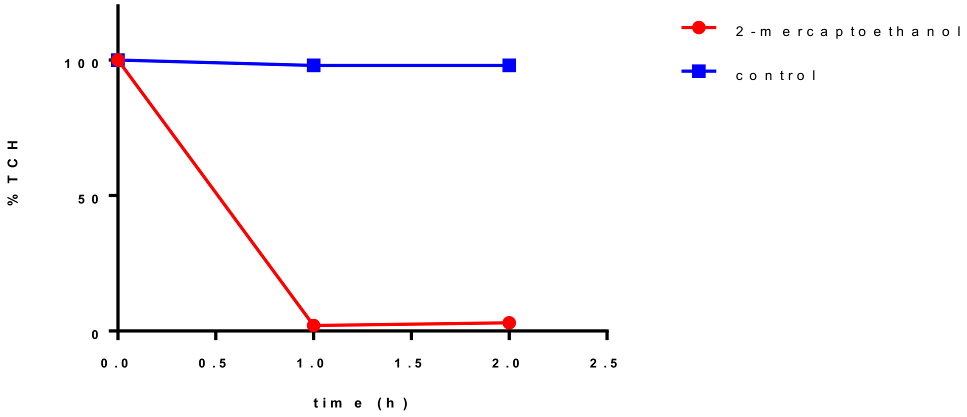

Fig. S31. Stability profile of **7b** (30 mM) in the presence or absence of 2-mercaptoethanol (30 mM) in MeOD-d_4 at room temperature (22°C).

At –17 °C.
To a solution of **7b** (5.8 mg, 0.030 mmol, with 7 % *cis* isomer) in MeOD-d_4 (1 mL) was added 2-mercaptoethanol (2.0 μL, 0.030 mmol) and 1,3,5-trimethoxylbenzene (1.6 mg, 0.01 mmol, as an NMR internal standard). The solution was transferred to an NMR tube. Another NMR sample without 2-mercaptoethanol was prepared as a control. The solutions were stored at –17 °C and were monitored by ^{1}H NMR to observe the isomerization of **7b** to (*Z*)-*Si*-(3-cyanopropyl)-*Si*-methyl-5-sila-cycloheptene. After 24 hours, 53% of **7b** remained. While for the control experiment, 97% of **7b** remained. Results were plotted using Prism software (V. 7.00, Graphpad Software Inc, Fig. S32).

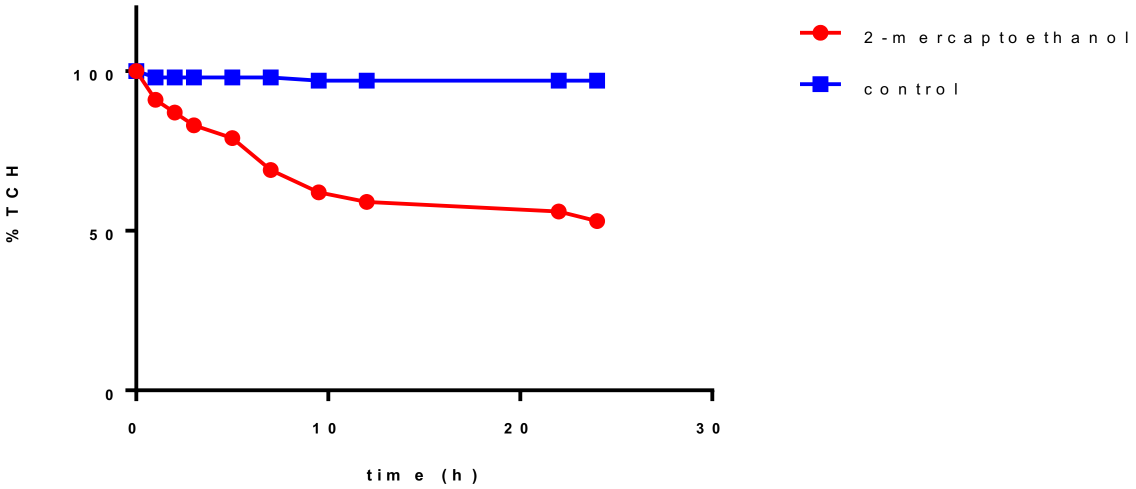

Fig. S32. Stability profile of **7b** (30 mM) in the presence or absence of 2-mercaptoethanol (30 mM) in MeOD-d_4 at -17°C

References

1) Royzen, M.; Yap, G. P. A.; Fox, J. M. *J. Am. Chem. Soc.* **2008,** 130(12), 3760-3761

2) Matsuo, J.; Sasaki,S.; Hoshikawa, T.; Ishibashi, H. *Chem. Commun.*, **2010**,46, 934-936

3) Lombardi, P. *Chemistry & Industry* **1990,** 708

4) Panne, P.; Fox, J. M. *J. Am. Chem. Soc.* **2007**, 129(1), 23 (Supporting information)

5) Los, G. V.; Encell, L. P.; McDougall, M. G.; Hartzell, D. D.; Karassina, N.; Zimprich, C.; Wood, M. G.; Learish, R.; Ohana, R. F.; Urh, M.; Simpson, D.; Mendez, J.; Zimmerman, K.; Otto, P.; Vidugiris, G.; Zhu, J.; Darzins, A.; Klaubert, D. H.; Bulleit, R. F.; Wood, K. V. *ACS Chem. Biol.* **2008**, 3, 373

6) Murrey, H. E.; Judkins, J. C.; Am Ende, C. W.; Ballard, T. E.; Fang, Y.; Riccardi, K.; Di, L.; Guilmette, E. R.; Schwartz, J. W.; Fox, J. M.; Johnson, D. S. *J. Am. Chem. Soc.* **2015**, 137(35), 11461

7) Lang, K.; Davis, L.; Torres-Kolbus, J.; Chou, C.; Deiters, A.; Chin, J. *Nat. Chem.* **2012**, 4, 298
